# RANKL blockade for erosive hand osteoarthritis: a randomized placebo-controlled phase 2a trial

**DOI:** 10.1038/s41591-024-02822-0

**Published:** 2024-02-15

**Authors:** Ruth Wittoek, Gust Verbruggen, Tine Vanhaverbeke, Roos Colman, Dirk Elewaut

**Affiliations:** 1 Department of Rheumatology, Ghent University Hospital, Ghent University, Ghent, Belgium; 2 VIB Center for Inflammation Research, Ghent University, Ghent, Belgium; 3 Biostatistics Unit, Faculty of Medicine and Health Sciences, Ghent University, Ghent, Belgium

**Keywords:** Randomized controlled trials, Osteoimmunology

## Abstract

Erosive hand osteoarthritis (OA) is a prevalent and disabling disease with limited treatment options. Here we present the results of a monocentric, placebo-controlled, double-blind, randomized phase 2a clinical trial with denosumab, a receptor activator of nuclear factor-κB ligand inhibitor, evaluating the effects on structure modification in erosive hand OA. Patients were randomized to 48 weeks treatment with denosumab 60 mg every 3 months (*n* = 51, 41 females) or placebo (*n* = 49, 37 females). The primary (radiographic) endpoint was the change in the total Ghent University Scoring System (GUSS) at week 24, where positive changes correspond to remodeling and negative changes to erosive progression. Secondary endpoints were the change in the GUSS at week 48 and the number of new erosive joints at week 48 by the anatomical phase scoring system. Baseline mean GUSS (standard deviation) of target joints was 155.9 (69.3) in the denosumab group and 158.7 (46.8) in the placebo group. The primary endpoint was met with an estimated difference between groups of 8.9 (95% confidence interval (CI) 1.0 to 16.9; *P* = 0.024) at week 24. This effect was confirmed at week 48 (baseline adjusted GUSS (standard error of the mean) denosumab and placebo were 163.5 (2.9) and 149.2 (3.9), respectively; with an estimated difference between groups of 14.3 (95% CI 4.6 to 24.0; *P* = 0.003)). At patient level, more new erosive joints were developed in the placebo group compared with denosumab at week 48 (odds ratio 0.24 (95% CI 0.08 to 0.72); *P* = 0.009). More adverse events occurred in the placebo group (125 events in 44 patients (90%)) compared with the denosumab group (97 events in 41 patients (80%)). These results demonstrate that denosumab has structure modifying effects in erosive hand OA by inducing remodeling and preventing new erosive joints. EU Clinical Trials Register identifier 2015-003223-53.

## Abstract (web summary)

Denosumab, a receptor activator of nuclear factor-κB ligand inhibitor, previously developed for osteoporosis, was shown to be effective at inducing structural modification in patients with erosive hand osteoarthritis after 24 weeks.

## Main

The radiographically erosive type of hand osteoarthritis (OA) affecting the interphalangeal (IP) finger joints is a highly prevalent, predominantly female disease^1–5^ and frequently considered as the more inflammatory subtype of hand OA^6^. It is characterized by a high burden of disease^7,8^. Currently, existing therapies only alleviate symptoms^9^ whilst not attenuating nor arresting structural damage that contributes largely to functional limitations and ultimately results in considerable disability and chronic pain^2,10–12^.

Radiographic hallmarks are the resorption of articular cartilage, which usually precedes osteolytic changes in the subchondral bone and collapse of the subchondral bony endplate. Articular tissue destruction is followed by reparative features, such as remodeling of the subchondral bony plate and the formation of bony nodules at the margins of the affected joints. Erosive features and signs of remodeling can occur simultaneously in the same patient, causing the disease to be active up to several decades until all joints progress to the terminal phase of remodeling^13,14^. Recent studies have demonstrated that individuals who develop erosive hand OA have thinner bones before its development and lose more bone and cartilage (even in joints without OA) as the disorder progresses^15^. Earlier studies already found relationships between bone loss and hand OA progression^16,17^. These findings suggest that erosive hand OA, in contrast to other types of OA, is rather associated with musculoskeletal frailty and are moving the field away from viewing erosive hand OA as a cartilage disease to rather a systemic bone disease. Histological studies in patients with erosive hand OA demonstrated osteoclast activity with resorptive lacunae in bone^18^, which is mirrored by increased serum levels of markers of bone resorption, such as C-telopeptide of type I collagen, suggesting an important degree of osteoclast activation^1,19^.

Denosumab, a fully human monoclonal antibody, inhibits bone resorption by binding to a receptor activator of nuclear factor-κB ligand (RANKL) and preventing it from activating receptor activator of nuclear factor-κB on bone and cartilage resorbing cells^20^. It is currently used for treatment of osteoporosis and cancer-associated bone loss. Proof-of-concept studies demonstrated its ability to delay erosive disease progression in rheumatoid arthritis patients, irrespective of disease activity control^21^ and a clear dose-dependant relationship with erosive inhibition was shown^22,23^.

**Extended Data Fig. 1:**
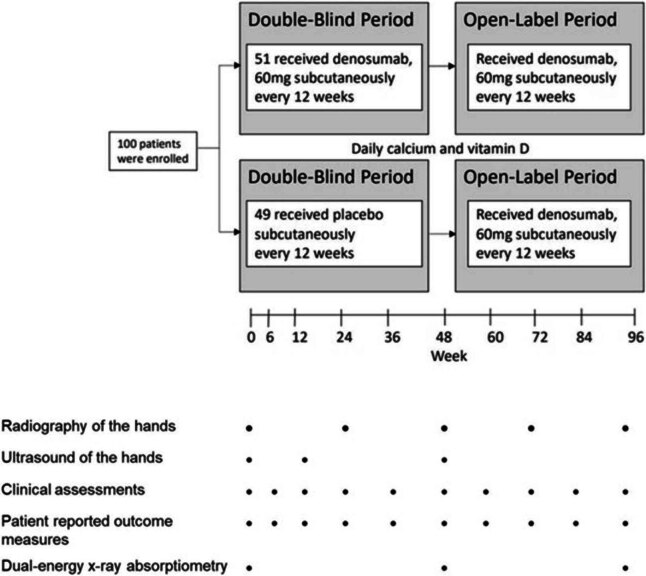
Trial regimen and assessments. Overview of the assessments performed during the double-blind and open-label period of the study.

In this Article, we aim to demonstrate with a proof-of-concept study that denosumab slows down progression of structural damage in erosive hand OA and prevents the development of new erosive joints. Given the mode of action, structure modification was deliberately chosen as a single primary outcome, while clinical and patient-reported outcome measures were only considered exploratory endpoints (Extended Data Fig. 1). Erosive hand OA is a heterogeneous disease, and therefore, patients with inflammatory activity (clinically and by ultrasound) in at least one IP joint in the loss of joint space (J) or subchondral erosion(s) (E) phase, according to the Verbruggen and Veys anatomical phase scoring system, were found eligible^13^. Patients were given the opportunity to enter an open-label extension study at week 48, where all patients were treated with denosumab 60 mg every 3 months. Since earlier studies in rheumatoid arthritis revealed dose-dependent structural inhibitory capacities of denosumab^22,23^, we anticipated to use a higher dosing interval than approved for use in osteoporosis. Safety was closely monitored through the entire study.

## Results

### Patients

**Fig. 1:**
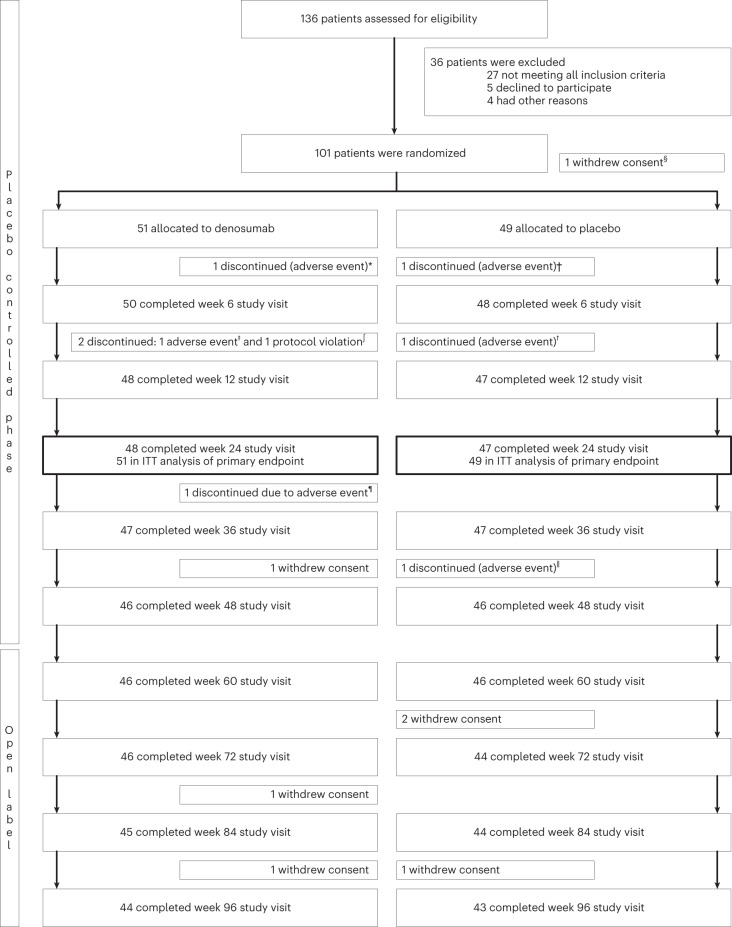
Trial schema. ^§^One eligible patient was randomized but did not receive any medication (patient withdrew consent before any medication was administered). Therefore, the designated randomization number was not used, and a 26th block had to be addressed for the final patient included, explaining the incomplete balance. All data from this patient were excluded from all analyses. *Acute coronary syndrome (a serious adverse event); ^†^breast carcinoma (a serious adverse event); ^‡^subjective calcium/vitamin D intolerance; ^∫^use of oral corticosteroids; ^¶^urticarial reaction; ^ǁ^pancreas carcinoma (a serious adverse event).

We screened patients for enrollment between 16 March 2016 and 10 July 2018. Patients completed baseline visits and started treatments between 30 March 2016 and 25 July 2018, and the last study visit of the last patient in the placebo-controlled phase was 3 April 2019. Of 136 patients assessed for eligibility, 36 (26%) were excluded (predominantly because of absence of radiographic J or E joint and/or absence of clinical and sonographic inflammation), and 100 (74%) patients were randomized and received at least one administration of study medication (Fig. 1). One eligible patient was randomized but did not receive any medication (patient decided to withdraw consent before any medication was administered). Therefore the designated randomization number was not used, and a 26th block had to be addressed for the final patient included, explaining the incomplete balance. A total of 51 patients (51%, 41 female) were assigned to denosumab, and 49 (49%, 37 female) were assigned to placebo and were included in the intention-to-treat (ITT) analysis of the primary endpoint. Five patients dropped out before week 24 due to adverse events, including three patients in the denosumab group (one acute coronary syndrome before week 6, one intolerance to oral calcium and vitamin D supplementation and one protocol deviation) and two patients in the placebo group (two new diagnosis of breast cancer). A total of 46 (90%, 37 female) patients in the denosumab and 46 (94%, 34 female) in the placebo group completed the 48-week study. Demographic and baseline characteristics were well balanced between groups (Table 1 and Extended Data Table 1), and 182 target joints were selected for ITT analysis of the primary outcome. All joints (*n* = 1590) were analyzed for secondary imaging endpoint analyses. Inter- and intrareader reliability analyses of the radiographic scores by two radiographic scoring systems, that is, the Ghent University Scoring System (GUSS) and the anatomical scoring system by Verbruggen and Veys, were performed and found excellent (Table 2).

**Table 1:** Demographic and clinical characteristics of the patients at baseline

Characteristics	Denosumab (*n* = 51)	Placebo (*n* = 49)
Age (years)	62.0 (7.7)	60.6 (7.9)
Female sex (no. (%))	41 (80)	37 (76)
Disease duration (years)	6.3 (6.6)	6.0 (6.4)
Body mass index^a^	25.3 (3.5)	25.3 (4.0)
NRS pain^b^	4.7 (2.5)	4.8 (2.7)
AUSCAN pain^c^	20.7 (11.6)	21.7 (13.0)
AUSCAN function^d^	41.3 (21.9)	42.0 (24.9)
FIHOA^e^	10.4 (0.9)	10.3 (1.0)
Mean GUSS^f^ of target joints	155.9 (69.3)	158.7 (46.8)
Anatomical phase according to Verbruggen and Veys (no. (%))^g^		
Normal joints (N phase)	196 (24.3)	150 (19.2)
Stable or stationary joints (S phase)	326 (40.4)	353 (45.1)
Joints with loss of joint space (J phase)	67 (8.3)	82 (10.5)
Erosive joints (E phase)	98 (13.3)	104 (13.3)
Remodeled joints (R phase)	107 (13.3)	91 (11.6)
Fused joints (F phase)	3 (0.4)	3 (0.4)
Number of affected joints (of 16 joints)^h^	3.6 (2.2)	4.0 (2.2)
Number of target joints [range]	1.6 (1.0) [1–5]	1.8 (1.1) [1–5]
Presence of ≥1 joint in E phase (no. (%))^i^	44 (86)	43 (88)

Data are mean (standard deviation) or number (%). Unadjusted *P* values were determined with the use of chi-square tests for categorical variables and *t*-test for continuous variables. No significant differences were found for any of the variables among the treatment groups at baseline.

^a^The body mass index is the weight in kilograms divided by the square of the height in meters.

^b^The numeric rating scale (NRS) pain is a scale from 0 to 10, with higher scores indicating greater severity.

^c^Scores of the AUSCAN subscale pain range from 0 to 50, with higher scores indicating more pain^37^.

^d^Scores of AUSCAN subscale function range from 0 to 90, with higher scores indicating more disability^37^.

^e^Scores of the FIHOA range from 0 to 30, with higher scores indicating more disability^36^.

^f^The GUSS ranges from 0 to 300 (ref. ^14^). This scoring system is composed of three subdomains: subchondral plate, subchondral bone and joint space. Specific features referring to the underlying pathology of the disease are being scored on a numerical scale from 0 to 100, with increments of 10. Higher scores indicate remodeling or repair. Thus, the maximum score refers to either a normal or a completely restored (that is, nonerosive) joint. Lower scores indicate presence of more or greater erosions, loss of joint space or subchondral plate^14^. The total score per joint is made by an equally weighted sum score of all three subdomains (minimum 0, maximum 300). Mean GUSS value of 16 joints per patient is shown. Smallest detectable change, after intensive training, was reduced to 10 units.

^g^The Verbruggen and Veys anatomical score system differentiates normal joints (N) from pre-erosive phases (S phase, that is, a stationary phase with minimal degenerative features, such as subchondral sclerosis, joint space narrowing and presence of small osteophytes, and J phase with partial or complete loss of joint space), the erosive phase (E) and phases of remodeling (R, that is, signs of repair, such as reappearance of subchondral plate and joint space width, disappearance of erosions at the subchondral bone and development of osteophytes at joint margins, and F, fused joint as extreme sign of remodeling)^13^. The presence of anatomical phases were assessed by the Verbruggen and Veys scoring system on baseline, week 24, 48, 72 and 96 radiographs.

^h^Any radiographically defined S, J, E or R joint, according to the Verbruggen and Veys score.

^i^Patients without E joints at baseline had one or more joints in the J phase to fulfill the inclusion criteria. Besides these, patients showed one or more joints in the R phase, confirming the diagnosis of erosive hand OA.

**Table 2:** Reliability analyses of radiographic readings

Readers	Baseline data					Change scores		
	VV^a^	GUSS				GUSS		
		SC plate	Joint width	SC bone	Total score	Δ Total score baseline—week 24	Δ Total score baseline—week 24	Δ Total score—week 24 to week 48
Intrareader reliability								
Reader 1 (GV)	0.92	0.94	0.96	0.98	0.99	0.82	0.89	0.80
Reader 2 (RW)	0.95	0.92	0.92	0.97	0.95	0.87	0.91	0.82
Interreader reliability								
Reader 1 versus reader 2	0.93	0.99	1.0	0.94	0.99	0.99	0.99	0.99

Inter- and intrareader reliability analyses of the radiographic scores by two radiographic scoring systems, that is, the anatomical scoring system by Verbruggen and Veys^13^ and the GUSS^14^. Scores of subdomains are shown for baseline data, and change of the total scores are shown for longitudinal data. Data shown are intraclass coefficients of correlation by two-way mixed, absolute agreement, average measures or stated if otherwise for the GUSS from the first 20 patients (accounting for 320 joints). Repeated readings were performed with an interval of minimally 1 month.

^a^Weighted kappa statistics from baseline data shown.

VV, anatomical phase scoring system by Verbruggen and Veys; SC, subchondral; Δ, change.

### Primary endpoint

**Fig. 2:**
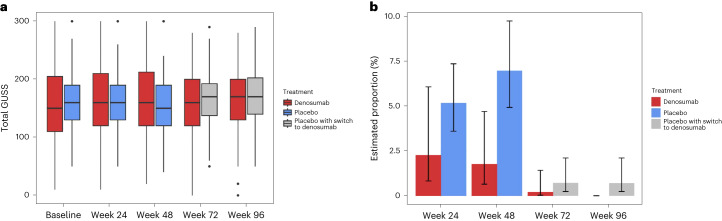
Radiographic changes: total GUSS scores and new erosive joints at weeks 24, 48, 72 and 96. **a**, Box plots of total GUSS scores of target joints during the placebo-controlled phase (baseline until week 48) and the extension phase (week 48 until week 96) showing the Q1, median and Q3, with whiskers extending to ±1.5 × IQR (where IQR represents the interquartile range). *N* *=* 51 patients in the denosumab group and *N* *=* 49 patients in the placebo group. **b**, Bar plots with 95% CI of the estimated percentages of new erosive joints of potential pre-erosive joints (that is, N, S and J). Week 72 and 96 data originate from post-hoc analyses of the open-label extension phase. Similar GEE logistic regression models were used with treatment groups based on the initial randomization code.

**Extended Data Fig. 2:**
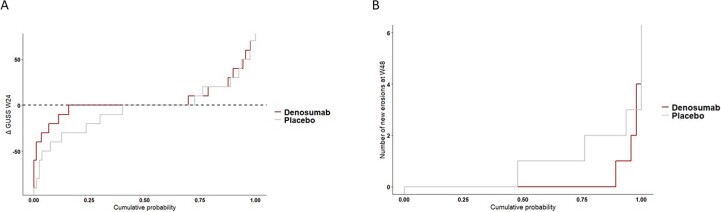
Cumulative probability plots of radiographic changes. Cumulative probability plots showing (**a**) the radiographic changes by GUSS at week 24 and (**b**) new erosions at week 48 in the denosumab (n = 51) and placebo (n = 49) treated group.

The baseline adjusted mean (standard error of the mean) GUSS at week 24 was 162.2 (2.4) in the denosumab group and 153.3 (3.2) in the placebo group, with an estimated difference between groups of 8.9 (95% confidence interval (CI) 1.0 to 16.9; *P* = 0.024; Table 3 and Fig. 2a). This effect was confirmed at week 48 (baseline adjusted GUSS (standard error of the mean), where denosumab and placebo were 163.5 (2.9) and 149.2 (3.9), respectively, with an estimated difference between groups of 14.3 (95% CI 4.6 to 24.0; *P* = 0.003)). Cumulative probability plots of the radiographic changes are shown in Extended Data Fig. 2.

**Table 3:** Results of the primary and secondary endpoint analyses

ENDPOINT	Denosumab (*n* = 51)	Placebo (*n* = 49)	Difference between groups (95% CI)	*P* value
PRIMARY ENDPOINT				
GUSS at week 24	162.2 (2.4)	153.3 (3.2)	8.9 (1.0 to 16.9)	**0.024**
SECONDARY ENDPOINTS				
New erosive joints at week 24 (no. (%))	12 (2.3)	29 (5.1)	OR 0.43 (0.1 to 1.3)	0.13
New erosive joints at week 48 (no. (%))	9 (1.8)	38 (7.0)	OR 0.24 (0.1 to 0.7)	**0.009**
GUSS at week 48	163.5 (2.9)	149.2 (3.9)	14.3 (4.6 to 24.0)	**0.003**

Values are least squares mean ± standard error of the mean, unless otherwise stated. Comparisons between groups was done by the generalized estimation equations at the patient level in the ITT population. Missing data were imputed according to a predefined imputation model for the primary endpoint and by baseline observations for the secondary endpoints. No correction for multiple comparison was done, since there was only one primary endpoint. The *P* values in bold represent statistical significance (<0.05).

no., number.

### Secondary radiographic endpoint

**Extended Data Fig. 3:**
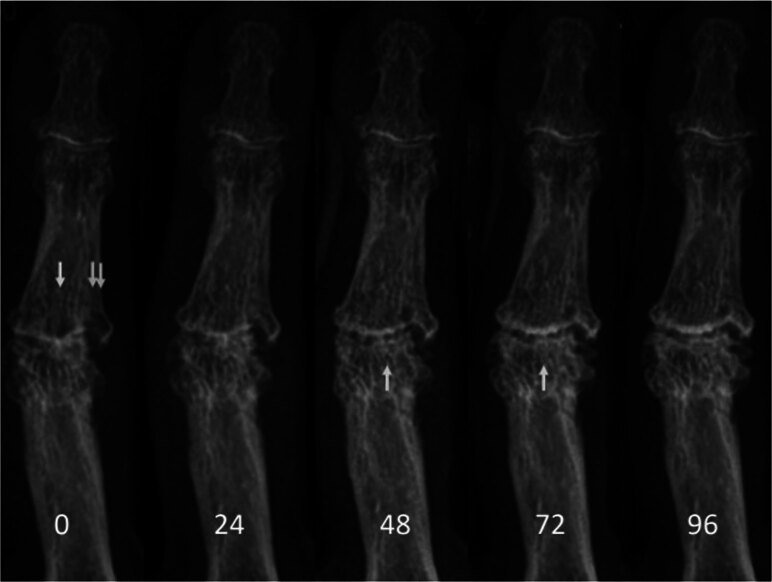
Longitudinal images of an affected joint. Serial images of a PIP joint in a patient treated with denosumab during the entire trial: joint entering in E phase at baseline; Presence of two subchondral erosions, indicated at the base of the mid phalanx (↓) and (↓↓)). Already clear signs of repair are seen after 24 weeks. At 48 weeks, the joint has entered the R phase with obvious repair of the lesions, reappearance of the subchondral bony plate at the top of the proximal phalanx (↑) and clear alignment of a new joint space. Complete repair is seen at week 72 and 96. PIP: proximal interphalangeal.

At patient level, the development of new erosive joints was statistically higher in the placebo group compared with denosumab at week 48 (38 (7.0%) new E joints out of 535 nonerosive joints in placebo versus 9 (1.8%) new E joints out of 501 nonerosive target joints in denosumab; Fig. 2b). From baseline to week 48, the estimated odds ratio (OR) for erosive progression was 76% lower in the denosumab group compared with placebo (OR 0.24 (95% CI 0.08 to 0.72); *P* = 0.009; Table 3). Three new erosive joints in the denosumab group at week 24 already disappeared and remodeled at week 48. Radiographic changes are shown (Extended Data Fig. 3).

### Exploratory endpoints

Change in pain numerical rating scale (NRS) at week 24 versus baseline did not differ significantly between placebo and denosumab (estimated difference between groups is −0.3 (95% CI −1.6 to 0.7), *P* = 0.42). This also accounts for Functional Index for Hand Osteoarthritis (FIHOA) and Australian–Canadian Hand Osteoarthritis Index (AUSCAN) function: −0.9 (95% CI −3.5 to 1.8; *P* = 0.52) and −1.4 (95% CI −11.1 to 8.3; *P* = 0.78). At week 48, pain nor function improved significantly compared with baseline (Extended Data Table 2).

Both the US effusion and US synovitis score decreased significantly in both groups at week 12 and week 48 compared with baseline. A numerically higher decrease in the US effusion score was seen in the denosumab group at week 12 but did not reach statistical significance (*P* = 0.06). The US erosion score did reduce significantly in the denosumab group at week 48 compared with baseline, while this was not the case in the placebo group (*P* = 0.007; Extended Data Table 3).

In this nonosteoporotic population, the mean bone mineral density T scores at the lumbar spine and femoral neck increased from baseline to week 48 in the denosumab group and in the placebo group at the lumbar spine. At 48 weeks, the percentage change from baseline was greater with denosumab compared with placebo at the lumbar spine by 2.8 percentage points (*P* < 0.001; Extended Data Table 3).

### Safety

Through week 48, the incidence of adverse events was higher in the placebo group versus denosumab (125 events in 44 (90%) patients in the placebo group versus 97 events in 41 (80%) patients in the denosumab group; Table 4). Thirteen serious adverse events were reported during the study: six in the denosumab group and seven in the placebo group. Six patients (6%) discontinued the study because of an adverse event: three (6%) in the denosumab group and three (6%) in the placebo group. The most common adverse events were infections and musculoskeletal complaints (in denosumab: *n* = 41 (in 26 (51%) patients) and *n* = 27 (in 21 (41%) patients); in placebo: *n* = 39 (in 22 (45%) patients) and *n* = 34 (in 24 (49%) patients), respectively). Cancer occurred in three patients (6%, all allocated to placebo). Asymptomatic hypocalcemia occurred in five (10%) patients in the denosumab group and in three (6%) in the placebo group (Extended Data Table 4). Three events (obstipation and diverticulitis) were found related to the study medication (all receiving denosumab).

**Table 4:** Summary of safety events through week 48^a^

	Denosumab (*n* = 51)	Placebo (*n* = 49)
Any adverse event (no.)	97	125
Serious adverse event (no.)	6	7
Adverse event leading to discontinuation (no.)	3^b^	3^c^
Adverse event of special interest (no.)		
Cancer	0	3^c^
Infection	41	39
Major cardiovascular event^d^	1	0
Gastrointestinal event	6	7
Surgical and medical procedures	2	9
Musculoskeletal complaints	27	34
Nervous system disorders (including dizziness, vertigo and headache)	4	17
Pulmonary and respiratory complaints (noninfectious)	2	1
Rash and skin problems	3	1
Allergy (systemic and urticaria)	3	1
Teeth problems	3	3
Other^e^	5	10
Hypocalcemia^f^		
At week 12	2	0
At week 24	1	1
At week 36	2	1
At week 48^g^	3	1

^a^Analyses were performed with data from the ITT population.

^b^In the denosumab group, one patient experienced an acute coronary syndrome, one had an urticarial skin reaction and one experienced a subjective intolerance to the calcium and vitamin D administration and, therefore, discontinued the study.

^c^In the placebo group, two patients had breast cancer and one patient had a pancreatic adenocarcinoma with metastases and discontinued the study.

^d^One patient in the denosumab group experienced an acute coronary syndrome 4 weeks after start of the study.

^e^Other adverse events in the denosumab group were as follows: carotid artery stenosis (*n* = 1), menstrual bleeding (*n* = 1), diabetes mellitus type 2 (*n* = 1), general malaise after intake of calcium/vitamin D (*n* = 1), and fatigue (*n* = 1); in the placebo group these were as follows: cerumen impaction (*n* = 1), nervosity/anxiety (*n* = 1), menopausal symptoms (*n* = 1), eye trauma (*n* = 2) and fatigue (*n* = 5).

^f^Hypocalcemia was defined as below 2.12 mmol l^−1^

^g^Hypocalcemia at week 48 was a new finding in one patient and already present in one patient at week 12 in the denosumab group and a new finding in one patient in the placebo group.

### Extension phase

A total of 92 patients (92%, 71 females) entered the extension phase at week 48, of whom 46 originally received denosumab and 46 received placebo. Five (5%) patients prematurely discontinued, and 44 (86%) patients from the original denosumab group and 43 (88%) patients from the initial placebo group ended visit week 96 and were included for the post-hoc analyses. These study visits took place between 3 March 2017 and 27 May 2020. All target joints evolved toward remodeling during this phase (Extended Data Table 5). Total GUSS kept increasing in both groups compared with baseline, with a larger increase in the former placebo group compared with the initial denosumab group (estimated difference between GUSS groups at week 72 was 2.3 (95% CI −2.9 to 6.9; *P* = 0.32) and at week 96 was 3.5 (95% CI −1.1 to 8.1; *P* = 0.13)). Compared with week 48, the GUSS score significantly increased at week 96 in the former placebo group (estimated difference GUSS placebo was 25.7 (95% CI 16.2 to 35.1) versus 9.9 (95% CI −1.3 to 21.1) in denosumab; *P* = 0.035). Concerning clinical exploratory endpoints, patients from the original denosumab group showed statistically significant decreasing pain levels at week 96, compared with baseline and to patients from the initial placebo group (difference between groups NRS pain was −1.0 (95% CI −1.8 to −0.2; *P* = 0.02)). Similar observations were done for FIHOA (difference between the groups was −1.7 (95% CI −3.3 to −0.1; *P* = 0.04)), suggesting clinical benefits with sustained treatment. All values of exploratory endpoints are presented in Extended Data Table 6.

Overall, intake of paracetamol and nonsteroidal anti-inflammatory drugs was low in this study population. The paracetamol intake numerically decreased in the denosumab group during the first year compared with baseline and remained stable in the second year. In the placebo-treated group, paracetamol intake numerically increased after week 6 until week 48 and statistically decreased under active treatment in the second year (*P* = 0.048 at week 72 and *P* = 0.012 at week 96). Nonsteroidal anti-inflammatory drug intake was low in both groups and did not change over time.

### Sensitivity and subgroup analyses

The analysis with the baseline observations replacing missing data for the primary endpoint showed similar results as the primary analysis (an estimated difference between the groups of 8.1 (95% CI 0.8 to 15.3; *P* = 0.026) at weeks 24 and 13.3 (95% CI 4.2 to 22.4, *P* = 0.003) at week 48).

According to the sensitivity analysis based on the three-level linear mixed model, the change between baseline and week 48 differs significantly between placebo and denosumab (an estimated difference between the groups of 13.9 (95% CI 4.1 to 23.7; *P* = 0.007), whereas the change between baseline and week 24 does not reach statistical significance (an estimated difference between the groups of 8.0 (95% CI −1.7 to 17.8; *P* = 0.114).

The interaction between the presence of baseline clinical signs of inflammation (yes/no) and treatment effect on change in GUSS scores was tested and showed no significant interaction between inflammation and treatment at week 24 (*P* = 0.48) nor at week 48 (*P* = 0.18).

A subgroup efficacy analysis performed on an extended group of target joints (*n* = 198; that is, all joints showing any progression to J, E or E/R phase throughout the study that were not defined as J or E phase at baseline) showed a mean change in the GUSS of 11.8 (95% CI 3.6 to 20.0), higher in denosumab compared with placebo (*P* = 0.004), at week 24 and a change of 19.7 (95% CI 9.4 to 29.9) in favor of denosumab treatment (*P* < 0.001) at week 48.

## Discussion

In this 48-week, placebo-controlled, double-blind study, denosumab 60 mg every 3 months reduced radiographic erosive progression in erosive hand OA versus placebo without increased toxicity. We found a significant effect on GUSS already being present at week 24 and further increasing through week 48. Furthermore, markedly less new erosive joints developed through week 48 in the denosumab group. As anticipated, clinical outcome measures did not significantly change between groups in the initial 48 weeks of treatment. However, we noted significant improvement in pain and disability levels in the extension phase through week 96, suggesting that prolonged treatment with denosumab not only inhibits structural progression but also culminates in clinical improvement over time. The safety profile of denosumab was found comparable to previous studies and use in clinical care^24^, even though the double dose regimen used compared with osteoporosis treatment. This is the first study that demonstrates consistent benefits on radiographic progression in erosive hand OA already after 24 weeks and subsequent clinical benefits after long-term treatment, although these results were based on post-hoc analyses. Previous studies in erosive hand OA with biological agents, such as inhibitors of tumor necrosis factor and interleukin-1, herein failed^25–28^. Treatment with intra-articular corticosteroids did provide pain relief as well as reduction of swelling and sonographic synovitis in a retrospective study^29^. Studies with intra-muscular injections of clodronate, a first-generation bisphosphonate, which showed to inhibit bone resorption, also showed pain relief and improvement of serum cartilage biomarkers^30,31^. The HOPE study, a randomized controlled trial with oral prednisolone versus placebo during 8 weeks (performed in patients with nonerosive hand OA) also demonstrated clear improvement of pain and synovial thickening^32^. However, structural changes were not assessed in these short-term studies, and radiographic changes are unlikely to occur in this short timeframe. In our study, clinical benefits did not occur in the first year, and relatively stable levels of pain remain throughout the entire year. However, this is not surprising, since we previously showed that patients with erosive hand OA suffer from considerable levels of chronic pain, and with every damaged joint, background levels of pain and disability increase^8^. Since our patient population is already suffering from the disease for more than 6 years on average and some quite damaged joints were already present at baseline, it does not surprise that a certain level of chronic pain remains. Prednisolone probably suppresses pain induced by acute, inflammatory attacks, but chronic pain due to underlying damage is more difficult to relieve. Patients in this study were allowed to continue taking pain killers or/and nonsteroidal anti-inflammatory drugs, since no immediate analgesic effects were expected. This might have influenced the clinical outcome, and may even have underestimated the clinical effect of denosumab. The current findings might create a shift toward treatment of erosive hand OA from targeting solely pain relief toward prevention of structural or erosive damage with a cumulative impact on pain and function over time. The ultimate goal of treatment of erosive hand OA, similar to any other type of OA, is to avoid further radiographic damage and substantially reduce the burden of the disease.

Recent findings from the Osteoarthritis Initiative suggest that cortical fragility is present in patients with erosive hand OA, and this might be driving the subchondral bone attrition and development of erosions^15^. The authors suggest that the development of erosions in erosive hand OA can be considered comparable to an osteoporotic fracture^33,34^ and introduce the concept of an ‘osteoporotic’ endotype of OA. The strong inhibitory effect of denosumab on bone resorption, and the development of new erosions in our study are perfectly in line with this concept.

In this current study, two scoring methods for structural radiographic progression were used, both showing significant impact of denosumab. The choice of a radiographic endpoint as primary endpoint was intentionally chosen, since evidence from rheumatoid arthritis clinical trial research with denosumab failed to show clinical benefits but clearly reduced structural damage^23^. Therefore, we hypothesize that in erosive hand OA, similar radiographic antibone resorptive effects would appear without direct clinical benefits. Moreover, as already mentioned earlier, a certain background level of pain and disability is already present in these patients due to underlying damage. This study was not intended to treat acute inflammatory flares of the disease. Obviously, symptom relief is important from the patients’ perspective, and a delayed pain inhibitory effect after 2 years of treatment is a limitation. In hand OA, where several joints can be affected while others remain undamaged, global questionnaires assessing pain and functional impairment in both hands may lack detail, and therefore, a more joint-based assessment of pain and functional impairment instead might be preferred. This may foster a better comprehension of the relationship between structural damage, pain and function in this disease.

Unfortunately, a surrogate outcome measure for disease activity in erosive hand OA is still lacking^35^. Development of such a tool could facilitate the clinical trial research in hand OA. Disease activity and structural progression are undeniably coupled but may be disconnected in time. Our data advocate a sustained need for RANKL inhibition to preserve hand function and onset of new erosive disease. The reduced estimated OR for erosive progression of 77% in the denosumab group compared with placebo supports the concept of osteoclast-dependent structural damage in erosive hand OA.

We found no safety signals for treatment with increased interval dosing of denosumab in our nonosteoporotic population. A higher number of nonserious and serious adverse events were reported in the placebo group. As expected, all bone mineral density values increased in the denosumab group and at the spine in the placebo group, which might be attributed to the calcium and vitamin D administration. Of course, several unclarities about safety remain upon chronic use of denosumab in this population: since discontinuation of denosumab in osteoporotic patients induces a rapid increase in bone turnover, this might be the case here as well and merits further attention.

The limitations of this study must be considered. Since hand OA is a heterogeneous disease, patients’ stratification is probably required in clinical trials to select the ones who will benefit from treatment. Inclusion of a specific subset of patients is, however, both a strength and limitation: while it increases the likelihood to observe an effect of the targeted treatment, it limits the generalizability of the results to patients with hand OA without inflammatory signs. Another limitation of the study is its monocentric design: a larger, multicenter study is warranted to confirm the results.

Future research should target the long-term effects of denosumab in this population, not only how long the erosive inhibitory features last after treatment cessation but also the effect on bone quality in this nonosteoporotic population. Furthermore, the role of RANKL in the pathogenesis of this disease, particularly the cellular source, merits further attention. Finally, the results of this proof-of-concept monocentric study needs to be confirmed in a larger, ideally multicenter phase 3 study.

In summary, this placebo-controlled trial provides the first proof of concept that structural damage in erosive hand OA can be modulated by a targeted therapy. Reduction of radiographic progression and prevention of new erosive joints were observed with denosumab 60 mg every 3 months. Subsequently, this led to improvement in pain and disability after long-term treatment through 96 weeks. This study introduces new promising treatment possibilities for patients suffering from a disease, such as erosive hand OA, with high unmet needs.

## Methods

### Study design

This monocentric, randomized, placebo-controlled, double-blind, parallel-group, phase 2a study in patients with erosive hand OA (EU Clinical Trials Registry, identifier 2015-003223-53) was carried out at the rheumatology outpatient clinic of the Ghent University Hospital in Belgium. The trial protocol was approved by the local ethics committee of the hospital and was conducted in accordance with Good Clinical Practice guidelines and the Declaration of Helsinki. See Supplementary Note 1 for a list of members of the ethical committee. The study protocol is shown in the Appendix. We used the CONSORT checklist when writing our report^38^. See Supplementary Note 1 for a list of data monitoring committee members.

### Patients

Patients aged ≥30 years and diagnosed with erosive hand OA were considered eligible. Patients were recruited from the rheumatology outpatient clinic of the Ghent University Hospital in Belgium. Erosive hand OA was defined as radiographic presence of ≥1 IP joint in the J or E phase according to the Verbruggen and Veys anatomical phase scoring system^13^. Key inclusion criteria included the presence of ≥1 IP joint with partial or complete loss of joint space (that is, ‘J’ phase of the anatomical phase scoring system) or with central erosions (that is, ‘E’ phase according to the anatomical phase scoring system) and with local inflammatory signs, defined both clinically (that is, presence of soft tissue swelling) and by ultrasound (that is, presence of effusion and/or synovial hyperproliferation at least grade 1 on ultrasound); suffering from transient inflammatory attacks of the IP joints, as referred to as inflammatory or erosive hand OA; an age over 30 years; and providing written informed consent and willing to comply to all requirements according to the protocol. Key exclusion criteria included previous denosumab use; intake of oral bisphosphonates during the past 12 months; oral strontium ranelate or intravenous bisphosphonates during the past 5 years; recent use of chondroprotective molecules or disease modifying drugs as summarized in the protocol during the past 90 days; vitamin D deficiency; current hypo- or hypercalcemia; important comorbidities, cancers or chronic infectious diseases; underlying conditions that compromise the ability to provide written informed consent or to comply to all requirements; history of osteonecrosis of the jaw, recent tooth extraction (within past 3 months) or other unhealed dental procedure; planned invasive dental procedures during the study; history of solid organ or bone marrow transplantation; known hypersensitivity to the study medication or its components; history of alcohol or drug abuse during the past year; breastfeeding; and pregnancy or wishing to be pregnant. Patients suffering from chronic inflammatory rheumatic diseases such rheumatoid arthritis, spondyloarthropathy, psoriatic arthritis, gout, chondrocalcinosis or other auto-immune disease (for example, systematic lupus erythematosus) were excluded. Serology screening was performed if appropriate. The sex of participants was determined on the basis of self-report (male or female). All patients provided written informed consent.

### Randomization and masking

Eligible patients were randomly assigned, in a 1:1 ratio, to receive in a blinded fashion denosumab (Amgen) or placebo during the placebo-controlled double-blind phase of the study, by use of a randomization scheme with a fixed block size of four. The randomization list was generated by a coworker independent of the study and not involved in any procedure during the study. The study medication was provided by the pharmacy department. The medication and placebo syringes were identical in terms of color and shape and labeled with an unique sample number and study patient identification number. Patients and investigators retained unaware of the initial allocation during the entire trial, including the open-label extension.

### Procedures

Denosumab 60 mg or placebo was administered subcutaneously by a dedicated (blinded) nurse or physician at site every 12 weeks for 48 weeks, followed by open-label denosumab 60 mg every 12 weeks for an additional 48 weeks (Extended Data Fig. 1). Since earlier studies in rheumatoid arthritis revealed dose-dependent structural inhibitory capacities of denosumab^22,23^, we anticipated to use a higher dosing interval than as approved for use in osteoporosis. The medication and placebo syringes were identical. All patients received daily oral calcium (1,000 mg elemental calcium) and vitamin D_3_ (880 IU). Medication intake, use of rescue medication or changes in concomitant medication was registered throughout the entire study. The patients were allowed to take analgesics and nonsteroidal anti-inflammatory drugs as rescue medication at stable dosages during the first 12 weeks. The intake of corticosteroids was prohibited.

A posteroanterior hand radiograph of both hands were taken at baseline, weeks 24 and 48. All 16 IP joints (the second to fifth distal and proximal IP joints) were evaluated by two experienced rheumatologists (G.V. and R.W.). The first IP was excluded due to reduced visibility on radiographs. Two radiographic scoring systems were used to assess the structural changes of the finger joints^13,14^. Both readers independently scored paired images with the known time sequence but were blinded for randomization, patient identity and clinical information. The GUSS^14^ includes three subdomains to assess changes in the subchondral bone, subchondral plate and joint space loss. Details of the GUSS are extensively described in the protocol, and an educational atlas is available^14^. In summary, each subdomain ranges from 0 to 100 and the total GUSS score is the composite score of the three subscales with equal weight. The total GUSS score ranges from 0 to 300, with the lowest scores representing severe erosive joint destruction and the highest scores representing no damage or complete subchondral and cartilage repair. For each (target) joint, a total GUSS score is computed. The second radiographic scoring system, the anatomical phase scoring system by Verbruggen and Veys, is based on the natural history of joints throughout the erosive OA process^13^. The Verbruggen and Veys anatomical score system differentiates normal joints (N) from pre-erosive phases (S phase, that is, stationary phase with minimal degenerative features such as subchondral sclerosis, joint space narrowing and presence of small osteophytes, and J phase, with partial or complete loss of joint space), erosive phase (E) and phases of remodeling (R, that is, signs of repair such as reappearance of subchondral plate and joint space width, disappearance of erosions at the subchondral bone and development of osteophytes at joint margins, and F, fused joint as extreme sign of remodeling). Inter- and intrareader reliability analysis was performed. The final radiographic scores were the agreement scores amongst the two readers. In case of no absolute agreement, a consensus score was made.

Ultrasound was performed by an experienced sonographer (R.W.), with more than 10 years of experience, at baseline and weeks 12 and 48. Synovial proliferation (0–3), effusion (0–3), power Doppler signal (0–3) and erosions (present/absent) in proximal IP and distal IP joints 2–5 were recorded.

Pain was questioned (“How would you rate the pain in the finger joints of both hands during the past 24 hours?”) and rated on a NRS from 0 to 10, with 0 corresponding to no pain and 10 maximal pain, at every visit, together with questionnaires of functional outcome, the FIHOA (0–30)^37^ and the AUSCAN (0–150)^38^. At each visit after baseline, patients were asked how effective they found the administered treatment (on a NRS from 0 to 10, with 0 corresponding to no effect and 10 to the best effect). Dual energy X-ray absorptiometry was performed at baseline and week 48. An overview of assessments is shown in Extended Data Fig. 1. Deindentified raw data collected through week 48 are available as Supplementary Information.

### Outcomes

The primary efficacy endpoint was the change in total GUSS^14^ from baseline to week 24. The scoring system can change in positive (that is, more remodeling) or negative direction (that is, more erosive progression). Target joints were defined as all proximal and/or distal IP joints in the J or E phase on baseline radiographs (except IP 1) with presence of inflammatory activity, defined by both clinical soft tissue swelling and ultrasonographic inflammation (that is, either synovial proliferation or effusion). If several target joints were available, all were included for efficacy analysis.

The secondary endpoints were the total GUSS changes from baseline to week 48 and the percentage of new erosive joints (J/E) by Verbruggen and Veys^13^ among the baseline pre-erosive joints (that is, baseline N, S and J joints) per patient at week 48.

Exploratory clinical endpoints and patient-reported outcomes were NRS pain, NRS global assessment of efficacy by patient, tender joint count, swollen joint count and AUSCAN and FIHOA at weeks 24 and 48 (refs. ^37,38^). In analogy with rheumatoid arthritis, where denosumab showed to reduce structural damage while having no effect on signs and symptoms^23^, it was anticipated that in erosive hand OA, no clinical effect could be expected in the first year of treatment, and therefore, no pain scales or patient-related outcome measures were considered as primary endpoints. Changes in ultrasound scores at week 12 for effusion, synovial proliferation, synovitis score and power Doppler signal, and for erosions at week 48, and percentage changes from baseline in bone mineral density at the femoral neck and lumbar spine at week 48 were other exploratory outcomes.

Safety endpoints included the number of (serious) adverse events, withdrawal because of adverse events and changes in laboratory data throughout the study.

### Statistical analysis

A sample size of 46 patients in each treatment arm was required to detect a difference in the mean change GUSS of 20 units between the placebo and treated group at week 24, attaining a power of 90%, assuming that the standard deviation was 29 using a *t*-test with a two-sided 0.05 level of significance (α). Taking into account an attrition rate of 8%, 100 patients were included.

Primary efficacy analyses were performed in an ITT approach (that is, all participants randomly assigned to groups and who attended a baseline visit). Changes in the GUSS were analyzed at joint level with generalized estimating equations (GEE), accounting for within-patient clustering and adjusted for baseline unbalances. Robust standard errors were used, and the working correlation structure specified exchangeable. The independent variables included in the model were the treatment group, visit number (categorical), interaction between treatment group and visit number and baseline value of the dependent variable (continuous). Missing values were imputed according to a predefined imputation model, including the randomization group, baseline value and values at other time points available, presence of baseline inflammation and baseline number of affected joints. As there was only one primary outcome, no adjustments for multiple testing were performed.

Secondary and exploratory outcomes were done in the ITT population and measured at patient level (except for total GUSS at week 48). Primary and secondary efficacy analyses were presented by least squares means and standard error of the mean, a point estimate of the difference between the treatment groups or ORs with a 95% CI and the two-sided *P* value. Missing values were replaced by the baseline observations.

A first sensitivity analysis of the primary endpoint was performed by using a more conservative approach to handle missing values by replacing these with the baseline observations. A second sensitivity analysis, three-level linear mixed model with patient and joint as random effects and total GUSS baseline, treatment, time (week 24 and week 48) and the interaction between time and treatment as fixed effects was performed.

For the primary outcome measure, a subgroup analysis for inflammatory activity in the joint (yes/no) was done. The interaction between the presence of baseline inflammation and treatment effect on change in the GUSS scores over 24 weeks was tested. For the primary outcome measure, another efficacy analysis was performed, extending the target joints to all joints showing any progression to the J, E or E/R phase throughout the study that were not defined as J or E at baseline.

For the analyses from the open-label extension phase, similar GEE logistic regression models were used with treatment groups based on the initial randomization code from the placebo-controlled phase (that is, patients having received placebo versus denosumab).

A *P* value below 0.05 was considered statistically significant. All statistical calculations were performed using R version 3.6.1 and IBM SPSS Statistics for Windows, version 25. The statistical analysis plan, which was written before breaking the randomization code, is available in the Appendix.

### Reporting summary

Further information on research design is available in the Nature Portfolio Reporting Summary linked to this article.

## Online content

Any methods, additional references, Nature Portfolio reporting summaries, source data, extended data, supplementary information, acknowledgements, peer review information; details of author contributions and competing interests; and statements of data and code availability are available at 10.1038/s41591-024-02822-0.

## Supplementary information

Supplementary Information List of members of ethics committee and data monitoring board; protocol; statistical analysis plan.

Reporting Summary

## Source data

Source Data Source data of primary and secondary endpoints.

## Figures and Tables

**Extended Data Table 1:**
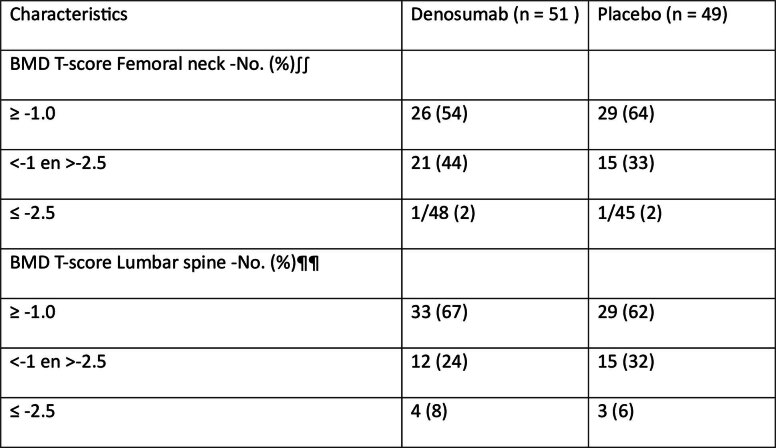
Baseline bone mineral density values

BMD: bone mineral density. ∫∫ Bone mineral density at femoral neck: 3 values missing in the denosumab group, 4 missing in the placebo group. ¶¶ Bone mineral density at the spine: 2 values missing in the denosumab group, 2 values missing in the placebo group.

**Extended Data Table 2:**
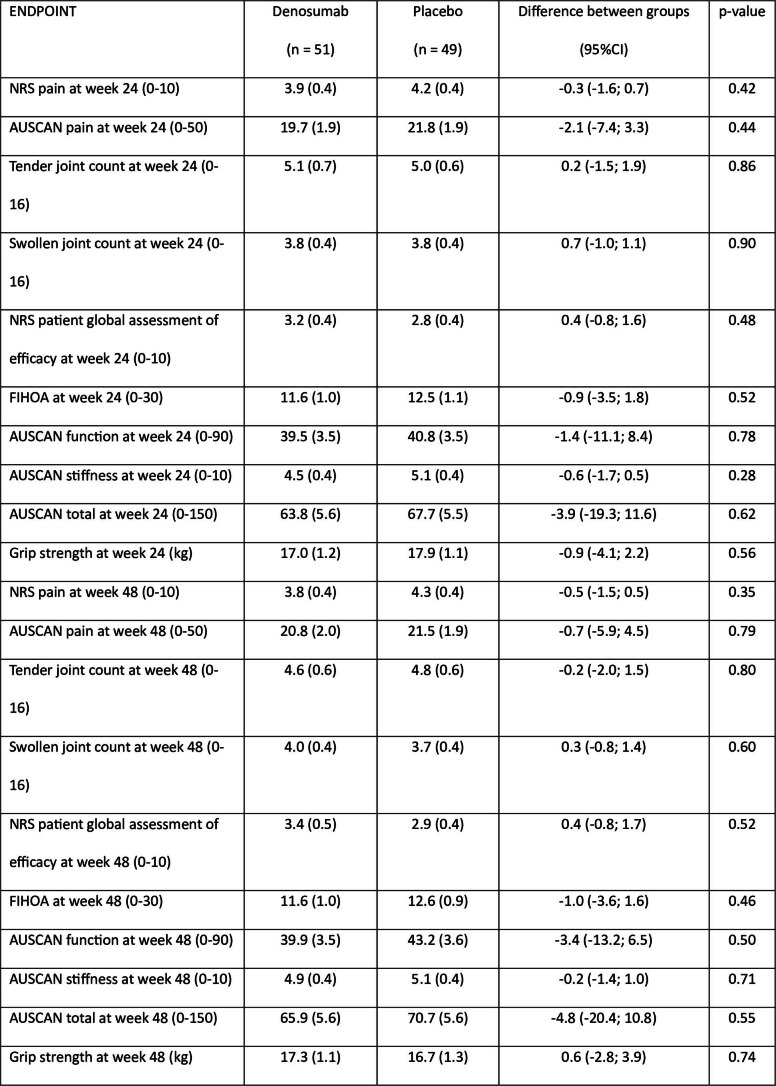
Results of the exploratory clinical endpoint analyses

Values are least squares mean ± standard error of the mean, unless otherwise stated. Comparisons between groups was done by Generalized Estimation Equations at patient level in the intention-to-treat population (n = 100). Missing data were imputed by baseline observations for the secondary and exploratory endpoints. No correction for multiple comparison done since only one primary endpoint. CI: confidence interval; OR: odds ratio; NRS: numeric rating scale; AUSCAN: Australian Canadian Hand Osteoarthritis Index; FIHOA: functional index for hand osteoarthritis.

**Extended Data Table 3:**
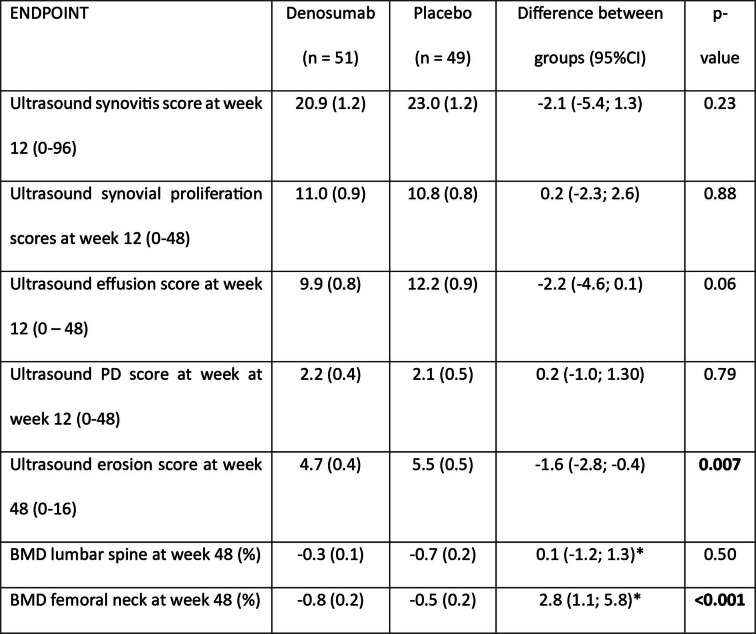
Results from exploratory imaging endpoint analyses

Values are least squares mean ± standard error of the mean, unless otherwise stated. Comparisons between groups was done by Generalized Estimation Equations at patient level in the intention-to-treat population (n = 100). Missing data were imputed by baseline observations for the secondary and exploratory endpoints. No correction for multiple comparison done since only one primary endpoint. P-values in bold represent statistical significance (<0.05). CI: confidence interval; OR: odds ratio; PD: Power Doppler; BMD: bone mineral density. *: percentage change at week 48 from baseline.

**Extended Data Table 4:**
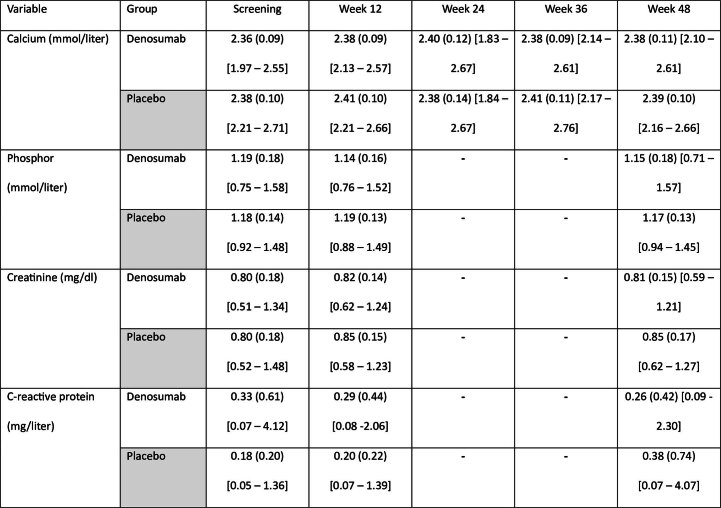
Longitudinal laboratory values

Data shown are mean (standard deviation)[range]; number of patients included for analysis in the denosumab group at screening (n = 51), week 12 (n = 46), week 24 (n = 47), week 36 (n = 45), and week 48 (n = 45); number of patients included for analysis in the placebo group at screening (n = 49), week 12 (n = 47), week 24 (n = 46),week 36 (n = 45), and week 48 (n = 45).

**Extended Data Table 5:**
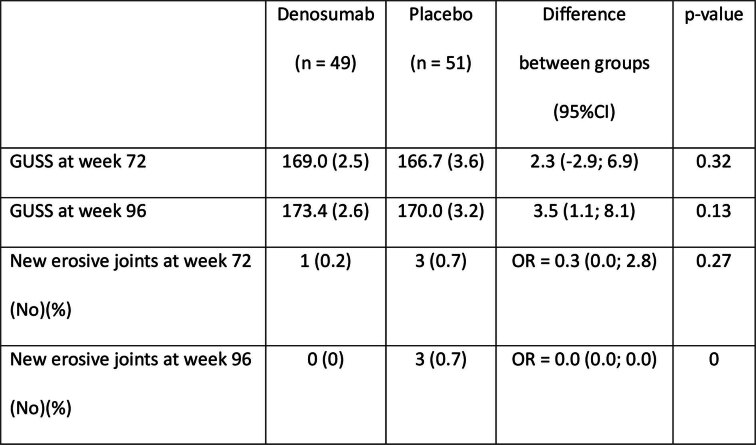
Radiographic data from the open-label extension phase

Values are least squares means ± standard error of the mean, unless otherwise stated. Comparisons between groups was done by Generalized Estimation Equations at joint level in the intention-to-treat population. Missing data were imputed according to a predefined imputation model for the primary endpoint. No correction for multiple comparison done since only one primary endpoint. Week 72 and 96 data originate from post-hoc analyses of the open label extension phase and are changes compared to week 48. CI: confidence interval; No.: number; GUSS: Ghent University scoring system; OR: odds ratio.

**Extended Data Table 6:**
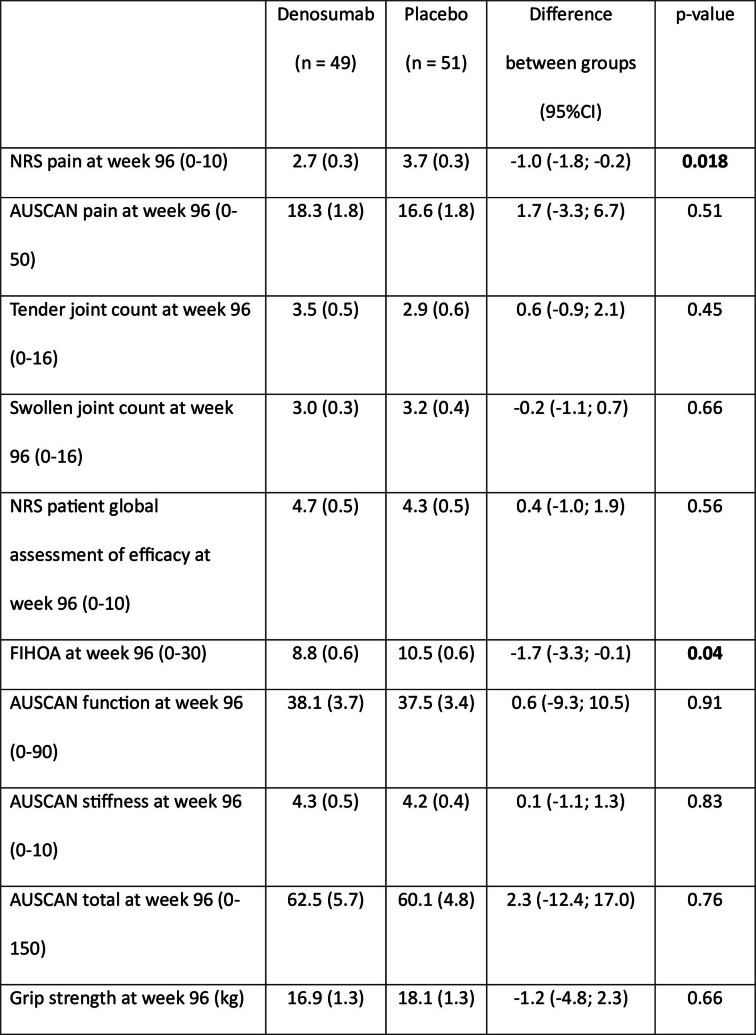
Clinical data from the open-label extension phase

Values are least squares mean ± standard error of the mean, unless otherwise stated. Comparisons between groups was done by Generalized Estimation Equations at patient level in the intention-to-treat population (n = 100). Missing data were imputed by baseline observations for the exploratory endpoints. Week 72 and 96 data originate from post-hoc analyses of the open label extension phase. No correction for multiple comparison done since only one primary endpoint. P-values in bold represent statistical significance (<0.05). CI: confidence interval; OR: odds ratio; NRS: numeric rating scale; AUSCAN: Australian Canadian Hand Osteoarthritis Index; FIHOA: functional index for hand osteoarthritis.

## Data Availability

Deidentified raw data available as supplementary information. To the extent that current legislation allows it, the authors will provide access to additional individual deidentified participant-level data that underlie the data presented in this article to researchers who provide a methodologically sound proposal for academic purposes to interpret, verify and extend research in the article that does not violate intellectual property or confidentiality obligations, beginning 12 months after article publication. Researchers should contact the corresponding author when applying for additional data access. Use of data will be restricted to the agreed purpose. Requests will be answered within 4 weeks. The study protocol with amendments and statistical analysis plan are available in the appendix. Source data are provided with this paper.

## Code availability

The statistical analyses code can be found at https://github.com/tinevh/NMED-A126967B.

## References

[1] Favero, M. et al. Erosive hand osteoarthritis: latest findings and outlook. *Nat. Rev. Rheumatol.* **18**, 171–183 (2022). 35105980 10.1038/s41584-021-00747-3

[2] Kwok, W. Y. et al. Erosive hand osteoarthritis: its prevalence and clinical impact in the general population and symptomatic hand osteoarthritis. *Ann. Rheum. Dis.* **70**, 1238–1242 (2011). 21474485 10.1136/ard.2010.143016

[3] Mannoni, A. et al. Epidemiological profile of symptomatic osteoarthritis in older adults: a population based study in Dicomano, Italy. *Ann. Rheum. Dis.* **62**, 576–578 (2003). 12759299 10.1136/ard.62.6.576 PMC1754567

[4] Haugen, I. K. et al. Prevalence, incidence and progression of hand osteoarthritis in the general population: the Framingham Osteoarthritis Study. *Ann. Rheum. Dis.* **70**, 1581–1586 (2011). 21622766 10.1136/ard.2011.150078 PMC3867970

[5] Cecil, R. L. & Archer, B. H. Arthritis of the menopause: a study of fifty cases. *JAMA* **84**, 75–79 (1925).

[6] Haugen, I. K. et al. Synovitis and radiographic progression in non-erosive and erosive hand osteoarthritis: is erosive hand osteoarthritis a separate inflammatory phenotype? *Osteoarthr. Cartil.* **24**, 647–654 (2016). 10.1016/j.joca.2015.11.014 26620088

[7] Bijsterbosch, J. et al. Clinical burden of erosive hand osteoarthritis and its relationship to nodes. *Ann. Rheum. Dis.* **69**, 1784–1788 (2010). 20410068 10.1136/ard.2009.125435

[8] Wittoek, R., Cruyssen, B. V. & Verbruggen, G. Predictors of functional impairment and pain in erosive osteoarthritis of the interphalangeal joints: comparison with controlled inflammatory arthritis. *Arthritis Rheum.* **64**, 1430–1436 (2012). 22139828 10.1002/art.33502

[9] Kloppenburg, M. et al. 2018 update of the EULAR recommendations for the management of hand osteoarthritis. *Ann. Rheum. Dis.* **78**, 16–24 (2019). 30154087 10.1136/annrheumdis-2018-213826

[10] Haugen, I. K., Slatkowsky-Christensen, B., Boyesen, P., van der Heijde, D. & Kvien, T. K. Cross-sectional and longitudinal associations between radiographic features and measures of pain and physical function in hand osteoarthritis. *Osteoarthr. Cartil.* **21**, 1191–1198 (2013). 10.1016/j.joca.2013.04.004 23973130

[11] Meersseman, P., Van de Vyver, C., Verbruggen, G., Elewaut, D. & Wittoek, R. Clinical and radiological factors associated with erosive radiographic progression in hand osteoarthritis. *Osteoarthr. Cartil.* **23**, 2129–2133 (2015). 10.1016/j.joca.2015.06.008 26093212

[12] Vanhaverbeke, T., Pardaens, L. & Wittoek, R. Natural disease progression in finger osteoarthritis: results from a 10 year follow-up cohort. *Scand. J. Rheumatol.* **49**, 498–504 (2020). 32727238 10.1080/03009742.2020.1771762

[13] Verbruggen, G. & Veys, E. M. Numerical scoring systems for the anatomic evolution of osteoarthritis of the finger joints. *Arthritis Rheum.* **39**, 308–320 (1996). 8849385 10.1002/art.1780390221

[14] Verbruggen, G., Wittoek, R., Vander Cruyssen, B. & Elewaut, D. Morbid anatomy of ‘erosive osteoarthritis’ of the interphalangeal finger joints: an optimised scoring system to monitor disease progression in affected joints. *Ann. Rheum. Dis.* **69**, 862–867 (2010). 19948521 10.1136/ard.2009.112714 PMC2925149

[15] McAlindon, T. E. et al. Erosive hand osteoarthritis: incidence and predictive characteristics among participants in the Osteoarthritis Initiative. *Arthritis Rheumatol.* **73**, 2015–2024 (2021). 33844453 10.1002/art.41757 PMC8505573

[16] Sowers, M., Zobel, D., Hawthorne, V. M., Carman, W. & Weissfeld, L. Progression of osteoarthritis of the hand and metacarpal bone loss. A twenty-year followup of incident cases. *Arthritis Rheum.* **34**, 36–42 (1991). 1984778 10.1002/art.1780340106

[17] Yusuf, E. et al. Association between weight or body mass index and hand osteoarthritis: a systematic review. *Ann. Rheum. Dis.* **69**, 761 (2010). 19487215 10.1136/ard.2008.106930

[18] Favero, M., Perino, G., Valente, M. L., Tiengo, C. & Ramonda, R. Radiological and histological analysis of two replaced interphalangeal joints with active subchondral bone resorption in erosive hand osteoarthritis: a novel mechanism? *Skelet. Radiol.* **46**, 385–391 (2017). 10.1007/s00256-016-2560-y 28054155

[19] Rovetta, G., Monteforte, P., Grignolo, M. C., Brignone, A. & Buffrini, L. Hematic levels of type I collagen C-telopeptide in erosive versus nonerosive osteoarthritis of the hands. *Int. J. Tissue React.* **25**, 25–28 (2003). 12854884

[20] McClung, M. R. et al. Denosumab in postmenopausal women with low bone mineral density. *N. Engl. J. Med.* **354**, 821–831 (2006). 16495394 10.1056/NEJMoa044459

[21] So, H. et al. Effects of RANKL inhibition on promoting healing of bone erosion in rheumatoid arthritis using HR-pQCT: a 2-year, randomised, double-blind, placebo-controlled trial. *Ann. Rheum. Dis.* **80**, 981–988 (2021). 33811034 10.1136/annrheumdis-2021-219846

[22] Cohen, S. B. et al.Denosumab treatment effects on structural damage, bone mineral density, and bone turnover in rheumatoid arthritis: a twelve-month, multicenter, randomized, double-blind, placebo-controlled, phase II clinical trial. *Arthritis Rheum.* **58**, 1299–1309 (2008). 18438830 10.1002/art.23417

[23] Ishiguro, N. et al. Consistent inhibition of bone destruction by denosumab in important subgroups of Japanese patients with rheumatoid. *Arthritis Rheumatol.* **66**, S831 (2014).

[24] Papapoulos, S. et al. The effect of 8 or 5 years of denosumab treatment in postmenopausal women with osteoporosis: results from the FREEDOM Extension study. *Osteoporos. Int.* **26**, 2773–2783 (2015). 26202488 10.1007/s00198-015-3234-7 PMC4656716

[25] Verbruggen, G., Wittoek, R., Vander Cruyssen, B. & Elewaut, D. Tumour necrosis factor blockade for the treatment of erosive osteoarthritis of the interphalangeal finger joints: a double blind, randomised trial on structure modification. *Ann. Rheum. Dis.* **71**, 891–898 (2012). 22128078 10.1136/ard.2011.149849 PMC3371224

[26] Chevalier, X. et al. Adalimumab in patients with hand osteoarthritis refractory to analgesics and NSAIDs: a randomised, multicentre, double-blind, placebo-controlled trial. *Ann. Rheum. Dis.* **74**, 1697–1705 (2015). 24817417 10.1136/annrheumdis-2014-205348

[27] Kloppenburg, M. et al. Etanercept in patients with inflammatory hand osteoarthritis (EHOA): a multicentre, randomised, double-blind, placebo-controlled trial. *Ann. Rheum. Dis.* **77**, 1757–1764 (2018). 30282670 10.1136/annrheumdis-2018-213202

[28] Kloppenburg, M. et al. Phase IIa, placebo-controlled, randomised study of lutikizumab, an anti-interleukin-1α and anti-interleukin-1β dual variable domain immunoglobulin, in patients with erosive hand osteoarthritis. *Ann. Rheum. Dis.* **78**, 413–420 (2019). 30552176 10.1136/annrheumdis-2018-213336 PMC6390132

[29] Favero, M. et al. Efficacy and safety of ultrasound-guided intra-articular glucocorticoid injection in erosive hand osteoarthritis. *Pain Med.* **22**, 1229–1232 (2021). 32914191 10.1093/pm/pnaa261

[30] Saviola, G. et al. Clodronate and hydroxychloroquine in erosive osteoarthritis: a 24-month open randomized pilot study. *Mod. Rheumatol.* **22**, 256–263 (2012). 21853386 10.1007/s10165-011-0506-8

[31] Saviola, G. et al. Intramuscular clodronate in erosive osteoarthritis of the hand is effective on pain and reduces serum COMP: a randomized pilot trial—the ER.O.D.E. study (ERosive Osteoarthritis and Disodium-clodronate Evaluation). *Clin. Rheumatol.* **36**, 2343–2350 (2017) ; erratum (2018) in *Clin. Rheumatol.* **37**, 2019 (2018). 10.1007/s10067-017-3681-y 28536825

[32] Kroon, F. P. B. et al. Results of a 6-week treatment with 10 mg prednisolone in patients with hand osteoarthritis (HOPE): a double-blind, randomised, placebo-controlled trial. *Lancet* **394**, 1993–2001 (2019). 31727410 10.1016/S0140-6736(19)32489-4

[33] Buckland-Wright, J. C., Lynch, J. A., Rymer, J. & Fogelman, I. Fractal signature analysis of macroradiographs measures trabecular organization in lumbar vertebrae of postmenopausal women. *Calcif. Tissue Int.* **54**, 106–112 (1994). 8012865 10.1007/BF00296060

[34] Weinstein, R. S. & Majumdar, S. Fractal geometry and vertebral compression fractures. *J. Bone Miner. Res.* **9**, 1797–1802 (1994). 7863830 10.1002/jbmr.5650091117

[35] Wittoek, R. et al. Report from the Hand Osteoarthritis Working Group at OMERACT 2018: update on core instrument set development. *J. Rheumatol.* **46**, 1183–1187 (2019). 30647176 10.3899/jrheum.181003

[36] Dreiser, R. L., Maheu, E., Guillou, G. B., Caspard, H. & Grouin, J. M. Validation of an algofunctional index for osteoarthritis of the hand. *Rev. Rhum. Engl. Ed.* **62**, 43S–53S (1995). 7583182

[37] Bellamy, N. et al. Dimensionality and clinical importance of pain and disability in hand osteoarthritis: development of the Australian/Canadian (AUSCAN) Osteoarthritis Hand Index. *Osteoarthr. Cartil.* **10**, 855–862 (2002). 10.1053/joca.2002.0837 12435330

[38] Schulz, K. F. et al. CONSORT 2010 Statement: updated guidelines for reporting parallel group randomised trials. *BMJ.* **23**, 340:c332 (2010). 10.1136/bmj.c332 PMC2844940 20332509

