# Supplementary material for: RANKL blockade for erosive hand osteoarthritis: a randomized placebo-controlled phase 2a trial
Source: Nat Med. 2024 Feb 15;30(3):829–36. doi: 10.1038/s41591-024-02822-0 (PMC10957468; doi:10.1038/s41591-024-02822-0)
Supplement: Supplementary file 1 — List of members of ethics committee and data monitoring board; protocol; statistical analysis plan. [file 41591_2024_2822_MOESM1_ESM.pdf]

nature medicine

Article

https://doi.org/10.1038/s41591-024-02822-0

RANKL blockade for erosive hand osteoarthritis: a randomized placebo-controlled phase 2a trial

In the format provided by the authors and unedited

Supplementary note 1

List of Members of the Ethics committee of the Ghent University Hospital that approved the study: D. MATTHYS (president), J. DECRUYENAERE (secretary), T. BALTHAZAR, W. CEELEN, K. DHONDT, C. DEMEESTERE, P. DERON, M. DE MUYNCK, G. DE SMET, S. JANSSENS, K. KINT, W. NOTEBAERT, M. PETROVIC, R. PIERS, R. RUBENS, P. SCHELSTRAETE, S. STERCKX, B. VANDERHAEGEN, Prof.dr. K. VANDEWOUDE, S. VERCOUTERE, F. VANDEKERCKHOVE, J. VAN ELSEN, K. VAN LIERDE

Data Monitoring Committee members: H. DE PLA, H. KOKUR, D. LONCKE.

Study Protocol

RANKL-blockade for the treatment of erosive osteoarthritis (OA) of interphalangeal finger joints

Randomized, double blind, placebo-controlled study to evaluate the efficacy of denosumab 60mg sc every 3 months in patients with erosive osteoarthritis of the interphalangeal finger joints

Principal Investigators:
[redacted]

Dept. of Rheumatology – Ghent University Hospital

EudraCT number: 2015-003223-53
Protocolnumber: AGO/2015/008

[redacted]

[redacted]

[redacted]

Protocol Signature Page

Principal/Chief Investigator signature

I confirm that I have read and understood protocol version ~~xx dated xx January 2015.~~ 1.0 - 12 AUGUST 2015 [redacted] 25/4/17 I agree to comply with the study protocol, the principals of GCP, research governance, clinical trial regulations and appropriate reporting requirements.

Signature.. Date 7 MAR 2016

Print name [redacted]

Protocol synopsis

**Study Type**	**I**nvestigator **S**ponsored **S**tudy
**Funder**	Amgen
**Study Design**	This is a randomized, double blind placebo controlled one-site proof-of-concept study in subjects with erosive osteoarthritis (OA) of interphangeal (IP) finger joints.<br>A total of 100 subjects will be enrolled into the study: 48 weeks placebo controlled double-blind phase with denosumab 60 mg every 12 weeks, followed by a 48-week open-label phase in which all subjects will receive denosumab.
**Investigational Therapy**	Denosumab 60 mg subcutaneous injection every 12 weeks. All subjects will receive Calcium/vit D supplementation.
**Efficacy Objectives**	The **primary objective** is to assess the effect of denosumab on the reduction of radiographic erosive progression using GUSS™ (Ghent University Score System).<br>The **secondary objective** is to assess the effect of denosumab on the reduction of radiographic erosive progression as defined by diminishing the appearance of new erosive IP finger joints.<br>The **exploratory objective** is mainly to assess the effect of denosumab on clinical variables, as well as ultrasonography and DEXA parameters.
**Main Endpoints**	**Primary Endpoint:** The change in the negative evolution of GUSS™ scores in the target IP joints from baseline to week 24.<br>**Other Endpoints:** 1) The change in the negative evolution of GUSS™ scores in the target IP joints from week 24 to week 48 and from baseline to week 48. 2) The number of patients that develop new erosive IP joints ('S/J' to 'E' phase joints) at 48 weeks; 3) The number of 'S/J' IP joints that develop 'E' phases at 48 weeks.
**Hypothesis**	**The main hypothesis** is that the repeated administration of denosumab 60 mg Q3 months can lead to reduce structural damage in erosive hand OA.
**Study Sites**	1 site – the Ghent site
**Subjects**	100 subjects
**Enrolment**	18 months
**Main Eligibility Criteria**	Males and females ≥ 30 years of age, with hand erosive OA:<br>1) having suffered from transient inflammatory attacks of the IP finger joints<br>2) showing at the time of enrolment inflammatory signs and at least one IP finger joint with the typical X-rays appearance of a 'J' or 'E' phase joint

**Study treatment Duration**	96 weeks

Table of Contents

- Protocol Title and Investigators
- Protocol signature page
- Protocol synopsis

1. Background and Rationale
1.1 Disease background
1.2 Denosumab
1.3 Rationale for study design
1.4 Hypotheses
2. Study Objectives and Endpoints
3. Experimental Plan
3.1 Study design and schematic
3.2 Number of sites
3.3 Number of subjects
3.4 Estimated study duration
4. Subject Eligibility
4.1 Inclusion criteria
4.2 Exclusion criteria
5. Treatment and Study Procedures
5.1 Investigational product
5.2 Reporting requirements for investigational product complaints
5.3 Concomitant therapy
5.4 Study procedures and schedule of assessments
6. Statistical and Analytical Plans
6.1 Efficacy analysis
6.2 Safety analysis
6.3 Determination of sample size
7. Adverse Events/Adverse Event reporting
7.1 Definitions
7.1.1. Adverse Event
7.1.2. Serious Adverse Event
7.1.3. Adverse Event Severity
7.1.4. Relationship to Study Drug
7.2. Adverse Event Reporting
8. Regulatory obligations
8.1Informed Consent
8.2 Independent Ethics Committee/Institutional Review Board
9. Documentation relating to the clinical trial- trial master file
10. Publication Policy
10. References
11. Appendices
Appendix 1. Scoring systems
Appendix 2. Overall assessments

1. Background and Rationale

1.1 Disease background

Erosive osteoarthritis (OA) of the interphalangeal (IP) finger joints is considered an inflammatory subset of osteoarthritis of the hand. Its inflammatory clinical presentation and destructive nature are unmistakable. [1,2,3,4,5] The cumulation of destructive changes in the IP joints eventually results in considerable disability.[6,7,8] There are no significant differences in hand function, stiffness and level of pain between patients with hand OA and rheumatoid arthritis. Scores for both patient groups differ significantly from those of healthy controls.[9]
Patients with erosive OA show more functional impairment and significantly more pain compared to patients with controlled inflammatory arthritis affecting the hands. The acquired structural damage of the IP joints due to destructive/reparative phenomena is the largest contributor to functional limitations.[8]
Radiological prevalence of moderate to severe hand OA is estimated to occur in 7.3% (2.65 million) US adults aged 60+ years.[10] Similar data have been reported in European countries.[7,11,12,13,14]

A significant proportion of these patients suffer from the erosive type of hand OA. In a prospective study of 500 consecutive patients attending a rheumatology clinic with symptomatic limb joint OA, 4.8% cases were identified with erosive IP joint OA.[15]

In a survey on the entire health district in the Venetian area, 2.2% out of 640 subjects aged 40+ years had erosive OA of their IP joints.[16] Mainly women in the perimenopauzal age were affected.[17]

Even higher prevalences were seen in a British cohort study[18] on 2.986 people[18]. Numbers in this study were based on clinics and the authors proposed that a proportion of their polyarticular cases were “inflammatory types of OA in association with erosions”. This assumption was based on an earlier study where clinical examination was validated against hand radiography (Egger et al., J Rheumatol 1995;22:1509–13).
Though the proportions of “erosive IP OA” reported here were probably overrated, the prevalence of what is considered to be “erosive IP OA” in this 53 years of age population was twice as high in women (10,6%), compared to men (5,9%).
More recently, these data were confirmed in 2 large population studies where the prevalence of radiographic erosive IP OA in subjects over 55 years of age ranged between 5.0 and 9.9%.[19,20]. The prevalence for men was lower at 3.3%.

These studies showed that erosive type of hand OA occurred predominantly in women.
Haugen IK et al. et al.[20] defined erosive IP OA at a joint level as Kellgren/Lawrence ≥2 plus erosions. The authors reported a prevalence of erosive IP OA in women of 9,9%, 3 times as high as in men (3,3%). In essence, the Kwok W-Y et al. figures[19] agree with the data above.

Moreover, the Haugen IK et al.[20] reported that symptomatic OA was twice as high in women (15,9%), compared to men (8,2%). Symptomatic OA here was defined as Kellgren/Lawrence stage ≥2 plus pain/aching/stiffness.

From these epidemiological studies we can conclude that the incidence of erosive OA of the IP finger joints ranges from five to ten percent particularly in women.

The aggressive destructive nature of the erosive OA is only recognized late in the disease and the radiological image of the "exhausted" final phase mimics a robust OA. Therefore, the disease was hitherto regarded as a form of primary OA - a degenerative joint disease that is caused by biomechanical overload of the joint structures. There is so far no therapy sought or found for the structural changes in the articular tissues occurring during the course of so-called degenerative joint diseases. Thus, no therapeutic measures are available that act on underlying disease mechanisms and therefore slow down or halt the progression of tissue degradation in joints affected by erosive hand OA. The current standard treatment of care in these patients is limited to symptomatic therapy to reduce pain.

There is still lack of agreement concerning the nature and specificity of erosive IP joint OA. Obviously, in erosive IP OA an important bone resorption is noted in the subchondral bone of IP finger joints, this bone resorption is readily visualized on conventional radiographs (Figure 1). The osteolytic ‘erosive’ lesions result in the collaps of the subchondral plate which supports the overlaying articular cartilage.[5,21] This is compatible with a pathologic osteoclast activity supported by the effects of RANKL (Receptor Activator of Nuclear Factor kappa-β Ligand).[22] RANKL is a key driver of maturation and activation of osteoclasts in bone in health and disease.[22] In pathologic conditions, RANKL can be strongly induced in a variety of cell types including stromal cells under the influence of locally produced proinflammatory cytokines such TNFα [23,24] and IL-1β.[25,26]
At the same time, a resorption of articular cartilage of the affected IP joints is also noted. As a result, the joint space gradually disappears on X-rays. Likely key factors in this process are TNF and IL-1 which both have important catabolic effects on human chondrocytes.[27] Indeed, during the course of the disease inflammatory processes in the synovial membrane of IP finger joints could be visualized.[28,29] Cytokines release thereof will have important catabolic effects on the neighbouring chondrocytes.

Thus, similar as observed in other destructive processes noted in inflammatory rheumatic diseases, the **TNF→ IL-1→ RANKL-pathway** appears to be a key therapeutic target in erosive hand OA.

Blockade of these cytokines has shown to delay ongoing tissue destruction in murine arthritis and in rheumatoid arthritis in human. [30,31,32,33,34]

Recently, TNFα-blockade was shown to retard the progression of joint damage in erosive IP finger joint OA.[35]

Considering the analogies between rheumatoid arthritis and erosive IP OA in the metabolic pathways that mediate tissue destruction, and the lack of any structure modifying treatment option in the latter, a pilot study exploring the effects of Denosumab on ongoing tissue destruction in IP finger joint OA is proposed.

1.2 Denosumab

Denosumab (Amgen), is a fully human monoclonal antibody designed to inhibit RANKL

(RANK Ligand). RANKL binds to RANK, which exists as a cell surface receptor molecule on “pre”-osteoclasts: precursors of osteoclasts.

Binding of RANKL to RANK acts as the primary signal for bone removal in normal physiological bone remodeling and in a number of pathological conditions, e.g. malignant tumors and bone metastasis.

Activation of RANK by RANKL promotes the maturation of pre-osteoclasts into osteoclasts. Denosumab inhibits osteoclasts’ maturation, function and survival by binding to and inhibiting RANKL. This mimics the natural action of osteoprotegerin, an endogenous RANKL inhibitor that presents with decreasing concentrations in patients who are suffering from osteoporosis. This protects bone from degradation, and helps to counter the progression of the disease.

Denosumab was approved by the EMA for use in postmenopausal women with osteoporosis at increased risk for fracture at the dose of 60 mg sc every 6 months (Prolia®), and for the prevention of skeletal-related events in patients with bone metastasis from solid tumors at the dose of 120 mg every 4 weeks (XGEVA®).
More recently, denosumab was shown to retard the progression of structural lesions in rheumatoid arthritis, an unapproved indication for the drug.[33,34] Its dosing and safety profile depended on the different medical conditions in which the drug was used. Patients with osteoporosis and rheumatoid arthritis received 60 mg and up to 180 mg injected SC, every 6 months, respectively.

Experience from clinical studies indicates that side effects depend on the dosage.

According to Prolia® Summary of Product Characteristics (SmPC) [36], pain in extremities and musculoskeletal pain (including back pain and joint pain) were among the most common adverse reactions.
In patients treated for osteoporosis a rare unwanted effect included low calcium levels, especially when in case of an impaired kidney function. Patients must therefore be adequately supplemented with calcium and vitamin D levels before starting and during denosumab therapy. In the postmarketing setting, rare cases of severe symptomatic hypocalcaemia have been reported. Clinical monitoring of calcium level is recommended before each dose and, in patients predisposed to hypocalcaemia, within two weeks after the initial dose.
There have been rare cases of atypical femoral fracture reported in association with Prolia.
Infections of the urinary and respiratory tracts were reported as well as cellulitis, ear infection and diverticulitis. The SmPC includes a Warning Statement regarding skin infections (predominantly cellulitis) leading to hospitalization. It has been proposed that this increase in infections under denosumab treatment might be connected to the role of RANKL in the immune system.
Cataracts, constipation, skin rashes and eczema were also seen.
Osteonecrosis of the jaw (ONJ) was reported rarely in Prolia osteoporosis clinical development program. Primarily, at the high dosages used in patients with bone metastases, similarly to bisphosphonates, denosumab appeared to be implicated in increasing the risk of osteonecrosis of the jaw (ONJ) especially following extraction of teeth or oral surgical procedures.

In the post-marketing setting, rare events of drug-related hypersensitivity, including rash, urticaria, facial swelling, erythema, and anaphylactic reactions have been reported.

In the FREEDOM extension study[37,38], with up to 8 years of denosumab 60 mg Q6M exposure, the incidence rates of adverse events did not increase over time.

Denosumab safety data were reported in RA phase 2 studies[33,34]. The safety profile appears to be consistent with that in patients with postmenopausal osteoporosis. Denosumab did not have an effect on RA disease activity, as measured by the ACR response criteria, the DAS28 scores, and the occurrence of RA flares.

1.3 Rationale for study design

In RA, the initial changes are seen in the synovium where inflammatory lymphomyeloid cells massively produce TNF, and secondarily, IL-1 and RANKL. These two cytokines are responsible for the invasion of the adjacent cartilage and bone by the inflamed and proliferative synovial pannus.

In erosive IP joint OA, the osteolytic changes in subchondral bone occur before or concurrently with resorption of cartilage. The primary drivers of the cartilage damage thus are these osteolytic processes in the subchondral bone area and the collapse of the subchondral bone plate. RANKL is the cytokine primarily responsible for this osteolytic (osteoclast) activity.

The enhanced osteoclast activity and tissue remodeling initially seen in arthritic IP joint bone is clearly illustrated in figure 1.

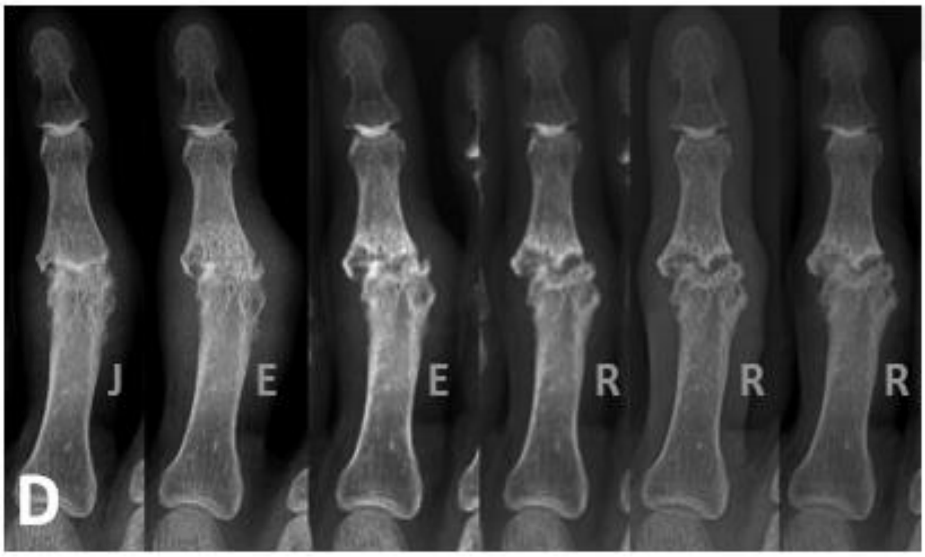

Figure 1: radiographic progression of a proximal IP joint from 'J' phase with loss of joint space to the 'E' phase with osteolytic activity in the subchondral bone area, and final remodeling of the destroyed tissues (R). Radiographs were taken with 6-months interval.

The effect of TNF alpha inhibitors on disease progression, previously seen in erosive IP joint OA[24], was an indirect effect on osteoclast activation. Obviously, this effect would be larger by directly inhibiting osteoclasts with Denosumab. Once the erosive process is blocked with

Denosumab, subchondral bone remodeling will be inhibited and one should see preservation of joint structure.

A proof-of-concept study is proposed herein to test the ability of repeated administration of denosumab to control the structural damage– and thus to maintain hand function - in erosive hand OA. These tests will be conducted compared to placebo during a first placebo controlled double-blind phase but also in a second open-label phase in which all subjects will receive denosumab. The 2 main factors that support conducting this second open-label phase are the following:
- This would enable the Long-term outcome assessment with the cumulative exposure over time; more substantial effect would be expected.
- The open label with help supporting patients' engagement in a placebo trial where no disease modifying drugs exist.

The adequate dose of denosumab should completely inhibit the erosive process in order to fully test the hypothesis. In the phase 2 RA studies [33,34,], the higher dose or shorter interval dosing regimen showed an earlier or a trend to more inhibition of bone destruction respectively. Considering further the well-established safety profile for denosumab at high doses, a higher frequency for denosumab 60 mg is proposed: denosumab 60 mg sc every 3 months.

1.4 Hypotheses

The main hypothesis is that the repeated administration of denosumab 60 mg every 3 months in erosive hand OA can inhibit structural progression of already affected joints and prevent occurrence of newly affected joints.
As it has been shown that denosumab, reduces structural damage in RA while having no effect on clinical symptoms [34], no clinical benefit is expected within the one-year period of this study. So, the effects of denosumab on the clinical manifestations of the disease will only be part of an exploratory study.

2. Study Objectives and Endpoints

The objective of this proof of concept study is to investigate the efficacy of denosumab 60 mg sc every 12 weeks for 48 weeks as a therapeutic intervention in erosive IP joint OA. In general, the expected outcome of this study would be the control of the structural damage.

Changes in the architecture of the joint will be assessed by the GUSS™. This score system allows an overall score to be calculated for an affected IP joint over time. The overall score is the sum of scores obtained for 3 compartments of the IP finger joint: the synovial space (articular cartilage), the subchondral bone plates and the subchondral bone area at each side of the synovial space. Overall scores, as well as scores for each individual compartment can be taken into consideration. Examples of the calculated scores for 2 different IP joints are given in appendix 1.

The **primary objective** is to assess the effect of denosumab on the reduction of radiographic erosive progression using GUSS™ (Ghent University Score System).
The **primary endpoints of this objective** is the change in the negative evolution in GUSS™ scores in the target IP joints from baseline to week 24.

Other endpoints are the changes in the negative evolution of GUSS™ scores in the target IP joints from week 24 to week 48 and from baseline to week 48.

The **secondary objective** is to evaluate a reduction in radiographic erosive progression as defined by diminishing the appearance of new erosive IP finger joints.
This will be assessed by 2 endpoints:
1. the number of patients that develop new erosive IP joints ('S/J' to 'E' phases) at 48 weeks.
2. the number of 'S/J' IP joints that develop 'E' phases at 48 weeks.

Radiological score systems are given in appendix 1.

The **exploratory** objective is to assess if denosumab provides clinical benefits (improvement of pain and functional limitations) compared to placebo. We will also evaluate the impact on ultrasonography and DEXA.
The endpoints of this objective are:
1. Changes in clinical and patient recorded outcome measures from baseline (day 1) to week 48 after administration of denosumab compared to placebo. The following outcome measures will be recorded: AUSCAN (AUStralian CANadian Osteoarthritis Hand Index), FIHOA (Functional Index of Hand Osteoarthritis), Pain on VAS scale, consumption of analgesics (paracetamol)/NSAIDs to be recorded by each patient on a diary, tenderness upon pressure, diameter of selected target joints, and grip strength of both hands.
2. Changes in sonographic inflammatory signals from baseline at week 12 and 48. Inflammatory changes will be assessed by measuring the amount of effusion and Power Doppler signal (scoring on a semi-quantitative scale).
3. Effect of denosumab on bone mass densitometry score in this group of patients compared to placebo from baseline to week 48. Changes from baseline (day 1) in T-score at lumbar spine and hip measured by bone densitometry at week 48 after administration of denosumab compared to placebo.

Other exploratory endpoints are to describe the above radiographic progression parameters at the end of the open-label phase.

Safety-objective
The safety profile of denosumab 60 mg (Prolia®) every 6 months in postmenopausal women with osteoporosis at increased risk of fracture is well established (Prolia SmPC). This study will assess the safety of the administration of denosumab 60 mg every 3 months in the population of patients with erosive OA. Safety evaluations will be made by recording the incidence of AE/SAE (see also paragraph 8).

3. Experimental Plan

3.1 Study design and schematic

This is a randomized, double blind, placebo-controlled, one-site proof of concept study to investigate the effect of denosumab 60 mg every 12 weeks on the radiological evolution of erosive OA of the digital joints.
Two groups of 50 patients each will be enrolled in the study with a total treatment duration of 24 months (96 weeks): 48 weeks double-blind placebo controlled phase (denosumab (60 mg

sc every 12 weeks or placebo) followed by a 48-weeks open–label phase in which all subjects will receive denosumab 60 mg every 12 weeks in an “Open Label Design” type study.

Study schematic

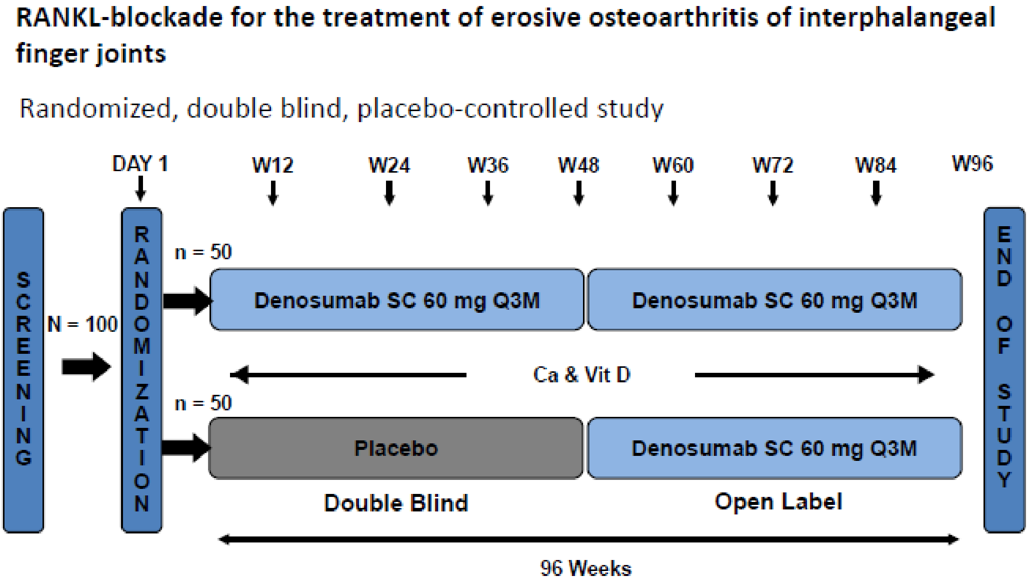

3.2 Number of sites

The study will be conducted in one site – the Ghent site in Belgium.

3.3 Number of subjects

A total of 100 subjects will be recruited in this study with an enrolment period of 18 months.

3.4 Estimated study duration

The total treatment duration per subject is 24 months (96 weeks). The expected total trial duration defined as the time from first patient first visit to last patient last visit is 42 months.

4. Subject Eligibility

4.1 Inclusion criteria

A subject will be eligible for study participation if he/she meets the following criteria:

- Males and females ≥ 30 years of age.
- Subjects with hand OA having suffered from transient inflammatory attacks of the interphalangeal finger joints characteristic for what has been termed 'inflammatory' or 'erosive' hand OA.
- Subjects with hand OA showing inflammatory signs, either clinically or ultrasonographically, of the interphalangeal finger joints.
- Subjects with hand OA in which at least 1 interphalangeal finger joint has the typical appearance on the X-rays of a 'J' or 'E' phase joint as defined by the criteria mentioned above.
- Subjects with hand OA where at least 1 interphalangeal finger joint in the 'J' or 'E' phase presents a palpable swelling.
- Able and willing to give written informed consent and to comply with the requirements of the study protocol.

4.2 Exclusion criteria

A subject will be excluded from the study if he/she meets any of the following criteria:

- Patients with known hypersensitivities to mammalian-derived drug preparations.
- Patients with clinically significant hypersensitivity to any of the components of Prolia.
- Current and/or Prior treatment with any investigational agent within 90 days, or five half-lives of the product, whichever is longer.
- Previous administration of denosumab from clinical trials or others (e.g. commercial use).
- Vitamin D deficiency [25(OH) vitamin D level < 20 ng/mL (< 49.9 nmol/L)]. Possibility of replenishment and re-screening.
- Subjects with current hypo- or hypercalcemia (normal serum calcium levels: 8.5-10.5 mg/dl or 2.12-2.62 mmol/L).
- Patients currently under bisphosphonate (BP) treatment or any use of oral BPs within 12 months of study enrollment or intravenous BPs or strontium ranelate within 5 years of study enrollment
- Prior use of any chondroprotective drug within 90 days e.g. chondroitin sulfate, glucosamine, avocado-soybean unsaponifiables, tetracyclins, corticosteroids.
- Prior use of any immunomodulating drug with possible effects on proinflammatory cytokine metabolism within 90 days a.o. corticosteroids, methotrexate, sulfasalazine, leflunomide, D-Penicillin, anti-malarials, cytotoxic drugs, TNF blocking agents.
- History of drug or alcohol abuse in the last year.
- Patients suffering from chronic inflammatory rheumatic disease (e.g. rheumatoid arthritis, spondylarthropathy, psoriatic arthritis, gout, chondrocalcinosis or other auto-immune diseases, e.g. systemic lupus erythematosus).
- History of cancer or lymphoproliferative disease other than a successfully and completely treated squamous cell or basal cell carcinoma of the skin or cervical dysplasia, with no recurrence within the last two years.
- History of any Solid Organ or Bone Marrow Transplant.
  - Comorbidities: significant renal function impairment (glomerular filtration < 30 ml/min/1.73m^2 or <50% of normal value), uncontrolled diabetes, unstable ischemic

heart disease, congestive heart failure (NYHA III, IV), uncontrolled hypo or hyperparathyroidism, active inflammatory bowel disease, malabsorption, liver failure or chronic hepatic disease (serum AST/ALT levels 3 times above normal), recent stroke (within three months), chronic leg ulcer and any other condition (*e.g,*. indwelling urinary catheter) which, in the opinion of the investigator, would put the subject at risk by participation in the protocol.

- Subject has any kind of disorder that compromises the ability of the subject to give written informed consent and/or to comply with study procedures .
- Patient who is pregnant or planning pregnancy; if the female subject is of child-bearing age, she must use a valid mean of contraception during the study and for 9 months after last dose of study medication. For males with a partner of childbearing potential: subject refuses to use 1 effective methods of contraception for the duration of the study and for 10 months after the last dose of study medication.
- Female subjects who are breast-feeding.
- History of osteonecrosis of the jaw, and/or recent (within 3 months) tooth extraction or other unhealed dental surgery; or planned invasive dental work during the study.

5. Treatment and Study Procedures

5.1 Investigational product (see also paragraph 1.2)
The study drug used in this clinical trial is denosumab 60 mg subcutaneously every 3 months. It will be provided as sterile, solution for injection in 1 ml pre-filled syringes containing denosumab 60mg/ ml or placebo. Placebo for Denosumab will be presented in identical containers and stored/packaged the same as drug product denosumab. Denosumab prefilled syringe placebo product is supplied in a prefilled syringe as a sterile, single use, preservative free solution for subcutaneous injection. Each prefilled syringe contains 1 mL deliverable volume of buffer consisting of 10 mM sodium acetate, 5% (w/v) sorbitol, 0.01% (w/v) polysorbate 20, at a pH of 5.2. The IP is packed with 1 PFS per box. Both Denosumab and Placebo are manufactured by Amgen Inc, United States and released in the EU by Amgen Breda, Netherlands. Amgen will provide batch release certificates that will be made available with each shipment of the drug. Amgen will provide GMP certification and investigational medicinal product dossiers directly to the Belgian Agency in the regulatory submission by Amgen for this ISS. The injections will be given at the study site. Instructions for the drug handling, packaging and storage are provided in details below. Briefly, the drug will be given under the skin of the thigh, abdomen or upper arm. The clinical supplies should be stored in the refrigerator at 2-8°C. Do not freeze. Do not shake excessively. The clinical supplies must be protected from light by storing in the outer carton.

Patients who completed the 1-year interventional study will have the opportunity to enter a second 1-year open-label extension (OLE) study with Denosumab (60 mg every 12 weeks, SC). The 1-year radiographic progression of their IP finger joints will be monitored after 6 and 12 months of treatment in the OLE.

Drug Handling:
"Denosumab is supplied as a sterile, colorless to slightly yellow, preservative-free solution for injection in a 1mL prefilled syringe (PFS). The formulation of IP is 60 mg/mL denosumab per

mL, formulated with10 mM Sodium Acetate, 5% Sorbitol, 0.01% Polysorbate, to a pH of 5.2. Each PFS of IP is intended for single use only. The IP is packed with 1 PFS per box. Placebo for denosumab will be presented in identical containers and stored/packaged in the same way as drug product denosumab.

The IP is shipped by air courier maintained at 2°C to 8°C in a qualified shipper suitable for biological substance shipments. IP in a PFS will arrive in a secondary packaging container and should be immediately placed in a refrigerator maintained at 2°C to 8°C in a secured location until planned use. The set point for the refrigerator should be at 5°C.
IP must be properly labelled and dispensed in accordance with current ICH GCP and local/regional requirements prior to dispensing for administration.
Before preparation check that IP:

- is visually intact and suitable for use
- is not expired
- has not been subjected to any potential temperature excursion
- label of the box and vial is correct

Prior to administration, IP may be removed from the refrigerator and brought to room temperature (up to 25°C) in the original container. This generally takes 15 to 30 minutes. Do not warm IP in any other way. Once removed from the refrigerator, IP must not be exposed to temperatures above 25°C/77°F and must be used within 24 hours. If not used within this time duration, IP must be discarded. Do not freeze IP. Protect IP from light and heat. Avoid vigorous shaking. Preparation of the clinical supplies should be performed using aseptic techniques and under sterile conditions.

Administration of IP must be performed as the last procedure after all the other study procedures have been completed for the visit. All SC injections must be administered by authorized site personnel. All subjects will receive 1 SC injection at each dosing visit (of either 60mg/ml Denosumab or Placebo) administered in the subject's upper arm, upper thigh or abdomen by a trained and qualified staff member. The injection should not be administered in the same arm from which blood is drawn."

5.2 Reporting requirements for investigational product complaints:
The following could be considered potential product complaints that need to be reported to Amgen. The Investigator will use a Product Complaint Form as provided by Amgen to report any complaint. Should any such concerns or irregularities occur, the IP will not be used until Amgen confirms that it is permissible to use. Examples of Product Complaints:
• Packaging: for example, broken container or cracked container
• Devices: issues with delivery of IP by device
• Usage: for example, subject or healthcare provider cannot appropriately use the product
• Labeling: for example, missing labels, illegible labels, incorrect labels, and/or suspect labels
• Change in IP appearance: for example color change or presence of foreign material
• Unexpected quantity in bottle: for example number of tablets or amount of fluid
• Evidence of tampering or stolen material

5.3 Concomitant therapy
All patients will have a daily calcium (1000 mg) and vitamin D (880 IU) supplementation. Subjects who are current or previous users of denosumab will be excluded at screening (see exclusion criteria).

Concomitant medication: NSAIDs and analgesics are allowed throughout during the study, but the dosages are kept constant during the first 12 weeks. Patients will keep records of their daily use of symptom modifying drugs.

5.4 Study procedures and schedule of assessments

A screening visit will include a clinical assessment, a hand radiograph and the laboratory investigations required. These will comprise a calcium and vitamin D status, peripheral blood cell count (PBC), serum chemistry glucose levels, liver (ALT, AST, alkaline phosphatase) and kidney function (serum ureum, serum creatinine, GFR) tests, Bone turnover markers (BTM) and, if appropriate, a pregnancy test.
An electrocardiogram (ECG) is part of the screening program.
Dual energy X-ray absorptiometry (DXA) is optional.

Patients will be evaluated for risk factors for ONJ before starting treatment. A dental examination with appropriate preventive dentistry is recommended prior to treatment with Prolia in patients with concomitant risk factors.

The maximum window allowed between the screening visit and the baseline visit is of 3 weeks.

Upon selection, patients will be included in the study during **the baseline visit**, which will include a clinical examination and an ultrasound (US) exam of the IP joints. Magnetic resonance imaging (MRI[39]) of the hand is optional. Study products (denosumab/placebo) will then be administered on-site by the investigator/study nurse. Calcium and vit D supplementation will be installed.

Schedule of assessments are provided in detail as Appendix 2. Clinical assessment is the standard practice and will be detailed in the CRF and the SAP. Safety assessment is clarified in the safety paragraph.

At week 6: a clinical/safety evaluation is planned.
At week 12: clinical/safety assessment, PBC and serum chemistry, serum calcium levels and BTM, US. MRI of the hand is optional. Study products (denosumab/placebo) to be administered on-site by the investigator/study nurse.
At week 24: clinical/safety assessment, serum calcium levels, hand radiographs.
Study products (denosumab/placebo) to be administered on-site by the investigator/study nurse.
At week 36: clinical/safety assessment, serum calcium levels. Study products (denosumab/placebo) to be administered.
W36 is the timing for the last IP dose in the blinded period.
At week 48: clinical/safety assessment, US, hand radiographs. Serum calcium levels, PBC and serum chemistry (glucose levels, liver and kidney function tests, and BTM. Study products (denosumab/placebo) to be administered. DXA is optional.

The visit at week 48 is the first visit of the Open Label Extension (OLE) program, which will encompass clinical/ safety exams, laboratory tests and hand radiographs as indicated in the table. The clinical monitoring of serum calcium during the OLE phase will follow the same schedule as in the placebo controlled phase.

All patients will receive a denosumab injection at W48 after the above assessment. This would be the first denosumab dose administered in the open label phase.

Safety: Patients will be able to report any unwanted effect during the regular visits and through telephone contact at any time in between these visits. Clinical examination is part of this safety assessment. Templates for AE/SAE recording created by the Investigators will be used.

As unwanted effects – other than these reported in previous Prolia osteoporosis programs - are not expected, the collection of other laboratory safety data beyond week 12 during the randomized treatment phase is not arranged.

A negative pregnancy test will be an entry requirement in female premenopausal patients. Premenopausal patients at risk to become pregnant will be excluded if no valid anti-conceptive method is used. In practice, premenopausal women will be an absolute minority in this study population. During the study and during the OLE phase, pregnancy tests will be done before each injection of denosumab in these subjects.

6. Statistical and Analytical Plans

6.1 Efficacy analysis

Complete and specific details of the final statistical analysis will be described and fully documented in the Statisticap Analysis Plan (SAP). The SAP will be finalized prior to the database lock. The analysis will be performed using the statistical software package IBM SPSS .

Demographic and baseline characteristics will be summarized. The number of observations, mean, standard deviation, median, minimum and maximum will be summarized for continuous variables. Discrete variables will be summarized by counts and percentages.

The primary efficacy variables will be the changes from baseline to week 24 in radiographic outcome measures, more specifically changes in GUSS. The primary efficacy comparisons will be between the denosumab treatment group and the placebo treatment group using GEE modelling with treatment as factors and baseline radiographic scores as a covariate. Additional endpoints will be assessed because several assumptions are made in this pilot study that are derived from a previous clinical study with a TNF-α blocking agent. The kinetics of TNF inhibitors might be different from the kinetics of denosumab on the bone level because of the different mode of action. Therefore it is not possible to predict if a similar rapid response on GUSS™ scores will be observed. Since the whole study is a proof-of-concept and to guarantee that a later response will not be missed, the study period needs to be extended to 48 weeks and the GUSS changes between week 24 and week 48, as well as GUSS changes between baseline and week 48 will be assessed.
Other analyses of radiographic measures will be the number of patients that develop new erosive joints and the number of patients in which erosive joints start the process of remodeling between baseline and 48 weeks. From previous studies it is known that the anatomical phase scoring system is not a sensitive on short term as GUSS.

Exploratory efficacy endpoints including change in Total AUSCAN score and individual subdomain (pain, physical function and stiffness) scores from baseline, change in FIHOA scores from baseline, change in pain scales (VAS pain) from baseline, change in consumption of analgesics (paracetamol)/NSAIDs, changes in number of painful and tender joints from baseline will be analyzed similarly at week 48. Other exploratory endpoints, including the change in number of joints with effusion and/or Power Doppler signal by ultrasound, the change in HOAMRIS scores and the changes in bone densitometry measures from baseline will be analyzed. Additional details will be provided in the SAP.

Primary and exploratory analyses will be repeated on subgroups defined by presence of soft tissue swelling at baseline. Details of analyses of efficacy endpoints at different time points as well as subgroups of interest will be given in the SAP.

The primary and exploratory efficacy variables will be analyzed on the intent-to-treat (ITT) population, defined as all subjects who were randomized.To evaluate the impact of major protocol violations on the results of the study, additional analyses of the primary efficacy analysis may be conducted on the per protocol population, which consists of all ITT subjects who completed the study and are not major protocol violators. The safety population consists of all subjects who received at least one dose of double-blind study medication.

In general, mean change analyses to compare the denosumab and placebo treatment group will be performed using GEE modelling with treatment group as factor and correction for baseline radiographic damage. Correction will be made for possible dependency between joints in the same patient by using an exchangeable matrix. Categorical data will be summarized using frequencies and percentages. Continuous data will be summarized with the number of non-missing observations by mean, standard deviation, median, maximum, and minimum values. In addition to the analyses based on observed data, analysis with imputed missing data will be conducted for selected efficacy variables. The details of such sensitivity analyses will be provided in the SAP. All statistical tests will be conducted at $\alpha = 0.05$ level (two-sided), unless otherwise stated. The last evaluation prior to the first study drug will be used as baseline for all analyses.

6.2 Safety analysis

Safety analyses will be carried out using the safety population, which includes all subjects who received at least one dose of study drug. Treatment-emergent AEs and SAEs will be summarized and reported. The number and percentage of subjects experiencing adverse events will be provided by system organ class and Medical Dictionary for Drug Regulatory Activities (MedDRA) preferred term. In addition, summary of AEs by severity and relationship to study drug will be presented. Serious, severe AEs, or AEs that lead to premature study discontinuation will be listed and described in detail. Mean change in vital signs and laboratory variables at each visit will be summarized for all treated subjects, and compared between treatment groups using one way Analysis of Variance (ANOVA).

6.3 Determination of Sample size

From a placebo controlled trial with adalimumab, we learned that, the risk that an individual IP joint evolves from J/S phase to the E phase is 2-3% per year. This risk increases to 15% for joints with a clinical effusion and to 25% for a painful joint with effusion. Adalimumab therapy reduced this risk for these inflammatory joints from 25% to 3% .

From these data 50 patients in each arm are needed to demonstrate a similar effect of denosumab with a power of 80%.
This power analysis took into account the following assumptions:
1) denosumab has a similar effect as adalimumab
2) a mean of minimal 1 inflamed joint (effusion and painful) per patient at baseline and in case of inclusion of patients with non-inflammatory joints, a within patient independent risk to evolve from J/S to E phase.
3) 5% drop-out
4) The proposed study involves two treatment arms. The level of significance (α) is 0.05.
5) a similar background risk for evolution from J/S to E phase.

Considering the semi-quantitative outcome measure, GUSS, a second power analysis was performed. Several assumptions were made, based on data from a previous study (Verbruggen G et al. ARD 2012;71(6):891-8). Power calculation was performed based on the estimated difference in the semi-quantitative outcome measure, GUSS ™ over time. This outcome measure is selected to detect the radiographic progression in the selected joints after treatment. The following assumptions were made:

- the natural progression (mean change) that can be expected over a period of 6 months is + 24 units (data from the placebo treated group), the mean difference in GUSS™ change between the placebo and adalimumab treated group after 6 months was 25 units. This was considered as clinically significant since
- the smallest detectable difference of GUSS™ was calculated as 40 units (Verbruggen G et al. ARD 2010;69(5):862-7) and improved to 10 units after intensive training.
- the standard deviation of the mean change in GUSS™ is 29,
- based on the above data, a total change of at least (24+ 25) 49 units in GUSS™ in the treatment group is considered to be a clinical relevant effect from a treatment.

The proposed study involves two treatment arms. The level of significance (α) is 0.05. From previous studies performed at our department, an drop out rate of 5% can be expected.
A sample size of 25 patients in each treatment arm will have 80% power to detect a difference in mean change GUSS™ of 25 units between the placebo and treated group, assuming that the standard deviation is 29 using a t-test with a two-sided 0.05 level of significance.
Taking into account a drop out rate of 5%, a total of 27 patients (25/ 1 – 0.05) should be included in each arm.
Taken into consideration both outcome measures, a minimum of 50 patients is required in both treatment arms in order to provide sufficient power for the study.

7. Adverse Events/Adverse Event reporting

The investigator will monitor each subject for clinical and laboratory (serum Ca^{++} levels) evidence of adverse events on a routine basis throughout the study. The investigator will assess and record any adverse event in detail on the adverse event DRF including the date and time of onset, description, seriousness severity, time course, duration and outcome, relationship of the adverse event to study drug, an alternate etiology for events not considered "probably related" to study drug, final diagnosis/syndrome (if known) and any action(s) taken. Adverse events, whether in response to a query, observed by study-site personnel, or reported spontaneously by the subject, will be recorded.

All adverse events will be followed to a satisfactory conclusion.

7.1 Definitions

7.1.1. Adverse Event

An **adverse event** is defined as any untoward medical occurrence in a subject or clinical investigation subject administered a pharmaceutical product and which does not necessarily have a causal relationship with this treatment. An adverse event can therefore be any unfavorable and unintended sign (including an abnormal laboratory finding), symptom, or disease temporally associated with the use of a medicinal product, whether or not the event is considered causally related to the use of the product.

Such an event can result from use of the drug as stipulated in the protocol or labeling, as well as from accidental or intentional overdose, drug abuse, or drug withdrawal. Any worsening of a pre-existing condition or illness is considered an adverse event. Laboratory abnormalities and changes in vital signs are considered to be adverse events only if they result in permanent

or temporary discontinuation of treatment with denosumab, necessitate therapeutic medical intervention and/or if the investigator considers them to be adverse events.

An elective surgery/procedure scheduled to occur during a study will not be considered an adverse event. However, if a pre-existing condition deteriorates unexpectedly during the trial (*e.g.*, surgery performed earlier than planned), then the deterioration of the condition for which the elective surgery/procedure is being done will be considered an adverse event.

7.1.2. Serious Adverse Event

If an adverse event meets any of the following criteria, it is to be considered as serious:

**Death of Subject**	An event that results in the death of a subject.
**Life-Threatening**	An event that, in the opinion of the investigator, would have resulted in immediate fatality if medical intervention had not been taken. This does not include an event that would have been fatal if it had occurred in a more severe form.
**Hospitalization**	An event that results in an admission to the hospital for any length of time. This does not include an emergency room visit or admission to an outpatient facility.
**Prolongation of Hospitalization**	An event that occurs while the study subject is hospitalized and prolongs the subject's hospital stay.
**Congenital Anomaly**	An anomaly detected at or after birth, or any anomaly that results in fetal loss.
**Persistent or Significant Disability/Incapacity**	An event that results in a condition that substantially interferes with the activities of daily living of a study subject. Disability is not intended to include experiences of relatively minor medical significance such as headache, nausea, vomiting, diarrhea, influenza, and accidental trauma (*e.g.*, sprained ankle).
**Important Medical Event Requiring Medical or Surgical Intervention to Prevent Serious Outcome**	An important medical event that may not be immediately life-threatening or result in death or hospitalization, but based on medical judgment may jeopardize the subject and may require medical or surgical intervention to prevent any of the outcomes listed above (*i.e.*, death of subject, life-threatening, hospitalization, prolongation of hospitalization, congenital anomaly, or persistent or significant disability/incapacity). Examples of such events include allergic bronchospasm requiring intensive treatment in an emergency room or at home, blood dyscrasias or convulsions that do not result in inpatient hospitalization, or the development of drug dependency or drug abuse.
**Spontaneous Abortion**	Miscarriage experienced by study subject.
**Elective Abortion**	Elective abortion performed on study subject.

7.1.3. Adverse Event Severity

The investigator will use the following definitions to define/rate the severity of each adverse event:

**Mild**	The adverse event is transient and easily tolerated by the subject.
**Moderate**	The adverse event causes the subject discomfort and interrupts the subject's usual activities.
**Severe**	The adverse event causes considerable interference with the subject's usual activities and may be incapacitating or life-threatening.

7.1.4. Relationship to Study Drug

The investigator will use the following definitions to assess the relationship of the adverse event to the use of study drug:

**Probably Related**	An adverse event has a strong temporal relationship to study drug or recurs on re-challenge and another etiology is unlikely or significantly less likely.
**Possibly Related**	An adverse event has a strong temporal relationship to the study drug and an alternative etiology is equally or less likely compared to the potential relationship to study drug.
**Probably Not Related**	An adverse event has little or no temporal relationship to the study drug and/or a more likely alternative etiology exists.
**Not Related**	An adverse event is due to an underlying or concurrent illness or effect of another drug and is not related to the study drug (*e.g.*, has no temporal relationship to study drug or has a much more likely alternative etiology).

7.2. Adverse Event Reporting

Reporting will be consistent with current safety reporting standards. .Adverse events will be reported between the first dose administration of trial medication and the last trial related activity.
All AEs and SAE's will be recorded in the patient's file and in the CRF. All SAE's will be reported as described below.
SAE's occurring within a period of 30 days following the last intake of trial medication will also be handled as such if spontaneously reported to the investigator.

All serious adverse events (SAE) and pregnancies occurring during clinical trials must be reported by the local Principal Investigator within 2 working days after becoming aware of the SAE to:

- The local EC
- Bimetra Clinics of the University Hospital Ghent

This reporting is done by using the appropriate SAE form. For the contact details, see below.

It is the responsibility of the local Principal Investigator to report the local SAE's to the local EC.

In case the investigator decides the SAE is a SUSAR (Suspected Unexpected Serious Adverse Reaction), Bimetra Clinics will report the SUSAR to the Central EC and the CA within the timelines as defined in national legislation.

In case of a life-threatening SUSAR the entire reporting process must be completed within 7 calendar days. In case of a non life-threatening SUSAR the reporting proces must completed within 15 calendar days.

The first report of a serious adverse event may be made by telephone, e-mail or facsimile (FAX).

Contact details of Bimetra Clinics:

e-mail: bimetra.clinics@uzgent.be
tel.: 09/332 05 00
fax: 09/332 05 20

In the event of a serious, unexpected and related adverse event, the investigator will report this to the Amgen Affiliate by faxing the appropriate adverse event form within 24 hours of being made aware of the serious adverse event and simultaneously to Bimetra Clinics who will report the event to the local regulatory agency within the timelines as defined in the national legislation..
Please fax SAE form to [redacted] Pharmacovigilance Manager : Fax number 0800 80877

The investigator must provide the minimal information: i.e. trial number, subject's initials and date of birth, medication code number, period of intake, nature of the adverse event and investigator's attribution.
This report of a serious adverse event by telephone must always be confirmed by a written, more detailed report. For this purpose the appropriate SAE form will be used.
Pregnancies occurring during clinical trials are considered immediately reportable events. They must be reported as soon as possible using the same SAE form. The outcome of the pregnancy must also be reported.

If the subjects are not under 24-hour supervision of the investigator or his/her staff (out-patients, volunteers), they (or their designee, if appropriate) must be provided with a "trial card" indicating the name of the investigational product, the trial number, the investigator's name and a 24-hour emergency contact number.

8. Regulatory Obligations

8.1 Informed Consent

Signed informed consent will be obtained from the subject before any study procedures are undertaken, or before any medications are withheld from the subject in order to participate in this study. Subject may withdraw consent at any time without prejudice. All efforts will be made to continue the patient follow-up until the end of the study. At withdrawal, patients will be treated and assessed according to standard recommendations and as per latest guidance for contraception criteria in female subjects of child-bearing age or partners of childbearing potential (see exclusion criteria section 4.2).

8.2 Independent Ethics Committee/Institutional Review Board

The study will be declared at www.ClinicalTrials.gov and will comply with the principles of the Declaration of Helsinki. A copy of the study protocol will be submitted for approval to the ethical committee of Ghent University Hospital and to the Federal Agency for Medicines and Health Products *(FAGG; federal agentschap voor geneesmidddelen en gezondheidsproducten)*

9. Documentation relating to the clinical trial – Trial Master File

All documents related to the trial, e.g. study protocol, source documents, case report forms, … will be handled, stored and archived according to the EU Commission's Directive 2005/28/EC 63 Chapter 4.[40]

10. Publication Policy

The results of this study will be reported and published at conferences and in peer-reviewed clinical journals. Authorship publications will follow the Uniform Requirement for

Manuscripts Submitted to Biomedical Journals (International Committee of Medical Journal Editors, 2009), which states:
Authorship credit should be based on (1) substantial contributions to conception and design, acquisition of data, or analysis and interpretation of data; (2) drafting the article or revising it critically for important intellectual content; (3) final approval of the version to be published and (4) Agreement to be accountable for all aspects of the work in ensuring that questions related to the accuracy or integrity of any part of the work are appropriately investigated and resolved. Authors should meet conditions 1, 2, 3 and 4.
For further details , see http://www.icmje.org/recommendations/browse/roles-and-responsibilities/defining-the-role-of-authors-and-contributors.html.

11.References

1- Stecher RM, Hauser H : Heberden's nodes. VII. The roentgenological and clinical appearance of degenerartive joint disease of the fingers. AmJ Roentgenol. 59 :326-337,1948
2- Crain DC : Interphalangeal osteoarthritis. Characterized by painful, inflammatory episodes resulting in deformity of the proximal and distal articulations. JAMA. 175: 1049-1053,1961
3- Peter JB, Pearson CM, Marmor L : Erosive arthritis of the hands. Arthritis Rheum. 9: 365-388,1966
4- Ehrlich GE. Osteoarthritis beginning with inflammation. Definitions and correlations. JAMA. 232: 157-159,1975
5- Verbruggen G and Veys EM. Numerical scoring systems for the anatomic evolution of osteoarthritis of the finger joints. Arthritis Rheum. 1996;**39**:308-20.
6- Zhang Y, Niu J, Kelly-Hayes M, Chaisson CE, Aliabadi P, Felson DT. Prevalence of symptomatic hand osteoarthritis and its impact on functional status among the elderly: The Framingham Study. Am J Epidemiol 2002;**156**:S225.
7- Dahaghin S, Bierma-Zeinstra SMA, Reijman M, Pols HAP, Hazes JMW, Koes BW. Prevalence and determinants of one month hand pain and hand related disability in the elderly (Rotterdam study). Ann Rheum Dis 2005;**64**:99-104.
8 Wittoek R, Vander Cruyssen B, Verbruggen G. Predictors of functional impairment and pain in erosive osteoarthritis of the interphalangeal joints: comparison with controlled inflammatory arthritis.Arthritis Rheum.
9. Leeb BF, Sautner J, Andel L, Rintelen B. A scale for assessment and quantification of chronic rheumatoid affections of the hands. Rheumatology 2003; 42: 1173-78.
10- Dillon CF, Hirsch R, Rasch E, Gu Q. Symptomatic hand osteoarthritis in the United States: prevalence and functional impairment estim ates from the third U.S. National Health and Nutrition Examination Survey, 1991-1994. Am J Phys Med Rehabil. 2007;86:12-21.
11- Kellgren JH. Osteoarthrosis in patients and populations. Br Med J. 1961;2:1-6.
12- Lawrence JS, Bremner JM, Biers F. Osteoarthritis. Prevalence in the population and relationship between symptoms and X-Ray changes. Ann Rheum Dis. 1966;25:1-24.
13- Bagge E, Bjelle A, Valkenburg HA, Svanborg A. Prevalence of radiographic osteoarthritis in two elderly European populations. Rheumatology Int. 1992;12:33-8.
14- Mannoni A, Briganti MP, Di Bari M, Ferrucci L, Constanzo S, Serni U, Masotti G, Marchionni N. Epidemiological profile of symptomatic osteoarthritis in older adults: a population based study in Dicomano, Italy. Ann Rheum Dis. 2003 Jun;62:576-8.
15- Cobby M, Cushnaghan J, Creamer P, Dieppe P, Watt I. Erosive osteoarthritis: is it a separate disease entity? Clinical Radiology 1990;**42**:258-63.
16- Cavasin F, Punzi L, Ramonda R, Pianon M, Oliviero F Sfriso P, Todesco S. Prevalence of erosive osteoarthritis of the hand in a population from Venetian area. Rheumatismo 2004;56:46-50.
17- Punzi L, Ramonda R, Sfriso P. Erosive osteoarthritis. Best Pract Res Clin Rheumatol 2004 ;18:739-58.
18- Poole J, Sayer AA, Hardy R, Wadsworth M, Kuh D, Cooper C. Patterns of interphalangeal hand joint involvement of osteoarthritis among men and women: a British cohort study. Arthritis Rheum. 2003 48:3371-6.
19. Kwok WY, Kloppenburg M, Rosendaal FR, van Meurs JB, Hofman A, Bierma-Zeinstra SMA. Erosive hand osteoarthritis: its prevalence and clinical impact in the general population and symptomatic hand osteoarthritis. Ann Rheum Dis 2011;70:1238-42.

20. Haugen IK, Englund M, Aliabadi P, Niu J, Clancy M, Kvien TK, Felson DT. Prevalence, incidence and progression of hand osteoarthritis in the general population: the Framingham Osteoarthritis Study. Ann Rheum Dis 2011;70:1581-6.
21. Verbruggen G, Wittoek R, Vander Cruyssen B et al. Morbid anatomy of 'erosive osteoarthritis' of the interphalangeal finger joints: an optimised scoring system to monitor disease progression in affected joints. Ann Rheum Dis. 2010;69:862-7.
22. Suda T, Takahashi N, Udagawa N et al. Modulation of osteoclast differentiation and function by the new members of the tumor necrosis factor receptor and ligand families. Endocr Rev.1999;20:345-57.
23. Kobayashi K, Takahashi N, Jimi E, et al. Tumor necrosis factor alpha stimulates osteoclast differentiation by a mechanism independent of the ODF/RANKL-RANK interaction. J Exp Med 2000;191:275-86.
24. Komine M, Kukita A, Kukita T et al. Tumor necrosis factor-alpha cooperates with receptor activator of nuclear factor kappaB ligand in generation of osteoclasts in stromal cell-depleted rat bone marrow cell culture. Bone 2001;28:474-83.
25. Wei S, Kitaura H, Zhou P et al. IL-1 mediates TNF-induced osteoclastogenesis. J Clin Invest 2005;115:282-90.
26- Zwerina J, Redlich K, Polzer K et al. TNF-induced structural joint damage is mediated by IL-1. Proc Natl Acad Sci U S A. 2007;104:11742-7.
27. Lefebvre V, Peeters-Joris C, Vaes G. Modulation by interleukin 1 and tumor necrosis factor alpha of production of collagenase, tissue inhibitor of metalloproteinases and collagen types in differentiated and dedifferentiated articular chondrocytes. Biochim Biophys Acta. 1990;1052:366-78.
28. Wittoek R, Carron P, Verbruggen G. Structural and inflammatory sonographic findings in erosive and non-erosive osteoarthritis of the interphalangeal finger joints. Ann Rheum Dis. 2010;69:2173-6.
29. Jans L, De Coninck T, Wittoek R et al. 3 T DCE-MRI assessment of synovitis of the interphalangeal joints in patients with erosive osteoarthritis for treatment response monitoring. Skeletal Radiol. 2013;42:255-60.
30- Bathon JM, Martin RW, Fleischmann RM, Tesser JR, Schiff MH, Keystone EC, Genovese MC, Chester Wasko M, Moreland LW, Weaver AL, Markenson J, Finck BK. A Comparison of Etanercept and Methotrexate in Patients with Early Rheumatoid Arthritis. NEJM. 2000; 343:1586-93.
31- Lipsky PE, van der Heijde DMFM, St. Clair EW, Furst DE, Breedveld FC, Kalden JR, Smolen JS, Weisman M, Emery P, Feldmann, Gregory R. Harriman GR, Maini RN. Infliximab and Methotrexate in the Treatment of Rheumatoid Arthritis. NEJM. 2000; 343:1594-602.
32. Alten R, Gram H, Joosten LA, van den Berg WB, Sieper J, Wassenberg S, Burmester G, van Riel P, Diaz-Lorente M, Bruin GJ, Woodworth TG, Rordorf C, Batard Y, Wright AM, Jung T. The human anti-IL-1 beta monoclonal antibody ACZ885 is effective in joint inflammation models in mice and in a proof-of-concept study in patients with rheumatoid arthritis. Arthritis Res Ther. 2008;10:R67.
33. Cohen SB, Dore RK, Lane NE, Ory PA, Peterfy CG, Sharp JT, van der Heijde D, Zhou L, Tsuji W, Newmark R; Denosumab Rheumatoid Arthritis Study Group. Denosumab treatment effects on structural damage, bone mineral density, and bone turnover in rheumatoid arthritis: a twelve-month, multicenter, randomized, double-blind, placebo-controlled, phase II clinical trial. Arthritis Rheum. 2008;58:1299-309.

34. Ishiguro N, Tanaka Y, Yamanaka H, Yoneda T, Ohira T, Okubo N, Genant HK, van der Heijde D and Takeuchi T. Consistent Inhibition of Bone Destruction By Denosumab in Important Subgroups of Japanese Patients with Rheumatoid Arthritis. Arthritis Rheumatol 2014; 66:11(Suppl): S831/Presented at ACR meeting 2014
35. Verbruggen G, Wittoek R, Cruyssen BV, Elewaut D. Tumour necrosis factor blockade for the treatment of erosive osteoarthritis of the interphalangeal finger joints: a double blind, randomised trial on structure modification. Ann Rheum Dis. 2012 Jun;71(6):891-8.
36. Prolia SmPC. Sections 4.4 & 4.838.
37. Bone HG, Chapurlat R, Brandi ML, Brown JP, Czerwinski E, Krieg MA, Mellström D, Radominski SC, Reginster JY, Resch H, Ivorra JA, Roux C, Vittinghoff E, Daizadeh NS, , Bradley MN, Franchimont N, Geller ML, Wagman RB, Cummings SR, Papapoulos S. The effect of three or six years of denosumab exposure in women with postmenopausal osteoporosis: results from the FREEDOM extension. Clin Endocrinol Metab. 2013;98:4483-92.
38. Papapoulos S, et al. Eight Years of Denosumab Treatment in Postmenopausal Women with Osteoporosis: Results From the First Five Years of the FREEDOM extension. WCO-IOF-ESCEO Congress 2014. Osteoporis Int 2014; 25 (Suppl 2):S118
39. Haugen IK, Østergaard M, Eshed I, McQueen FM, Bird P, Gandjbakhch F, Foltz V, Genant H, Peterfy C, Lillegraven S, Haavardsholm EA, Bøyesen P, Conaghan PG.3. Iterative development and reliability of the OMERACT hand osteoarthritis MRI scoring system.
J Rheumatol. 2014 Feb;41(2):386-91
40. http://eur-lex.europa.eu/LexUriServ/LexUriServ.do?uri=OJ:L:2005:091:0013:0019:en

12.Appendices

Appendix 1. Scoring systems

A. Categorical scoring system was proposed for the progressive radiographic changes in IP finger joint OA. These changes were characterized by complete loss of the joint space preceding or coinciding with the appearance of subchondral cysts eroding the entire subchondral plate. These erosive episodes subsided spontaneously and were followed by processes of repair.[28]

The anatomical phases in the evolution of IP finger joint OA are the following.

Normal ('N') joints: no signs of OA.
Stationary ('S') phase: classical appearance of OA. Small ossification centers and osteophytes are present at the joint margins. They can both increase in size and discrete narrowing of the joint space can occur.
Loss of joint space ('J' phase): after remaining for a variable time in the stationary phase, some joints (almost exclusively PIPs or DIPs) become destroyed. The joint space completely disappears within a relatively short period of time.
Erosive ('E') phase: concurrently with or shortly after the disappearance of the articular cartilage (J phase), the subchondral plate becomes eroded. The appearance is that of a pseudo-enlargement of an irregular joint space. Roentgenograms obtained at yearly intervals showed that changes in phases from 'S' over 'J' to 'E' could occur within one year. This destructive 'J' and 'E' phases are always followed by repair or remodeling.
Remodeling ('R') phase: new irregular sclerotic subchondral plates are formed, and in between these a new joint space becomes visible. Huge osteophytes are formed during this phase. No further evolution is seen in remodeled joints.

B. A quantitative radiographic scoring system, the Ghent University Scoring System, GUSS® [29], is a reliable method to score radiographic change over time in erosive IP OA and detects more progression over a shorter period of time than the classical scoring system. Erosive progression and signs of repair or remodeling are then scored by indicating the proportions of normal subchondral bone, subchondral plate and joint space over time.

The subchondral bone area. The proportions of the subchondral bone area with normal/abnormal-looking bone architecture were assessed in a quadrangle square of which the side equalled the width of the joint space. The joint space was positioned in the centre of this square (figure 2A). In this square, regions where osteolytic activity and remodelling caused a disarrangement of the trabecular pattern, as well as areas where a complete loss of the trabecular structure had occurred, are defined.
Identifiable osteolytic subchondral bone areas are marked on the radiographs and proportions of remaining intact subchondral bone will be calculated, considering the delineated IP joint area being the 100% value.

The subchondral bone plate. In an IP joint that had completely lost its joint space, an existing subchondral plate was defined as a regular radio-opaque linear structure within the position of the original joint space. When the joint space was still identifiable, the subchondral bone plate was identified as a regular linear radio-opaque bone margin flanking the joint space.

Identifiable linear subchondral plate structures were marked on the radiographic images and proportions of remaining subchondral bone plate were computed, considering a twofold joint space width being the 100% value (figure 2B).

The joint space was recognized as a radiotranslucent area bordered with two subchondral plates. Identifiable joint spaces were marked on the radiographic images. Proportions of remaining joint space were estimated as the proportion of the joint width, considering the total joint space width being the 100% value (figure 2B).

Computation of the changes in IP joints in “J”, “E” and “E/R” phases. Pictures from the IP joints at three time points in the correct sequence will be read and used by the readers to evaluate the extent of the pathological changes in subchondral bone architecture, and to estimate the presence/absence of both subchondral bone plate and synovial joint space. Proportional changes in these three variables will be recorded. The sum of the three separate scorings constituted the total IP joint score. Equal weight will be attributed to each of the subdomains.

Appendix 2. Overall assessments

	admin dmab/plac	clinical assessm	safety	laboratory PBC	serum chem	BTM	25OH vit D	Ca++	preg test*	CR hand	US hand	DXA	ECG
SCREENING		X		X	X	X	X	X	X	X		X	X
BASELINE	X	X	X								X		
WEEK 6		X	X										
WEEK 12	X	X	X	X	X	X		X	X		X		
WEEK 24	X	X	X					X	X	X			
WEEK 36	X	X	X					X	X				
WEEK 48	X	X	X	X	X	X		X	X	X	X	X	
WEEK 60	X	X	X					X	X				
WEEK 72	X	X	X			X		X	X	X			
WEEK 84	X	X	X					X	X				
WEEK 96		X	X	X	X	X		X	X	X		X	

* if appropriate

dmab: denosumab; plac: placebo; PBC: peripheral blood cell count; chem: chemistry; BTM: bone turnover markers
preg: pregnancy - sticks to be provided by the rheumatology dept.; CR: conventional radiography; US: ultrasound;
MRI: magnetic resonace imaging; ECG: electrocardiogramDXA: dual energy X-ray absorptiomery

Basic Serum chemistry will include urea, creatinine, ASAT, ALAT, Albumin. Depending on the individual patient, additional parameters may be added.

W36 is the timing for the last IP dose in the blinded period. All patients will receive a denosumab injection at W48 after the assessment. This would be the first denosumab dose administered in the open label phase.

Study Protocol

RANKL-blockade for the treatment of erosive osteoarthritis (OA) of interphalangeal finger joints

Randomized, double blind, placebo-controlled study to evaluate the efficacy of denosumab 60mg sc every 3 months in patients with erosive osteoarthritis of the interphalangeal finger joints

Principal Investigators:
[redacted]

Dept. of Rheumatology – Ghent University Hospital

EudraCT number: 2015-003223-53
Protocolnumber: AGO/2015/008

[redacted]

[redacted]

[redacted]

Protocol Signature Page

Principal/Chief Investigator signature

I confirm that I have read and understood protocol version ~~xx dated xx January 2015~~ 2.0 - 16 FEBRUARY 2016. I agree to comply with the study protocol, the principals of GCP, research governance, clinical trial regulations and appropriate reporting requirements. [redacted] 25/4/17

Signature.. Date 18 FEB 2016

Print name [redacted]

Protocol synopsis

**Study Type**	**I**nvestigator **S**ponsored **S**tudy
**Funder**	Amgen
**Study Design**	This is a randomized, double blind placebo controlled one-site proof-of-concept study in subjects with erosive osteoarthritis (OA) of interphangeal (IP) finger joints.<br>A total of 100 subjects will be enrolled into the study: 48 weeks placebo controlled double-blind phase with denosumab 60 mg every 12 weeks, followed by a 48-week open-label phase in which all subjects will receive denosumab.
**Investigational Therapy**	Denosumab 60 mg subcutaneous injection every 12 weeks. All subjects will receive Calcium/vit D supplementation.
**Efficacy Objectives**	The **primary objective** is to assess the effect of denosumab on the reduction of radiographic erosive progression using GUSS™ (Ghent University Score System).<br>The **secondary objective** is to assess the effect of denosumab on the reduction of radiographic erosive progression as defined by diminishing the appearance of new erosive IP finger joints.<br>The **exploratory objective** is mainly to assess the effect of denosumab on clinical variables, as well as ultrasonography and DEXA parameters.
**Main Endpoints**	**Primary Endpoint:** The change in the negative evolution of GUSS™ scores in the target IP joints from baseline to week 24.<br>**Other Endpoints:** 1) The change in the negative evolution of GUSS™ scores in the target IP joints from week 24 to week 48 and from baseline to week 48. 2) The number of patients that develop new erosive IP joints ('S/J' to 'E' phase joints) at 48 weeks; 3) The number of 'S/J' IP joints that develop 'E' phases at 48 weeks.
**Hypothesis**	**The main hypothesis** is that the repeated administration of denosumab 60 mg Q3 months can lead to reduce structural damage in erosive hand OA.
**Study Sites**	1 site – the Ghent site
**Subjects**	100 subjects
**Enrolment**	18 months
**Main Eligibility Criteria**	Males and females ≥ 30 years of age, with hand erosive OA:<br>1) having suffered from transient inflammatory attacks of the IP finger joints<br>2) showing at the time of enrolment inflammatory signs and at least one IP finger joint with the typical X-rays appearance of a 'J' or 'E' phase joint

**Study treatment Duration**	96 weeks

Table of Contents

- Protocol Title and Investigators
- Protocol signature page
- Protocol synopsis

1. Background and Rationale
1.1 Disease background
1.2 Denosumab
1.3 Rationale for study design
1.4 Hypotheses
2. Study Objectives and Endpoints
3. Experimental Plan
3.1 Study design and schematic
3.2 Number of sites
3.3 Number of subjects
3.4 Estimated study duration
4. Subject Eligibility
4.1 Inclusion criteria
4.2 Exclusion criteria
5. Treatment and Study Procedures
5.1 Investigational product
5.2 Reporting requirements for investigational product complaints
5.3 Concomitant therapy
5.4 Study procedures and schedule of assessments
6. Statistical and Analytical Plans
6.1 Efficacy analysis
6.2 Safety analysis
6.3 Determination of sample size
7. Adverse Events/Adverse Event reporting
7.1 Definitions
7.1.1. Adverse Event
7.1.2. Serious Adverse Event
7.1.3. Adverse Event Severity
7.1.4. Relationship to Study Drug
7.2. Adverse Event Reporting
8. Regulatory obligations
8.1Informed Consent
8.2 Independent Ethics Committee/Institutional Review Board
9. Documentation relating to the clinical trial- trial master file
10. Publication Policy
10. References
11. Appendices
Appendix 1. Scoring systems
Appendix 2. Overall assessments

1. Background and Rationale

1.1 Disease background

Erosive osteoarthritis (OA) of the interphalangeal (IP) finger joints is considered an inflammatory subset of osteoarthritis of the hand. Its inflammatory clinical presentation and destructive nature are unmistakable. [1,2,3,4,5] The cumulation of destructive changes in the IP joints eventually results in considerable disability.[6,7,8] There are no significant differences in hand function, stiffness and level of pain between patients with hand OA and rheumatoid arthritis. Scores for both patient groups differ significantly from those of healthy controls.[9]
Patients with erosive OA show more functional impairment and significantly more pain compared to patients with controlled inflammatory arthritis affecting the hands. The acquired structural damage of the IP joints due to destructive/reparative phenomena is the largest contributor to functional limitations.[8]
Radiological prevalence of moderate to severe hand OA is estimated to occur in 7.3% (2.65 million) US adults aged 60+ years.[10] Similar data have been reported in European countries.[7,11,12,13,14]

A significant proportion of these patients suffer from the erosive type of hand OA. In a prospective study of 500 consecutive patients attending a rheumatology clinic with symptomatic limb joint OA, 4.8% cases were identified with erosive IP joint OA.[15]

In a survey on the entire health district in the Venetian area, 2.2% out of 640 subjects aged 40+ years had erosive OA of their IP joints.[16] Mainly women in the perimenopauzal age were affected.[17]

Even higher prevalences were seen in a British cohort study[18] on 2.986 people[18]. Numbers in this study were based on clinics and the authors proposed that a proportion of their polyarticular cases were “inflammatory types of OA in association with erosions”. This assumption was based on an earlier study where clinical examination was validated against hand radiography (Egger et al., J Rheumatol 1995;22:1509–13).
Though the proportions of “erosive IP OA” reported here were probably overrated, the prevalence of what is considered to be “erosive IP OA” in this 53 years of age population was twice as high in women (10,6%), compared to men (5,9%).
More recently, these data were confirmed in 2 large population studies where the prevalence of radiographic erosive IP OA in subjects over 55 years of age ranged between 5.0 and 9.9%.[19,20]. The prevalence for men was lower at 3.3%.

These studies showed that erosive type of hand OA occurred predominantly in women.
Haugen IK et al. et al.[20] defined erosive IP OA at a joint level as Kellgren/Lawrence ≥2 plus erosions. The authors reported a prevalence of erosive IP OA in women of 9,9%, 3 times as high as in men (3,3%). In essence, the Kwok W-Y et al. figures[19] agree with the data above.

Moreover, the Haugen IK et al.[20] reported that symptomatic OA was twice as high in women (15,9%), compared to men (8,2%). Symptomatic OA here was defined as Kellgren/Lawrence stage ≥2 plus pain/aching/stiffness.

From these epidemiological studies we can conclude that the incidence of erosive OA of the IP finger joints ranges from five to ten percent particularly in women.

The aggressive destructive nature of the erosive OA is only recognized late in the disease and the radiological image of the "exhausted" final phase mimics a robust OA. Therefore, the disease was hitherto regarded as a form of primary OA - a degenerative joint disease that is caused by biomechanical overload of the joint structures. There is so far no therapy sought or found for the structural changes in the articular tissues occurring during the course of so-called degenerative joint diseases. Thus, no therapeutic measures are available that act on underlying disease mechanisms and therefore slow down or halt the progression of tissue degradation in joints affected by erosive hand OA. The current standard treatment of care in these patients is limited to symptomatic therapy to reduce pain.

There is still lack of agreement concerning the nature and specificity of erosive IP joint OA. Obviously, in erosive IP OA an important bone resorption is noted in the subchondral bone of IP finger joints, this bone resorption is readily visualized on conventional radiographs (Figure 1). The osteolytic 'erosive' lesions result in the collaps of the subchondral plate which supports the overlaying articular cartilage.[5,21] This is compatible with a pathologic osteoclast activity supported by the effects of RANKL (Receptor Activator of Nuclear Factor kappa-β Ligand).[22] RANKL is a key driver of maturation and activation of osteoclasts in bone in health and disease.[22] In pathologic conditions, RANKL can be strongly induced in a variety of cell types including stromal cells under the influence of locally produced proinflammatory cytokines such TNFα [23,24] and IL-1β.[25,26]
At the same time, a resorption of articular cartilage of the affected IP joints is also noted. As a result, the joint space gradually disappears on X-rays. Likely key factors in this process are TNF and IL-1 which both have important catabolic effects on human chondrocytes.[27] Indeed, during the course of the disease inflammatory processes in the synovial membrane of IP finger joints could be visualized.[28,29] Cytokines release thereof will have important catabolic effects on the neighbouring chondrocytes.

Thus, similar as observed in other destructive processes noted in inflammatory rheumatic diseases, the **TNF→ IL-1→ RANKL-pathway** appears to be a key therapeutic target in erosive hand OA.

Blockade of these cytokines has shown to delay ongoing tissue destruction in murine arthritis and in rheumatoid arthritis in human. [30,31,32,33,34]

Recently, TNFα-blockade was shown to retard the progression of joint damage in erosive IP finger joint OA.[35]

Considering the analogies between rheumatoid arthritis and erosive IP OA in the metabolic pathways that mediate tissue destruction, and the lack of any structure modifying treatment option in the latter, a pilot study exploring the effects of Denosumab on ongoing tissue destruction in IP finger joint OA is proposed.

1.2 Denosumab

Denosumab (Amgen), is a fully human monoclonal antibody designed to inhibit RANKL

(RANK Ligand). RANKL binds to RANK, which exists as a cell surface receptor molecule on “pre”-osteoclasts: precursors of osteoclasts.

Binding of RANKL to RANK acts as the primary signal for bone removal in normal physiological bone remodeling and in a number of pathological conditions, e.g. malignant tumors and bone metastasis.

Activation of RANK by RANKL promotes the maturation of pre-osteoclasts into osteoclasts. Denosumab inhibits osteoclasts’ maturation, function and survival by binding to and inhibiting RANKL. This mimics the natural action of osteoprotegerin, an endogenous RANKL inhibitor that presents with decreasing concentrations in patients who are suffering from osteoporosis. This protects bone from degradation, and helps to counter the progression of the disease.

Denosumab was approved by the EMA for use in postmenopausal women with osteoporosis at increased risk for fracture at the dose of 60 mg sc every 6 months (Prolia®), and for the prevention of skeletal-related events in patients with bone metastasis from solid tumors at the dose of 120 mg every 4 weeks (XGEVA®).
More recently, denosumab was shown to retard the progression of structural lesions in rheumatoid arthritis, an unapproved indication for the drug.[33,34] Its dosing and safety profile depended on the different medical conditions in which the drug was used. Patients with osteoporosis and rheumatoid arthritis received 60 mg and up to 180 mg injected SC, every 6 months, respectively.

Experience from clinical studies indicates that side effects depend on the dosage.

According to Prolia® Summary of Product Characteristics (SmPC) [36], pain in extremities and musculoskeletal pain (including back pain and joint pain) were among the most common adverse reactions.
In patients treated for osteoporosis a rare unwanted effect included low calcium levels, especially when in case of an impaired kidney function. Patients must therefore be adequately supplemented with calcium and vitamin D levels before starting and during denosumab therapy. In the postmarketing setting, rare cases of severe symptomatic hypocalcaemia have been reported. Clinical monitoring of calcium level is recommended before each dose and, in patients predisposed to hypocalcaemia, within two weeks after the initial dose.
There have been rare cases of atypical femoral fracture reported in association with Prolia.
Infections of the urinary and respiratory tracts were reported as well as cellulitis, ear infection and diverticulitis. The SmPC includes a Warning Statement regarding skin infections (predominantly cellulitis) leading to hospitalization. It has been proposed that this increase in infections under denosumab treatment might be connected to the role of RANKL in the immune system.
Cataracts, constipation, skin rashes and eczema were also seen.
Osteonecrosis of the jaw (ONJ) was reported rarely in Prolia osteoporosis clinical development program. Primarily, at the high dosages used in patients with bone metastases, similarly to bisphosphonates, denosumab appeared to be implicated in increasing the risk of osteonecrosis of the jaw (ONJ) especially following extraction of teeth or oral surgical procedures.

In the post-marketing setting, rare events of drug-related hypersensitivity, including rash, urticaria, facial swelling, erythema, and anaphylactic reactions have been reported.

In the FREEDOM extension study[37,38], with up to 8 years of denosumab 60 mg Q6M exposure, the incidence rates of adverse events did not increase over time.

Denosumab safety data were reported in RA phase 2 studies[33,34]. The safety profile appears to be consistent with that in patients with postmenopausal osteoporosis. Denosumab did not have an effect on RA disease activity, as measured by the ACR response criteria, the DAS28 scores, and the occurrence of RA flares.

1.3 Rationale for study design

In RA, the initial changes are seen in the synovium where inflammatory lymphomyeloid cells massively produce TNF, and secondarily, IL-1 and RANKL. These two cytokines are responsible for the invasion of the adjacent cartilage and bone by the inflamed and proliferative synovial pannus.

In erosive IP joint OA, the osteolytic changes in subchondral bone occur before or concurrently with resorption of cartilage. The primary drivers of the cartilage damage thus are these osteolytic processes in the subchondral bone area and the collapse of the subchondral bone plate. RANKL is the cytokine primarily responsible for this osteolytic (osteoclast) activity.

The enhanced osteoclast activity and tissue remodeling initially seen in arthritic IP joint bone is clearly illustrated in figure 1.

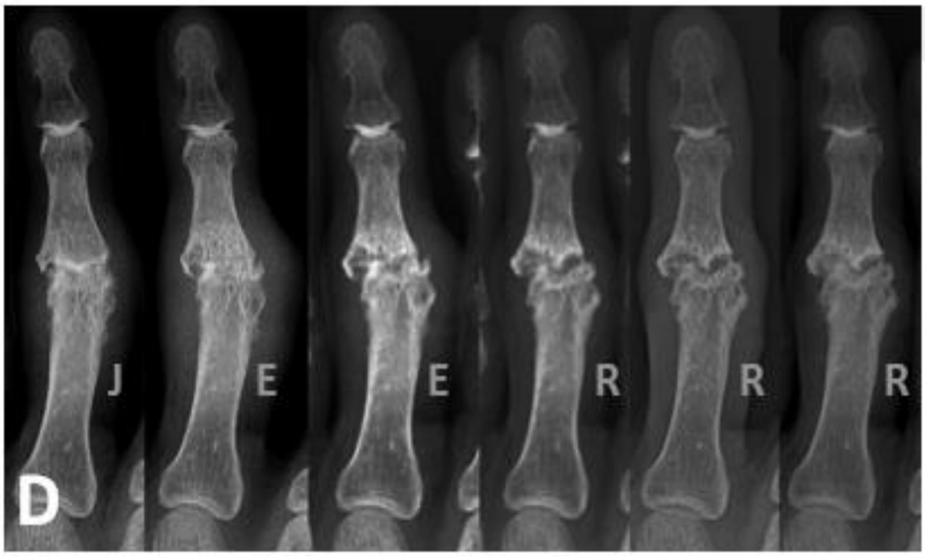

Figure 1: radiographic progression of a proximal IP joint from ‘J’ phase with loss of joint space to the ‘E’ phase with osteolytic activity in the subchondral bone area, and final remodeling of the destroyed tissues (R). Radiographs were taken with 6-months interval.

The effect of TNF alpha inhibitors on disease progression, previously seen in erosive IP joint OA[24], was an indirect effect on osteoclast activation. Obviously, this effect would be larger by directly inhibiting osteoclasts with Denosumab. Once the erosive process is blocked with

Denosumab, subchondral bone remodeling will be inhibited and one should see preservation of joint structure.

A proof-of-concept study is proposed herein to test the ability of repeated administration of denosumab to control the structural damage– and thus to maintain hand function - in erosive hand OA. These tests will be conducted compared to placebo during a first placebo controlled double-blind phase but also in a second open-label phase in which all subjects will receive denosumab. The 2 main factors that support conducting this second open-label phase are the following:
- This would enable the Long-term outcome assessment with the cumulative exposure over time; more substantial effect would be expected.
- The open label with help supporting patients' engagement in a placebo trial where no disease modifying drugs exist.

The adequate dose of denosumab should completely inhibit the erosive process in order to fully test the hypothesis. In the phase 2 RA studies [33,34,], the higher dose or shorter interval dosing regimen showed an earlier or a trend to more inhibition of bone destruction respectively. Considering further the well-established safety profile for denosumab at high doses, a higher frequency for denosumab 60 mg is proposed: denosumab 60 mg sc every 3 months.

1.4 Hypotheses

The main hypothesis is that the repeated administration of denosumab 60 mg every 3 months in erosive hand OA can inhibit structural progression of already affected joints and prevent occurrence of newly affected joints.
As it has been shown that denosumab, reduces structural damage in RA while having no effect on clinical symptoms [34], no clinical benefit is expected within the one-year period of this study. So, the effects of denosumab on the clinical manifestations of the disease will only be part of an exploratory study.

2. Study Objectives and Endpoints

The objective of this proof of concept study is to investigate the efficacy of denosumab 60 mg sc every 12 weeks for 48 weeks as a therapeutic intervention in erosive IP joint OA. In general, the expected outcome of this study would be the control of the structural damage.

Changes in the architecture of the joint will be assessed by the GUSS™. This score system allows an overall score to be calculated for an affected IP joint over time. The overall score is the sum of scores obtained for 3 compartments of the IP finger joint: the synovial space (articular cartilage), the subchondral bone plates and the subchondral bone area at each side of the synovial space. Overall scores, as well as scores for each individual compartment can be taken into consideration. Examples of the calculated scores for 2 different IP joints are given in appendix 1.

The **primary objective** is to assess the effect of denosumab on the reduction of radiographic erosive progression using GUSS™ (Ghent University Score System).
The **primary endpoints of this objective** is the change in the negative evolution in GUSS™ scores in the target IP joints from baseline to week 24.

Other endpoints are the changes in the negative evolution of GUSS™ scores in the target IP joints from week 24 to week 48 and from baseline to week 48.

The **secondary objective** is to evaluate a reduction in radiographic erosive progression as defined by diminishing the appearance of new erosive IP finger joints.
This will be assessed by 2 endpoints:

1. the number of patients that develop new erosive IP joints (‘S/J’ to ‘E’ phases) at 48 weeks.
2. the number of ‘S/J’ IP joints that develop ‘E’ phases at 48 weeks.

Radiological score systems are given in appendix 1.

The **exploratory** objective is to assess if denosumab provides clinical benefits (improvement of pain and functional limitations) compared to placebo. We will also evaluate the impact on ultrasonography and DEXA.
The endpoints of this objective are:

1. Changes in clinical and patient recorded outcome measures from baseline (day 1) to week 48 after administration of denosumab compared to placebo. The following outcome measures will be recorded: AUSCAN (AUStralian CANadian Osteoarthritis Hand Index), FIHOA (Functional Index of Hand Osteoarthritis), Pain on VAS scale, consumption of analgesics (paracetamol)/NSAIDs to be recorded by each patient on a diary, tenderness upon pressure, diameter of selected target joints, and grip strength of both hands.
2. Changes in sonographic inflammatory signals at week 12 and 48 compared to screening and baseline. Inflammatory changes will be assessed by measuring the amount of effusion and Power Doppler signal (scoring on a semi-quantitative scale).
3. Effect of denosumab on bone mass densitometry score in this group of patients compared to placebo from baseline to week 48. Changes from baseline (day 1) in T-score at lumbar spine and hip measured by bone densitometry at week 48 after administration of denosumab compared to placebo.

Other exploratory endpoints are to describe the above radiographic progression parameters at the end of the open-label phase.

Safety-objective
The safety profile of denosumab 60 mg (Prolia®) every 6 months in postmenopausal women with osteoporosis at increased risk of fracture is well established (Prolia SmPC). This study will assess the safety of the administration of denosumab 60 mg every 3 months in the population of patients with erosive OA. Safety evaluations will be made by recording the incidence of AE/SAE (see also paragraph 8).

3. Experimental Plan

3.1 Study design and schematic

This is a randomized, double blind, placebo-controlled, one-site proof of concept study to investigate the effect of denosumab 60 mg every 12 weeks on the radiological evolution of erosive OA of the digital joints.
Two groups of 50 patients each will be enrolled in the study with a total treatment duration of 24 months (96 weeks): 48 weeks double-blind placebo controlled phase (denosumab (60 mg

sc every 12 weeks or placebo) followed by a 48-weeks open–label phase in which all subjects will receive denosumab 60 mg every 12 weeks in an “Open Label Design” type study.

Study schematic

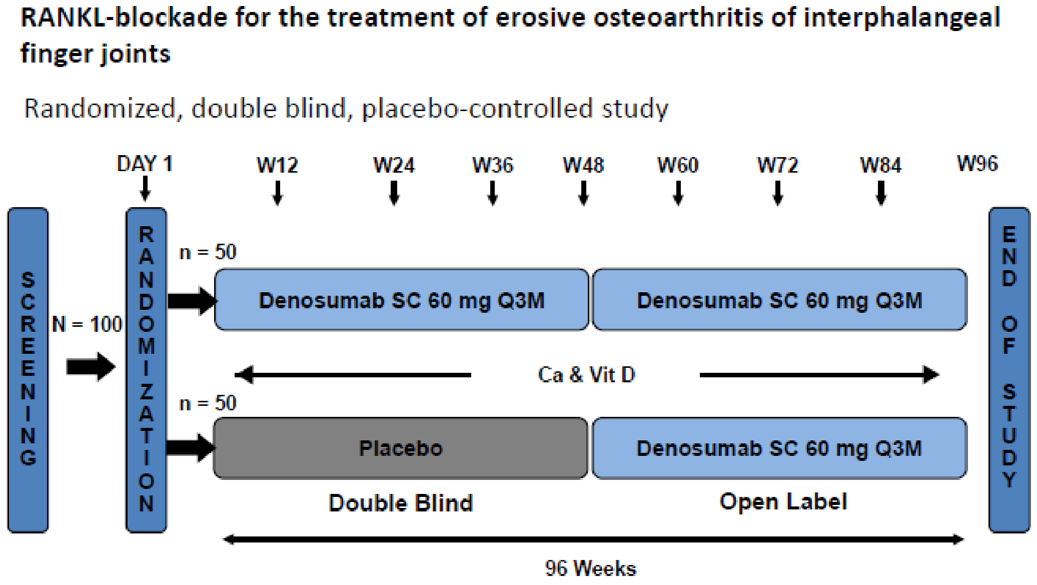

3.2 Number of sites

The study will be conducted in one site – the Ghent site in Belgium.

3.3 Number of subjects

A total of 100 subjects will be recruited in this study with an enrolment period of 18 months.

3.4 Estimated study duration

The total treatment duration per subject is 24 months (96 weeks). The expected total trial duration defined as the time from first patient first visit to last patient last visit is 42 months.

4. Subject Eligibility

4.1 Inclusion criteria

A subject will be eligible for study participation if he/she meets the following criteria:

- Males and females ≥ 30 years of age.
- Subjects with hand OA having suffered from transient inflammatory attacks of the interphalangeal finger joints characteristic for what has been termed ‘inflammatory’ or ‘erosive’ hand OA.
- Subjects with hand OA showing inflammatory signs, either clinically or ultrasonographically, of the interphalangeal finger joints.
- Subjects with hand OA in which at least 1 interphalangeal finger joint has the typical appearance on the X-rays of a ‘J’ or ‘E’ phase joint as defined by the criteria mentioned above.
- Subjects with hand OA where at least 1 interphalangeal finger joint in the ‘J’ or ‘E’ phase presents a palpable swelling.
- Able and willing to give written informed consent and to comply with the requirements of the study protocol.

4.2 Exclusion criteria

A subject will be excluded from the study if he/she meets any of the following criteria:

- Patients with known hypersensitivities to mammalian-derived drug preparations.
- Patients with clinically significant hypersensitivity to any of the components of Prolia.
- Current and/or Prior treatment with any investigational agent within 90 days, or five half-lives of the product, whichever is longer.
- Previous administration of denosumab from clinical trials or others (e.g. commercial use).
- Vitamin D deficiency [25(OH) vitamin D level < 20 ng/mL (< 49.9 nmol/L)]. Possibility of replenishment and re-screening.
- Subjects with current hypo- or hypercalcemia (normal serum calcium levels: 8.5-10.5 mg/dl or 2.12-2.62 mmol/L).
- Patients currently under bisphosphonate (BP) treatment or any use of oral BPs within 12 months of study enrollment or intravenous BPs or strontium ranelate within 5 years of study enrollment
- Prior use of any chondroprotective drug within 90 days e.g. chondroitin sulfate, glucosamine, avocado-soybean unsaponifiables, tetracyclins, corticosteroids.
- Prior use of any immunomodulating drug with possible effects on proinflammatory cytokine metabolism within 90 days a.o. corticosteroids, methotrexate, sulfasalazine, leflunomide, D-Penicillin, anti-malarials, cytotoxic drugs, TNF blocking agents.
- History of drug or alcohol abuse in the last year.
- Patients suffering from chronic inflammatory rheumatic disease (e.g. rheumatoid arthritis, spondylarthropathy, psoriatic arthritis, gout, chondrocalcinosis or other auto-immune diseases, e.g. systemic lupus erythematosus).
- History of cancer or lymphoproliferative disease other than a successfully and completely treated squamous cell or basal cell carcinoma of the skin or cervical dysplasia, with no recurrence within the last two years.
- History of any Solid Organ or Bone Marrow Transplant.
  - Comorbidities: significant renal function impairment (glomerular filtration < 30 ml/min/1.73m^2 or <50% of normal value), uncontrolled diabetes, unstable ischemic

heart disease, congestive heart failure (NYHA III, IV), uncontrolled hypo or hyperparathyroidism, active inflammatory bowel disease, malabsorption, liver failure or chronic hepatic disease (serum AST/ALT levels 3 times above normal), recent stroke (within three months), chronic leg ulcer and any other condition (*e.g,*. indwelling urinary catheter) which, in the opinion of the investigator, would put the subject at risk by participation in the protocol.

- Subject has any kind of disorder that compromises the ability of the subject to give written informed consent and/or to comply with study procedures .
- Patient who is pregnant or planning pregnancy; if the female subject is of child-bearing age, she must use a valid mean of contraception during the study and for 9 months after last dose of study medication. For males with a partner of childbearing potential: subject refuses to use 1 effective methods of contraception for the duration of the study and for 10 months after the last dose of study medication.
- Female subjects who are breast-feeding.
- History of osteonecrosis of the jaw, and/or recent (within 3 months) tooth extraction or other unhealed dental surgery; or planned invasive dental work during the study.

5. Treatment and Study Procedures

5.1 Investigational product (see also paragraph 1.2)
The study drug used in this clinical trial is denosumab 60 mg subcutaneously every 3 months. It will be provided as sterile, solution for injection in 1 ml pre-filled syringes containing denosumab 60mg/ ml or placebo. Placebo for Denosumab will be presented in identical containers and stored/packaged the same as drug product denosumab. Denosumab prefilled syringe placebo product is supplied in a prefilled syringe as a sterile, single use, preservative free solution for subcutaneous injection. Each prefilled syringe contains 1 mL deliverable volume of buffer consisting of 10 mM sodium acetate, 5% (w/v) sorbitol, 0.01% (w/v) polysorbate 20, at a pH of 5.2. The IP is packed with 1 PFS per box. Both Denosumab and Placebo are manufactured by Amgen Inc, United States and released in the EU by Amgen Breda, Netherlands. Amgen will provide batch release certificates that will be made available with each shipment of the drug. Amgen will provide GMP certification and investigational medicinal product dossiers directly to the Belgian Agency in the regulatory submission by Amgen for this ISS. The injections will be given at the study site. Instructions for the drug handling, packaging and storage are provided in details below. Briefly, the drug will be given under the skin of the thigh, abdomen or upper arm. The clinical supplies should be stored in the refrigerator at 2-8°C. Do not freeze. Do not shake excessively. The clinical supplies must be protected from light by storing in the outer carton.

Patients who completed the 1-year interventional study will have the opportunity to enter a second 1-year open-label extension (OLE) study with Denosumab (60 mg every 12 weeks, SC). The 1-year radiographic progression of their IP finger joints will be monitored after 6 and 12 months of treatment in the OLE.

Drug Handling:
"Denosumab is supplied as a sterile, colorless to slightly yellow, preservative-free solution for injection in a 1mL prefilled syringe (PFS). The formulation of IP is 60 mg/mL denosumab per

mL, formulated with10 mM Sodium Acetate, 5% Sorbitol, 0.01% Polysorbate, to a pH of 5.2. Each PFS of IP is intended for single use only. The IP is packed with 1 PFS per box. Placebo for denosumab will be presented in identical containers and stored/packaged in the same way as drug product denosumab.

The IP is shipped by air courier maintained at 2°C to 8°C in a qualified shipper suitable for biological substance shipments. IP in a PFS will arrive in a secondary packaging container and should be immediately placed in a refrigerator maintained at 2°C to 8°C in a secured location until planned use. The set point for the refrigerator should be at 5°C.
IP must be properly labelled and dispensed in accordance with current ICH GCP and local/regional requirements prior to dispensing for administration.
Before preparation check that IP:

- is visually intact and suitable for use
- is not expired
- has not been subjected to any potential temperature excursion
- label of the box and vial is correct

Prior to administration, IP may be removed from the refrigerator and brought to room temperature (up to 25°C) in the original container. This generally takes 15 to 30 minutes. Do not warm IP in any other way. Once removed from the refrigerator, IP must not be exposed to temperatures above 25°C/77°F and must be used within 24 hours. If not used within this time duration, IP must be discarded. Do not freeze IP. Protect IP from light and heat. Avoid vigorous shaking. Preparation of the clinical supplies should be performed using aseptic techniques and under sterile conditions.

Administration of IP must be performed as the last procedure after all the other study procedures have been completed for the visit. All SC injections must be administered by authorized site personnel. All subjects will receive 1 SC injection at each dosing visit (of either 60mg/ml Denosumab or Placebo) administered in the subject's upper arm, upper thigh or abdomen by a trained and qualified staff member. The injection should not be administered in the same arm from which blood is drawn."

5.2 Reporting requirements for investigational product complaints:
The following could be considered potential product complaints that need to be reported to Amgen. The Investigator will use a Product Complaint Form as provided by Amgen to report any complaint. Should any such concerns or irregularities occur, the IP will not be used until Amgen confirms that it is permissible to use. Examples of Product Complaints:
• Packaging: for example, broken container or cracked container
• Devices: issues with delivery of IP by device
• Usage: for example, subject or healthcare provider cannot appropriately use the product
• Labeling: for example, missing labels, illegible labels, incorrect labels, and/or suspect labels
• Change in IP appearance: for example color change or presence of foreign material
• Unexpected quantity in bottle: for example number of tablets or amount of fluid
• Evidence of tampering or stolen material

5.3 Concomitant therapy
All patients will have a daily calcium (1000 mg) and vitamin D (880 IU) supplementation. Subjects who are current or previous users of denosumab will be excluded at screening (see exclusion criteria).

Concomitant medication: NSAIDs and analgesics are allowed throughout during the study, but the dosages are kept constant during the first 12 weeks. Patients will keep records of their daily use of symptom modifying drugs.

5.4 Study procedures and schedule of assessments

A screening visit will include a clinical assessment, a hand radiograph and the laboratory investigations required. These will comprise a calcium and vitamin D status, peripheral blood cell count (PBC), serum chemistry glucose levels, liver (ALT, AST, alkaline phosphatase) and kidney function (serum ureum, serum creatinine, GFR) tests, Bone turnover markers (BTM) and, if appropriate, a pregnancy test.
An electrocardiogram (ECG) and an ultrasound (US) exam of the IP joints are part of the screening program.

Patients will be evaluated for risk factors for ONJ before starting treatment. A dental examination with appropriate preventive dentistry is recommended prior to treatment with Prolia in patients with concomitant risk factors.

The maximum window allowed between the screening visit and the baseline visit is of 3 weeks.

Upon selection, patients will be included in the study during **the baseline visit**, which will include a clinical examination and an ultrasound (US) exam of the IP joints. Magnetic resonance imaging (MRI[39]) of the hand is optional. Study products (denosumab/placebo) will then be administered on-site by the investigator/study nurse. Calcium and vit D supplementation will be installed. Dual energy X-ray absorptiometry (DXA).

Schedule of assessments are provided in detail as Appendix 2. Clinical assessment is the standard practice and will be detailed in the CRF and the SAP. Safety assessment is clarified in the safety paragraph.

At week 6: a clinical/safety evaluation is planned.
At week 12: clinical/safety assessment, PBC and serum chemistry, serum calcium levels and BTM, US. MRI of the hand is optional. Study products (denosumab/placebo) to be administered on-site by the investigator/study nurse.
At week 24: clinical/safety assessment, serum calcium levels, hand radiographs.
Study products (denosumab/placebo) to be administered on-site by the investigator/study nurse.
At week 36: clinical/safety assessment, serum calcium levels. Study products (denosumab/placebo) to be administered.
W36 is the timing for the last IP dose in the blinded period.
At week 48: clinical/safety assessment, US, hand radiographs. Serum calcium levels, PBC and serum chemistry (glucose levels, liver and kidney function tests, and BTM. Study products (denosumab/placebo) to be administered. DXA is optional.

The visit at week 48 is the first visit of the Open Label Extension (OLE) program, which will encompass clinical/ safety exams, laboratory tests and hand radiographs as indicated in the

table. The clinical monitoring of serum calcium during the OLE phase will follow the same schedule as in the placebo controlled phase.
All patients will receive a denosumab injection at W48 after the above assessment. This would be the first denosumab dose administered in the open label phase.

Safety: Patients will be able to report any unwanted effect during the regular visits and through telephone contact at any time in between these visits. Clinical examination is part of this safety assessment. Templates for AE/SAE recording created by the Investigators will be used.

As unwanted effects – other than these reported in previous Prolia osteoporosis programs - are not expected, the collection of other laboratory safety data beyond week 12 during the randomized treatment phase is not arranged.

A negative pregnancy test will be an entry requirement in female premenopausal patients. Premenopausal patients at risk to become pregnant will be excluded if no valid anti-conceptive method is used. In practice, premenopausal women will be an absolute minority in this study population. During the study and during the OLE phase, pregnancy tests will be done before each injection of denosumab in these subjects.

6. Statistical and Analytical Plans

6.1 Efficacy analysis

Complete and specific details of the final statistical analysis will be described and fully documented in the Statisticap Analysis Plan (SAP). The SAP will be finalized prior to the database lock. The analysis will be performed using the statistical software package IBM SPSS .

Demographic and baseline characteristics will be summarized. The number of observations, mean, standard deviation, median, minimum and maximum will be summarized for continuous variables. Discrete variables will be summarized by counts and percentages.

The primary efficacy variables will be the changes from baseline to week 24 in radiographic outcome measures, more specifically changes in GUSS. The primary efficacy comparisons will be between the denosumab treatment group and the placebo treatment group using GEE modelling with treatment as factors and baseline radiographic scores as a covariate.
Additional endpoints will be assessed because several assumptions are made in this pilot study that are derived from a previous clinical study with a TNF-α blocking agent. The kinetics of TNF inhibitors might be different from the kinetics of denosumab on the bone level because of the different mode of action. Therefore it is not possible to predict if a similar rapid response on GUSS™ scores will be observed. Since the whole study is a proof-of-concept and to guarantee that a later response will not be missed, the study period needs to be extended to 48 weeks and the GUSS changes between week 24 and week 48, as well as GUSS changes between baseline and week 48 will be assessed.
Other analyses of radiographic measures will be the number of patients that develop new erosive joints and the number of patients in which erosive joints start the process of remodeling between baseline and 48 weeks. From previous studies it is known that the anatomical phase scoring system is not a sensitive on short term as GUSS.

Exploratory efficacy endpoints including change in Total AUSCAN score and individual subdomain (pain, physical function and stiffness) scores from baseline, change in FIHOA scores from baseline, change in pain scales (VAS pain) from baseline, change in consumption of analgesics (paracetamol)/NSAIDs, changes in number of painful and tender joints from baseline will be analyzed similarly at week 48. Other exploratory endpoints, including the change in number of joints with effusion and/or Power Doppler signal by ultrasound, the change in HOAMRIS scores and the changes in bone densitometry measures from baseline will be analyzed. Additional details will be provided in the SAP.

Primary and exploratory analyses will be repeated on subgroups defined by presence of soft tissue swelling at baseline. Details of analyses of efficacy endpoints at different time points as well as subgroups of interest will be given in the SAP.

The primary and exploratory efficacy variables will be analyzed on the intent-to-treat (ITT) population, defined as all subjects who were randomized.To evaluate the impact of major protocol violations on the results of the study, additional analyses of the primary efficacy analysis may be conducted on the per protocol population, which consists of all ITT subjects who completed the study and are not major protocol violators. The safety population consists of all subjects who received at least one dose of double-blind study medication.

In general, mean change analyses to compare the denosumab and placebo treatment group will be performed using GEE modelling with treatment group as factor and correction for baseline radiographic damage. Correction will be made for possible dependency between joints in the same patient by using an exchangeable matrix. Categorical data will be summarized using frequencies and percentages. Continuous data will be summarized with the number of non-missing observations by mean, standard deviation, median, maximum, and minimum values. In addition to the analyses based on observed data, analysis with imputed missing data will be conducted for selected efficacy variables. The details of such sensitivity analyses will be provided in the SAP. All statistical tests will be conducted at $\alpha = 0.05$ level (two-sided), unless otherwise stated. The last evaluation prior to the first study drug will be used as baseline for all analyses.

6.2 Safety analysis

Safety analyses will be carried out using the safety population, which includes all subjects who received at least one dose of study drug. Treatment-emergent AEs and SAEs will be summarized and reported. The number and percentage of subjects experiencing adverse events will be provided by system organ class and Medical Dictionary for Drug Regulatory Activities (MedDRA) preferred term. In addition, summary of AEs by severity and relationship to study drug will be presented. Serious, severe AEs, or AEs that lead to premature study discontinuation will be listed and described in detail. Mean change in vital signs and laboratory variables at each visit will be summarized for all treated subjects, and compared between treatment groups using one way Analysis of Variance (ANOVA).

6.3 Determination of Sample size

From a placebo controlled trial with adalimumab, we learned that, the risk that an individual IP joint evolves from J/S phase to the E phase is 2-3% per year. This risk increases to 15% for joints with a clinical effusion and to 25% for a painful joint with effusion. Adalimumab therapy reduced this risk for these inflammatory joints from 25% to 3% .

From these data 50 patients in each arm are needed to demonstrate a similar effect of denosumab with a power of 80%.

This power analysis took into account the following assumptions:

1) denosumab has a similar effect as adalimumab
2) a mean of minimal 1 inflamed joint (effusion and painful) per patient at baseline and in case of inclusion of patients with non-inflammatory joints, a within patient independent risk to evolve from J/S to E phase.
3) 5% drop-out
4) The proposed study involves two treatment arms. The level of significance (α) is 0.05.
5) a similar background risk for evolution from J/S to E phase.

Considering the semi-quantitative outcome measure, GUSS, a second power analysis was performed. Several assumptions were made, based on data from a previous study (Verbruggen G et al. ARD 2012;71(6):891-8). Power calculation was performed based on the estimated difference in the semi-quantitative outcome measure, GUSS ™ over time. This outcome measure is selected to detect the radiographic progression in the selected joints after treatment. The following assumptions were made:

- the natural progression (mean change) that can be expected over a period of 6 months is + 24 units (data from the placebo treated group), the mean difference in GUSS™ change between the placebo and adalimumab treated group after 6 months was 25 units. This was considered as clinically significant since
- the smallest detectable difference of GUSS™ was calculated as 40 units (Verbruggen G et al. ARD 2010;69(5):862-7) and improved to 10 units after intensive training.
- the standard deviation of the mean change in GUSS™ is 29,
- based on the above data, a total change of at least (24+ 25) 49 units in GUSS™ in the treatment group is considered to be a clinical relevant effect from a treatment.

The proposed study involves two treatment arms. The level of significance (α) is 0.05. From previous studies performed at our department, an drop out rate of 5% can be expected.
A sample size of 25 patients in each treatment arm will have 80% power to detect a difference in mean change GUSS™ of 25 units between the placebo and treated group, assuming that the standard deviation is 29 using a t-test with a two-sided 0.05 level of significance.
Taking into account a drop out rate of 5%, a total of 27 patients (25/ 1 – 0.05) should be included in each arm.
Taken into consideration both outcome measures, a minimum of 50 patients is required in both treatment arms in order to provide sufficient power for the study.

7. Adverse Events/Adverse Event reporting

The investigator will monitor each subject for clinical and laboratory (serum Ca^{++} levels) evidence of adverse events on a routine basis throughout the study. The investigator will assess and record any adverse event in detail on the adverse event DRF including the date and time of onset, description, seriousness severity, time course, duration and outcome, relationship of the adverse event to study drug, an alternate etiology for events not considered "probably related" to study drug, final diagnosis/syndrome (if known) and any action(s) taken. Adverse events, whether in response to a query, observed by study-site personnel, or reported spontaneously by the subject, will be recorded.

All adverse events will be followed to a satisfactory conclusion.

7.1 Definitions

7.1.1. Adverse Event

An **adverse event** is defined as any untoward medical occurrence in a subject or clinical investigation subject administered a pharmaceutical product and which does not necessarily have a causal relationship with this treatment. An adverse event can therefore be any unfavorable and unintended sign (including an abnormal laboratory finding), symptom, or disease temporally associated with the use of a medicinal product, whether or not the event is considered causally related to the use of the product.

Such an event can result from use of the drug as stipulated in the protocol or labeling, as well as from accidental or intentional overdose, drug abuse, or drug withdrawal. Any worsening of a pre-existing condition or illness is considered an adverse event. Laboratory abnormalities and changes in vital signs are considered to be adverse events only if they result in permanent

or temporary discontinuation of treatment with denosumab, necessitate therapeutic medical intervention and/or if the investigator considers them to be adverse events.

An elective surgery/procedure scheduled to occur during a study will not be considered an adverse event. However, if a pre-existing condition deteriorates unexpectedly during the trial (*e.g.*, surgery performed earlier than planned), then the deterioration of the condition for which the elective surgery/procedure is being done will be considered an adverse event.

7.1.2. Serious Adverse Event

If an adverse event meets any of the following criteria, it is to be considered as serious:

**Death of Subject**	An event that results in the death of a subject.
**Life-Threatening**	An event that, in the opinion of the investigator, would have resulted in immediate fatality if medical intervention had not been taken. This does not include an event that would have been fatal if it had occurred in a more severe form.
**Hospitalization**	An event that results in an admission to the hospital for any length of time. This does not include an emergency room visit or admission to an outpatient facility.
**Prolongation of Hospitalization**	An event that occurs while the study subject is hospitalized and prolongs the subject's hospital stay.
**Congenital Anomaly**	An anomaly detected at or after birth, or any anomaly that results in fetal loss.
**Persistent or Significant Disability/Incapacity**	An event that results in a condition that substantially interferes with the activities of daily living of a study subject. Disability is not intended to include experiences of relatively minor medical significance such as headache, nausea, vomiting, diarrhea, influenza, and accidental trauma (*e.g.*, sprained ankle).
**Important Medical Event Requiring Medical or Surgical Intervention to Prevent Serious Outcome**	An important medical event that may not be immediately life-threatening or result in death or hospitalization, but based on medical judgment may jeopardize the subject and may require medical or surgical intervention to prevent any of the outcomes listed above (*i.e.*, death of subject, life-threatening, hospitalization, prolongation of hospitalization, congenital anomaly, or persistent or significant disability/incapacity). Examples of such events include allergic bronchospasm requiring intensive treatment in an emergency room or at home, blood dyscrasias or convulsions that do not result in inpatient hospitalization, or the development of drug dependency or drug abuse.
**Spontaneous Abortion**	Miscarriage experienced by study subject.
**Elective Abortion**	Elective abortion performed on study subject.

7.1.3. Adverse Event Severity

The investigator will use the following definitions to define/rate the severity of each adverse event:

**Mild**	The adverse event is transient and easily tolerated by the subject.
**Moderate**	The adverse event causes the subject discomfort and interrupts the subject's usual activities.
**Severe**	The adverse event causes considerable interference with the subject's usual activities and may be incapacitating or life-threatening.

7.1.4. Relationship to Study Drug

The investigator will use the following definitions to assess the relationship of the adverse event to the use of study drug:

**Probably Related**	An adverse event has a strong temporal relationship to study drug or recurs on re-challenge and another etiology is unlikely or significantly less likely.
**Possibly Related**	An adverse event has a strong temporal relationship to the study drug and an alternative etiology is equally or less likely compared to the potential relationship to study drug.
**Probably Not Related**	An adverse event has little or no temporal relationship to the study drug and/or a more likely alternative etiology exists.
**Not Related**	An adverse event is due to an underlying or concurrent illness or effect of another drug and is not related to the study drug (*e.g.*, has no temporal relationship to study drug or has a much more likely alternative etiology).

7.2. Adverse Event Reporting

Reporting will be consistent with current safety reporting standards. .Adverse events will be reported between the first dose administration of trial medication and the last trial related activity.
All AEs and SAE's will be recorded in the patient's file and in the CRF. All SAE's will be reported as described below.
SAE's occurring within a period of 30 days following the last intake of trial medication will also be handled as such if spontaneously reported to the investigator.

All serious adverse events (SAE) and pregnancies occurring during clinical trials must be reported by the local Principal Investigator within 2 working days after becoming aware of the SAE to:

- The local EC
- Bimetra Clinics of the University Hospital Ghent

This reporting is done by using the appropriate SAE form. For the contact details, see below.

It is the responsibility of the local Principal Investigator to report the local SAE's to the local EC.

In case the investigator decides the SAE is a SUSAR (Suspected Unexpected Serious Adverse Reaction), Bimetra Clinics will report the SUSAR to the Central EC and the CA within the timelines as defined in national legislation.

In case of a life-threatening SUSAR the entire reporting process must be completed within 7 calendar days. In case of a non life-threatening SUSAR the reporting proces must completed within 15 calendar days.

The first report of a serious adverse event may be made by telephone, e-mail or facsimile (FAX).

Contact details of Bimetra Clinics:

e-mail: bimetra.clinics@uzgent.be
tel.: 09/332 05 00
fax: 09/332 05 20

In the event of a serious, unexpected and related adverse event, the investigator will report this to the Amgen Affiliate by faxing the appropriate adverse event form within 24 hours of being made aware of the serious adverse event and simultaneously to Bimetra Clinics who will report the event to the local regulatory agency within the timelines as defined in the national legislation..
Please fax SAE form to [redacted] Pharmacovigilance Manager : Fax number 0800 80877

The investigator must provide the minimal information: i.e. trial number, subject's initials and date of birth, medication code number, period of intake, nature of the adverse event and investigator's attribution.
This report of a serious adverse event by telephone must always be confirmed by a written, more detailed report. For this purpose the appropriate SAE form will be used.
Pregnancies occurring during clinical trials are considered immediately reportable events. They must be reported as soon as possible using the same SAE form. The outcome of the pregnancy must also be reported.

If the subjects are not under 24-hour supervision of the investigator or his/her staff (out-patients, volunteers), they (or their designee, if appropriate) must be provided with a "trial card" indicating the name of the investigational product, the trial number, the investigator's name and a 24-hour emergency contact number.

8. Regulatory Obligations

8.1 Informed Consent

Signed informed consent will be obtained from the subject before any study procedures are undertaken, or before any medications are withheld from the subject in order to participate in this study. Subject may withdraw consent at any time without prejudice. All efforts will be made to continue the patient follow-up until the end of the study. At withdrawal, patients will be treated and assessed according to standard recommendations and as per latest guidance for contraception criteria in female subjects of child-bearing age or partners of childbearing potential (see exclusion criteria section 4.2).

8.2 Independent Ethics Committee/Institutional Review Board

The study will be declared at www.ClinicalTrials.gov and will comply with the principles of the Declaration of Helsinki. A copy of the study protocol will be submitted for approval to the ethical committee of Ghent University Hospital and to the Federal Agency for Medicines and Health Products *(FAGG; federal agentschap voor geneesmidddelen en gezondheidsproducten)*

9. Documentation relating to the clinical trial – Trial Master File

All documents related to the trial, e.g. study protocol, source documents, case report forms, … will be handled, stored and archived according to the EU Commission's Directive 2005/28/EC 63 Chapter 4.[40]

10. Publication Policy

The results of this study will be reported and published at conferences and in peer-reviewed clinical journals. Authorship publications will follow the Uniform Requirement for

Manuscripts Submitted to Biomedical Journals (International Committee of Medical Journal Editors, 2009), which states:
Authorship credit should be based on (1) substantial contributions to conception and design, acquisition of data, or analysis and interpretation of data; (2) drafting the article or revising it critically for important intellectual content; (3) final approval of the version to be published and (4) Agreement to be accountable for all aspects of the work in ensuring that questions related to the accuracy or integrity of any part of the work are appropriately investigated and resolved. Authors should meet conditions 1, 2, 3 and 4.
For further details , see http://www.icmje.org/recommendations/browse/roles-and-responsibilities/defining-the-role-of-authors-and-contributors.html.

11.References

1- Stecher RM, Hauser H : Heberden's nodes. VII. The roentgenological and clinical appearance of degenerartive joint disease of the fingers. AmJ Roentgenol. 59 :326-337,1948
2- Crain DC : Interphalangeal osteoarthritis. Characterized by painful, inflammatory episodes resulting in deformity of the proximal and distal articulations. JAMA. 175: 1049-1053,1961
3- Peter JB, Pearson CM, Marmor L : Erosive arthritis of the hands. Arthritis Rheum. 9: 365-388,1966
4- Ehrlich GE. Osteoarthritis beginning with inflammation. Definitions and correlations. JAMA. 232: 157-159,1975
5- Verbruggen G and Veys EM. Numerical scoring systems for the anatomic evolution of osteoarthritis of the finger joints. Arthritis Rheum. 1996;**39**:308-20.
6- Zhang Y, Niu J, Kelly-Hayes M, Chaisson CE, Aliabadi P, Felson DT. Prevalence of symptomatic hand osteoarthritis and its impact on functional status among the elderly: The Framingham Study. Am J Epidemiol 2002;**156**:S225.
7- Dahaghin S, Bierma-Zeinstra SMA, Reijman M, Pols HAP, Hazes JMW, Koes BW. Prevalence and determinants of one month hand pain and hand related disability in the elderly (Rotterdam study). Ann Rheum Dis 2005;**64**:99-104.
8 Wittoek R, Vander Cruyssen B, Verbruggen G. Predictors of functional impairment and pain in erosive osteoarthritis of the interphalangeal joints: comparison with controlled inflammatory arthritis.Arthritis Rheum.
9. Leeb BF, Sautner J, Andel L, Rintelen B. A scale for assessment and quantification of chronic rheumatoid affections of the hands. Rheumatology 2003; 42: 1173-78.
10- Dillon CF, Hirsch R, Rasch E, Gu Q. Symptomatic hand osteoarthritis in the United States: prevalence and functional impairment estim ates from the third U.S. National Health and Nutrition Examination Survey, 1991-1994. Am J Phys Med Rehabil. 2007;86:12-21.
11- Kellgren JH. Osteoarthrosis in patients and populations. Br Med J. 1961;2:1-6.
12- Lawrence JS, Bremner JM, Biers F. Osteoarthritis. Prevalence in the population and relationship between symptoms and X-Ray changes. Ann Rheum Dis. 1966;25:1-24.
13- Bagge E, Bjelle A, Valkenburg HA, Svanborg A. Prevalence of radiographic osteoarthritis in two elderly European populations. Rheumatology Int. 1992;12:33-8.
14- Mannoni A, Briganti MP, Di Bari M, Ferrucci L, Constanzo S, Serni U, Masotti G, Marchionni N. Epidemiological profile of symptomatic osteoarthritis in older adults: a population based study in Dicomano, Italy. Ann Rheum Dis. 2003 Jun;62:576-8.
15- Cobby M, Cushnaghan J, Creamer P, Dieppe P, Watt I. Erosive osteoarthritis: is it a separate disease entity? Clinical Radiology 1990;**42**:258-63.
16- Cavasin F, Punzi L, Ramonda R, Pianon M, Oliviero F Sfriso P, Todesco S. Prevalence of erosive osteoarthritis of the hand in a population from Venetian area. Rheumatismo 2004;56:46-50.
17- Punzi L, Ramonda R, Sfriso P. Erosive osteoarthritis. Best Pract Res Clin Rheumatol 2004 ;18:739-58.
18- Poole J, Sayer AA, Hardy R, Wadsworth M, Kuh D, Cooper C. Patterns of interphalangeal hand joint involvement of osteoarthritis among men and women: a British cohort study. Arthritis Rheum. 2003 48:3371-6.
19. Kwok WY, Kloppenburg M, Rosendaal FR, van Meurs JB, Hofman A, Bierma-Zeinstra SMA. Erosive hand osteoarthritis: its prevalence and clinical impact in the general population and symptomatic hand osteoarthritis. Ann Rheum Dis 2011;70:1238-42.

20. Haugen IK, Englund M, Aliabadi P, Niu J, Clancy M, Kvien TK, Felson DT. Prevalence, incidence and progression of hand osteoarthritis in the general population: the Framingham Osteoarthritis Study. Ann Rheum Dis 2011;70:1581-6.
21. Verbruggen G, Wittoek R, Vander Cruyssen B et al. Morbid anatomy of 'erosive osteoarthritis' of the interphalangeal finger joints: an optimised scoring system to monitor disease progression in affected joints. Ann Rheum Dis. 2010;69:862-7.
22. Suda T, Takahashi N, Udagawa N et al. Modulation of osteoclast differentiation and function by the new members of the tumor necrosis factor receptor and ligand families. Endocr Rev.1999;20:345-57.
23. Kobayashi K, Takahashi N, Jimi E, et al. Tumor necrosis factor alpha stimulates osteoclast differentiation by a mechanism independent of the ODF/RANKL-RANK interaction. J Exp Med 2000;191:275-86.
24. Komine M, Kukita A, Kukita T et al. Tumor necrosis factor-alpha cooperates with receptor activator of nuclear factor kappaB ligand in generation of osteoclasts in stromal cell-depleted rat bone marrow cell culture. Bone 2001;28:474-83.
25. Wei S, Kitaura H, Zhou P et al. IL-1 mediates TNF-induced osteoclastogenesis. J Clin Invest 2005;115:282-90.
26- Zwerina J, Redlich K, Polzer K et al. TNF-induced structural joint damage is mediated by IL-1. Proc Natl Acad Sci U S A. 2007;104:11742-7.
27. Lefebvre V, Peeters-Joris C, Vaes G. Modulation by interleukin 1 and tumor necrosis factor alpha of production of collagenase, tissue inhibitor of metalloproteinases and collagen types in differentiated and dedifferentiated articular chondrocytes. Biochim Biophys Acta. 1990;1052:366-78.
28. Wittoek R, Carron P, Verbruggen G. Structural and inflammatory sonographic findings in erosive and non-erosive osteoarthritis of the interphalangeal finger joints. Ann Rheum Dis. 2010;69:2173-6.
29. Jans L, De Coninck T, Wittoek R et al. 3 T DCE-MRI assessment of synovitis of the interphalangeal joints in patients with erosive osteoarthritis for treatment response monitoring. Skeletal Radiol. 2013;42:255-60.
30- Bathon JM, Martin RW, Fleischmann RM, Tesser JR, Schiff MH, Keystone EC,
Genovese MC, Chester Wasko M, Moreland LW, Weaver AL, Markenson J, Finck BK. A Comparison of Etanercept and Methotrexate in Patients with Early Rheumatoid Arthritis. NEJM. 2000; 343:1586-93.
31- Lipsky PE, van der Heijde DMFM, St. Clair EW, Furst DE, Breedveld FC, Kalden JR, Smolen JS, Weisman M, Emery P, Feldmann, Gregory R. Harriman GR, Maini RN. Infliximab and Methotrexate in the Treatment of Rheumatoid Arthritis. NEJM. 2000; 343:1594-602.
32. Alten R, Gram H, Joosten LA, van den Berg WB, Sieper J, Wassenberg S, Burmester G, van Riel P, Diaz-Lorente M, Bruin GJ, Woodworth TG, Rordorf C, Batard Y, Wright AM, Jung T. The human anti-IL-1 beta monoclonal antibody ACZ885 is effective in joint inflammation models in mice and in a proof-of-concept study in patients with rheumatoid arthritis. Arthritis Res Ther. 2008;10:R67.
33. Cohen SB, Dore RK, Lane NE, Ory PA, Peterfy CG, Sharp JT, van der Heijde D, Zhou L, Tsuji W, Newmark R; Denosumab Rheumatoid Arthritis Study Group. Denosumab treatment effects on structural damage, bone mineral density, and bone turnover in rheumatoid arthritis: a twelve-month, multicenter, randomized, double-blind, placebo-controlled, phase II clinical trial. Arthritis Rheum. 2008;58:1299-309.

34. Ishiguro N, Tanaka Y, Yamanaka H, Yoneda T, Ohira T, Okubo N, Genant HK, van der Heijde D and Takeuchi T. Consistent Inhibition of Bone Destruction By Denosumab in Important Subgroups of Japanese Patients with Rheumatoid Arthritis. Arthritis Rheumatol 2014; 66:11(Suppl): S831/Presented at ACR meeting 2014
35. Verbruggen G, Wittoek R, Cruyssen BV, Elewaut D. Tumour necrosis factor blockade for the treatment of erosive osteoarthritis of the interphalangeal finger joints: a double blind, randomised trial on structure modification. Ann Rheum Dis. 2012 Jun;71(6):891-8.
36. Prolia SmPC. Sections 4.4 & 4.838.
37. Bone HG, Chapurlat R, Brandi ML, Brown JP, Czerwinski E, Krieg MA, Mellström D, Radominski SC, Reginster JY, Resch H, Ivorra JA, Roux C, Vittinghoff E, Daizadeh NS, , Bradley MN, Franchimont N, Geller ML, Wagman RB, Cummings SR, Papapoulos S. The effect of three or six years of denosumab exposure in women with postmenopausal osteoporosis: results from the FREEDOM extension. Clin Endocrinol Metab. 2013;98:4483-92.
38. Papapoulos S, et al. Eight Years of Denosumab Treatment in Postmenopausal Women with Osteoporosis: Results From the First Five Years of the FREEDOM extension. WCO-IOF-ESCEO Congress 2014. Osteoporis Int 2014; 25 (Suppl 2):S118
39. Haugen IK, Østergaard M, Eshed I, McQueen FM, Bird P, Gandjbakhch F, Foltz V, Genant H, Peterfy C, Lillegraven S, Haavardsholm EA, Bøyesen P, Conaghan PG.3. Iterative development and reliability of the OMERACT hand osteoarthritis MRI scoring system. J Rheumatol. 2014 Feb;41(2):386-91
40. http://eur-lex.europa.eu/LexUriServ/LexUriServ.do?uri=OJ:L:2005:091:0013:0019:en

12.Appendices

Appendix 1. Scoring systems

A. Categorical scoring system was proposed for the progressive radiographic changes in IP finger joint OA. These changes were characterized by complete loss of the joint space preceding or coinciding with the appearance of subchondral cysts eroding the entire subchondral plate. These erosive episodes subsided spontaneously and were followed by processes of repair.[28]

The anatomical phases in the evolution of IP finger joint OA are the following.

Normal ('N') joints: no signs of OA.
Stationary ('S') phase: classical appearance of OA. Small ossification centers and osteophytes are present at the joint margins. They can both increase in size and discrete narrowing of the joint space can occur.
Loss of joint space ('J' phase): after remaining for a variable time in the stationary phase, some joints (almost exclusively PIPs or DIPs) become destroyed. The joint space completely disappears within a relatively short period of time.
Erosive ('E') phase: concurrently with or shortly after the disappearance of the articular cartilage (J phase), the subchondral plate becomes eroded. The appearance is that of a pseudo-enlargement of an irregular joint space. Roentgenograms obtained at yearly intervals showed that changes in phases from 'S' over 'J' to 'E' could occur within one year. This destructive 'J' and 'E' phases are always followed by repair or remodeling.
Remodeling ('R') phase: new irregular sclerotic subchondral plates are formed, and in between these a new joint space becomes visible. Huge osteophytes are formed during this phase. No further evolution is seen in remodeled joints.

B. A quantitative radiographic scoring system, the Ghent University Scoring System, GUSS® [29], is a reliable method to score radiographic change over time in erosive IP OA and detects more progression over a shorter period of time than the classical scoring system. Erosive progression and signs of repair or remodeling are then scored by indicating the proportions of normal subchondral bone, subchondral plate and joint space over time.

The subchondral bone area. The proportions of the subchondral bone area with normal/abnormal-looking bone architecture were assessed in a quadrangle square of which the side equalled the width of the joint space. The joint space was positioned in the centre of this square (figure 2A). In this square, regions where osteolytic activity and remodelling caused a disarrangement of the trabecular pattern, as well as areas where a complete loss of the trabecular structure had occurred, are defined.
Identifiable osteolytic subchondral bone areas are marked on the radiographs and proportions of remaining intact subchondral bone will be calculated, considering the delineated IP joint area being the 100% value.

The subchondral bone plate. In an IP joint that had completely lost its joint space, an existing subchondral plate was defined as a regular radio-opaque linear structure within the position of the original joint space. When the joint space was still identifiable, the subchondral bone plate was identified as a regular linear radio-opaque bone margin flanking the joint space.

Identifiable linear subchondral plate structures were marked on the radiographic images and proportions of remaining subchondral bone plate were computed, considering a twofold joint space width being the 100% value (figure 2B).

The joint space was recognized as a radiotranslucent area bordered with two subchondral plates. Identifiable joint spaces were marked on the radiographic images. Proportions of remaining joint space were estimated as the proportion of the joint width, considering the total joint space width being the 100% value (figure 2B).

Computation of the changes in IP joints in "J", "E" and "E/R" phases. Pictures from the IP joints at three time points in the correct sequence will be read and used by the readers to evaluate the extent of the pathological changes in subchondral bone architecture, and to estimate the presence/absence of both subchondral bone plate and synovial joint space. Proportional changes in these three variables will be recorded. The sum of the three separate scorings constituted the total IP joint score. Equal weight will be attributed to each of the subdomains.

Appendix 2. Overall assessments

	admin dmab/plac	clinical assessm	safety	laboratory						CR hand	US hand	DXA	ECG
				PBC	serum chem	BTM	25OH Vit D	Ca++	preg test*				
SCREENING		X		X	X	X	X	X	X	X	X		X
BASELINE	X	X	X								X	X	
WEEK 6			X										
WEEK 12	X	X	X	X	X	X		X	X		X		
WEEK 24	X	X	X					X	X	X			
WEEK 36	X	X	X					X	X				
WEEK 48	X	X	X	X	X	X		X	X	X	X	X	
WEEK 60	X	X	X					X	X				
WEEK 72	X	X	X			X		X	X	X			
WEEK 84	X	X	X					X	X				
WEEK 96		X	X	X	X	X		X	X	X		X	

* if appropriate

dmab: denosumab; plac: placebo; PBC: peripheral blood cell count; chem: chemistry; BTM: bone turnover markers
preg: pregnancy - sticks to be provided by the rheumatology dept.; CR: conventional radiography; US: ultrasound;
MRI: magnetic resonace imaging; ECG: electrocardiogramDXA: dual energy X-ray absorptiomery

Basic Serum chemistry will include urea, creatinine, ASAT, ALAT, Albumin. Depending on the individual patient, additional parameters may be added.

W36 is the timing for the last IP dose in the blinded period. All patients will receive a denosumab injection at W48 after the assessment. This would be the first denosumab dose administered in the open label phase.

Study Protocol

RANKL-blockade for the treatment of erosive osteoarthritis (OA) of interphalangeal finger joints

Randomized, double blind, placebo-controlled study to evaluate the efficacy of denosumab 60mg sc every 3 months in patients with erosive osteoarthritis of the interphalangeal finger joints

Principal Investigators:
[redacted]

Dept. of Rheumatology – Ghent University Hospital

EudraCT number: 2015-003223-53
Protocolnumber: AGO/2015/008

[redacted]

[redacted]

[redacted]

Protocol Signature Page

Principal/Chief Investigator signature

3.0 - 03 JUNE 2016 [redacted] 25/4/17

I confirm that I have read and understood protocol version ~~xx dated xx January 2015~~. I agree to comply with the study protocol, the principals of GCP, research governance, clinical trial regulations and appropriate reporting requirements.

Signature.. Date...03 JUN 2016.................................

Print name [redacted]..

Protocol synopsis

**Study Type**	**I**nvestigator **S**ponsored **S**tudy
**Funder**	Amgen
**Study Design**	This is a randomized, double blind placebo controlled one-site proof-of-concept study in subjects with erosive osteoarthritis (OA) of interphangeal (IP) finger joints.<br>A total of 100 subjects will be enrolled into the study: 48 weeks placebo controlled double-blind phase with denosumab 60 mg every 12 weeks, followed by a 48-week open-label phase in which all subjects will receive denosumab.
**Investigational Therapy**	Denosumab 60 mg subcutaneous injection every 12 weeks. All subjects will receive Calcium/vit D supplementation.
**Efficacy Objectives**	The **primary objective** is to assess the effect of denosumab on the reduction of radiographic erosive progression using GUSS™ (Ghent University Score System).<br>The **secondary objective** is to assess the effect of denosumab on the reduction of radiographic erosive progression as defined by diminishing the appearance of new erosive IP finger joints.<br>The **exploratory objective** is mainly to assess the effect of denosumab on clinical variables, as well as ultrasonography and DEXA parameters.
**Main Endpoints**	**Primary Endpoint:** The change in the negative evolution of GUSS™ scores in the target IP joints from baseline to week 24.<br>**Other Endpoints:** 1) The change in the negative evolution of GUSS™ scores in the target IP joints from week 24 to week 48 and from baseline to week 48. 2) The number of patients that develop new erosive IP joints ('S/J' to 'E' phase joints) at 48 weeks; 3) The number of 'S/J' IP joints that develop 'E' phases at 48 weeks.
**Hypothesis**	**The main hypothesis** is that the repeated administration of denosumab 60 mg Q3 months can lead to reduce structural damage in erosive hand OA.
**Study Sites**	1 site – the Ghent site
**Subjects**	100 subjects
**Enrolment**	18 months
**Main Eligibility Criteria**	Males and females ≥ 30 years of age, with hand erosive OA:<br>1) having suffered from transient inflammatory attacks of the IP finger joints<br>2) showing at the time of enrolment inflammatory signs and at least one IP finger joint with the typical X-rays appearance of a 'J' or 'E' phase joint

**Study treatment Duration**	96 weeks

Table of Contents

- Protocol Title and Investigators
- Protocol signature page
- Protocol synopsis

1. Background and Rationale
1.1 Disease background
1.2 Denosumab
1.3 Rationale for study design
1.4 Hypotheses
2. Study Objectives and Endpoints
3. Experimental Plan
3.1 Study design and schematic
3.2 Number of sites
3.3 Number of subjects
3.4 Estimated study duration
4. Subject Eligibility
4.1 Inclusion criteria
4.2 Exclusion criteria
5. Treatment and Study Procedures
5.1 Investigational product
5.2 Reporting requirements for investigational product complaints
5.3 Concomitant therapy
5.4 Study procedures and schedule of assessments
6. Statistical and Analytical Plans
6.1 Efficacy analysis
6.2 Safety analysis
6.3 Determination of sample size
7. Adverse Events/Adverse Event reporting
7.1 Definitions
7.1.1. Adverse Event
7.1.2. Serious Adverse Event
7.1.3. Adverse Event Severity
7.1.4. Relationship to Study Drug
7.2. Adverse Event Reporting
8. Regulatory obligations
8.1Informed Consent
8.2 Independent Ethics Committee/Institutional Review Board
9. Documentation relating to the clinical trial- trial master file
10. Publication Policy
10. References
11. Appendices
Appendix 1. Scoring systems
Appendix 2. Overall assessments

1. Background and Rationale

1.1 Disease background

Erosive osteoarthritis (OA) of the interphalangeal (IP) finger joints is considered an inflammatory subset of osteoarthritis of the hand. Its inflammatory clinical presentation and destructive nature are unmistakable. [1,2,3,4,5] The cumulation of destructive changes in the IP joints eventually results in considerable disability.[6,7,8] There are no significant differences in hand function, stiffness and level of pain between patients with hand OA and rheumatoid arthritis. Scores for both patient groups differ significantly from those of healthy controls.[9]
Patients with erosive OA show more functional impairment and significantly more pain compared to patients with controlled inflammatory arthritis affecting the hands. The acquired structural damage of the IP joints due to destructive/reparative phenomena is the largest contributor to functional limitations.[8]
Radiological prevalence of moderate to severe hand OA is estimated to occur in 7.3% (2.65 million) US adults aged 60+ years.[10] Similar data have been reported in European countries.[7,11,12,13,14]

A significant proportion of these patients suffer from the erosive type of hand OA. In a prospective study of 500 consecutive patients attending a rheumatology clinic with symptomatic limb joint OA, 4.8% cases were identified with erosive IP joint OA.[15]

In a survey on the entire health district in the Venetian area, 2.2% out of 640 subjects aged 40+ years had erosive OA of their IP joints.[16] Mainly women in the perimenopauzal age were affected.[17]

Even higher prevalences were seen in a British cohort study[18] on 2.986 people[18]. Numbers in this study were based on clinics and the authors proposed that a proportion of their polyarticular cases were “inflammatory types of OA in association with erosions”. This assumption was based on an earlier study where clinical examination was validated against hand radiography (Egger et al., J Rheumatol 1995;22:1509–13).
Though the proportions of “erosive IP OA” reported here were probably overrated, the prevalence of what is considered to be “erosive IP OA” in this 53 years of age population was twice as high in women (10,6%), compared to men (5,9%).
More recently, these data were confirmed in 2 large population studies where the prevalence of radiographic erosive IP OA in subjects over 55 years of age ranged between 5.0 and 9.9%.[19,20]. The prevalence for men was lower at 3.3%.

These studies showed that erosive type of hand OA occurred predominantly in women.
Haugen IK et al. et al.[20] defined erosive IP OA at a joint level as Kellgren/Lawrence ≥2 plus erosions. The authors reported a prevalence of erosive IP OA in women of 9,9%, 3 times as high as in men (3,3%). In essence, the Kwok W-Y et al. figures[19] agree with the data above.

Moreover, the Haugen IK et al.[20] reported that symptomatic OA was twice as high in women (15,9%), compared to men (8,2%). Symptomatic OA here was defined as Kellgren/Lawrence stage ≥2 plus pain/aching/stiffness.

From these epidemiological studies we can conclude that the incidence of erosive OA of the IP finger joints ranges from five to ten percent particularly in women.

The aggressive destructive nature of the erosive OA is only recognized late in the disease and the radiological image of the "exhausted" final phase mimics a robust OA. Therefore, the disease was hitherto regarded as a form of primary OA - a degenerative joint disease that is caused by biomechanical overload of the joint structures. There is so far no therapy sought or found for the structural changes in the articular tissues occurring during the course of so-called degenerative joint diseases. Thus, no therapeutic measures are available that act on underlying disease mechanisms and therefore slow down or halt the progression of tissue degradation in joints affected by erosive hand OA. The current standard treatment of care in these patients is limited to symptomatic therapy to reduce pain.

There is still lack of agreement concerning the nature and specificity of erosive IP joint OA. Obviously, in erosive IP OA an important bone resorption is noted in the subchondral bone of IP finger joints, this bone resorption is readily visualized on conventional radiographs (Figure 1). The osteolytic 'erosive' lesions result in the collaps of the subchondral plate which supports the overlaying articular cartilage.[5,21] This is compatible with a pathologic osteoclast activity supported by the effects of RANKL (Receptor Activator of Nuclear Factor kappa-β Ligand).[22] RANKL is a key driver of maturation and activation of osteoclasts in bone in health and disease.[22] In pathologic conditions, RANKL can be strongly induced in a variety of cell types including stromal cells under the influence of locally produced proinflammatory cytokines such TNFα [23,24] and IL-1β.[25,26]
At the same time, a resorption of articular cartilage of the affected IP joints is also noted. As a result, the joint space gradually disappears on X-rays. Likely key factors in this process are TNF and IL-1 which both have important catabolic effects on human chondrocytes.[27] Indeed, during the course of the disease inflammatory processes in the synovial membrane of IP finger joints could be visualized.[28,29] Cytokines release thereof will have important catabolic effects on the neighbouring chondrocytes.

Thus, similar as observed in other destructive processes noted in inflammatory rheumatic diseases, the **TNF→ IL-1→ RANKL-pathway** appears to be a key therapeutic target in erosive hand OA.

Blockade of these cytokines has shown to delay ongoing tissue destruction in murine arthritis and in rheumatoid arthritis in human. [30,31,32,33,34]

Recently, TNFα-blockade was shown to retard the progression of joint damage in erosive IP finger joint OA.[35]

Considering the analogies between rheumatoid arthritis and erosive IP OA in the metabolic pathways that mediate tissue destruction, and the lack of any structure modifying treatment option in the latter, a pilot study exploring the effects of Denosumab on ongoing tissue destruction in IP finger joint OA is proposed.

1.2 Denosumab

Denosumab (Amgen), is a fully human monoclonal antibody designed to inhibit RANKL

(RANK Ligand). RANKL binds to RANK, which exists as a cell surface receptor molecule on “pre”-osteoclasts: precursors of osteoclasts.

Binding of RANKL to RANK acts as the primary signal for bone removal in normal physiological bone remodeling and in a number of pathological conditions, e.g. malignant tumors and bone metastasis.

Activation of RANK by RANKL promotes the maturation of pre-osteoclasts into osteoclasts. Denosumab inhibits osteoclasts’ maturation, function and survival by binding to and inhibiting RANKL. This mimics the natural action of osteoprotegerin, an endogenous RANKL inhibitor that presents with decreasing concentrations in patients who are suffering from osteoporosis. This protects bone from degradation, and helps to counter the progression of the disease.

Denosumab was approved by the EMA for use in postmenopausal women with osteoporosis at increased risk for fracture at the dose of 60 mg sc every 6 months (Prolia®), and for the prevention of skeletal-related events in patients with bone metastasis from solid tumors at the dose of 120 mg every 4 weeks (XGEVA®).
More recently, denosumab was shown to retard the progression of structural lesions in rheumatoid arthritis, an unapproved indication for the drug.[33,34] Its dosing and safety profile depended on the different medical conditions in which the drug was used. Patients with osteoporosis and rheumatoid arthritis received 60 mg and up to 180 mg injected SC, every 6 months, respectively.

Experience from clinical studies indicates that side effects depend on the dosage.

According to Prolia® Summary of Product Characteristics (SmPC) [36], pain in extremities and musculoskeletal pain (including back pain and joint pain) were among the most common adverse reactions.
In patients treated for osteoporosis a rare unwanted effect included low calcium levels, especially when in case of an impaired kidney function. Patients must therefore be adequately supplemented with calcium and vitamin D levels before starting and during denosumab therapy. In the postmarketing setting, rare cases of severe symptomatic hypocalcaemia have been reported. Clinical monitoring of calcium level is recommended before each dose and, in patients predisposed to hypocalcaemia, within two weeks after the initial dose.
There have been rare cases of atypical femoral fracture reported in association with Prolia.
Infections of the urinary and respiratory tracts were reported as well as cellulitis, ear infection and diverticulitis. The SmPC includes a Warning Statement regarding skin infections (predominantly cellulitis) leading to hospitalization. It has been proposed that this increase in infections under denosumab treatment might be connected to the role of RANKL in the immune system.
Cataracts, constipation, skin rashes and eczema were also seen.
Osteonecrosis of the jaw (ONJ) was reported rarely in Prolia osteoporosis clinical development program. Primarily, at the high dosages used in patients with bone metastases, similarly to bisphosphonates, denosumab appeared to be implicated in increasing the risk of osteonecrosis of the jaw (ONJ) especially following extraction of teeth or oral surgical procedures.

In the post-marketing setting, rare events of drug-related hypersensitivity, including rash, urticaria, facial swelling, erythema, and anaphylactic reactions have been reported.

In the FREEDOM extension study[37,38], with up to 8 years of denosumab 60 mg Q6M exposure, the incidence rates of adverse events did not increase over time.

Denosumab safety data were reported in RA phase 2 studies[33,34]. The safety profile appears to be consistent with that in patients with postmenopausal osteoporosis. Denosumab did not have an effect on RA disease activity, as measured by the ACR response criteria, the DAS28 scores, and the occurrence of RA flares.

1.3 Rationale for study design

In RA, the initial changes are seen in the synovium where inflammatory lymphomyeloid cells massively produce TNF, and secondarily, IL-1 and RANKL. These two cytokines are responsible for the invasion of the adjacent cartilage and bone by the inflamed and proliferative synovial pannus.

In erosive IP joint OA, the osteolytic changes in subchondral bone occur before or concurrently with resorption of cartilage. The primary drivers of the cartilage damage thus are these osteolytic processes in the subchondral bone area and the collapse of the subchondral bone plate. RANKL is the cytokine primarily responsible for this osteolytic (osteoclast) activity.

The enhanced osteoclast activity and tissue remodeling initially seen in arthritic IP joint bone is clearly illustrated in figure 1.

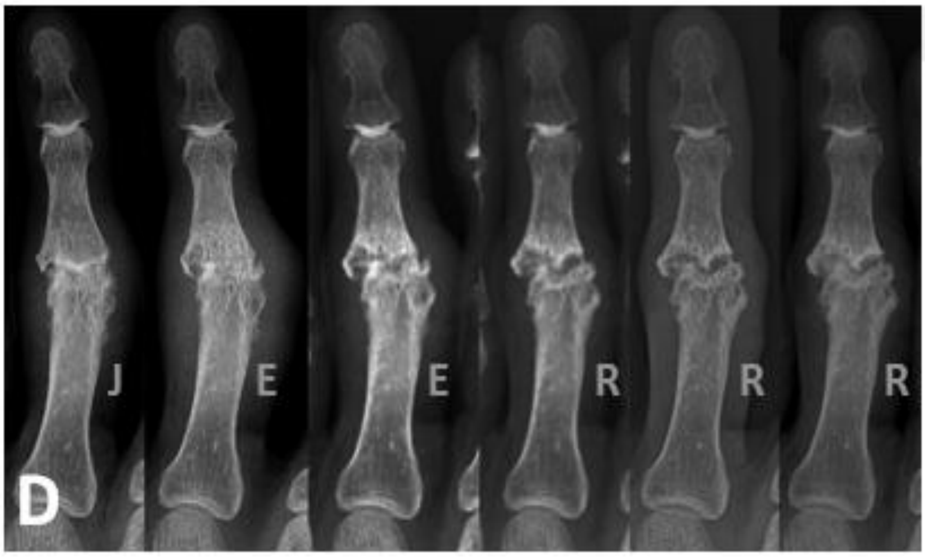

Figure 1: radiographic progression of a proximal IP joint from 'J' phase with loss of joint space to the 'E' phase with osteolytic activity in the subchondral bone area, and final remodeling of the destroyed tissues (R). Radiographs were taken with 6-months interval.

The effect of TNF alpha inhibitors on disease progression, previously seen in erosive IP joint OA[24], was an indirect effect on osteoclast activation. Obviously, this effect would be larger by directly inhibiting osteoclasts with Denosumab. Once the erosive process is blocked with

Denosumab, subchondral bone remodeling will be inhibited and one should see preservation of joint structure.

A proof-of-concept study is proposed herein to test the ability of repeated administration of denosumab to control the structural damage– and thus to maintain hand function - in erosive hand OA. These tests will be conducted compared to placebo during a first placebo controlled double-blind phase but also in a second open-label phase in which all subjects will receive denosumab. The 2 main factors that support conducting this second open-label phase are the following:
- This would enable the Long-term outcome assessment with the cumulative exposure over time; more substantial effect would be expected.
- The open label with help supporting patients' engagement in a placebo trial where no disease modifying drugs exist.

The adequate dose of denosumab should completely inhibit the erosive process in order to fully test the hypothesis. In the phase 2 RA studies [33,34,], the higher dose or shorter interval dosing regimen showed an earlier or a trend to more inhibition of bone destruction respectively. Considering further the well-established safety profile for denosumab at high doses, a higher frequency for denosumab 60 mg is proposed: denosumab 60 mg sc every 3 months.

1.4 Hypotheses

The main hypothesis is that the repeated administration of denosumab 60 mg every 3 months in erosive hand OA can inhibit structural progression of already affected joints and prevent occurrence of newly affected joints.
As it has been shown that denosumab, reduces structural damage in RA while having no effect on clinical symptoms [34], no clinical benefit is expected within the one-year period of this study. So, the effects of denosumab on the clinical manifestations of the disease will only be part of an exploratory study.

2. Study Objectives and Endpoints

The objective of this proof of concept study is to investigate the efficacy of denosumab 60 mg sc every 12 weeks for 48 weeks as a therapeutic intervention in erosive IP joint OA. In general, the expected outcome of this study would be the control of the structural damage.

Changes in the architecture of the joint will be assessed by the GUSS™. This score system allows an overall score to be calculated for an affected IP joint over time. The overall score is the sum of scores obtained for 3 compartments of the IP finger joint: the synovial space (articular cartilage), the subchondral bone plates and the subchondral bone area at each side of the synovial space. Overall scores, as well as scores for each individual compartment can be taken into consideration. Examples of the calculated scores for 2 different IP joints are given in appendix 1.

The **primary objective** is to assess the effect of denosumab on the reduction of radiographic erosive progression using GUSS™ (Ghent University Score System).
The **primary endpoints of this objective** is the change in the negative evolution in GUSS™ scores in the target IP joints from baseline to week 24.

Other endpoints are the changes in the negative evolution of GUSS™ scores in the target IP joints from week 24 to week 48 and from baseline to week 48.

The **secondary objective** is to evaluate a reduction in radiographic erosive progression as defined by diminishing the appearance of new erosive IP finger joints.
This will be assessed by 2 endpoints:
1. the number of patients that develop new erosive IP joints (‘S/J’ to ‘E’ phases) at 48 weeks.
2. the number of ‘S/J’ IP joints that develop ‘E’ phases at 48 weeks.

Radiological score systems are given in appendix 1.

The **exploratory** objective is to assess if denosumab provides clinical benefits (improvement of pain and functional limitations) compared to placebo. We will also evaluate the impact on ultrasonography and DEXA.
The endpoints of this objective are:
1. Changes in clinical and patient recorded outcome measures from baseline (day 1) to week 48 after administration of denosumab compared to placebo. The following outcome measures will be recorded: AUSCAN (AUStralian CANadian Osteoarthritis Hand Index), FIHOA (Functional Index of Hand Osteoarthritis), Pain on VAS scale, consumption of analgesics (paracetamol)/NSAIDs to be recorded by each patient on a diary, tenderness upon pressure, diameter of selected target joints, and grip strength of both hands.
2. Changes in sonographic inflammatory signals at week 12 and 48 compared to baseline. Inflammatory changes will be assessed by measuring the amount of effusion and Power Doppler signal (scoring on a semi-quantitative scale).
3. Effect of denosumab on bone mass densitometry score in this group of patients compared to placebo from baseline to week 48. Changes from baseline (day 1) in T-score at lumbar spine and hip measured by bone densitometry at week 48 after administration of denosumab compared to placebo.

Other exploratory endpoints are to describe the above radiographic progression parameters at the end of the open-label phase.

Safety-objective
The safety profile of denosumab 60 mg (Prolia®) every 6 months in postmenopausal women with osteoporosis at increased risk of fracture is well established (Prolia SmPC). This study will assess the safety of the administration of denosumab 60 mg every 3 months in the population of patients with erosive OA. Safety evaluations will be made by recording the incidence of AE/SAE (see also paragraph 8).

3. Experimental Plan

3.1 Study design and schematic

This is a randomized, double blind, placebo-controlled, one-site proof of concept study to investigate the effect of denosumab 60 mg every 12 weeks on the radiological evolution of erosive OA of the digital joints.
Two groups of 50 patients each will be enrolled in the study with a total treatment duration of 24 months (96 weeks): 48 weeks double-blind placebo controlled phase (denosumab (60 mg

sc every 12 weeks or placebo) followed by a 48-weeks open–label phase in which all subjects will receive denosumab 60 mg every 12 weeks in an "Open Label Design" type study.

Study schematic

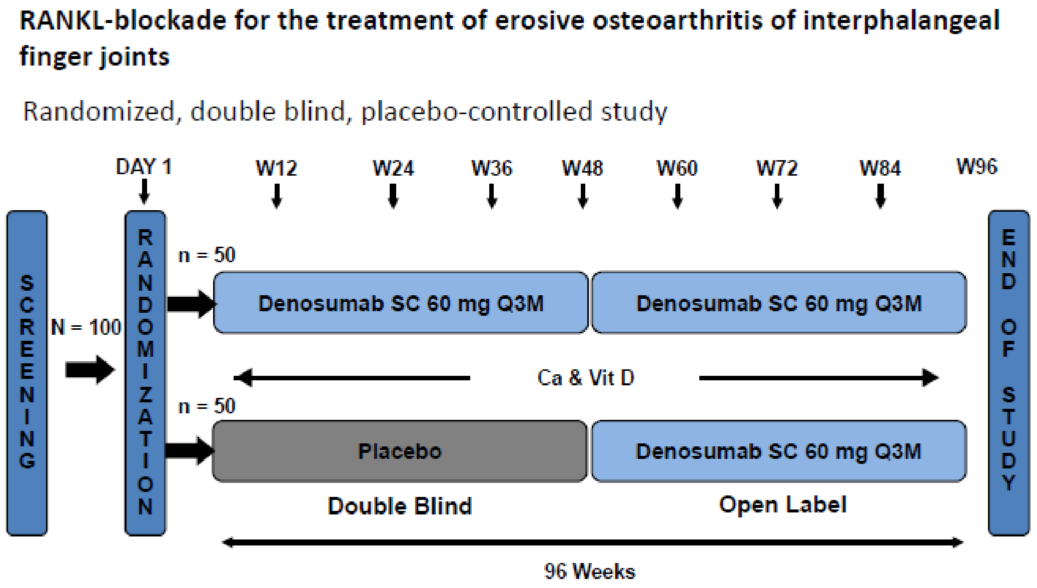

3.2 Number of sites

The study will be conducted in one site – the Ghent site in Belgium.

3.3 Number of subjects

A total of 100 subjects will be recruited in this study with an enrolment period of 18 months.

3.4 Estimated study duration

The total treatment duration per subject is 24 months (96 weeks). The expected total trial duration defined as the time from first patient first visit to last patient last visit is 42 months.

4. Subject Eligibility

4.1 Inclusion criteria

A subject will be eligible for study participation if he/she meets the following criteria:

- Males and females ≥ 30 years of age.
- Subjects with hand OA having suffered from transient inflammatory attacks of the interphalangeal finger joints characteristic for what has been termed ‘inflammatory’ or ‘erosive’ hand OA.
- Subjects with hand OA showing inflammatory signs, either clinically or ultrasonographically, of the interphalangeal finger joints.
- Subjects with hand OA in which at least 1 interphalangeal finger joint has the typical appearance on the X-rays of a ‘J’ or ‘E’ phase joint as defined by the criteria mentioned above.
- Subjects with hand OA where at least 1 interphalangeal finger joint in the ‘J’ or ‘E’ phase presents a palpable swelling.
- Able and willing to give written informed consent and to comply with the requirements of the study protocol.

4.2 Exclusion criteria

A subject will be excluded from the study if he/she meets any of the following criteria:

- Patients with known hypersensitivities to mammalian-derived drug preparations.
- Patients with clinically significant hypersensitivity to any of the components of Prolia.
- Current and/or Prior treatment with any investigational agent within 90 days, or five half-lives of the product, whichever is longer.
- Previous administration of denosumab from clinical trials or others (e.g. commercial use).
- Vitamin D deficiency [25(OH) vitamin D level < 20 ng/mL (< 49.9 nmol/L)]. Possibility of replenishment and re-screening.
- Subjects with current hypo- or hypercalcemia (normal serum calcium levels: 8.5-10.5 mg/dl or 2.12-2.62 mmol/L).
- Patients currently under bisphosphonate (BP) treatment or any use of oral BPs within 12 months of study enrollment or intravenous BPs or strontium ranelate within 5 years of study enrollment
- Prior use of any chondroprotective drug within 90 days e.g. chondroitin sulfate, glucosamine, avocado-soybean unsaponifiables, tetracyclins, corticosteroids (oral, intramuscular, intra-articular or intralesional).
- Prior use of any immunomodulating drug with possible effects on proinflammatory cytokine metabolism within 90 days a.o. corticosteroids (oral, intramuscular, intra-articular or intralesional), methotrexate, sulfasalazine, leflunomide, D-Penicillin, anti-malarials, cytotoxic drugs, TNF blocking agents.
- History of drug or alcohol abuse in the last year.
- Patients suffering from chronic inflammatory rheumatic disease (e.g. rheumatoid arthritis, spondylarthropathy, psoriatic arthritis, gout, chondrocalcinosis or other auto-immune diseases, e.g. systemic lupus erythematosus).
- History of cancer or lymphoproliferative disease within the past five years, other than a successfully and completely treated squamous cell or basal cell carcinoma of the skin or cervical dysplasia, with no recurrence within the last two years.
- History of any Solid Organ or Bone Marrow Transplant.

- Comorbidities: significant renal function impairment (glomerular filtration < 30 ml/min/1.73m^2 or <50% of normal value), uncontrolled diabetes, unstable ischemic heart disease, congestive heart failure (NYHA III, IV), uncontrolled hypo or hyperparathyroidism, active inflammatory bowel disease, malabsorption, liver failure or chronic hepatic disease (serum AST/ALT levels 3 times above normal), recent stroke (within three months), chronic leg ulcer and any other condition (*e.g,*. indwelling urinary catheter) which, in the opinion of the investigator, would put the subject at risk by participation in the protocol.

- Subject has any kind of disorder that compromises the ability of the subject to give written informed consent and/or to comply with study procedures .
- Patient who is pregnant or planning pregnancy; if the female subject is of child-bearing age, she must use a valid mean of contraception during the study and for 9 months after last dose of study medication. For males with a partner of childbearing potential: subject refuses to use 1 effective methods of contraception for the duration of the study and for 10 months after the last dose of study medication.
- Female subjects who are breast-feeding.
- History of osteonecrosis of the jaw, and/or recent (within 3 months) tooth extraction or other unhealed dental surgery; or planned invasive dental work during the study.

5. Treatment and Study Procedures

5.1 Investigational product (see also paragraph 1.2)
The study drug used in this clinical trial is denosumab 60 mg subcutaneously every 3 months. It will be provided as sterile, solution for injection in 1 ml pre-filled syringes containing denosumab 60mg/ ml or placebo. Placebo for Denosumab will be presented in identical containers and stored/packaged the same as drug product denosumab. Denosumab prefilled syringe placebo product is supplied in a prefilled syringe as a sterile, single use, preservative free solution for subcutaneous injection. Each prefilled syringe contains 1 mL deliverable volume of buffer consisting of 10 mM sodium acetate, 5% (w/v) sorbitol, 0.01% (w/v) polysorbate 20, at a pH of 5.2. The IP is packed with 1 PFS per box. Both Denosumab and Placebo are manufactured by Amgen Inc, United States and released in the EU by Amgen Breda, Netherlands. Amgen will provide batch release certificates that will be made available with each shipment of the drug. Amgen will provide GMP certification and investigational medicinal product dossiers directly to the Belgian Agency in the regulatory submission by Amgen for this ISS. The injections will be given at the study site. Instructions for the drug handling, packaging and storage are provided in details below. Briefly, the drug will be given under the skin of the thigh, abdomen or upper arm. The clinical supplies should be stored in the refrigerator at 2-8°C. Do not freeze. Do not shake excessively. The clinical supplies must be protected from light by storing in the outer carton.

Patients who completed the 1-year interventional study will have the opportunity to enter a second 1-year open-label extension (OLE) study with Denosumab (60 mg every 12 weeks, SC). The 1-year radiographic progression of their IP finger joints will be monitored after 6 and 12 months of treatment in the OLE.

Drug Handling:
“Denosumab is supplied as a sterile, colorless to slightly yellow, preservative-free solution for injection in a 1mL prefilled syringe (PFS). The formulation of IP is 60 mg/mL denosumab per mL, formulated with10 mM Sodium Acetate, 5% Sorbitol, 0.01% Polysorbate, to a pH of 5.2. Each PFS of IP is intended for single use only. The IP is packed with 1 PFS per box. Placebo for denosumab will be presented in identical containers and stored/packaged in the same way as drug product denosumab.

The IP is shipped by air courier maintained at 2°C to 8°C in a qualified shipper suitable for biological substance shipments. IP in a PFS will arrive in a secondary packaging container and should be immediately placed in a refrigerator maintained at 2°C to 8°C in a secured location until planned use. The set point for the refrigerator should be at 5°C.
IP must be properly labelled and dispensed in accordance with current ICH GCP and local/regional requirements prior to dispensing for administration.
Before preparation check that IP:

- is visually intact and suitable for use
- is not expired
- has not been subjected to any potential temperature excursion
- label of the box and vial is correct

Prior to administration, IP may be removed from the refrigerator and brought to room temperature (up to 25°C) in the original container. This generally takes 15 to 30 minutes. Do not warm IP in any other way. Once removed from the refrigerator, IP must not be exposed to temperatures above 25°C/77°F and must be used within 24 hours. If not used within this time duration, IP must be discarded. Do not freeze IP. Protect IP from light and heat. Avoid vigorous shaking. Preparation of the clinical supplies should be performed using aseptic techniques and under sterile conditions.

Administration of IP must be performed as the last procedure after all the other study procedures have been completed for the visit. All SC injections must be administered by authorized site personnel. All subjects will receive 1 SC injection at each dosing visit (of either 60mg/ml Denosumab or Placebo) administered in the subject’s upper arm, upper thigh or abdomen by a trained and qualified staff member. The injection should not be administered in the same arm from which blood is drawn.”

5.2 Reporting requirements for investigational product complaints:
The following could be considered potential product complaints that need to be reported to Amgen. The Investigator will use a Product Complaint Form as provided by Amgen to report any complaint. Should any such concerns or irregularities occur, the IP will not be used until Amgen confirms that it is permissible to use. Examples of Product Complaints:
• Packaging: for example, broken container or cracked container
• Devices: issues with delivery of IP by device
• Usage: for example, subject or healthcare provider cannot appropriately use the product
• Labeling: for example, missing labels, illegible labels, incorrect labels, and/or suspect labels
• Change in IP appearance: for example color change or presence of foreign material
• Unexpected quantity in bottle: for example number of tablets or amount of fluid
• Evidence of tampering or stolen material

5.3 Concomitant therapy

All patients will have a daily calcium (1000 mg) and vitamin D (880 IU) supplementation. Subjects who are current or previous users of denosumab will be excluded at screening (see exclusion criteria).
Concomitant medication: NSAIDs and analgesics are allowed throughout during the study, but the dosages are kept constant during the first 12 weeks. Patients will keep records of their daily use of symptom modifying drugs.

5.4 Study procedures and schedule of assessments

A screening visit will include a clinical assessment, a hand radiograph and the laboratory investigations required. These will comprise a calcium and vitamin D status, peripheral blood cell count (PBC), serum chemistry glucose levels, liver (ALT, AST, alkaline phosphatase) and kidney function (serum ureum, serum creatinine, GFR) tests, Bone turnover markers (BTM) and, if appropriate, a pregnancy test.
An electrocardiogram (ECG) and an ultrasound (US) exam of the IP joints are part of the screening program.

Patients will be evaluated for risk factors for ONJ before starting treatment. A dental examination with appropriate preventive dentistry is recommended prior to treatment with Prolia in patients with concomitant risk factors.

The maximum window allowed between the screening visit and the baseline visit is of 3 weeks.

Upon selection, patients will be included in the study during **the baseline visit**, which will include a clinical examination and an ultrasound (US) exam of the IP joints. Magnetic resonance imaging (MRI[39]) of the hand is optional. Study products (denosumab/placebo) will then be administered on-site by the investigator/study nurse. Calcium and vit D supplementation will be installed. Dual energy X-ray absorptiometry (DXA).

Schedule of assessments are provided in detail as Appendix 2. Clinical assessment is the standard practice and will be detailed in the CRF and the SAP. Safety assessment is clarified in the safety paragraph.

At week 6: a clinical/safety evaluation is planned.
At week 12: clinical/safety assessment, PBC and serum chemistry, serum calcium levels and BTM, US. MRI of the hand is optional. Study products (denosumab/placebo) to be administered on-site by the investigator/study nurse.
At week 24: clinical/safety assessment, serum calcium levels, hand radiographs.
Study products (denosumab/placebo) to be administered on-site by the investigator/study nurse.
At week 36: clinical/safety assessment, serum calcium levels. Study products (denosumab/placebo) to be administered.
W36 is the timing for the last IP dose in the blinded period.
At week 48: clinical/safety assessment, US, hand radiographs, DXA. Serum calcium levels, PBC and serum chemistry (glucose levels, liver and kidney function tests, and BTM. Study products (denosumab/placebo) to be administered.

The visit at week 48 is the first visit of the Open Label Extension (OLE) program, which will encompass clinical/ safety exams, laboratory tests and hand radiographs as indicated in the table. The clinical monitoring of serum calcium during the OLE phase will follow the same schedule as in the placebo controlled phase.
All patients will receive a denosumab injection at W48 after the above assessment. This would be the first denosumab dose administered in the open label phase.

Safety: Patients will be able to report any unwanted effect during the regular visits and through telephone contact at any time in between these visits. Clinical examination is part of this safety assessment. Templates for AE/SAE recording created by the Investigators will be used.

As unwanted effects – other than these reported in previous Prolia osteoporosis programs - are not expected, the collection of other laboratory safety data beyond week 12 during the randomized treatment phase is not arranged.

A negative pregnancy test will be an entry requirement in female premenopausal patients. Premenopausal patients at risk to become pregnant will be excluded if no valid anti-conceptive method is used. In practice, premenopausal women will be an absolute minority in this study population. During the study and during the OLE phase, pregnancy tests will be done before each injection of denosumab in these subjects.

6. Statistical and Analytical Plans

6.1 Efficacy analysis

Complete and specific details of the final statistical analysis will be described and fully documented in the Statisticap Analysis Plan (SAP). The SAP will be finalized prior to the database lock. The analysis will be performed using the statistical software package IBM SPSS .

Demographic and baseline characteristics will be summarized. The number of observations, mean, standard deviation, median, minimum and maximum will be summarized for continuous variables. Discrete variables will be summarized by counts and percentages.

The primary efficacy variables will be the changes from baseline to week 24 in radiographic outcome measures, more specifically changes in GUSS. The primary efficacy comparisons will be between the denosumab treatment group and the placebo treatment group using GEE modelling with treatment as factors and baseline radiographic scores as a covariate.
Additional endpoints will be assessed because several assumptions are made in this pilot study that are derived from a previous clinical study with a TNF-α blocking agent. The kinetics of TNF inhibitors might be different from the kinetics of denosumab on the bone level because of the different mode of action. Therefore it is not possible to predict if a similar rapid response on GUSS™ scores will be observed. Since the whole study is a proof-of-concept and to guarantee that a later response will not be missed, the study period needs to be extended to 48 weeks and the GUSS changes between week 24 and week 48, as well as GUSS changes between baseline and week 48 will be assessed.
Other analyses of radiographic measures will be the number of patients that develop new erosive joints and the number of patients in which erosive joints start the process of remodeling between baseline and 48 weeks. From previous studies it is known that the anatomical phase scoring system is not a sensitive on short term as GUSS.

Exploratory efficacy endpoints including change in Total AUSCAN score and individual subdomain (pain, physical function and stiffness) scores from baseline, change in FIHOA scores from baseline, change in pain scales (VAS pain) from baseline, change in consumption of analgesics (paracetamol)/NSAIDs, changes in number of painful and tender joints from baseline will be analyzed similarly at week 48. Other exploratory endpoints, including the change in number of joints with effusion and/or Power Doppler signal by ultrasound, the change in HOAMRIS scores and the changes in bone densitometry measures from baseline will be analyzed. Additional details will be provided in the SAP.

Primary and exploratory analyses will be repeated on subgroups defined by presence of soft tissue swelling at baseline. Details of analyses of efficacy endpoints at different time points as well as subgroups of interest will be given in the SAP.

The primary and exploratory efficacy variables will be analyzed on the intent-to-treat (ITT) population, defined as all subjects who were randomized.To evaluate the impact of major protocol violations on the results of the study, additional analyses of the primary efficacy analysis may be conducted on the per protocol population, which consists of all ITT subjects who completed the study and are not major protocol violators. The safety population consists of all subjects who received at least one dose of double-blind study medication.

In general, mean change analyses to compare the denosumab and placebo treatment group will be performed using GEE modelling with treatment group as factor and correction for baseline radiographic damage. Correction will be made for possible dependency between joints in the same patient by using an exchangeable matrix. Categorical data will be summarized using frequencies and percentages. Continuous data will be summarized with the number of non-missing observations by mean, standard deviation, median, maximum, and minimum values. In addition to the analyses based on observed data, analysis with imputed missing data will be conducted for selected efficacy variables. The details of such sensitivity analyses will be provided in the SAP. All statistical tests will be conducted at $\alpha = 0.05$ level (two-sided), unless otherwise stated. The last evaluation prior to the first study drug will be used as baseline for all analyses.

6.2 Safety analysis
Safety analyses will be carried out using the safety population, which includes all subjects who received at least one dose of study drug. Treatment-emergent AEs and SAEs will be summarized and reported. The number and percentage of subjects experiencing adverse events will be provided by system organ class and Medical Dictionary for Drug Regulatory Activities (MedDRA) preferred term. In addition, summary of AEs by severity and relationship to study drug will be presented. Serious, severe AEs, or AEs that lead to premature study discontinuation will be listed and described in detail. Mean change in vital signs and laboratory variables at each visit will be summarized for all treated subjects, and compared between treatment groups using one way Analysis of Variance (ANOVA).

6.3 Determination of Sample size
From a placebo controlled trial with adalimumab, we learned that, the risk that an individual IP joint evolves from J/S phase to the E phase is 2-3% per year. This risk increases to 15% for joints with a clinical effusion and to 25% for a painful joint with effusion. Adalimumab therapy reduced this risk for these inflammatory joints from 25% to 3% .

From these data 50 patients in each arm are needed to demonstrate a similar effect of denosumab with a power of 80%.
This power analysis took into account the following assumptions:
1) denosumab has a similar effect as adalimumab
2) a mean of minimal 1 inflamed joint (effusion and painful) per patient at baseline and in case of inclusion of patients with non-inflammatory joints, a within patient independent risk to evolve from J/S to E phase.
3) 5% drop-out
4) The proposed study involves two treatment arms. The level of significance (α) is 0.05.
5) a similar background risk for evolution from J/S to E phase.

Considering the semi-quantitative outcome measure, GUSS, a second power analysis was performed. Several assumptions were made, based on data from a previous study (Verbruggen G et al. ARD 2012;71(6):891-8). Power calculation was performed based on the estimated difference in the semi-quantitative outcome measure, GUSS ™ over time. This outcome measure is selected to detect the radiographic progression in the selected joints after treatment. The following assumptions were made:

- the natural progression (mean change) that can be expected over a period of 6 months is + 24 units (data from the placebo treated group), the mean difference in GUSS™ change between the placebo and adalimumab treated group after 6 months was 25 units. This was considered as clinically significant since
- the smallest detectable difference of GUSS™ was calculated as 40 units (Verbruggen G et al. ARD 2010;69(5):862-7) and improved to 10 units after intensive training.
- the standard deviation of the mean change in GUSS™ is 29,
- based on the above data, a total change of at least (24+ 25) 49 units in GUSS™ in the treatment group is considered to be a clinical relevant effect from a treatment.

The proposed study involves two treatment arms. The level of significance (α) is 0.05. From previous studies performed at our department, an drop out rate of 5% can be expected.
A sample size of 25 patients in each treatment arm will have 80% power to detect a difference in mean change GUSS™ of 25 units between the placebo and treated group, assuming that the standard deviation is 29 using a t-test with a two-sided 0.05 level of significance.
Taking into account a drop out rate of 5%, a total of 27 patients (25/ 1 – 0.05) should be included in each arm.
Taken into consideration both outcome measures, a minimum of 50 patients is required in both treatment arms in order to provide sufficient power for the study.

7. Adverse Events/Adverse Event reporting

The investigator will monitor each subject for clinical and laboratory (serum Ca^{++} levels) evidence of adverse events on a routine basis throughout the study. The investigator will assess and record any adverse event in detail on the adverse event DRF including the date and time of onset, description, seriousness severity, time course, duration and outcome, relationship of the adverse event to study drug, an alternate etiology for events not considered "probably related" to study drug, final diagnosis/syndrome (if known) and any action(s) taken. Adverse events, whether in response to a query, observed by study-site personnel, or reported spontaneously by the subject, will be recorded.

All adverse events will be followed to a satisfactory conclusion.

7.1 Definitions

7.1.1. Adverse Event

An **adverse event** is defined as any untoward medical occurrence in a subject or clinical investigation subject administered a pharmaceutical product and which does not necessarily have a causal relationship with this treatment. An adverse event can therefore be any unfavorable and unintended sign (including an abnormal laboratory finding), symptom, or disease temporally associated with the use of a medicinal product, whether or not the event is considered causally related to the use of the product.

Such an event can result from use of the drug as stipulated in the protocol or labeling, as well as from accidental or intentional overdose, drug abuse, or drug withdrawal. Any worsening of a pre-existing condition or illness is considered an adverse event. Laboratory abnormalities and changes in vital signs are considered to be adverse events only if they result in permanent

or temporary discontinuation of treatment with denosumab, necessitate therapeutic medical intervention and/or if the investigator considers them to be adverse events.

An elective surgery/procedure scheduled to occur during a study will not be considered an adverse event. However, if a pre-existing condition deteriorates unexpectedly during the trial (*e.g.,* surgery performed earlier than planned), then the deterioration of the condition for which the elective surgery/procedure is being done will be considered an adverse event.

7.1.2. Serious Adverse Event

If an adverse event meets any of the following criteria, it is to be considered as serious:

**Death of Subject**	An event that results in the death of a subject.
**Life-Threatening**	An event that, in the opinion of the investigator, would have resulted in immediate fatality if medical intervention had not been taken. This does not include an event that would have been fatal if it had occurred in a more severe form.
**Hospitalization**	An event that results in an admission to the hospital for any length of time. This does not include an emergency room visit or admission to an outpatient facility.
**Prolongation of Hospitalization**	An event that occurs while the study subject is hospitalized and prolongs the subject's hospital stay.
**Congenital Anomaly**	An anomaly detected at or after birth, or any anomaly that results in fetal loss.
**Persistent or Significant Disability/Incapacity**	An event that results in a condition that substantially interferes with the activities of daily living of a study subject. Disability is not intended to include experiences of relatively minor medical significance such as headache, nausea, vomiting, diarrhea, influenza, and accidental trauma (*e.g.,* sprained ankle).
**Important Medical Event Requiring Medical or Surgical Intervention to Prevent Serious Outcome**	An important medical event that may not be immediately life-threatening or result in death or hospitalization, but based on medical judgment may jeopardize the subject and may require medical or surgical intervention to prevent any of the outcomes listed above (*i.e.,* death of subject, life-threatening, hospitalization, prolongation of hospitalization, congenital anomaly, or persistent or significant disability/incapacity). Examples of such events include allergic bronchospasm requiring intensive treatment in an emergency room or at home, blood dyscrasias or convulsions that do not result in inpatient hospitalization, or the development of drug dependency or drug abuse.
**Spontaneous Abortion**	Miscarriage experienced by study subject.
**Elective Abortion**	Elective abortion performed on study subject.

7.1.3. Adverse Event Severity

The investigator will use the following definitions to define/rate the severity of each adverse event:

**Mild**	The adverse event is transient and easily tolerated by the subject.
**Moderate**	The adverse event causes the subject discomfort and interrupts the subject's usual activities.
**Severe**	The adverse event causes considerable interference with the subject's usual activities and may be incapacitating or life-threatening.

7.1.4. Relationship to Study Drug

The investigator will use the following definitions to assess the relationship of the adverse event to the use of study drug:

**Probably Related**	An adverse event has a strong temporal relationship to study drug or recurs on re-challenge and another etiology is unlikely or significantly less likely.
**Possibly Related**	An adverse event has a strong temporal relationship to the study drug and an alternative etiology is equally or less likely compared to the potential relationship to study drug.
**Probably Not Related**	An adverse event has little or no temporal relationship to the study drug and/or a more likely alternative etiology exists.
**Not Related**	An adverse event is due to an underlying or concurrent illness or effect of another drug and is not related to the study drug (*e.g.*, has no temporal relationship to study drug or has a much more likely alternative etiology).

7.2. Adverse Event Reporting

Reporting will be consistent with current safety reporting standards. .Adverse events will be reported between the first dose administration of trial medication and the last trial related activity.
All AEs and SAE's will be recorded in the patient's file and in the CRF. All SAE's will be reported as described below.
SAE's occurring within a period of 30 days following the last intake of trial medication will also be handled as such if spontaneously reported to the investigator.

All serious adverse events (SAE) and pregnancies occurring during clinical trials must be reported by the local Principal Investigator within 2 working days after becoming aware of the SAE to:

- The local EC
- Bimetra Clinics of the University Hospital Ghent

This reporting is done by using the appropriate SAE form. For the contact details, see below.

It is the responsibility of the local Principal Investigator to report the local SAE's to the local EC.

In case the investigator decides the SAE is a SUSAR (Suspected Unexpected Serious Adverse Reaction), Bimetra Clinics will report the SUSAR to the Central EC and the CA within the timelines as defined in national legislation.

In case of a life-threatening SUSAR the entire reporting process must be completed within 7 calendar days. In case of a non life-threatening SUSAR the reporting proces must completed within 15 calendar days.

The first report of a serious adverse event may be made by telephone, e-mail or facsimile (FAX).

Contact details of Bimetra Clinics:

e-mail: bimetra.clinics@uzgent.be
tel.: 09/332 05 00
fax: 09/332 05 20

In the event of a serious, unexpected and related adverse event, the investigator will report this to the Amgen Affiliate by faxing the appropriate adverse event form within 24 hours of being made aware of the serious adverse event and simultaneously to Bimetra Clinics who will report the event to the local regulatory agency within the timelines as defined in the national legislation..
Please fax SAE form to [redacted] Pharmacovigilance Manager : Fax number 0800 80877

The investigator must provide the minimal information: i.e. trial number, subject's initials and date of birth, medication code number, period of intake, nature of the adverse event and investigator's attribution.
This report of a serious adverse event by telephone must always be confirmed by a written, more detailed report. For this purpose the appropriate SAE form will be used. Pregnancies occurring during clinical trials are considered immediately reportable events. They must be reported as soon as possible using the same SAE form. The outcome of the pregnancy must also be reported.

If the subjects are not under 24-hour supervision of the investigator or his/her staff (out-patients, volunteers), they (or their designee, if appropriate) must be provided with a "trial card" indicating the name of the investigational product, the trial number, the investigator's name and a 24-hour emergency contact number.

8. Regulatory Obligations

8.1 Informed Consent

Signed informed consent will be obtained from the subject before any study procedures are undertaken, or before any medications are withheld from the subject in order to participate in this study. Subject may withdraw consent at any time without prejudice. All efforts will be made to continue the patient follow-up until the end of the study. At withdrawal, patients will be treated and assessed according to standard recommendations and as per latest guidance for contraception criteria in female subjects of child-bearing age or partners of childbearing potential (see exclusion criteria section 4.2).

8.2 Independent Ethics Committee/Institutional Review Board

The study will be declared at www.ClinicalTrials.gov and will comply with the principles of the Declaration of Helsinki. A copy of the study protocol will be submitted for approval to the ethical committee of Ghent University Hospital and to the Federal Agency for Medicines and Health Products *(FAGG; federal agentschap voor geneesmidddelen en gezondheidsproducten)*

9. Documentation relating to the clinical trial – Trial Master File

All documents related to the trial, e.g. study protocol, source documents, case report forms, … will be handled, stored and archived according to the EU Commission's Directive 2005/28/EC 63 Chapter 4.[40]

10. Publication Policy

The results of this study will be reported and published at conferences and in peer-reviewed clinical journals. Authorship publications will follow the Uniform Requirement for

Manuscripts Submitted to Biomedical Journals (International Committee of Medical Journal Editors, 2009), which states:
Authorship credit should be based on (1) substantial contributions to conception and design, acquisition of data, or analysis and interpretation of data; (2) drafting the article or revising it critically for important intellectual content; (3) final approval of the version to be published and (4) Agreement to be accountable for all aspects of the work in ensuring that questions related to the accuracy or integrity of any part of the work are appropriately investigated and resolved. Authors should meet conditions 1, 2, 3 and 4.
For further details , see http://www.icmje.org/recommendations/browse/roles-and-responsibilities/defining-the-role-of-authors-and-contributors.html.

11.References

1- Stecher RM, Hauser H : Heberden's nodes. VII. The roentgenological and clinical appearance of degenerartive joint disease of the fingers. AmJ Roentgenol. 59 :326-337,1948
2- Crain DC : Interphalangeal osteoarthritis. Characterized by painful, inflammatory episodes resulting in deformity of the proximal and distal articulations. JAMA. 175: 1049-1053,1961
3- Peter JB, Pearson CM, Marmor L : Erosive arthritis of the hands. Arthritis Rheum. 9: 365-388,1966
4- Ehrlich GE. Osteoarthritis beginning with inflammation. Definitions and correlations. JAMA. 232: 157-159,1975
5- Verbruggen G and Veys EM. Numerical scoring systems for the anatomic evolution of osteoarthritis of the finger joints. Arthritis Rheum. 1996;**39**:308-20.
6- Zhang Y, Niu J, Kelly-Hayes M, Chaisson CE, Aliabadi P, Felson DT. Prevalence of symptomatic hand osteoarthritis and its impact on functional status among the elderly: The Framingham Study. Am J Epidemiol 2002;**156**:S225.
7- Dahaghin S, Bierma-Zeinstra SMA, Reijman M, Pols HAP, Hazes JMW, Koes BW. Prevalence and determinants of one month hand pain and hand related disability in the elderly (Rotterdam study). Ann Rheum Dis 2005;**64**:99-104.
8 Wittoek R, Vander Cruyssen B, Verbruggen G. Predictors of functional impairment and pain in erosive osteoarthritis of the interphalangeal joints: comparison with controlled inflammatory arthritis.Arthritis Rheum.
9. Leeb BF, Sautner J, Andel L, Rintelen B. A scale for assessment and quantification of chronic rheumatoid affections of the hands. Rheumatology 2003; 42: 1173-78.
10- Dillon CF, Hirsch R, Rasch E, Gu Q. Symptomatic hand osteoarthritis in the United States: prevalence and functional impairment estim ates from the third U.S. National Health and Nutrition Examination Survey, 1991-1994. Am J Phys Med Rehabil. 2007;86:12-21.
11- Kellgren JH. Osteoarthrosis in patients and populations. Br Med J. 1961;2:1-6.
12- Lawrence JS, Bremner JM, Biers F. Osteoarthritis. Prevalence in the population and relationship between symptoms and X-Ray changes. Ann Rheum Dis. 1966;25:1-24.
13- Bagge E, Bjelle A, Valkenburg HA, Svanborg A. Prevalence of radiographic osteoarthritis in two elderly European populations. Rheumatology Int. 1992;12:33-8.
14- Mannoni A, Briganti MP, Di Bari M, Ferrucci L, Constanzo S, Serni U, Masotti G, Marchionni N. Epidemiological profile of symptomatic osteoarthritis in older adults: a population based study in Dicomano, Italy. Ann Rheum Dis. 2003 Jun;62:576-8.
15- Cobby M, Cushnaghan J, Creamer P, Dieppe P, Watt I. Erosive osteoarthritis: is it a separate disease entity? Clinical Radiology 1990;**42**:258-63.
16- Cavasin F, Punzi L, Ramonda R, Pianon M, Oliviero F Sfriso P, Todesco S. Prevalence of erosive osteoarthritis of the hand in a population from Venetian area. Rheumatismo 2004;56:46-50.
17- Punzi L, Ramonda R, Sfriso P. Erosive osteoarthritis. Best Pract Res Clin Rheumatol 2004 ;18:739-58.
18- Poole J, Sayer AA, Hardy R, Wadsworth M, Kuh D, Cooper C. Patterns of interphalangeal hand joint involvement of osteoarthritis among men and women: a British cohort study. Arthritis Rheum. 2003 48:3371-6.
19. Kwok WY, Kloppenburg M, Rosendaal FR, van Meurs JB, Hofman A, Bierma-Zeinstra SMA. Erosive hand osteoarthritis: its prevalence and clinical impact in the general population and symptomatic hand osteoarthritis. Ann Rheum Dis 2011;70:1238-42.

20. Haugen IK, Englund M, Aliabadi P, Niu J, Clancy M, Kvien TK, Felson DT. Prevalence, incidence and progression of hand osteoarthritis in the general population: the Framingham Osteoarthritis Study. Ann Rheum Dis 2011;70:1581-6.
21. Verbruggen G, Wittoek R, Vander Cruyssen B et al. Morbid anatomy of 'erosive osteoarthritis' of the interphalangeal finger joints: an optimised scoring system to monitor disease progression in affected joints. Ann Rheum Dis. 2010;69:862-7.
22. Suda T, Takahashi N, Udagawa N et al. Modulation of osteoclast differentiation and function by the new members of the tumor necrosis factor receptor and ligand families. Endocr Rev.1999;20:345-57.
23. Kobayashi K, Takahashi N, Jimi E, et al. Tumor necrosis factor alpha stimulates osteoclast differentiation by a mechanism independent of the ODF/RANKL-RANK interaction. J Exp Med 2000;191:275-86.
24. Komine M, Kukita A, Kukita T et al. Tumor necrosis factor-alpha cooperates with receptor activator of nuclear factor kappaB ligand in generation of osteoclasts in stromal cell-depleted rat bone marrow cell culture. Bone 2001;28:474-83.
25. Wei S, Kitaura H, Zhou P et al. IL-1 mediates TNF-induced osteoclastogenesis. J Clin Invest 2005;115:282-90.
26- Zwerina J, Redlich K, Polzer K et al. TNF-induced structural joint damage is mediated by IL-1. Proc Natl Acad Sci U S A. 2007;104:11742-7.
27. Lefebvre V, Peeters-Joris C, Vaes G. Modulation by interleukin 1 and tumor necrosis factor alpha of production of collagenase, tissue inhibitor of metalloproteinases and collagen types in differentiated and dedifferentiated articular chondrocytes. Biochim Biophys Acta. 1990;1052:366-78.
28. Wittoek R, Carron P, Verbruggen G. Structural and inflammatory sonographic findings in erosive and non-erosive osteoarthritis of the interphalangeal finger joints. Ann Rheum Dis. 2010;69:2173-6.
29. Jans L, De Coninck T, Wittoek R et al. 3 T DCE-MRI assessment of synovitis of the interphalangeal joints in patients with erosive osteoarthritis for treatment response monitoring. Skeletal Radiol. 2013;42:255-60.
30- Bathon JM, Martin RW, Fleischmann RM, Tesser JR, Schiff MH, Keystone EC, Genovese MC, Chester Wasko M, Moreland LW, Weaver AL, Markenson J, Finck BK. A Comparison of Etanercept and Methotrexate in Patients with Early Rheumatoid Arthritis. NEJM. 2000; 343:1586-93.
31- Lipsky PE, van der Heijde DMFM, St. Clair EW, Furst DE, Breedveld FC, Kalden JR, Smolen JS, Weisman M, Emery P, Feldmann, Gregory R. Harriman GR, Maini RN. Infliximab and Methotrexate in the Treatment of Rheumatoid Arthritis. NEJM. 2000; 343:1594-602.
32. Alten R, Gram H, Joosten LA, van den Berg WB, Sieper J, Wassenberg S, Burmester G, van Riel P, Diaz-Lorente M, Bruin GJ, Woodworth TG, Rordorf C, Batard Y, Wright AM, Jung T. The human anti-IL-1 beta monoclonal antibody ACZ885 is effective in joint inflammation models in mice and in a proof-of-concept study in patients with rheumatoid arthritis. Arthritis Res Ther. 2008;10:R67.
33. Cohen SB, Dore RK, Lane NE, Ory PA, Peterfy CG, Sharp JT, van der Heijde D, Zhou L, Tsuji W, Newmark R; Denosumab Rheumatoid Arthritis Study Group. Denosumab treatment effects on structural damage, bone mineral density, and bone turnover in rheumatoid arthritis: a twelve-month, multicenter, randomized, double-blind, placebo-controlled, phase II clinical trial. Arthritis Rheum. 2008;58:1299-309.

34. Ishiguro N, Tanaka Y, Yamanaka H, Yoneda T, Ohira T, Okubo N, Genant HK, van der Heijde D and Takeuchi T. Consistent Inhibition of Bone Destruction By Denosumab in Important Subgroups of Japanese Patients with Rheumatoid Arthritis. Arthritis Rheumatol 2014; 66:11(Suppl): S831/Presented at ACR meeting 2014
35. Verbruggen G, Wittoek R, Cruyssen BV, Elewaut D. Tumour necrosis factor blockade for the treatment of erosive osteoarthritis of the interphalangeal finger joints: a double blind, randomised trial on structure modification. Ann Rheum Dis. 2012 Jun;71(6):891-8.
36. Prolia SmPC. Sections 4.4 & 4.838.
37. Bone HG, Chapurlat R, Brandi ML, Brown JP, Czerwinski E, Krieg MA, Mellström D, Radominski SC, Reginster JY, Resch H, Ivorra JA, Roux C, Vittinghoff E, Daizadeh NS, , Bradley MN, Franchimont N, Geller ML, Wagman RB, Cummings SR, Papapoulos S. The effect of three or six years of denosumab exposure in women with postmenopausal osteoporosis: results from the FREEDOM extension. Clin Endocrinol Metab. 2013;98:4483-92.
38. Papapoulos S, et al. Eight Years of Denosumab Treatment in Postmenopausal Women with Osteoporosis: Results From the First Five Years of the FREEDOM extension. WCO-IOF-ESCEO Congress 2014. Osteoporis Int 2014; 25 (Suppl 2):S118
39. Haugen IK, Østergaard M, Eshed I, McQueen FM, Bird P, Gandjbakhch F, Foltz V, Genant H, Peterfy C, Lillegraven S, Haavardsholm EA, Bøyesen P, Conaghan PG.3. Iterative development and reliability of the OMERACT hand osteoarthritis MRI scoring system. J Rheumatol. 2014 Feb;41(2):386-91
40. http://eur-lex.europa.eu/LexUriServ/LexUriServ.do?uri=OJ:L:2005:091:0013:0019:en

12.Appendices

Appendix 1. Scoring systems

A. Categorical scoring system was proposed for the progressive radiographic changes in IP finger joint OA. These changes were characterized by complete loss of the joint space preceding or coinciding with the appearance of subchondral cysts eroding the entire subchondral plate. These erosive episodes subsided spontaneously and were followed by processes of repair.[28]

The anatomical phases in the evolution of IP finger joint OA are the following.

Normal ('N') joints: no signs of OA.
Stationary ('S') phase: classical appearance of OA. Small ossification centers and osteophytes are present at the joint margins. They can both increase in size and discrete narrowing of the joint space can occur.
Loss of joint space ('J' phase): after remaining for a variable time in the stationary phase, some joints (almost exclusively PIPs or DIPs) become destroyed. The joint space completely disappears within a relatively short period of time.
Erosive ('E') phase: concurrently with or shortly after the disappearance of the articular cartilage (J phase), the subchondral plate becomes eroded. The appearance is that of a pseudo-enlargement of an irregular joint space. Roentgenograms obtained at yearly intervals showed that changes in phases from 'S' over 'J' to 'E' could occur within one year. This destructive 'J' and 'E' phases are always followed by repair or remodeling.
Remodeling ('R') phase: new irregular sclerotic subchondral plates are formed, and in between these a new joint space becomes visible. Huge osteophytes are formed during this phase. No further evolution is seen in remodeled joints.

B. A quantitative radiographic scoring system, the Ghent University Scoring System, GUSS® [29], is a reliable method to score radiographic change over time in erosive IP OA and detects more progression over a shorter period of time than the classical scoring system. Erosive progression and signs of repair or remodeling are then scored by indicating the proportions of normal subchondral bone, subchondral plate and joint space over time.

The subchondral bone area. The proportions of the subchondral bone area with normal/abnormal-looking bone architecture were assessed in a quadrangle square of which the side equalled the width of the joint space. The joint space was positioned in the centre of this square (figure 2A). In this square, regions where osteolytic activity and remodelling caused a disarrangement of the trabecular pattern, as well as areas where a complete loss of the trabecular structure had occurred, are defined.
Identifiable osteolytic subchondral bone areas are marked on the radiographs and proportions of remaining intact subchondral bone will be calculated, considering the delineated IP joint area being the 100% value.

The subchondral bone plate. In an IP joint that had completely lost its joint space, an existing subchondral plate was defined as a regular radio-opaque linear structure within the position of the original joint space. When the joint space was still identifiable, the subchondral bone plate was identified as a regular linear radio-opaque bone margin flanking the joint space.

Identifiable linear subchondral plate structures were marked on the radiographic images and proportions of remaining subchondral bone plate were computed, considering a twofold joint space width being the 100% value (figure 2B).

The joint space was recognized as a radiotranslucent area bordered with two subchondral plates. Identifiable joint spaces were marked on the radiographic images. Proportions of remaining joint space were estimated as the proportion of the joint width, considering the total joint space width being the 100% value (figure 2B).

Computation of the changes in IP joints in “J”, “E” and “E/R” phases. Pictures from the IP joints at three time points in the correct sequence will be read and used by the readers to evaluate the extent of the pathological changes in subchondral bone architecture, and to estimate the presence/absence of both subchondral bone plate and synovial joint space. Proportional changes in these three variables will be recorded. The sum of the three separate scorings constituted the total IP joint score. Equal weight will be attributed to each of the subdomains.

Appendix 2. Overall assessments

	admin dmab/plac	clinical assessm	safety	laboratory						CR hand	US hand	DXA	ECG
				PBC	serum chem	BTM	25OH Vit D	Ca++	preg test*				
SCREENING		X		X	X	X	X	X	X	X	X		X
BASELINE	X	X	X								X	X	
WEEK 6			X										
WEEK 12	X	X	X	X	X	X		X	X		X		
WEEK 24	X	X	X					X	X	X			
WEEK 36	X	X	X					X	X				
WEEK 48	X	X	X	X	X	X		X	X	X	X	X	
WEEK 60	X	X	X					X	X				
WEEK 72	X	X	X			X		X	X	X			
WEEK 84	X	X	X					X	X				
WEEK 96		X	X	X	X	X		X	X	X		X	
								* if appropriate					

dmab: denosumab; plac: placebo; PBC: peripheral blood cell count; chem: chemistry; BTM: bone turnover markers
preg: pregnancy - sticks to be provided by the rheumatology dept.; CR: conventional radiography; US: ultrasound;
MRI: magnetic resonace imaging; ECG: electrocardiogramDXA: dual energy X-ray absorptiomery

Basic Serum chemistry will include urea, creatinine, ASAT, ALAT, Albumin. Depending on the individual patient, additional parameters may be added.

W36 is the timing for the last IP dose in the blinded period. All patients will receive a denosumab injection at W48 after the assessment. This would be the first denosumab dose administered in the open label phase.

Study Protocol

RANKL-blockade for the treatment of erosive osteoarthritis (OA) of interphalangeal finger joints

Randomized, double blind, placebo-controlled study to evaluate the efficacy of denosumab 60mg sc every 3 months in patients with erosive osteoarthritis of the interphalangeal finger joints

Principal Investigators:
[redacted]

Dept. of Rheumatology – Ghent University Hospital

EudraCT number: 2015-003223-53
Protocolnumber: AGO/2015/008

[redacted]

[redacted]

[redacted]

Protocol Signature Page

Principal/Chief Investigator signature

I confirm that I have read and understood protocol version xx dated xx January 2015. I agree to comply with the study protocol, the principals of GCP, research governance, clinical trial regulations and appropriate reporting requirements.

Signature.. Date..29 / AUG / 2018..........

Print name ....[redacted]..............................

Protocol synopsis

**Study Type**	Investigator Sponsored Study
**Funder**	Amgen
**Study Design**	This is a randomized, double blind placebo controlled one-site proof-of-concept study in subjects with erosive osteoarthritis (OA) of interphangeal (IP) finger joints.<br>A total of 100 subjects will be enrolled into the study: 48 weeks placebo controlled double-blind phase with denosumab 60 mg every 12 weeks, followed by a 48-week open-label phase in which all subjects will receive denosumab.
**Investigational Therapy**	Denosumab 60 mg subcutaneous injection every 12 weeks. All subjects will receive Calcium/vit D supplementation.
**Efficacy Objectives**	The **primary objective** is to assess the effect of denosumab on the reduction of radiographic erosive progression using GUSS™ (Ghent University Score System).<br>The **secondary objective** is to assess the effect of denosumab on the reduction of radiographic erosive progression as defined by diminishing the appearance of new erosive IP finger joints.<br>The **exploratory objective** is mainly to assess the effect of denosumab on clinical variables, as well as ultrasonography and DEXA parameters.
**Main Endpoints**	**Primary Endpoint:** The change in the negative evolution of GUSS™ scores in the target IP joints from baseline to week 24.<br>**Other Endpoints:** 1) The change in the negative evolution of GUSS™ scores in the target IP joints from week 24 to week 48 and from baseline to week 48. 2) The number of patients that develop new erosive IP joints ('S/J' to 'E' phase joints) at 48 weeks; 3) The number of 'S/J' IP joints that develop 'E' phases at 48 weeks.
**Hypothesis**	**The main hypothesis** is that the repeated administration of denosumab 60 mg Q3 months can lead to reduce structural damage in erosive hand OA.
**Study Sites**	1 site – the Ghent site
**Subjects**	100 subjects
**Enrolment**	18 months
**Main Eligibility Criteria**	Males and females ≥ 30 years of age, with hand erosive OA:<br>1) having suffered from transient inflammatory attacks of the IP finger joints<br>2) showing at the time of enrolment inflammatory signs and at least one IP finger joint with the typical X-rays appearance of a 'J' or 'E' phase joint

**Study treatment Duration**	96 weeks

Table of Contents

- Protocol Title and Investigators
- Protocol signature page
- Protocol synopsis

1. Background and Rationale
1.1 Disease background
1.2 Denosumab
1.3 Rationale for study design
1.4 Hypotheses
2. Study Objectives and Endpoints
3. Experimental Plan
3.1 Study design and schematic
3.2 Number of sites
3.3 Number of subjects
3.4 Estimated study duration
4. Subject Eligibility
4.1 Inclusion criteria
4.2 Exclusion criteria
5. Treatment and Study Procedures
5.1 Investigational product
5.2 Reporting requirements for investigational product complaints
5.3 Concomitant therapy
5.4 Study procedures and schedule of assessments
6. Statistical and Analytical Plans
6.1 Efficacy analysis
6.2 Safety analysis
6.3 Determination of sample size
7. Adverse Events/Adverse Event reporting
7.1 Definitions
7.1.1. Adverse Event
7.1.2. Serious Adverse Event
7.1.3. Adverse Event Severity
7.1.4. Relationship to Study Drug
7.2. Adverse Event Reporting
8. Regulatory obligations
8.1Informed Consent
8.2 Independent Ethics Committee/Institutional Review Board
9. Documentation relating to the clinical trial- trial master file
10. Publication Policy
10. References
11. Appendices
Appendix 1. Scoring systems
Appendix 2. Overall assessments

1. Background and Rationale

1.1 Disease background

Erosive osteoarthritis (OA) of the interphalangeal (IP) finger joints is considered an inflammatory subset of osteoarthritis of the hand. Its inflammatory clinical presentation and destructive nature are unmistakable. [1,2,3,4,5] The cumulation of destructive changes in the IP joints eventually results in considerable disability.[6,7,8] There are no significant differences in hand function, stiffness and level of pain between patients with hand OA and rheumatoid arthritis. Scores for both patient groups differ significantly from those of healthy controls.[9]
Patients with erosive OA show more functional impairment and significantly more pain compared to patients with controlled inflammatory arthritis affecting the hands. The acquired structural damage of the IP joints due to destructive/reparative phenomena is the largest contributor to functional limitations.[8]
Radiological prevalence of moderate to severe hand OA is estimated to occur in 7.3% (2.65 million) US adults aged 60+ years.[10] Similar data have been reported in European countries.[7,11,12,13,14]

A significant proportion of these patients suffer from the erosive type of hand OA. In a prospective study of 500 consecutive patients attending a rheumatology clinic with symptomatic limb joint OA, 4.8% cases were identified with erosive IP joint OA.[15]

In a survey on the entire health district in the Venetian area, 2.2% out of 640 subjects aged 40+ years had erosive OA of their IP joints.[16] Mainly women in the perimenopauzal age were affected.[17]

Even higher prevalences were seen in a British cohort study[18] on 2.986 people[18]. Numbers in this study were based on clinics and the authors proposed that a proportion of their polyarticular cases were “inflammatory types of OA in association with erosions”. This assumption was based on an earlier study where clinical examination was validated against hand radiography (Egger et al., J Rheumatol 1995;22:1509–13).
Though the proportions of “erosive IP OA” reported here were probably overrated, the prevalence of what is considered to be “erosive IP OA” in this 53 years of age population was twice as high in women (10,6%), compared to men (5,9%).
More recently, these data were confirmed in 2 large population studies where the prevalence of radiographic erosive IP OA in subjects over 55 years of age ranged between 5.0 and 9.9%.[19,20]. The prevalence for men was lower at 3.3%.

These studies showed that erosive type of hand OA occurred predominantly in women.
Haugen IK et al. et al.[20] defined erosive IP OA at a joint level as Kellgren/Lawrence ≥2 plus erosions. The authors reported a prevalence of erosive IP OA in women of 9,9%, 3 times as high as in men (3,3%). In essence, the Kwok W-Y et al. figures[19] agree with the data above.

Moreover, the Haugen IK et al.[20] reported that symptomatic OA was twice as high in women (15,9%), compared to men (8,2%). Symptomatic OA here was defined as Kellgren/Lawrence stage ≥2 plus pain/aching/stiffness.

From these epidemiological studies we can conclude that the incidence of erosive OA of the IP finger joints ranges from five to ten percent particularly in women.

The aggressive destructive nature of the erosive OA is only recognized late in the disease and the radiological image of the "exhausted" final phase mimics a robust OA. Therefore, the disease was hitherto regarded as a form of primary OA - a degenerative joint disease that is caused by biomechanical overload of the joint structures. There is so far no therapy sought or found for the structural changes in the articular tissues occurring during the course of so-called degenerative joint diseases. Thus, no therapeutic measures are available that act on underlying disease mechanisms and therefore slow down or halt the progression of tissue degradation in joints affected by erosive hand OA. The current standard treatment of care in these patients is limited to symptomatic therapy to reduce pain.

There is still lack of agreement concerning the nature and specificity of erosive IP joint OA. Obviously, in erosive IP OA an important bone resorption is noted in the subchondral bone of IP finger joints, this bone resorption is readily visualized on conventional radiographs (Figure 1). The osteolytic 'erosive' lesions result in the collaps of the subchondral plate which supports the overlaying articular cartilage.[5,21] This is compatible with a pathologic osteoclast activity supported by the effects of RANKL (Receptor Activator of Nuclear Factor kappa-β Ligand).[22] RANKL is a key driver of maturation and activation of osteoclasts in bone in health and disease.[22] In pathologic conditions, RANKL can be strongly induced in a variety of cell types including stromal cells under the influence of locally produced proinflammatory cytokines such TNFα [23,24] and IL-1β.[25,26]
At the same time, a resorption of articular cartilage of the affected IP joints is also noted. As a result, the joint space gradually disappears on X-rays. Likely key factors in this process are TNF and IL-1 which both have important catabolic effects on human chondrocytes.[27] Indeed, during the course of the disease inflammatory processes in the synovial membrane of IP finger joints could be visualized.[28,29] Cytokines release thereof will have important catabolic effects on the neighbouring chondrocytes.

Thus, similar as observed in other destructive processes noted in inflammatory rheumatic diseases, the **TNF→ IL-1→ RANKL-pathway** appears to be a key therapeutic target in erosive hand OA.

Blockade of these cytokines has shown to delay ongoing tissue destruction in murine arthritis and in rheumatoid arthritis in human. [30,31,32,33,34]

Recently, TNFα-blockade was shown to retard the progression of joint damage in erosive IP finger joint OA.[35]

Considering the analogies between rheumatoid arthritis and erosive IP OA in the metabolic pathways that mediate tissue destruction, and the lack of any structure modifying treatment option in the latter, a pilot study exploring the effects of Denosumab on ongoing tissue destruction in IP finger joint OA is proposed.

1.2 Denosumab

Denosumab (Amgen), is a fully human monoclonal antibody designed to inhibit RANKL

(RANK Ligand). RANKL binds to RANK, which exists as a cell surface receptor molecule on "pre"-osteoclasts: precursors of osteoclasts.

Binding of RANKL to RANK acts as the primary signal for bone removal in normal physiological bone remodeling and in a number of pathological conditions, e.g. malignant tumors and bone metastasis.

Activation of RANK by RANKL promotes the maturation of pre-osteoclasts into osteoclasts. Denosumab inhibits osteoclasts' maturation, function and survival by binding to and inhibiting RANKL. This mimics the natural action of osteoprotegerin, an endogenous RANKL inhibitor that presents with decreasing concentrations in patients who are suffering from osteoporosis. This protects bone from degradation, and helps to counter the progression of the disease.

Denosumab was approved by the EMA for use in postmenopausal women with osteoporosis at increased risk for fracture at the dose of 60 mg sc every 6 months (Prolia®), and for the prevention of skeletal-related events in patients with bone metastasis from solid tumors at the dose of 120 mg every 4 weeks (XGEVA®).
More recently, denosumab was shown to retard the progression of structural lesions in rheumatoid arthritis, an unapproved indication for the drug.[33,34] Its dosing and safety profile depended on the different medical conditions in which the drug was used. Patients with osteoporosis and rheumatoid arthritis received 60 mg and up to 180 mg injected SC, every 6 months, respectively.

Experience from clinical studies indicates that side effects depend on the dosage.

According to Prolia® Summary of Product Characteristics (SmPC) [36], pain in extremities and musculoskeletal pain (including back pain and joint pain) were among the most common adverse reactions.
In patients treated for osteoporosis a rare unwanted effect included low calcium levels, especially when in case of an impaired kidney function. Patients must therefore be adequately supplemented with calcium and vitamin D levels before starting and during denosumab therapy. In the postmarketing setting, rare cases of severe symptomatic hypocalcaemia have been reported. Clinical monitoring of calcium level is recommended before each dose and, in patients predisposed to hypocalcaemia, within two weeks after the initial dose.
There have been rare cases of atypical femoral fracture reported in association with Prolia.
Infections of the urinary and respiratory tracts were reported as well as cellulitis, ear infection and diverticulitis. The SmPC includes a Warning Statement regarding skin infections (predominantly cellulitis) leading to hospitalization. It has been proposed that this increase in infections under denosumab treatment might be connected to the role of RANKL in the immune system.
Cataracts, constipation, skin rashes and eczema were also seen.
Osteonecrosis of the jaw (ONJ) was reported rarely in Prolia osteoporosis clinical development program. Primarily, at the high dosages used in patients with bone metastases, similarly to bisphosphonates, denosumab appeared to be implicated in increasing the risk of osteonecrosis of the jaw (ONJ) especially following extraction of teeth or oral surgical procedures.

In the post-marketing setting, rare events of drug-related hypersensitivity, including rash, urticaria, facial swelling, erythema, and anaphylactic reactions have been reported.

In the FREEDOM extension study[37,38], with up to 8 years of denosumab 60 mg Q6M exposure, the incidence rates of adverse events did not increase over time.

Denosumab safety data were reported in RA phase 2 studies[33,34]. The safety profile appears to be consistent with that in patients with postmenopausal osteoporosis. Denosumab did not have an effect on RA disease activity, as measured by the ACR response criteria, the DAS28 scores, and the occurrence of RA flares.

1.3 Rationale for study design

In RA, the initial changes are seen in the synovium where inflammatory lymphomyeloid cells massively produce TNF, and secondarily, IL-1 and RANKL. These two cytokines are responsible for the invasion of the adjacent cartilage and bone by the inflamed and proliferative synovial pannus.

In erosive IP joint OA, the osteolytic changes in subchondral bone occur before or concurrently with resorption of cartilage. The primary drivers of the cartilage damage thus are these osteolytic processes in the subchondral bone area and the collapse of the subchondral bone plate. RANKL is the cytokine primarily responsible for this osteolytic (osteoclast) activity.

The enhanced osteoclast activity and tissue remodeling initially seen in arthritic IP joint bone is clearly illustrated in figure 1.

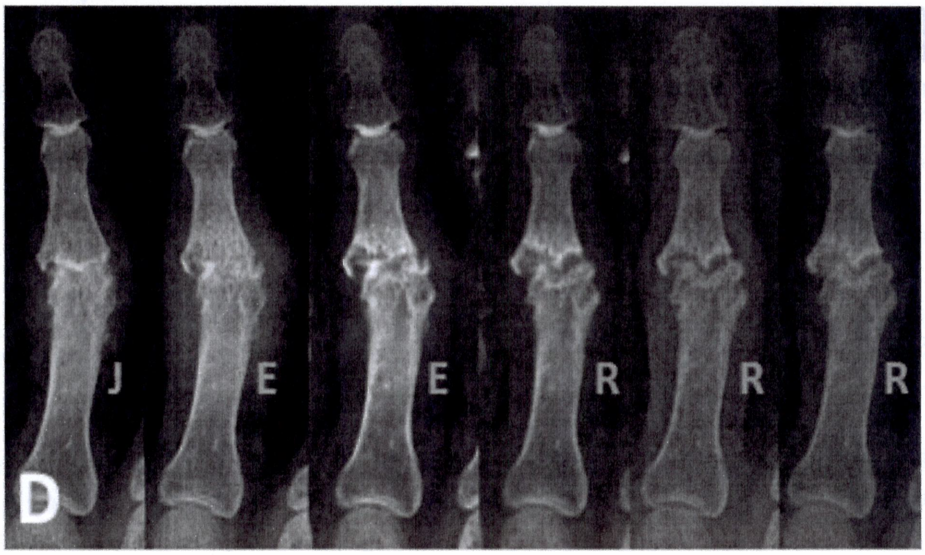

Figure 1: radiographic progression of a proximal IP joint from 'J' phase with loss of joint space to the 'E' phase with osteolytic activity in the subchondral bone area, and final remodeling of the destroyed tissues (R). Radiographs were taken with 6-months interval.

The effect of TNF alpha inhibitors on disease progression, previously seen in erosive IP joint OA[24], was an indirect effect on osteoclast activation. Obviously, this effect would be larger by directly inhibiting osteoclasts with Denosumab. Once the erosive process is blocked with

Denosumab, subchondral bone remodeling will be inhibited and one should see preservation of joint structure.

A proof-of-concept study is proposed herein to test the ability of repeated administration of denosumab to control the structural damage– and thus to maintain hand function - in erosive hand OA. These tests will be conducted compared to placebo during a first placebo controlled double-blind phase but also in a second open-label phase in which all subjects will receive denosumab. The 2 main factors that support conducting this second open-label phase are the following:
- This would enable the Long-term outcome assessment with the cumulative exposure over time; more substantial effect would be expected.
- The open label with help supporting patients' engagement in a placebo trial where no disease modifying drugs exist.

The adequate dose of denosumab should completely inhibit the erosive process in order to fully test the hypothesis. In the phase 2 RA studies [33,34,], the higher dose or shorter interval dosing regimen showed an earlier or a trend to more inhibition of bone destruction respectively. Considering further the well-established safety profile for denosumab at high doses, a higher frequency for denosumab 60 mg is proposed: denosumab 60 mg sc every 3 months.

1.4 Hypotheses

The main hypothesis is that the repeated administration of denosumab 60 mg every 3 months in erosive hand OA can inhibit structural progression of already affected joints and prevent occurrence of newly affected joints.
As it has been shown that denosumab, reduces structural damage in RA while having no effect on clinical symptoms [34], no clinical benefit is expected within the one-year period of this study. So, the effects of denosumab on the clinical manifestations of the disease will only be part of an exploratory study.

2. Study Objectives and Endpoints

The objective of this proof of concept study is to investigate the efficacy of denosumab 60 mg sc every 12 weeks for 48 weeks as a therapeutic intervention in erosive IP joint OA. In general, the expected outcome of this study would be the control of the structural damage.

Changes in the architecture of the joint will be assessed by the GUSS™. This score system allows an overall score to be calculated for an affected IP joint over time. The overall score is the sum of scores obtained for 3 compartments of the IP finger joint: the synovial space (articular cartilage), the subchondral bone plates and the subchondral bone area at each side of the synovial space. Overall scores, as well as scores for each individual compartment can be taken into consideration. Examples of the calculated scores for 2 different IP joints are given in appendix 1.

The **primary objective** is to assess the effect of denosumab on the reduction of radiographic erosive progression using GUSS™ (Ghent University Score System).
The **primary endpoints of this objective** is the change in the negative evolution in GUSS™ scores in the target IP joints from baseline to week 24.

Other endpoints are the changes in the negative evolution of GUSS™ scores in the target IP joints from week 24 to week 48 and from baseline to week 48.

The **secondary objective** is to evaluate a reduction in radiographic erosive progression as defined by diminishing the appearance of new erosive IP finger joints.
This will be assessed by 2 endpoints:
1. the number of patients that develop new erosive IP joints ('S/J' to 'E' phases) at 48 weeks.
2. the number of 'S/J' IP joints that develop 'E' phases at 48 weeks.

Radiological score systems are given in appendix 1.

The **exploratory** objective is to assess if denosumab provides clinical benefits (improvement of pain and functional limitations) compared to placebo. We will also evaluate the impact on ultrasonography and DEXA.
The endpoints of this objective are:
1. Changes in clinical and patient recorded outcome measures from baseline (day 1) to week 48 after administration of denosumab compared to placebo. The following outcome measures will be recorded: AUSCAN (AUStralian CANadian Osteoarthritis Hand Index), FIHOA (Functional Index of Hand Osteoarthritis), Pain on VAS scale, consumption of analgesics (paracetamol)/NSAIDs to be recorded by each patient on a diary, tenderness upon pressure, diameter of selected target joints, and grip strength of both hands.
2. Changes in sonographic inflammatory signals at week 12 and 48 compared to baseline. Inflammatory changes will be assessed by measuring the amount of effusion and Power Doppler signal (scoring on a semi-quantitative scale).
3. Effect of denosumab on bone mass densitometry score in this group of patients compared to placebo from baseline to week 48. Changes from baseline (day 1) in T-score at lumbar spine and hip measured by bone densitometry at week 48 after administration of denosumab compared to placebo.

Other exploratory endpoints are to describe the above radiographic progression parameters at the end of the open-label phase.

Safety-objective
The safety profile of denosumab 60 mg (Prolia®) every 6 months in postmenopausal women with osteoporosis at increased risk of fracture is well established (Prolia SmPC). This study will assess the safety of the administration of denosumab 60 mg every 3 months in the population of patients with erosive OA. Safety evaluations will be made by recording the incidence of AE/SAE (see also paragraph 8).

3. Experimental Plan

3.1 Study design and schematic

This is a randomized, double blind, placebo-controlled, one-site proof of concept study to investigate the effect of denosumab 60 mg every 12 weeks on the radiological evolution of erosive OA of the digital joints.
Two groups of 50 patients each will be enrolled in the study with a total treatment duration of 24 months (96 weeks): 48 weeks double-blind placebo controlled phase (denosumab (60 mg

sc every 12 weeks or placebo) followed by a 48-weeks open–label phase in which all subjects will receive denosumab 60 mg every 12 weeks in an "Open Label Design" type study.

Study schematic

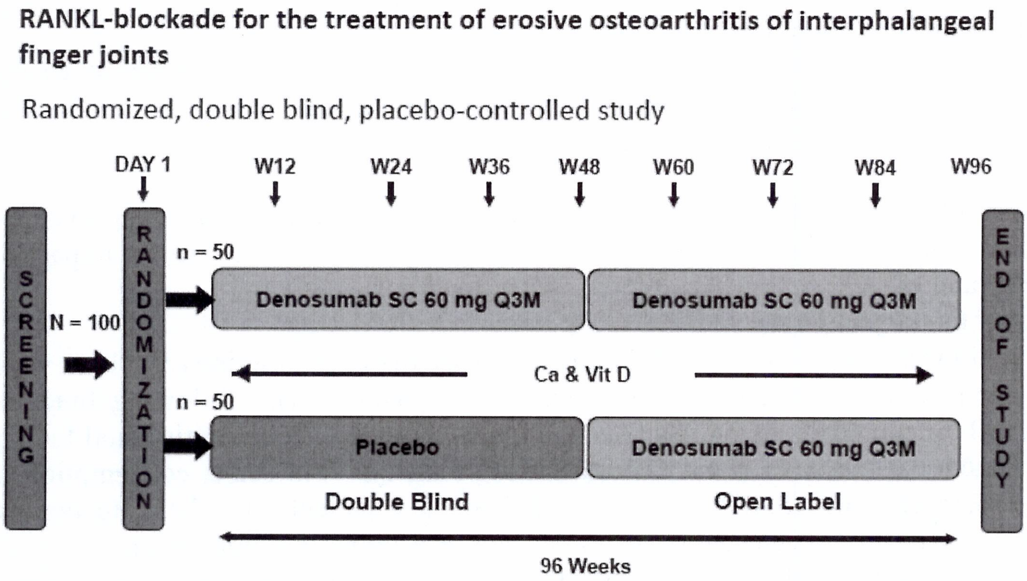

3.2 Number of sites

The study will be conducted in one site – the Ghent site in Belgium.

3.3 Number of subjects

A total of 100 subjects will be recruited in this study with an enrolment period of 18 months.

3.4 Estimated study duration

The total treatment duration per subject is 24 months (96 weeks). The expected total trial duration defined as the time from first patient first visit to last patient last visit is 50 months.

4. Subject Eligibility

4.1 Inclusion criteria

A subject will be eligible for study participation if he/she meets the following criteria:

- Males and females ≥ 30 years of age.
- Subjects with hand OA having suffered from transient inflammatory attacks of the interphalangeal finger joints characteristic for what has been termed 'inflammatory' or 'erosive' hand OA.
- Subjects with hand OA showing inflammatory signs, either clinically or ultrasonographically, of the interphalangeal finger joints.
- Subjects with hand OA in which at least 1 interphalangeal finger joint has the typical appearance on the X-rays of a 'J' or 'E' phase joint as defined by the criteria mentioned above.
- Subjects with hand OA where at least 1 interphalangeal finger joint in the 'J' or 'E' phase presents a palpable swelling.
- Able and willing to give written informed consent and to comply with the requirements of the study protocol.

4.2 Exclusion criteria

A subject will be excluded from the study if he/she meets any of the following criteria:

- Patients with known hypersensitivities to mammalian-derived drug preparations.
- Patients with clinically significant hypersensitivity to any of the components of Prolia.
- Current and/or Prior treatment with any investigational agent within 90 days, or five half-lives of the product, whichever is longer.
- Previous administration of denosumab from clinical trials or others (e.g. commercial use).
- Vitamin D deficiency [25(OH) vitamin D level < 20 ng/mL (< 49.9 nmol/L)]. Possibility of replenishment and re-screening.
- Subjects with current hypo- or hypercalcemia (normal serum calcium levels: 8.5-10.5 mg/dl or 2.12-2.62 mmol/L).
- Patients currently under bisphosphonate (BP) treatment or any use of oral BPs within 12 months of study enrollment or intravenous BPs or strontium ranelate within 5 years of study enrollment
- Prior use of any chondroprotective drug within 90 days e.g. chondroitin sulfate, glucosamine, avocado-soybean unsaponifiables, tetracyclins, corticosteroids (oral, intramuscular, intra-articular or intralesional).
- Prior use of any immunomodulating drug with possible effects on proinflammatory cytokine metabolism within 90 days a.o. corticosteroids (oral, intramuscular, intra-articular or intralesional), methotrexate, sulfasalazine, leflunomide, D-Penicillin, anti-malarials, cytotoxic drugs, TNF blocking agents.
- History of drug or alcohol abuse in the last year.
- Patients suffering from chronic inflammatory rheumatic disease (e.g. rheumatoid arthritis, spondylarthropathy, psoriatic arthritis, gout, chondrocalcinosis or other auto-immune diseases, e.g. systemic lupus erythematosus).
- History of cancer or lymphoproliferative disease within the past five years, other than a successfully and completely treated squamous cell or basal cell carcinoma of the skin or cervical dysplasia, with no recurrence within the last two years.
- History of any Solid Organ or Bone Marrow Transplant.

   - Comorbidities: significant renal function impairment (glomerular filtration < 30 ml/min/1.73m^2 or <50% of normal value), uncontrolled diabetes, unstable ischemic heart disease, congestive heart failure (NYHA III, IV), uncontrolled hypo or hyperparathyroidism, active inflammatory bowel disease, malabsorption, liver failure or chronic hepatic disease (serum AST/ALT levels 3 times above normal), recent stroke (within three months), chronic leg ulcer and any other condition (*e.g,*. indwelling urinary catheter) which, in the opinion of the investigator, would put the subject at risk by participation in the protocol.
- Subject has any kind of disorder that compromises the ability of the subject to give written informed consent and/or to comply with study procedures .
- Patient who is pregnant or planning pregnancy; if the female subject is of child-bearing age, she must use a valid mean of contraception during the study and for 9 months after last dose of study medication. For males with a partner of childbearing potential: subject refuses to use 1 effective methods of contraception for the duration of the study and for 10 months after the last dose of study medication.
- Female subjects who are breast-feeding.
- History of osteonecrosis of the jaw, and/or recent (within 3 months) tooth extraction or other unhealed dental surgery; or planned invasive dental work during the study.

5. Treatment and Study Procedures

5.1 Investigational product (see also paragraph 1.2)
The study drug used in this clinical trial is denosumab 60 mg subcutaneously every 3 months. It will be provided as sterile, solution for injection in 1 ml pre-filled syringes containing denosumab 60mg/ ml or placebo. Placebo for Denosumab will be presented in identical containers and stored/packaged the same as drug product denosumab. Denosumab prefilled syringe placebo product is supplied in a prefilled syringe as a sterile, single use, preservative free solution for subcutaneous injection. Each prefilled syringe contains 1 mL deliverable volume of buffer consisting of 10 mM sodium acetate, 5% (w/v) sorbitol, 0.01% (w/v) polysorbate 20, at a pH of 5.2. The IP is packed with 1 PFS per box. Both Denosumab and Placebo are manufactured by Amgen Inc, United States and released in the EU by Amgen Breda, Netherlands. Amgen will provide batch release certificates that will be made available with each shipment of the drug. Amgen will provide GMP certification and investigational medicinal product dossiers directly to the Belgian Agency in the regulatory submission by Amgen for this ISS. The injections will be given at the study site. Instructions for the drug handling, packaging and storage are provided in details below. Briefly, the drug will be given under the skin of the thigh, abdomen or upper arm. The clinical supplies should be stored in the refrigerator at 2-8°C. Do not freeze. Do not shake excessively. The clinical supplies must be protected from light by storing in the outer carton.

Patients who completed the 1-year interventional study will have the opportunity to enter a second 1-year open-label extension (OLE) study with Denosumab (60 mg every 12 weeks, SC). The 1-year radiographic progression of their IP finger joints will be monitored after 6 and 12 months of treatment in the OLE.

Drug Handling:
"Denosumab is supplied as a sterile, colorless to slightly yellow, preservative-free solution for injection in a 1mL prefilled syringe (PFS). The formulation of IP is 60 mg/mL denosumab per mL, formulated with10 mM Sodium Acetate, 5% Sorbitol, 0.01% Polysorbate, to a pH of 5.2. Each PFS of IP is intended for single use only. The IP is packed with 1 PFS per box. Placebo for denosumab will be presented in identical containers and stored/packaged in the same way as drug product denosumab.

The IP is shipped by air courier maintained at 2°C to 8°C in a qualified shipper suitable for biological substance shipments. IP in a PFS will arrive in a secondary packaging container and should be immediately placed in a refrigerator maintained at 2°C to 8°C in a secured location until planned use. The set point for the refrigerator should be at 5°C.
IP must be properly labelled and dispensed in accordance with current ICH GCP and local/regional requirements prior to dispensing for administration.
Before preparation check that IP:

- is visually intact and suitable for use
- is not expired
- has not been subjected to any potential temperature excursion
- label of the box and vial is correct

Prior to administration, IP may be removed from the refrigerator and brought to room temperature (up to 25°C) in the original container. This generally takes 15 to 30 minutes. Do not warm IP in any other way. Once removed from the refrigerator, IP must not be exposed to temperatures above 25°C/77°F and must be used within 24 hours. If not used within this time duration, IP must be discarded. Do not freeze IP. Protect IP from light and heat. Avoid vigorous shaking. Preparation of the clinical supplies should be performed using aseptic techniques and under sterile conditions.

All SC injections must be administered by authorized site personnel. All subjects will receive 1 SC injection at each dosing visit (of either 60mg/ml Denosumab or Placebo) administered in the subject's upper arm, upper thigh or abdomen by a trained and qualified staff member. The injection should not be administered in the same arm from which blood is drawn."

5.2 Reporting requirements for investigational product complaints:
The following could be considered potential product complaints that need to be reported to Amgen. The Investigator will use a Product Complaint Form as provided by Amgen to report any complaint. Should any such concerns or irregularities occur, the IP will not be used until Amgen confirms that it is permissible to use. Examples of Product Complaints:

- Packaging: for example, broken container or cracked container
- Devices: issues with delivery of IP by device
- Usage: for example, subject or healthcare provider cannot appropriately use the product
- Labeling: for example, missing labels, illegible labels, incorrect labels, and/or suspect labels
- Change in IP appearance: for example color change or presence of foreign material
- Unexpected quantity in bottle: for example number of tablets or amount of fluid
- Evidence of tampering or stolen material

5.3 Concomitant therapy

All patients will have a daily calcium (1000 mg) and vitamin D (880 IU) supplementation. Subjects who are current or previous users of denosumab will be excluded at screening (see exclusion criteria).

Concomitant medication: NSAIDs and analgesics are allowed throughout during the study, but the dosages are kept constant during the first 12 weeks. Patients will keep records of their daily use of symptom modifying drugs.

5.4 Study procedures and schedule of assessments

A screening visit will include a clinical assessment, a hand radiograph and the laboratory investigations required. These will comprise a calcium and vitamin D status, peripheral blood cell count (PBC), serum chemistry glucose levels, liver (ALT, AST, alkaline phosphatase) and kidney function (serum ureum, serum creatinine, GFR) tests, Bone turnover markers (BTM) and, if appropriate, a pregnancy test.

An electrocardiogram (ECG) and an ultrasound (US) exam of the IP joints are part of the screening program.

Patients will be evaluated for risk factors for ONJ before starting treatment. A dental examination with appropriate preventive dentistry is recommended prior to treatment with Prolia in patients with concomitant risk factors.

The maximum window allowed between the screening visit and the baseline visit is of 3 weeks.

Upon selection, patients will be included in the study during **the baseline visit**, which will include a clinical examination and an ultrasound (US) exam of the IP joints. Magnetic resonance imaging (MRI[39]) of the hand is optional. Study products (denosumab/placebo) will then be administered on-site by the investigator/study nurse. Calcium and vit D supplementation will be installed. Dual energy X-ray absorptiometry (DXA).

Schedule of assessments are provided in detail as Appendix 2. Clinical assessment is the standard practice and will be detailed in the CRF and the SAP. Safety assessment is clarified in the safety paragraph.

At week 6: a clinical/safety evaluation is planned.

At week 12: clinical/safety assessment, PBC and serum chemistry, serum calcium levels and BTM, US. MRI of the hand is optional. Study products (denosumab/placebo) to be administered on-site by the investigator/study nurse.

At week 24: clinical/safety assessment, serum calcium levels, hand radiographs.

Study products (denosumab/placebo) to be administered on-site by the investigator/study nurse.

At week 36: clinical/safety assessment, serum calcium levels. Study products (denosumab/placebo) to be administered.

W36 is the timing for the last IP dose in the blinded period.

At week 48: clinical/safety assessment, US, hand radiographs, DXA. Serum calcium levels, PBC and serum chemistry (glucose levels, liver and kidney function tests, and BTM. Study products (denosumab/placebo) to be administered.

The visit at week 48 is the first visit of the Open Label Extension (OLE) program, which will encompass clinical/ safety exams, laboratory tests and hand radiographs as indicated in the table. The clinical monitoring of serum calcium during the OLE phase will follow the same schedule as in the placebo controlled phase.
All patients will receive a denosumab injection at W48 after the above assessment. This would be the first denosumab dose administered in the open label phase.

Safety: Patients will be able to report any unwanted effect during the regular visits and through telephone contact at any time in between these visits. Clinical examination is part of this safety assessment. Templates for AE/SAE recording created by the Investigators will be used.

As unwanted effects – other than these reported in previous Prolia osteoporosis programs - are not expected, the collection of other laboratory safety data beyond week 12 during the randomized treatment phase is not arranged.

A negative pregnancy test will be an entry requirement in female premenopausal patients. Premenopausal patients at risk to become pregnant will be excluded if no valid anti-conceptive method is used. In practice, premenopausal women will be an absolute minority in this study population. During the study and during the OLE phase, pregnancy tests will be done before each injection of denosumab in these subjects.

6. Statistical and Analytical Plans

6.1 Efficacy analysis

Complete and specific details of the final statistical analysis will be described and fully documented in the Statisticap Analysis Plan (SAP). The SAP will be finalized prior to the database lock. The analysis will be performed using the statistical software package IBM SPSS .

Demographic and baseline characteristics will be summarized. The number of observations, mean, standard deviation, median, minimum and maximum will be summarized for continuous variables. Discrete variables will be summarized by counts and percentages.

The primary efficacy variables will be the changes from baseline to week 24 in radiographic outcome measures, more specifically changes in GUSS. The primary efficacy comparisons will be between the denosumab treatment group and the placebo treatment group using GEE modelling with treatment as factors and baseline radiographic scores as a covariate. Additional endpoints will be assessed because several assumptions are made in this pilot study that are derived from a previous clinical study with a TNF-α blocking agent. The kinetics of TNF inhibitors might be different from the kinetics of denosumab on the bone level because of the different mode of action. Therefore it is not possible to predict if a similar rapid response on GUSS™ scores will be observed. Since the whole study is a proof-of-concept and to guarantee that a later response will not be missed, the study period needs to be extended to 48 weeks and the GUSS changes between week 24 and week 48, as well as GUSS changes between baseline and week 48 will be assessed.
Other analyses of radiographic measures will be the number of patients that develop new erosive joints and the number of patients in which erosive joints start the process of remodeling between baseline and 48 weeks. From previous studies it is known that the anatomical phase scoring system is not a sensitive on short term as GUSS.

Exploratory efficacy endpoints including change in Total AUSCAN score and individual subdomain (pain, physical function and stiffness) scores from baseline, change in FIHOA scores from baseline, change in pain scales (VAS pain) from baseline, change in consumption of analgesics (paracetamol)/NSAIDs, changes in number of painful and tender joints from baseline will be analyzed similarly at week 48. Other exploratory endpoints, including the change in number of joints with effusion and/or Power Doppler signal by ultrasound, the change in HOAMRIS scores and the changes in bone densitometry measures from baseline will be analyzed. Additional details will be provided in the SAP.

Primary and exploratory analyses will be repeated on subgroups defined by presence of soft tissue swelling at baseline. Details of analyses of efficacy endpoints at different time points as well as subgroups of interest will be given in the SAP.

The primary and exploratory efficacy variables will be analyzed on the intent-to-treat (ITT) population, defined as all subjects who were randomized.To evaluate the impact of major protocol violations on the results of the study, additional analyses of the primary efficacy analysis may be conducted on the per protocol population, which consists of all ITT subjects who completed the study and are not major protocol violators. The safety population consists of all subjects who received at least one dose of double-blind study medication.

In general, mean change analyses to compare the denosumab and placebo treatment group will be performed using GEE modelling with treatment group as factor and correction for baseline radiographic damage. Correction will be made for possible dependency between joints in the same patient by using an exchangeable matrix. Categorical data will be summarized using frequencies and percentages. Continuous data will be summarized with the number of non-missing observations by mean, standard deviation, median, maximum, and minimum values. In addition to the analyses based on observed data, analysis with imputed missing data will be conducted for selected efficacy variables. The details of such sensitivity analyses will be provided in the SAP. All statistical tests will be conducted at $\alpha = 0.05$ level (two-sided), unless otherwise stated. The last evaluation prior to the first study drug will be used as baseline for all analyses.

6.2 Safety analysis

Safety analyses will be carried out using the safety population, which includes all subjects who received at least one dose of study drug. Treatment-emergent AEs and SAEs will be summarized and reported. The number and percentage of subjects experiencing adverse events will be provided by system organ class and Medical Dictionary for Drug Regulatory Activities (MedDRA) preferred term. In addition, summary of AEs by severity and relationship to study drug will be presented. Serious, severe AEs, or AEs that lead to premature study discontinuation will be listed and described in detail. Mean change in vital signs and laboratory variables at each visit will be summarized for all treated subjects, and compared between treatment groups using one way Analysis of Variance (ANOVA).

6.3 Determination of Sample size

From a placebo controlled trial with adalimumab, we learned that, the risk that an individual IP joint evolves from J/S phase to the E phase is 2-3% per year. This risk increases to 15% for joints with a clinical effusion and to 25% for a painful joint with effusion. Adalimumab therapy reduced this risk for these inflammatory joints from 25% to 3% .

From these data 50 patients in each arm are needed to demonstrate a similar effect of denosumab with a power of 80%.

This power analysis took into account the following assumptions:

1) denosumab has a similar effect as adalimumab
2) a mean of minimal 1 inflamed joint (effusion and painful) per patient at baseline and in case of inclusion of patients with non-inflammatory joints, a within patient independent risk to evolve from J/S to E phase.
3) 5% drop-out
4) The proposed study involves two treatment arms. The level of significance (α) is 0.05.
5) a similar background risk for evolution from J/S to E phase.

Considering the semi-quantitative outcome measure, GUSS, a second power analysis was performed. Several assumptions were made, based on data from a previous study (Verbruggen G et al. ARD 2012;71(6):891-8). Power calculation was performed based on the estimated difference in the semi-quantitative outcome measure, GUSS ™ over time. This outcome measure is selected to detect the radiographic progression in the selected joints after treatment. The following assumptions were made:

- the natural progression (mean change) that can be expected over a period of 6 months is + 24 units (data from the placebo treated group), the mean difference in GUSS™ change between the placebo and adalimumab treated group after 6 months was 25 units. This was considered as clinically significant since
- the smallest detectable difference of GUSS™ was calculated as 40 units (Verbruggen G et al. ARD 2010;69(5):862-7) and improved to 10 units after intensive training.
- the standard deviation of the mean change in GUSS™ is 29,
- based on the above data, a total change of at least (24+ 25) 49 units in GUSS™ in the treatment group is considered to be a clinical relevant effect from a treatment.

The proposed study involves two treatment arms. The level of significance (α) is 0.05. From previous studies performed at our department, an drop out rate of 5% can be expected.
A sample size of 25 patients in each treatment arm will have 80% power to detect a difference in mean change GUSS™ of 25 units between the placebo and treated group, assuming that the standard deviation is 29 using a t-test with a two-sided 0.05 level of significance.
Taking into account a drop out rate of 5%, a total of 27 patients (25/ 1 – 0.05) should be included in each arm.
Taken into consideration both outcome measures, a minimum of 50 patients is required in both treatment arms in order to provide sufficient power for the study.

7. Adverse Events/Adverse Event reporting

The investigator will monitor each subject for clinical and laboratory (serum Ca^{++} levels) evidence of adverse events on a routine basis throughout the study. The investigator will assess and record any adverse event in detail on the adverse event DRF including the date and time of onset, description, seriousness severity, time course, duration and outcome, relationship of the adverse event to study drug, an alternate etiology for events not considered "probably related" to study drug, final diagnosis/syndrome (if known) and any action(s) taken. Adverse events, whether in response to a query, observed by study-site personnel, or reported spontaneously by the subject, will be recorded.

All adverse events will be followed to a satisfactory conclusion.

7.1 Definitions

7.1.1. Adverse Event

An **adverse event** is defined as any untoward medical occurrence in a subject or clinical investigation subject administered a pharmaceutical product and which does not necessarily have a causal relationship with this treatment. An adverse event can therefore be any unfavorable and unintended sign (including an abnormal laboratory finding), symptom, or disease temporally associated with the use of a medicinal product, whether or not the event is considered causally related to the use of the product.

Such an event can result from use of the drug as stipulated in the protocol or labeling, as well as from accidental or intentional overdose, drug abuse, or drug withdrawal. Any worsening of a pre-existing condition or illness is considered an adverse event. Laboratory abnormalities and changes in vital signs are considered to be adverse events only if they result in permanent

or temporary discontinuation of treatment with denosumab, necessitate therapeutic medical intervention and/or if the investigator considers them to be adverse events.

An elective surgery/procedure scheduled to occur during a study will not be considered an adverse event. However, if a pre-existing condition deteriorates unexpectedly during the trial (*e.g.,* surgery performed earlier than planned), then the deterioration of the condition for which the elective surgery/procedure is being done will be considered an adverse event.

7.1.2. Serious Adverse Event

If an adverse event meets any of the following criteria, it is to be considered as serious:

**Death of Subject**	An event that results in the death of a subject.
**Life-Threatening**	An event that, in the opinion of the investigator, would have resulted in immediate fatality if medical intervention had not been taken. This does not include an event that would have been fatal if it had occurred in a more severe form.
**Hospitalization**	An event that results in an admission to the hospital for any length of time. This does not include an emergency room visit or admission to an outpatient facility.
**Prolongation of Hospitalization**	An event that occurs while the study subject is hospitalized and prolongs the subject's hospital stay.
**Congenital Anomaly**	An anomaly detected at or after birth, or any anomaly that results in fetal loss.
**Persistent or Significant Disability/Incapacity**	An event that results in a condition that substantially interferes with the activities of daily living of a study subject. Disability is not intended to include experiences of relatively minor medical significance such as headache, nausea, vomiting, diarrhea, influenza, and accidental trauma (*e.g.*, sprained ankle).
**Important Medical Event Requiring Medical or Surgical Intervention to Prevent Serious Outcome**	An important medical event that may not be immediately life-threatening or result in death or hospitalization, but based on medical judgment may jeopardize the subject and may require medical or surgical intervention to prevent any of the outcomes listed above (*i.e.,* death of subject, life-threatening, hospitalization, prolongation of hospitalization, congenital anomaly, or persistent or significant disability/incapacity). Examples of such events include allergic bronchospasm requiring intensive treatment in an emergency room or at home, blood dyscrasias or convulsions that do not result in inpatient hospitalization, or the development of drug dependency or drug abuse.
**Spontaneous Abortion**	Miscarriage experienced by study subject.
**Elective Abortion**	Elective abortion performed on study subject.

7.1.3. Adverse Event Severity
The investigator will use the following definitions to define/rate the severity of each adverse event:

**Mild**	The adverse event is transient and easily tolerated by the subject.
**Moderate**	The adverse event causes the subject discomfort and interrupts the subject's usual activities.
**Severe**	The adverse event causes considerable interference with the subject's usual activities and may be incapacitating or life-threatening.

7.1.4. Relationship to Study Drug
The investigator will use the following definitions to assess the relationship of the adverse event to the use of study drug:

**Probably Related**	An adverse event has a strong temporal relationship to study drug or recurs on re-challenge and another etiology is unlikely or significantly less likely.
**Possibly Related**	An adverse event has a strong temporal relationship to the study drug and an alternative etiology is equally or less likely compared to the potential relationship to study drug.
**Probably Not Related**	An adverse event has little or no temporal relationship to the study drug and/or a more likely alternative etiology exists.
**Not Related**	An adverse event is due to an underlying or concurrent illness or effect of another drug and is not related to the study drug (*e.g.*, has no temporal relationship to study drug or has a much more likely alternative etiology).

7.2. Adverse Event Reporting
Reporting will be consistent with current safety reporting standards. .Adverse events will be reported between the first dose administration of trial medication and the last trial related activity.
All AEs and SAE's will be recorded in the patient's file and in the CRF. All SAE's will be reported as described below.
SAE's occurring within a period of 30 days following the last intake of trial medication will also be handled as such if spontaneously reported to the investigator.

All serious adverse events (SAE) and pregnancies occurring during clinical trials must be reported by the local Principal Investigator within 2 working days after becoming aware of the SAE to:

- The local EC
- Bimetra Clinics of the University Hospital Ghent

This reporting is done by using the appropriate SAE form. For the contact details, see below.

It is the responsibility of the local Principal Investigator to report the local SAE's to the local EC.

In case the investigator decides the SAE is a SUSAR (Suspected Unexpected Serious Adverse Reaction), Bimetra Clinics will report the SUSAR to the Central EC and the CA within the timelines as defined in national legislation.

In case of a life-threatening SUSAR the entire reporting process must be completed within 7 calendar days. In case of a non life-threatening SUSAR the reporting proces must completed within 15 calendar days.

The first report of a serious adverse event may be made by telephone, e-mail or facsimile (FAX).

Contact details of Bimetra Clinics:
e-mail: bimetra.clinics@uzgent.be
tel.: 09/332 05 00
fax: 09/332 05 20

In the event of a serious, unexpected and related adverse event, the investigator will report this to the Amgen Affiliate by faxing the appropriate adverse event form within 24 hours of being made aware of the serious adverse event and simultaneously to Bimetra Clinics who will report the event to the local regulatory agency within the timelines as defined in the national legislation..
Please fax SAE form to [redacted], Pharmacovigilance Manager : Fax number 0800 80877

The investigator must provide the minimal information: i.e. trial number, subject's initials and date of birth, medication code number, period of intake, nature of the adverse event and investigator's attribution.
This report of a serious adverse event by telephone must always be confirmed by a written, more detailed report. For this purpose the appropriate SAE form will be used. Pregnancies occurring during clinical trials are considered immediately reportable events. They must be reported as soon as possible using the same SAE form. The outcome of the pregnancy must also be reported.

If the subjects are not under 24-hour supervision of the investigator or his/her staff (out-patients, volunteers), they (or their designee, if appropriate) must be provided with a "trial card" indicating the name of the investigational product, the trial number, the investigator's name and a 24-hour emergency contact number.

8. Regulatory Obligations

8.1 Informed Consent

Signed informed consent will be obtained from the subject before any study procedures are undertaken, or before any medications are withheld from the subject in order to participate in this study. Subject may withdraw consent at any time without prejudice. All efforts will be made to continue the patient follow-up until the end of the study. At withdrawal, patients will be treated and assessed according to standard recommendations and as per latest guidance for contraception criteria in female subjects of child-bearing age or partners of childbearing potential (see exclusion criteria section 4.2).

8.2 Independent Ethics Committee/Institutional Review Board

The study will be declared at www.ClinicalTrials.gov and will comply with the principles of the Declaration of Helsinki. A copy of the study protocol will be submitted for approval to the ethical committee of Ghent University Hospital and to the Federal Agency for Medicines and Health Products *(FAGG; federal agentschap voor geneesmidddelen en gezondheidsproducten)*

9. Documentation relating to the clinical trial – Trial Master File

All documents related to the trial, e.g. study protocol, source documents, case report forms, … will be handled, stored and archived according to the EU Commission's Directive 2005/28/EC 63 Chapter 4.[40]

10. Publication Policy

The results of this study will be reported and published at conferences and in peer-reviewed clinical journals. Authorship publications will follow the Uniform Requirement for

Manuscripts Submitted to Biomedical Journals (International Committee of Medical Journal Editors, 2009), which states:
Authorship credit should be based on (1) substantial contributions to conception and design, acquisition of data, or analysis and interpretation of data; (2) drafting the article or revising it critically for important intellectual content; (3) final approval of the version to be published and (4) Agreement to be accountable for all aspects of the work in ensuring that questions related to the accuracy or integrity of any part of the work are appropriately investigated and resolved. Authors should meet conditions 1, 2, 3 and 4.
For further details , see http://www.icmje.org/recommendations/browse/roles-and-responsibilities/defining-the-role-of-authors-and-contributors.html.

11.References

1- Stecher RM, Hauser H : Heberden's nodes. VII. The roentgenological and clinical appearance of degenerartive joint disease of the fingers. AmJ Roentgenol. 59 :326-337,1948

2- Crain DC : Interphalangeal osteoarthritis. Characterized by painful, inflammatory episodes resulting in deformity of the proximal and distal articulations. JAMA. 175: 1049-1053,1961

3- Peter JB, Pearson CM, Marmor L : Erosive arthritis of the hands. Arthritis Rheum. 9: 365-388,1966

4- Ehrlich GE. Osteoarthritis beginning with inflammation. Definitions and correlations. JAMA. 232: 157-159,1975

5- Verbruggen G and Veys EM. Numerical scoring systems for the anatomic evolution of osteoarthritis of the finger joints. Arthritis Rheum. 1996;**39**:308-20.

6- Zhang Y, Niu J, Kelly-Hayes M, Chaisson CE, Aliabadi P, Felson DT. Prevalence of symptomatic hand osteoarthritis and its impact on functional status among the elderly: The Framingham Study. Am J Epidemiol 2002;**156**:S225.

7- Dahaghin S, Bierma-Zeinstra SMA, Reijman M, Pols HAP, Hazes JMW, Koes BW. Prevalence and determinants of one month hand pain and hand related disability in the elderly (Rotterdam study). Ann Rheum Dis 2005;**64**:99-104.

8 Wittoek R, Vander Cruyssen B, Verbruggen G. Predictors of functional impairment and pain in erosive osteoarthritis of the interphalangeal joints: comparison with controlled inflammatory arthritis.Arthritis Rheum.

9. Leeb BF, Sautner J, Andel L, Rintelen B. A scale for assessment and quantification of chronic rheumatoid affections of the hands. Rheumatology 2003; 42: 1173-78.

10- Dillon CF, Hirsch R, Rasch E, Gu Q. Symptomatic hand osteoarthritis in the United States: prevalence and functional impairment estim ates from the third U.S. National Health and Nutrition Examination Survey, 1991-1994. Am J Phys Med Rehabil. 2007;86:12-21.

11- Kellgren JH. Osteoarthrosis in patients and populations. Br Med J. 1961;2:1-6.

12- Lawrence JS, Bremner JM, Biers F. Osteoarthritis. Prevalence in the population and relationship between symptoms and X-Ray changes. Ann Rheum Dis. 1966;25:1-24.

13- Bagge E, Bjelle A, Valkenburg HA, Svanborg A. Prevalence of radiographic osteoarthritis in two elderly European populations. Rheumatology Int. 1992;12:33-8.

14- Mannoni A, Briganti MP, Di Bari M, Ferrucci L, Constanzo S, Serni U, Masotti G, Marchionni N. Epidemiological profile of symptomatic osteoarthritis in older adults: a population based study in Dicomano, Italy. Ann Rheum Dis. 2003 Jun;62:576-8.

15- Cobby M, Cushnaghan J, Creamer P, Dieppe P, Watt I. Erosive osteoarthritis: is it a separate disease entity? Clinical Radiology 1990;**42**:258-63.

16- Cavasin F, Punzi L, Ramonda R, Pianon M, Oliviero F Sfriso P, Todesco S. Prevalence of erosive osteoarthritis of the hand in a population from Venetian area. Rheumatismo 2004;56:46-50.

17- Punzi L, Ramonda R, Sfriso P. Erosive osteoarthritis. Best Pract Res Clin Rheumatol 2004 ;18:739-58.

18- Poole J, Sayer AA, Hardy R, Wadsworth M, Kuh D, Cooper C. Patterns of interphalangeal hand joint involvement of osteoarthritis among men and women: a British cohort study. Arthritis Rheum. 2003 48:3371-6.

19. Kwok WY, Kloppenburg M, Rosendaal FR, van Meurs JB, Hofman A, Bierma-Zeinstra SMA. Erosive hand osteoarthritis: its prevalence and clinical impact in the general population and symptomatic hand osteoarthritis. Ann Rheum Dis 2011;70:1238-42.

20. Haugen IK, Englund M, Aliabadi P, Niu J, Clancy M, Kvien TK, Felson DT. Prevalence, incidence and progression of hand osteoarthritis in the general population: the Framingham Osteoarthritis Study. Ann Rheum Dis 2011;70:1581-6.
21. Verbruggen G, Wittoek R, Vander Cruyssen B et al. Morbid anatomy of 'erosive osteoarthritis' of the interphalangeal finger joints: an optimised scoring system to monitor disease progression in affected joints. Ann Rheum Dis. 2010;69:862-7.
22. Suda T, Takahashi N, Udagawa N et al. Modulation of osteoclast differentiation and function by the new members of the tumor necrosis factor receptor and ligand families. Endocr Rev.1999;20:345-57.
23. Kobayashi K, Takahashi N, Jimi E, et al. Tumor necrosis factor alpha stimulates osteoclast differentiation by a mechanism independent of the ODF/RANKL-RANK interaction. J Exp Med 2000;191:275-86.
24. Komine M, Kukita A, Kukita T et al. Tumor necrosis factor-alpha cooperates with receptor activator of nuclear factor kappaB ligand in generation of osteoclasts in stromal cell-depleted rat bone marrow cell culture. Bone 2001;28:474-83.
25. Wei S, Kitaura H, Zhou P et al. IL-1 mediates TNF-induced osteoclastogenesis. J Clin Invest 2005;115:282-90.
26- Zwerina J, Redlich K, Polzer K et al. TNF-induced structural joint damage is mediated by IL-1. Proc Natl Acad Sci U S A. 2007;104:11742-7.
27. Lefebvre V, Peeters-Joris C, Vaes G. Modulation by interleukin 1 and tumor necrosis factor alpha of production of collagenase, tissue inhibitor of metalloproteinases and collagen types in differentiated and dedifferentiated articular chondrocytes. Biochim Biophys Acta. 1990;1052:366-78.
28. Wittoek R, Carron P, Verbruggen G. Structural and inflammatory sonographic findings in erosive and non-erosive osteoarthritis of the interphalangeal finger joints. Ann Rheum Dis. 2010;69:2173-6.
29. Jans L, De Coninck T, Wittoek R et al. 3 T DCE-MRI assessment of synovitis of the interphalangeal joints in patients with erosive osteoarthritis for treatment response monitoring. Skeletal Radiol. 2013;42:255-60.
30- Bathon JM, Martin RW, Fleischmann RM, Tesser JR, Schiff MH, Keystone EC, Genovese MC, Chester Wasko M, Moreland LW, Weaver AL, Markenson J, Finck BK. A Comparison of Etanercept and Methotrexate in Patients with Early Rheumatoid Arthritis. NEJM. 2000; 343:1586-93.
31- Lipsky PE, van der Heijde DMFM, St. Clair EW, Furst DE, Breedveld FC, Kalden JR, Smolen JS, Weisman M, Emery P, Feldmann, Gregory R. Harriman GR, Maini RN. Infliximab and Methotrexate in the Treatment of Rheumatoid Arthritis. NEJM. 2000; 343:1594-602.
32. Alten R, Gram H, Joosten LA, van den Berg WB, Sieper J, Wassenberg S, Burmester G, van Riel P, Diaz-Lorente M, Bruin GJ, Woodworth TG, Rordorf C, Batard Y, Wright AM, Jung T. The human anti-IL-1 beta monoclonal antibody ACZ885 is effective in joint inflammation models in mice and in a proof-of-concept study in patients with rheumatoid arthritis. Arthritis Res Ther. 2008;10:R67.
33. Cohen SB, Dore RK, Lane NE, Ory PA, Peterfy CG, Sharp JT, van der Heijde D, Zhou L, Tsuji W, Newmark R; Denosumab Rheumatoid Arthritis Study Group. Denosumab treatment effects on structural damage, bone mineral density, and bone turnover in rheumatoid arthritis: a twelve-month, multicenter, randomized, double-blind, placebo-controlled, phase II clinical trial. Arthritis Rheum. 2008;58:1299-309.

34. Ishiguro N, Tanaka Y, Yamanaka H, Yoneda T, Ohira T, Okubo N, Genant HK, van der Heijde D and Takeuchi T. Consistent Inhibition of Bone Destruction By Denosumab in Important Subgroups of Japanese Patients with Rheumatoid Arthritis. Arthritis Rheumatol 2014; 66:11(Suppl): S831/Presented at ACR meeting 2014

35. Verbruggen G, Wittoek R, Cruyssen BV, Elewaut D. Tumour necrosis factor blockade for the treatment of erosive osteoarthritis of the interphalangeal finger joints: a double blind, randomised trial on structure modification. Ann Rheum Dis. 2012 Jun;71(6):891-8.

36. Prolia SmPC. Sections 4.4 & 4.838.

37. Bone HG, Chapurlat R, Brandi ML, Brown JP, Czerwinski E, Krieg MA, Mellström D, Radominski SC, Reginster JY, Resch H, Ivorra JA, Roux C, Vittinghoff E, Daizadeh NS, , Bradley MN, Franchimont N, Geller ML, Wagman RB, Cummings SR, Papapoulos S. The effect of three or six years of denosumab exposure in women with postmenopausal osteoporosis: results from the FREEDOM extension. Clin Endocrinol Metab. 2013;98:4483-92.

38. Papapoulos S, et al. Eight Years of Denosumab Treatment in Postmenopausal Women with Osteoporosis: Results From the First Five Years of the FREEDOM extension. WCO-IOF-ESCEO Congress 2014. Osteoporis Int 2014; 25 (Suppl 2):S118

39. Haugen IK, Østergaard M, Eshed I, McQueen FM, Bird P, Gandjbakhch F, Foltz V, Genant H, Peterfy C, Lillegraven S, Haavardsholm EA, Bøyesen P, Conaghan PG.3. Iterative development and reliability of the OMERACT hand osteoarthritis MRI scoring system.
J Rheumatol. 2014 Feb;41(2):386-91

40. http://eur-lex.europa.eu/LexUriServ/LexUriServ.do?uri=OJ:L:2005:091:0013:0019:en

12.Appendices

Appendix 1. Scoring systems

A. Categorical scoring system was proposed for the progressive radiographic changes in IP finger joint OA. These changes were characterized by complete loss of the joint space preceding or coinciding with the appearance of subchondral cysts eroding the entire subchondral plate. These erosive episodes subsided spontaneously and were followed by processes of repair.[28]

The anatomical phases in the evolution of IP finger joint OA are the following.

Normal ('N') joints: no signs of OA.

Stationary ('S') phase: classical appearance of OA. Small ossification centers and osteophytes are present at the joint margins. They can both increase in size and discrete narrowing of the joint space can occur.

Loss of joint space ('J' phase): after remaining for a variable time in the stationary phase, some joints (almost exclusively PIPs or DIPs) become destroyed. The joint space completely disappears within a relatively short period of time.

Erosive ('E') phase: concurrently with or shortly after the disappearance of the articular cartilage (J phase), the subchondral plate becomes eroded. The appearance is that of a pseudo-enlargement of an irregular joint space. Roentgenograms obtained at yearly intervals showed that changes in phases from 'S' over 'J' to 'E' could occur within one year. This destructive 'J' and 'E' phases are always followed by repair or remodeling.

Remodeling ('R') phase: new irregular sclerotic subchondral plates are formed, and in between these a new joint space becomes visible. Huge osteophytes are formed during this phase. No further evolution is seen in remodeled joints.

B. A quantitative radiographic scoring system, the Ghent University Scoring System, GUSS® [29], is a reliable method to score radiographic change over time in erosive IP OA and detects more progression over a shorter period of time than the classical scoring system. Erosive progression and signs of repair or remodeling are then scored by indicating the proportions of normal subchondral bone, subchondral plate and joint space over time.

The subchondral bone area. The proportions of the subchondral bone area with normal/abnormal-looking bone architecture were assessed in a quadrangle square of which the side equalled the width of the joint space. The joint space was positioned in the centre of this square (figure 2A). In this square, regions where osteolytic activity and remodelling caused a disarrangement of the trabecular pattern, as well as areas where a complete loss of the trabecular structure had occurred, are defined.
Identifiable osteolytic subchondral bone areas are marked on the radiographs and proportions of remaining intact subchondral bone will be calculated, considering the delineated IP joint area being the 100% value.

The subchondral bone plate. In an IP joint that had completely lost its joint space, an existing subchondral plate was defined as a regular radio-opaque linear structure within the position of the original joint space. When the joint space was still identifiable, the subchondral bone plate was identified as a regular linear radio-opaque bone margin flanking the joint space.

Identifiable linear subchondral plate structures were marked on the radiographic images and proportions of remaining subchondral bone plate were computed, considering a twofold joint space width being the 100% value (figure 2B).

The joint space was recognized as a radiotranslucent area bordered with two subchondral plates. Identifiable joint spaces were marked on the radiographic images. Proportions of remaining joint space were estimated as the proportion of the joint width, considering the total joint space width being the 100% value (figure 2B).

Computation of the changes in IP joints in "J", "E" and "E/R" phases. Pictures from the IP joints at three time points in the correct sequence will be read and used by the readers to evaluate the extent of the pathological changes in subchondral bone architecture, and to estimate the presence/absence of both subchondral bone plate and synovial joint space. Proportional changes in these three variables will be recorded. The sum of the three separate scorings constituted the total IP joint score. Equal weight will be attributed to each of the subdomains.

Appendix 2. Overall assessments

	admin dmab/plac	clinical assessm	safety	laboratory						CR hand	US hand	DXA	ECG
				PBC	serum chem	BTM	25OH Vit D	Ca++	preg test*				
SCREENING		X		X	X	X	X	X	X	X	X		X
BASELINE	X	X	X								X	X	
WEEK 6			X										
WEEK 12	X	X	X	X	X	X		X	X		X		
WEEK 24	X	X	X					X	X	X			
WEEK 36	X	X	X					X	X				
WEEK 48	X	X	X	X	X	X		X	X	X	X	X	
WEEK 60	X	X	X					X	X				
WEEK 72	X	X	X			X		X	X	X			
WEEK 84	X	X	X					X	X				
WEEK 96		X	X	X	X	X		X	X	X		X	
								* if appropriate					

dmab: denosumab; plac: placebo; PBC: peripheral blood cell count; chem: chemistry; BTM: bone turnover markers
preg: pregnancy - sticks to be provided by the rheumatology dept.; CR: conventional radiography; US: ultrasound;
MRI: magnetic resonace imaging; ECG: electrocardiogramDXA: dual energy X-ray absorptiomery

Basic Serum chemistry will include urea, creatinine, ASAT, ALAT, Albumin. Depending on the individual patient, additional parameters may be added.

W36 is the timing for the last IP dose in the blinded period. All patients will receive a denosumab injection at W48 after the assessment. This would be the first denosumab dose administered in the open label phase.

Study Protocol

RANKL-blockade for the treatment of erosive osteoarthritis (OA) of interphalangeal finger joints

Randomized, double blind, placebo-controlled study to evaluate the efficacy of denosumab 60mg sc every 3 months in patients with erosive osteoarthritis of the interphalangeal finger joints

Principal Investigators:
[redacted]

Dept. of Rheumatology – Ghent University Hospital

EudraCT number: 2015-003223-53
Protocolnumber: AGO/2015/008

[redacted]

[redacted]

[redacted]

Protocol Signature Page

Principal/Chief Investigator signature

I confirm that I have read and understood protocol version 4.0 17 October 2018. I agree to comply with the study protocol, the principals of GCP, research governance, clinical trial regulations and appropriate reporting requirements.

Signature.. Date....Oct 26, 2018........

Print name ......[redacted]...

Protocol synopsis

**Study Type**	Investigator Sponsored Study
**Funder**	Amgen
**Study Design**	This is a randomized, double blind placebo controlled one-site proof-of-concept study in subjects with erosive osteoarthritis (OA) of interphangeal (IP) finger joints.<br>A total of 100 subjects will be enrolled into the study: 48 weeks placebo controlled double-blind phase with denosumab 60 mg every 12 weeks, followed by a 48-week open-label phase in which all subjects will receive denosumab. Extension phase of 48-week denosumab 60 mg every 12 weeks.
**Investigational Therapy**	Denosumab 60 mg subcutaneous injection every 12 weeks. All subjects will receive Calcium/vit D supplementation.
**Efficacy Objectives**	The **primary objective** is to assess the effect of denosumab on the reduction of radiographic erosive progression using GUSS™ (Ghent University Score System).The **secondary objective** is to assess the effect of denosumab on the reduction of radiographic erosive progression as defined by diminishing the appearance of new erosive IP finger joints.<br>The **exploratory objective** is mainly to assess the effect of denosumab on clinical variables, as well as ultrasonography and DEXA parameters. To evaluate the benefit for the patient after an additional year of treatment.
**Main Endpoints**	**Primary Endpoint:** The change in the negative evolution of GUSS™ scores in the target IP joints from baseline to week 24**Other Endpoints:** 1) The change in the negative evolution of GUSS™ scores in the target IP joints from week 24 to week 48 and from baseline to week 48. 2) The number of patients that develop new erosive IP joints ('S/J' to 'E' phase joints) at 48 weeks; 3) The number of 'S/J' IP joints that develop 'E' phases at 48 weeks.<br>**Endpoints extension**: 1) Changes in the negative evolution in GUSS™ scores in the target IP joints from week 96 to week 144.<br>2) The number of 'E' IP joints that develop 'R' phases at 144 weeks.
**Hypothesis**	**The main hypothesis** is that the repeated administration of denosumab 60 mg Q3 months can lead to reduce structural damage in erosive hand OA.
**Study Sites**	1 site – the Ghent site
**Subjects**	100 subjects
**Enrolment**	18 months
**Main Eligibility Criteria**	Males and females ≥ 30 years of age, with hand erosive OA:<br>1) having suffered from transient inflammatory attacks of the IP finger joints

	2) showing at the time of enrolment inflammatory signs and at least one IP finger joint with the typical X-rays appearance of a ‘J’ or ‘E’ phase joint
**Study treatment Duration**	96 weeks followed by extension phase of 48 weeks

Table of Contents

- Protocol Title and Investigators
- Protocol signature page
- Protocol synopsis

1. Background and Rationale
1.1 Disease background
1.2 Denosumab
1.3 Rationale for study design
1.4 Hypotheses
2. Study Objectives and Endpoints
3. Experimental Plan
3.1 Study design and schematic
3.2 Number of sites
3.3 Number of subjects
3.4 Estimated study duration
4. Subject Eligibility
4.1 Inclusion criteria
4.2 Exclusion criteria
5. Treatment and Study Procedures
5.1 Investigational product
5.2 Reporting requirements for investigational product complaints
5.3 Concomitant therapy
5.4 Study procedures and schedule of assessments
6. Statistical and Analytical Plans
6.1 Efficacy analysis
6.2 Safety analysis
6.3 Determination of sample size
7. Adverse Events/Adverse Event reporting
7.1 Definitions
7.1.1. Adverse Event
7.1.2. Serious Adverse Event
7.1.3. Adverse Event Severity
7.1.4. Relationship to Study Drug
7.2. Adverse Event Reporting
8. Regulatory obligations
8.1Informed Consent
8.2 Independent Ethics Committee/Institutional Review Board
9. Documentation relating to the clinical trial- trial master file
10. Publication Policy
10. References
11. Appendices
Appendix 1. Scoring systems
Appendix 2. Overall assessments

1. Background and Rationale

1.1 Disease background

Erosive osteoarthritis (OA) of the interphalangeal (IP) finger joints is considered an inflammatory subset of osteoarthritis of the hand. Its inflammatory clinical presentation and destructive nature are unmistakable. [1,2,3,4,5] The cumulation of destructive changes in the IP joints eventually results in considerable disability.[6,7,8] There are no significant differences in hand function, stiffness and level of pain between patients with hand OA and rheumatoid arthritis. Scores for both patient groups differ significantly from those of healthy controls.[9]
Patients with erosive OA show more functional impairment and significantly more pain compared to patients with controlled inflammatory arthritis affecting the hands. The acquired structural damage of the IP joints due to destructive/reparative phenomena is the largest contributor to functional limitations.[8]
Radiological prevalence of moderate to severe hand OA is estimated to occur in 7.3% (2.65 million) US adults aged 60+ years.[10] Similar data have been reported in European countries.[7,11,12,13,14]

A significant proportion of these patients suffer from the erosive type of hand OA. In a prospective study of 500 consecutive patients attending a rheumatology clinic with symptomatic limb joint OA, 4.8% cases were identified with erosive IP joint OA.[15]

In a survey on the entire health district in the Venetian area, 2.2% out of 640 subjects aged 40+ years had erosive OA of their IP joints.[16] Mainly women in the perimenopauzal age were affected.[17]

Even higher prevalences were seen in a British cohort study[18] on 2.986 people[18]. Numbers in this study were based on clinics and the authors proposed that a proportion of their polyarticular cases were "inflammatory types of OA in association with erosions". This assumption was based on an earlier study where clinical examination was validated against hand radiography (Egger et al., J Rheumatol 1995;22:1509–13).
Though the proportions of "erosive IP OA" reported here were probably overrated, the prevalence of what is considered to be "erosive IP OA" in this 53 years of age population was twice as high in women (10,6%), compared to men (5,9%).
More recently, these data were confirmed in 2 large population studies where the prevalence of radiographic erosive IP OA in subjects over 55 years of age ranged between 5.0 and 9.9%.[19,20]. The prevalence for men was lower at 3.3%.

These studies showed that erosive type of hand OA occurred predominantly in women.
Haugen IK et al. et al.[20] defined erosive IP OA at a joint level as Kellgren/Lawrence ≥2 plus erosions. The authors reported a prevalence of erosive IP OA in women of 9,9%, 3 times as high as in men (3,3%). In essence, the Kwok W-Y et al. figures[19] agree with the data above.

Moreover, the Haugen IK et al.[20] reported that symptomatic OA was twice as high in women (15,9%), compared to men (8,2%). Symptomatic OA here was defined as Kellgren/Lawrence stage ≥2 plus pain/aching/stiffness.

From these epidemiological studies we can conclude that the incidence of erosive OA of the IP finger joints ranges from five to ten percent particularly in women.

The aggressive destructive nature of the erosive OA is only recognized late in the disease and the radiological image of the "exhausted" final phase mimics a robust OA. Therefore, the disease was hitherto regarded as a form of primary OA - a degenerative joint disease that is caused by biomechanical overload of the joint structures. There is so far no therapy sought or found for the structural changes in the articular tissues occurring during the course of so-called degenerative joint diseases. Thus, no therapeutic measures are available that act on underlying disease mechanisms and therefore slow down or halt the progression of tissue degradation in joints affected by erosive hand OA. The current standard treatment of care in these patients is limited to symptomatic therapy to reduce pain.

There is still lack of agreement concerning the nature and specificity of erosive IP joint OA. Obviously, in erosive IP OA an important bone resorption is noted in the subchondral bone of IP finger joints, this bone resorption is readily visualized on conventional radiographs (Figure 1). The osteolytic 'erosive' lesions result in the collaps of the subchondral plate which supports the overlaying articular cartilage.[5,21] This is compatible with a pathologic osteoclast activity supported by the effects of RANKL (Receptor Activator of Nuclear Factor kappa-β Ligand).[22] RANKL is a key driver of maturation and activation of osteoclasts in bone in health and disease.[22] In pathologic conditions, RANKL can be strongly induced in a variety of cell types including stromal cells under the influence of locally produced proinflammatory cytokines such TNFα [23,24] and IL-1β.[25,26]
At the same time, a resorption of articular cartilage of the affected IP joints is also noted. As a result, the joint space gradually disappears on X-rays. Likely key factors in this process are TNF and IL-1 which both have important catabolic effects on human chondrocytes.[27] Indeed, during the course of the disease inflammatory processes in the synovial membrane of IP finger joints could be visualized.[28,29] Cytokines release thereof will have important catabolic effects on the neighbouring chondrocytes.

Thus, similar as observed in other destructive processes noted in inflammatory rheumatic diseases, the **TNF→ IL-1→ RANKL-pathway** appears to be a key therapeutic target in erosive hand OA.

Blockade of these cytokines has shown to delay ongoing tissue destruction in murine arthritis and in rheumatoid arthritis in human. [30,31,32,33,34]

Recently, TNFα-blockade was shown to retard the progression of joint damage in erosive IP finger joint OA.[35]

Considering the analogies between rheumatoid arthritis and erosive IP OA in the metabolic pathways that mediate tissue destruction, and the lack of any structure modifying treatment option in the latter, a pilot study exploring the effects of Denosumab on ongoing tissue destruction in IP finger joint OA is proposed.

1.2 Denosumab

Denosumab (Amgen), is a fully human monoclonal antibody designed to inhibit RANKL (RANK Ligand). RANKL binds to RANK, which exists as a cell surface receptor molecule on “pre”-osteoclasts: precursors of osteoclasts.

Binding of RANKL to RANK acts as the primary signal for bone removal in normal physiological bone remodeling and in a number of pathological conditions, e.g. malignant tumors and bone metastasis.

Activation of RANK by RANKL promotes the maturation of pre-osteoclasts into osteoclasts. Denosumab inhibits osteoclasts’ maturation, function and survival by binding to and inhibiting RANKL. This mimics the natural action of osteoprotegerin, an endogenous RANKL inhibitor that presents with decreasing concentrations in patients who are suffering from osteoporosis. This protects bone from degradation, and helps to counter the progression of the disease.

Denosumab was approved by the EMA for use in postmenopausal women with osteoporosis at increased risk for fracture at the dose of 60 mg sc every 6 months (Prolia®), and for the prevention of skeletal-related events in patients with bone metastasis from solid tumors at the dose of 120 mg every 4 weeks (XGEVA®).
More recently, denosumab was shown to retard the progression of structural lesions in rheumatoid arthritis, an unapproved indication for the drug.[33,34] Its dosing and safety profile depended on the different medical conditions in which the drug was used. Patients with osteoporosis and rheumatoid arthritis received 60 mg and up to 180 mg injected SC, every 6 months, respectively.

Experience from clinical studies indicates that side effects depend on the dosage.

According to Prolia® Summary of Product Characteristics (SmPC) [36], pain in extremities and musculoskeletal pain (including back pain and joint pain) were among the most common adverse reactions.
In patients treated for osteoporosis a rare unwanted effect included low calcium levels, especially when in case of an impaired kidney function. Patients must therefore be adequately supplemented with calcium and vitamin D levels before starting and during denosumab therapy. In the postmarketing setting, rare cases of severe symptomatic hypocalcaemia have been reported. Clinical monitoring of calcium level is recommended before each dose and, in patients predisposed to hypocalcaemia, within two weeks after the initial dose.
There have been rare cases of atypical femoral fracture reported in association with Prolia.
Infections of the urinary and respiratory tracts were reported as well as cellulitis, ear infection and diverticulitis. The SmPC includes a Warning Statement regarding skin infections (predominantly cellulitis) leading to hospitalization. It has been proposed that this increase in infections under denosumab treatment might be connected to the role of RANKL in the immune system.
Cataracts, constipation, skin rashes and eczema were also seen.
Osteonecrosis of the jaw (ONJ) was reported rarely in Prolia osteoporosis clinical development program. Primarily, at the high dosages used in patients with bone metastases, similarly to bisphosphonates, denosumab appeared to be implicated in increasing the risk of osteonecrosis of the jaw (ONJ) especially following extraction of teeth or oral surgical procedures.

In the post-marketing setting, rare events of drug-related hypersensitivity, including rash, urticaria, facial swelling, erythema, and anaphylactic reactions have been reported.

In the FREEDOM extension study[37,38], with up to 8 years of denosumab 60 mg Q6M exposure, the incidence rates of adverse events did not increase over time.

Denosumab safety data were reported in RA phase 2 studies[33,34]. The safety profile appears to be consistent with that in patients with postmenopausal osteoporosis. Denosumab did not have an effect on RA disease activity, as measured by the ACR response criteria, the DAS28 scores, and the occurrence of RA flares.

1.3 Rationale for study design

In RA, the initial changes are seen in the synovium where inflammatory lymphomyeloid cells massively produce TNF, and secondarily, IL-1 and RANKL. These two cytokines are responsible for the invasion of the adjacent cartilage and bone by the inflamed and proliferative synovial pannus.

In erosive IP joint OA, the osteolytic changes in subchondral bone occur before or concurrently with resorption of cartilage. The primary drivers of the cartilage damage thus are these osteolytic processes in the subchondral bone area and the collapse of the subchondral bone plate. RANKL is the cytokine primarily responsible for this osteolytic (osteoclast) activity.

The enhanced osteoclast activity and tissue remodeling initially seen in arthritic IP joint bone is clearly illustrated in figure 1.

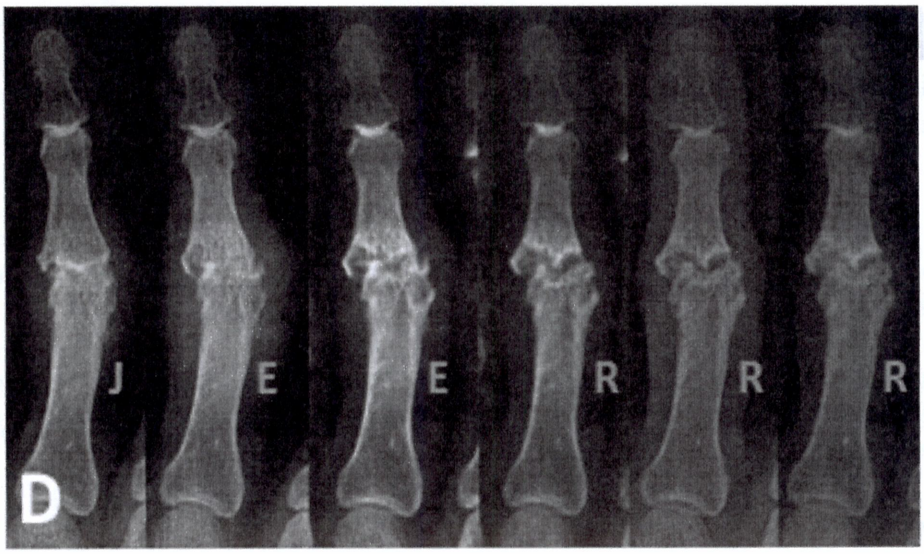

Figure 1: radiographic progression of a proximal IP joint from 'J' phase with loss of joint space to the 'E' phase with osteolytic activity in the subchondral bone area, and final remodeling of the destroyed tissues (R). Radiographs were taken with 6-months interval.

The effect of TNF alpha inhibitors on disease progression, previously seen in erosive IP joint OA[24], was an indirect effect on osteoclast activation. Obviously, this effect would be larger by directly inhibiting osteoclasts with Denosumab. Once the erosive process is blocked with Denosumab, subchondral bone remodeling will be inhibited and one should see preservation of joint structure.

A proof-of-concept study is proposed herein to test the ability of repeated administration of denosumab to control the structural damage– and thus to maintain hand function - in erosive hand OA. These tests will be conducted compared to placebo during a first placebo controlled double-blind phase but also in a second open-label phase in which all subjects will receive denosumab. The 2 main factors that support conducting this second open-label phase are the following:
- This would enable the Long-term outcome assessment with the cumulative exposure over time; more substantial effect would be expected.
- The open label with help supporting patients' engagement in a placebo trial where no disease modifying drugs exist.

The adequate dose of denosumab should completely inhibit the erosive process in order to fully test the hypothesis. In the phase 2 RA studies [33,34,], the higher dose or shorter interval dosing regimen showed an earlier or a trend to more inhibition of bone destruction respectively. Considering further the well-established safety profile for denosumab at high doses, a higher frequency for denosumab 60 mg is proposed: denosumab 60 mg sc every 3 months.

During previous studies an increased impact on the structural progression of the IP joints was shown over time in a subgroups of this population. Beside the one year placebo controlled phase followed by an open label phase, the extension phase will allow us to explore the benefit for the patient of one extra year of treatment. To compare the clinical benefits between study groups the treatment frequency cannot be interrupted. Approximately 50 patients, whom received the last injection of denosumab not more than 3 months prior to the inclusion in the extension phase can be included.

1.4 Hypotheses

The main hypothesis is that the repeated administration of denosumab 60 mg every 3 months in erosive hand OA can inhibit structural progression of already affected joints and prevent occurrence of newly affected joints.
As it has been shown that denosumab, reduces structural damage in RA while having no effect on clinical symptoms [34], no clinical benefit is expected within the one-year period of this study. So, the effects of denosumab on the clinical manifestations of the disease will only be part of an exploratory study.

2. Study Objectives and Endpoints

The objective of this proof of concept study is to investigate the efficacy of denosumab 60 mg sc every 12 weeks for 48 weeks as a therapeutic intervention in erosive IP joint OA. In general, the expected outcome of this study would be the control of the structural damage.

Changes in the architecture of the joint will be assessed by the GUSS™. This score system allows an overall score to be calculated for an affected IP joint over time. The overall score is the sum of scores obtained for 3 compartments of the IP finger joint: the synovial space (articular cartilage), the subchondral bone plates and the subchondral bone area at each side of the synovial space. Overall scores, as well as scores for each individual compartment can be taken into consideration. Examples of the calculated scores for 2 different IP joints are given in appendix 1.

The **primary objective** is to assess the effect of denosumab on the reduction of radiographic erosive progression using GUSS™ (Ghent University Score System).
The **primary endpoints of this objective** is the change in the negative evolution in GUSS™ scores in the target IP joints from baseline to week 24.
Other endpoints are the changes in the negative evolution of GUSS™ scores in the target IP joints from week 24 to week 48 and from baseline to week 48.
The **secondary objective** is to evaluate a reduction in radiographic erosive progression as defined by diminishing the appearance of new erosive IP finger joints.
This will be assessed by 2 endpoints:

1. the number of patients that develop new erosive IP joints ('S/J' to 'E' phases) at 48 weeks.
2. the number of 'S/J' IP joints that develop 'E' phases at 48 weeks.

Radiological score systems are given in appendix 1.

The **exploratory** objective is to assess if denosumab provides clinical benefits (improvement of pain and functional limitations) compared to placebo. We will also evaluate the impact on ultrasonography and DEXA.
The endpoints of this objective are:

1. Changes in clinical and patient recorded outcome measures from baseline (day 1) to week 48 after administration of denosumab compared to placebo. The following outcome measures will be recorded: AUSCAN (AUStralian CANadian Osteoarthritis Hand Index), FIHOA (Functional Index of Hand Osteoarthritis), Pain on VAS scale, consumption of analgesics (paracetamol)/NSAIDs to be recorded by each patient on a diary, tenderness upon pressure, diameter of selected target joints, and grip strength of both hands.
2. Changes in sonographic inflammatory signals at week 12 and 48 compared to baseline. Inflammatory changes will be assessed by measuring the amount of effusion and Power Doppler signal (scoring on a semi-quantitative scale).
3. Effect of denosumab on bone mass densitometry score in this group of patients compared to placebo from baseline to week 48. Changes from baseline (day 1) in T-score at lumbar spine and hip measured by bone densitometry at week 48 after administration of denosumab compared to placebo.

Other exploratory endpoints are to describe the above radiographic progression parameters at the end of the open-label phase. **Safety-objective**
The safety profile of denosumab 60 mg (Prolia®) every 6 months in postmenopausal women with osteoporosis at increased risk of fracture is well established (Prolia SmPC). This study will assess the safety of the administration of denosumab 60 mg every 3 months in the population of patients with erosive OA. Safety evaluations will be made by recording the incidence of AE/SAE (see also paragraph 8).

During the extension phase the objective is to explore the benefit of 1 year extra treatment.

The endpoints of this objective are:

1. Changes in the negative evolution in GUSS™ scores in the target IP joints from week 96 to week 144 and BL to week 144.
2. The number of 'E' IP joints that develop 'R' phases at 144 weeks.
3. Effect of denosumab on bone mass densitometry score in this group of patients compared from week 96 to week 144 and BL to week 144.

3. Experimental Plan

3.1 Study design and schematic

This is a randomized, double blind, placebo-controlled, one-site proof of concept study to investigate the effect of denosumab 60 mg every 12 weeks on the radiological evolution of erosive OA of the digital joints.
Two groups of 50 patients each will be enrolled in the study with a total treatment duration of 24 months (96 weeks): 48 weeks double-blind placebo controlled phase (denosumab (60 mg sc every 12 weeks or placebo) followed by a 48-weeks open–label phase in which all subjects will receive denosumab 60 mg every 12 weeks in an "Open Label Design" type study.
This 2 year study will be followed by an optional extension phase of 48 weeks.

Study schematic

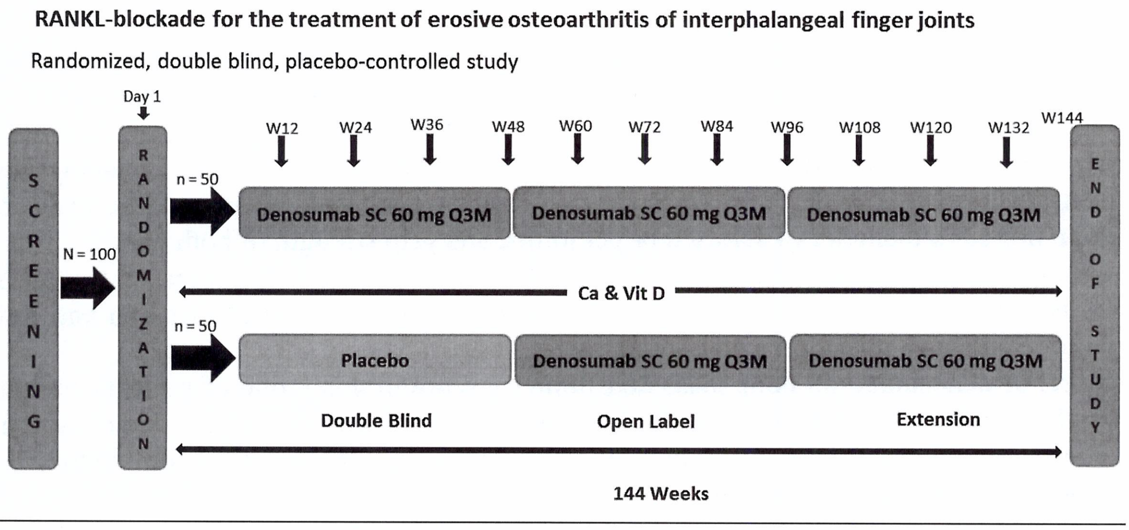

3.2 Number of sites

The study will be conducted in one site – the Ghent site in Belgium.

3.3 Number of subjects

A total of 100 subjects will be recruited in this study with an enrolment period of 18 months. Approximately 50 patients will qualify for the extension phase of the study.

3.4 Estimated study duration

The total treatment duration per subject is 24 months (96 weeks). The duration of the extension phase per subject is 12 months. The expected total trial duration defined as the time from first patient first visit to last patient last visit is 62 months.

4. Subject Eligibility

4.1 Inclusion criteria

A subject will be eligible for study participation if he/she meets the following criteria:

- Males and females ≥ 30 years of age.
- Subjects with hand OA having suffered from transient inflammatory attacks of the interphalangeal finger joints characteristic for what has been termed 'inflammatory' or 'erosive' hand OA.
- Subjects with hand OA showing inflammatory signs, either clinically or ultrasonographically, of the interphalangeal finger joints.
- Subjects with hand OA in which at least 1 interphalangeal finger joint has the typical appearance on the X-rays of a 'J' or 'E' phase joint as defined by the criteria mentioned above.
- Subjects with hand OA where at least 1 interphalangeal finger joint in the 'J' or 'E' phase presents a palpable swelling.
- Able and willing to give written informed consent and to comply with the requirements of the study protocol.

Inclusion criteria for the extension phase

- Subjects must have completed the 48 weeks of the randomised placebo-controlled study phase followed by the 48 weeks open label denosumab 60 mg SC every 3 months phase.
- Last injection of the investigational product denosumab was not more than 3 months prior to the inclusion in the extension phase.

4.2 Exclusion criteria

A subject will be excluded from the study if he/she meets any of the following criteria:

- Patients with known hypersensitivities to mammalian-derived drug preparations.
- Patients with clinically significant hypersensitivity to any of the components of Prolia.
- Current and/or Prior treatment with any investigational agent within 90 days, or five half-lives of the product, whichever is longer.
- Previous administration of denosumab from clinical trials or others (e.g. commercial use).
- Vitamin D deficiency [25(OH) vitamin D level < 20 ng/mL (< 49.9 nmol/L)]. Possibility of replenishment and re-screening.
- Subjects with current hypo- or hypercalcemia (normal serum calcium levels: 8.5-10.5 mg/dl or 2.12-2.62 mmol/L).
- Patients currently under bisphosphonate (BP) treatment or any use of oral BPs within 12 months of study enrollment or intravenous BPs or strontium ranelate within 5 years of study enrollment
- Prior use of any chondroprotective drug within 90 days e.g. chondroitin sulfate, glucosamine, avocado-soybean unsaponifiables, tetracyclins, corticosteroids (oral, intramuscular, intra-articular or intralesional).
- Prior use of any immunomodulating drug with possible effects on proinflammatory cytokine metabolism within 90 days a.o. corticosteroids (oral, intramuscular, intra-articular or intralesional), methotrexate, sulfasalazine, leflunomide, D-Penicillin, anti-malarials, cytotoxic drugs, TNF blocking agents.
- History of drug or alcohol abuse in the last year.

- Patients suffering from chronic inflammatory rheumatic disease (e.g. rheumatoid arthritis, spondylarthropathy, psoriatic arthritis, gout, chondrocalcinosis or other auto-immune diseases, e.g. systemic lupus erythematosus).
- History of cancer or lymphoproliferative disease within the past five years, other than a successfully and completely treated squamous cell or basal cell carcinoma of the skin or cervical dysplasia, with no recurrence within the last two years.
- History of any Solid Organ or Bone Marrow Transplant.
  - Comorbidities: significant renal function impairment (glomerular filtration < 30 ml/min/1.73m^2 or <50% of normal value), uncontrolled diabetes, unstable ischemic heart disease, congestive heart failure (NYHA III, IV), uncontrolled hypo or hyperparathyroidism, active inflammatory bowel disease, malabsorption, liver failure or chronic hepatic disease (serum AST/ALT levels 3 times above normal), recent stroke (within three months), chronic leg ulcer and any other condition (*e.g,.* indwelling urinary catheter) which, in the opinion of the investigator, would put the subject at risk by participation in the protocol.
- Subject has any kind of disorder that compromises the ability of the subject to give written informed consent and/or to comply with study procedures .
- Patient who is pregnant or planning pregnancy; if the female subject is of child-bearing age, she must use a valid mean of contraception during the study and for 9 months after last dose of study medication. For males with a partner of childbearing potential: subject refuses to use 1 effective methods of contraception for the duration of the study and for 10 months after the last dose of study medication.
- Female subjects who are breast-feeding.
- History of osteonecrosis of the jaw, and/or recent (within 3 months) tooth extraction or other unhealed dental surgery; or planned invasive dental work during the study.

5. Treatment and Study Procedures

5.1 Investigational product (see also paragraph 1.2)
The study drug used in this clinical trial is denosumab 60 mg subcutaneously every 3 months. It will be provided as sterile, solution for injection in 1 ml pre-filled syringes containing denosumab 60mg/ ml or placebo. Placebo for Denosumab will be presented in identical containers and stored/packaged the same as drug product denosumab. Denosumab prefilled syringe placebo product is supplied in a prefilled syringe as a sterile, single use, preservative free solution for subcutaneous injection. Each prefilled syringe contains 1 mL deliverable volume of buffer consisting of 10 mM sodium acetate, 5% (w/v) sorbitol, 0.01% (w/v) polysorbate 20, at a pH of 5.2. The IP is packed with 1 PFS per box. Both Denosumab and Placebo are manufactured by Amgen Inc, United States and released in the EU by Amgen Breda, Netherlands. Amgen will provide batch release certificates that will be made available with each shipment of the drug. Amgen will provide GMP certification and investigational medicinal product dossiers directly to the Belgian Agency in the regulatory submission by Amgen for this ISS. The injections will be given at the study site. Instructions for the drug handling, packaging and storage are provided in details below. Briefly, the drug will be given under the skin of the thigh, abdomen or upper arm. The clinical supplies should be stored in the refrigerator at 2-8°C. Do not freeze. Do not shake excessively. The clinical supplies must be protected from light by storing in the outer carton.

Patients who completed the 1-year interventional study will have the opportunity to enter a second 1-year open-label extension (OLE) study with Denosumab (60 mg every 12 weeks, SC). The 1-year radiographic progression of their IP finger joints will be monitored after 6 and 12 months of treatment in the OLE. After completion of the open label phase, patients will have the option to enter a second year extension with the same treatment. During this extension radiographic progression of their IP finger joints will also be monitored after 6 and 12 months of treatment.

Drug Handling:
"Denosumab is supplied as a sterile, colorless to slightly yellow, preservative-free solution for injection in a 1mL prefilled syringe (PFS). The formulation of IP is 60 mg/mL denosumab per mL, formulated with10 mM Sodium Acetate, 5% Sorbitol, 0.01% Polysorbate, to a pH of 5.2. Each PFS of IP is intended for single use only. The IP is packed with 1 PFS per box. Placebo for denosumab will be presented in identical containers and stored/packaged in the same way as drug product denosumab.

The IP is shipped by air courier maintained at 2°C to 8°C in a qualified shipper suitable for biological substance shipments. IP in a PFS will arrive in a secondary packaging container and should be immediately placed in a refrigerator maintained at 2°C to 8°C in a secured location until planned use. The set point for the refrigerator should be at 5°C.
IP must be properly labelled and dispensed in accordance with current ICH GCP and local/regional requirements prior to dispensing for administration.
Before preparation check that IP:

- is visually intact and suitable for use
- is not expired
- has not been subjected to any potential temperature excursion
- label of the box and vial is correct

Prior to administration, IP may be removed from the refrigerator and brought to room temperature (up to 25°C) in the original container. This generally takes 15 to 30 minutes. Do not warm IP in any other way. Once removed from the refrigerator, IP must not be exposed to temperatures above 25°C/77°F and must be used within 24 hours. If not used within this time duration, IP must be discarded. Do not freeze IP. Protect IP from light and heat. Avoid vigorous shaking. Preparation of the clinical supplies should be performed using aseptic techniques and under sterile conditions.

All SC injections must be administered by authorized site personnel. All subjects will receive 1 SC injection at each dosing visit (of either 60mg/ml Denosumab or Placebo) administered in the subject's upper arm, upper thigh or abdomen by a trained and qualified staff member. The injection should not be administered in the same arm from which blood is drawn."

5.2 Reporting requirements for investigational product complaints:
The following could be considered potential product complaints that need to be reported to Amgen. The Investigator will use a Product Complaint Form as provided by Amgen to report any complaint. Should any such concerns or irregularities occur, the IP will not be used until Amgen confirms that it is permissible to use. Examples of Product Complaints:
• Packaging: for example, broken container or cracked container

• Devices: issues with delivery of IP by device
• Usage: for example, subject or healthcare provider cannot appropriately use the product
• Labeling: for example, missing labels, illegible labels, incorrect labels, and/or suspect labels
• Change in IP appearance: for example color change or presence of foreign material
• Unexpected quantity in bottle: for example number of tablets or amount of fluid
• Evidence of tampering or stolen material

5.3 Concomitant therapy
All patients will have a daily calcium (1000 mg) and vitamin D (880 IU) supplementation. Subjects who are current or previous users of denosumab will be excluded at screening (see exclusion criteria).
Concomitant medication: NSAIDs and analgesics are allowed throughout during the study, but the dosages are kept constant during the first 12 weeks. Patients will keep records of their daily use of symptom modifying drugs.

5.4 Study procedures and schedule of assessments
A screening visit will include a clinical assessment, a hand radiograph and the laboratory investigations required. These will comprise a calcium and vitamin D status, peripheral blood cell count (PBC), serum chemistry glucose levels, liver (ALT, AST, alkaline phosphatase) and kidney function (serum ureum, serum creatinine, GFR) tests, Bone turnover markers (BTM) and, if appropriate, a pregnancy test.
An electrocardiogram (ECG) and an ultrasound (US) exam of the IP joints are part of the screening program.

Patients will be evaluated for risk factors for ONJ before starting treatment. A dental examination with appropriate preventive dentistry is recommended prior to treatment with Prolia in patients with concomitant risk factors.

The maximum window allowed between the screening visit and the baseline visit is of 3 weeks.

Upon selection, patients will be included in the study during **the baseline visit**, which will include a clinical examination and an ultrasound (US) exam of the IP joints. Magnetic resonance imaging (MRI[39]) of the hand is optional. Study products (denosumab/placebo) will then be administered on-site by the investigator/study nurse. Calcium and vit D supplementation will be installed. Dual energy X-ray absorptiometry (DXA).

Schedule of assessments are provided in detail as Appendix 2. Clinical assessment is the standard practice and will be detailed in the CRF and the SAP. Safety assessment is clarified in the safety paragraph.

At week 6: a clinical/safety evaluation is planned.

At week 12: clinical/safety assessment, PBC and serum chemistry, serum calcium levels and BTM, US. MRI of the hand is optional. Study products (denosumab/placebo) to be administered on-site by the investigator/study nurse.
At week 24: clinical/safety assessment, serum calcium levels, hand radiographs.
Study products (denosumab/placebo) to be administered on-site by the investigator/study nurse.
At week 36: clinical/safety assessment, serum calcium levels. Study products (denosumab/placebo) to be administered.
W36 is the timing for the last IP dose in the blinded period.
At week 48: clinical/safety assessment, US, hand radiographs, DXA. Serum calcium levels, PBC and serum chemistry (glucose levels, liver and kidney function tests, and BTM. Study products (denosumab/placebo) to be administered.

The visit at week 48 is the first visit of the Open Label Extension (OLE) program, which will encompass clinical/ safety exams, laboratory tests and hand radiographs as indicated in the table. The clinical monitoring of serum calcium during the OLE phase will follow the same schedule as in the placebo controlled phase.
All patients will receive a denosumab injection at W48 after the above assessment. This would be the first denosumab dose administered in the open label phase.

At week 96: clinical/safety assessment, hand radiographs, DXA. Serum calcium levels, PBC and serum chemistry (glucose levels, liver and kidney function tests, and BTM.

The visit at week 96 is the first visit of the Extension phase which will encompass clinical/ safety exams, laboratory tests and hand radiographs as indicated in the table. The clinical monitoring of serum calcium during the Extension phase will follow the same schedule as in the placebo controlled and OLE phase.

Safety: Patients will be able to report any unwanted effect during the regular visits and through telephone contact at any time in between these visits. Clinical examination is part of this safety assessment. Templates for AE/SAE recording created by the Investigators will be used.

As unwanted effects – other than these reported in previous Prolia osteoporosis programs - are not expected, the collection of other laboratory safety data beyond week 12 during the randomized treatment phase is not arranged.

A negative pregnancy test will be an entry requirement in female premenopausal patients. Premenopausal patients at risk to become pregnant will be excluded if no valid anti-conceptive method is used. In practice, premenopausal women will be an absolute minority in this study population. During the study and during the OLE phase, pregnancy tests will be done before each injection of denosumab in these subjects.

6. Statistical and Analytical Plans

6.1 Efficacy analysis

Complete and specific details of the final statistical analysis will be described and fully documented in the Statisticap Analysis Plan (SAP). The SAP will be finalized prior to the database lock. The analysis will be performed using the statistical software package IBM SPSS .

Demographic and baseline characteristics will be summarized. The number of observations, mean, standard deviation, median, minimum and maximum will be summarized for continuous variables. Discrete variables will be summarized by counts and percentages.

The primary efficacy variables will be the changes from baseline to week 24 in radiographic outcome measures, more specifically changes in GUSS. The primary efficacy comparisons will be between the denosumab treatment group and the placebo treatment group using GEE modelling with treatment as factors and baseline radiographic scores as a covariate. Additional endpoints will be assessed because several assumptions are made in this pilot study that are derived from a previous clinical study with a TNF-α blocking agent. The kinetics of TNF inhibitors might be different from the kinetics of denosumab on the bone level because of the different mode of action. Therefore it is not possible to predict if a similar rapid response on GUSS™ scores will be observed. Since the whole study is a proof-of-concept and to guarantee that a later response will not be missed, the study period needs to be extended to 48 weeks and the GUSS changes between week 24 and week 48, as well as GUSS changes between baseline and week 48 will be assessed.
Other analyses of radiographic measures will be the number of patients that develop new erosive joints and the number of patients in which erosive joints start the process of remodeling between baseline and 48 weeks. From previous studies it is known that the anatomical phase scoring system is not a sensitive on short term as GUSS.

Exploratory efficacy endpoints including change in Total AUSCAN score and individual subdomain (pain, physical function and stiffness) scores from baseline, change in FIHOA scores from baseline, change in pain scales (VAS pain) from baseline, change in consumption of analgesics (paracetamol)/NSAIDs, changes in number of painful and tender joints from baseline will be analyzed similarly at week 48. Other exploratory endpoints, including the change in number of joints with effusion and/or Power Doppler signal by ultrasound, the change in HOAMRIS scores and the changes in bone densitometry measures from baseline will be analyzed. Additional details will be provided in the SAP.

Primary and exploratory analyses will be repeated on subgroups defined by presence of soft tissue swelling at baseline. Details of analyses of efficacy endpoints at different time points as well as subgroups of interest will be given in the SAP.

The primary and exploratory efficacy variables will be analyzed on the intent-to-treat (ITT) population, defined as all subjects who were randomized. To evaluate the impact of major protocol violations on the results of the study, additional analyses of the primary efficacy analysis may be conducted on the per protocol population, which consists of all ITT subjects who completed the study and are not major protocol violators. The safety population consists of all subjects who received at least one dose of double-blind study medication.

In general, mean change analyses to compare the denosumab and placebo treatment group will be performed using GEE modelling with treatment group as factor and correction for baseline radiographic damage. Correction will be made for possible dependency between joints in the same patient by using an exchangeable matrix. Categorical data will be summarized using frequencies and percentages. Continuous data will be summarized with the number of non-missing observations by mean, standard deviation, median, maximum, and minimum values. In addition to the analyses based on observed data, analysis with imputed missing data will be conducted for selected efficacy variables. The details of such sensitivity analyses will be provided in the SAP. All statistical tests will be conducted at $\alpha = 0.05$ level (two-sided), unless otherwise stated. The last evaluation prior to the first study drug will be used as baseline for all analyses.

6.2 Safety analysis

Safety analyses will be carried out using the safety population, which includes all subjects who received at least one dose of study drug. Treatment-emergent AEs and SAEs will be summarized and reported. The number and percentage of subjects experiencing adverse events will be provided by system organ class and Medical Dictionary for Drug Regulatory Activities (MedDRA) preferred term. In addition, summary of AEs by severity and relationship to study drug will be presented. Serious, severe AEs, or AEs that lead to premature study discontinuation will be listed and described in detail. Mean change in vital signs and laboratory variables at each visit will be summarized for all treated subjects, and compared between treatment groups using one way Analysis of Variance (ANOVA).

6.3 Determination of Sample size

From a placebo controlled trial with adalimumab, we learned that, the risk that an individual IP joint evolves from J/S phase to the E phase is 2-3% per year. This risk increases to 15% for joints with a clinical effusion and to 25% for a painful joint with effusion. Adalimumab therapy reduced this risk for these inflammatory joints from 25% to 3% .

From these data 50 patients in each arm are needed to demonstrate a similar effect of denosumab with a power of 80%.
This power analysis took into account the following assumptions:

1) denosumab has a similar effect as adalimumab
2) a mean of minimal 1 inflamed joint (effusion and painful) per patient at baseline and in case of inclusion of patients with non-inflammatory joints, a within patient independent risk to evolve from J/S to E phase.
3) 5% drop-out
4) The proposed study involves two treatment arms. The level of significance (α) is 0.05.
5) a similar background risk for evolution from J/S to E phase.

Considering the semi-quantitative outcome measure, GUSS, a second power analysis was performed. Several assumptions were made, based on data from a previous study (Verbruggen G et al. ARD 2012;71(6):891-8). Power calculation was performed based on the estimated difference in the semi-quantitative outcome measure, GUSS ™ over time. This outcome measure is selected to detect the radiographic progression in the selected joints after treatment. The following assumptions were made:

- the natural progression (mean change) that can be expected over a period of 6 months is + 24 units (data from the placebo treated group), the mean difference in GUSS™ change between the placebo and adalimumab treated group after 6 months was 25 units. This was considered as clinically significant since
- the smallest detectable difference of GUSS™ was calculated as 40 units (Verbruggen G et al. ARD 2010;69(5):862-7) and improved to 10 units after intensive training.
- the standard deviation of the mean change in GUSS™ is 29,
- based on the above data, a total change of at least (24+ 25) 49 units in GUSS™ in the treatment group is considered to be a clinical relevant effect from a treatment.

The proposed study involves two treatment arms. The level of significance (α) is 0.05. From previous studies performed at our department, an drop out rate of 5% can be expected.
A sample size of 25 patients in each treatment arm will have 80% power to detect a difference in mean change GUSS™ of 25 units between the placebo and treated group, assuming that the standard deviation is 29 using a t-test with a two-sided 0.05 level of significance.
Taking into account a drop out rate of 5%, a total of 27 patients (25/ 1 – 0.05) should be included in each arm.
Taken into consideration both outcome measures, a minimum of 50 patients is required in both treatment arms in order to provide sufficient power for the study.

7. Adverse Events/Adverse Event reporting

The investigator will monitor each subject for clinical and laboratory (serum Ca^{++} levels) evidence of adverse events on a routine basis throughout the study. The investigator will assess and record any adverse event in detail on the adverse event DRF including the date and time of onset, description, seriousness severity, time course, duration and outcome, relationship of the adverse event to study drug, an alternate etiology for events not considered "probably related" to study drug, final diagnosis/syndrome (if known) and any action(s) taken. Adverse events, whether in response to a query, observed by study-site personnel, or reported spontaneously by the subject, will be recorded.

All adverse events will be followed to a satisfactory conclusion.

7.1 Definitions

7.1.1. Adverse Event

An **adverse event** is defined as any untoward medical occurrence in a subject or clinical investigation subject administered a pharmaceutical product and which does not necessarily have a causal relationship with this treatment. An adverse event can therefore be any unfavorable and unintended sign (including an abnormal laboratory finding), symptom, or disease temporally associated with the use of a medicinal product, whether or not the event is considered causally related to the use of the product.

Such an event can result from use of the drug as stipulated in the protocol or labeling, as well as from accidental or intentional overdose, drug abuse, or drug withdrawal. Any worsening of a pre-existing condition or illness is considered an adverse event. Laboratory abnormalities and changes in vital signs are considered to be adverse events only if they result in permanent

or temporary discontinuation of treatment with denosumab, necessitate therapeutic medical intervention and/or if the investigator considers them to be adverse events.

An elective surgery/procedure scheduled to occur during a study will not be considered an adverse event. However, if a pre-existing condition deteriorates unexpectedly during the trial (*e.g.,* surgery performed earlier than planned), then the deterioration of the condition for which the elective surgery/procedure is being done will be considered an adverse event.

7.1.2. Serious Adverse Event

If an adverse event meets any of the following criteria, it is to be considered as serious:

**Death of Subject**	An event that results in the death of a subject.
**Life-Threatening**	An event that, in the opinion of the investigator, would have resulted in immediate fatality if medical intervention had not been taken. This does not include an event that would have been fatal if it had occurred in a more severe form.
**Hospitalization**	An event that results in an admission to the hospital for any length of time. This does not include an emergency room visit or admission to an outpatient facility.
**Prolongation of Hospitalization**	An event that occurs while the study subject is hospitalized and prolongs the subject's hospital stay.
**Congenital Anomaly**	An anomaly detected at or after birth, or any anomaly that results in fetal loss.
**Persistent or Significant Disability/Incapacity**	An event that results in a condition that substantially interferes with the activities of daily living of a study subject. Disability is not intended to include experiences of relatively minor medical significance such as headache, nausea, vomiting, diarrhea, influenza, and accidental trauma (*e.g.*, sprained ankle).
**Important Medical Event Requiring Medical or Surgical Intervention to Prevent Serious Outcome**	An important medical event that may not be immediately life-threatening or result in death or hospitalization, but based on medical judgment may jeopardize the subject and may require medical or surgical intervention to prevent any of the outcomes listed above (*i.e.,* death of subject, life-threatening, hospitalization, prolongation of hospitalization, congenital anomaly, or persistent or significant disability/incapacity). Examples of such events include allergic bronchospasm requiring intensive treatment in an emergency room or at home, blood dyscrasias or convulsions that do not result in inpatient hospitalization, or the development of drug dependency or drug abuse.
**Spontaneous Abortion**	Miscarriage experienced by study subject.
**Elective Abortion**	Elective abortion performed on study subject.

7.1.3. Adverse Event Severity

The investigator will use the following definitions to define/rate the severity of each adverse event:

**Mild**	The adverse event is transient and easily tolerated by the subject.
**Moderate**	The adverse event causes the subject discomfort and interrupts the subject's usual activities.
**Severe**	The adverse event causes considerable interference with the subject's usual activities and may be incapacitating or life-threatening.

7.1.4. Relationship to Study Drug

The investigator will use the following definitions to assess the relationship of the adverse event to the use of study drug:

**Probably Related**	An adverse event has a strong temporal relationship to study drug or recurs on re-challenge and another etiology is unlikely or significantly less likely.
**Possibly Related**	An adverse event has a strong temporal relationship to the study drug and an alternative etiology is equally or less likely compared to the potential relationship to study drug.
**Probably Not Related**	An adverse event has little or no temporal relationship to the study drug and/or a more likely alternative etiology exists.
**Not Related**	An adverse event is due to an underlying or concurrent illness or effect of another drug and is not related to the study drug (*e.g.*, has no temporal relationship to study drug or has a much more likely alternative etiology).

7.2. Adverse Event Reporting

Reporting will be consistent with current safety reporting standards. .Adverse events will be reported between the first dose administration of trial medication and the last trial related activity.
All AEs and SAE's will be recorded in the patient's file and in the CRF. All SAE's will be reported as described below.
SAE's occurring within a period of 30 days following the last intake of trial medication will also be handled as such if spontaneously reported to the investigator.

All serious adverse events (SAE) and pregnancies occurring during clinical trials must be reported by the local Principal Investigator within 2 working days after becoming aware of the SAE to:

- The local EC
- Bimetra Clinics of the University Hospital Ghent

This reporting is done by using the appropriate SAE form. For the contact details, see below.

It is the responsibility of the local Principal Investigator to report the local SAE's to the local EC.

In case the investigator decides the SAE is a SUSAR (Suspected Unexpected Serious Adverse Reaction), Bimetra Clinics will report the SUSAR to the Central EC and the CA within the timelines as defined in national legislation.

In case of a life-threatening SUSAR the entire reporting process must be completed within 7 calendar days. In case of a non life-threatening SUSAR the reporting proces must completed within 15 calendar days.

The first report of a serious adverse event may be made by telephone, e-mail or facsimile (FAX).

Contact details of Bimetra Clinics:
e-mail: bimetra.clinics@uzgent.be
tel.: 09/332 05 00
fax: 09/332 05 20

In the event of a serious, unexpected and related adverse event, the investigator will report this to the Amgen Affiliate by faxing the appropriate adverse event form within 24 hours of being made aware of the serious adverse event and simultaneously to Bimetra Clinics who will report the event to the local regulatory agency within the timelines as defined in the national legislation..
Please fax SAE form to [redacted], Pharmacovigilance Manager : Fax number 0800 80877

The investigator must provide the minimal information: i.e. trial number, subject's initials and date of birth, medication code number, period of intake, nature of the adverse event and investigator's attribution.
This report of a serious adverse event by telephone must always be confirmed by a written, more detailed report. For this purpose the appropriate SAE form will be used. Pregnancies occurring during clinical trials are considered immediately reportable events. They must be reported as soon as possible using the same SAE form. The outcome of the pregnancy must also be reported.

If the subjects are not under 24-hour supervision of the investigator or his/her staff (out-patients, volunteers), they (or their designee, if appropriate) must be provided with a "trial card" indicating the name of the investigational product, the trial number, the investigator's name and a 24-hour emergency contact number.

8. Regulatory Obligations

8.1 Informed Consent

Signed informed consent will be obtained from the subject before any study procedures are undertaken, or before any medications are withheld from the subject in order to participate in this study. Subject may withdraw consent at any time without prejudice. All efforts will be made to continue the patient follow-up until the end of the study. At withdrawal, patients will be treated and assessed according to standard recommendations and as per latest guidance for contraception criteria in female subjects of child-bearing age or partners of childbearing potential (see exclusion criteria section 4.2).

8.2 Independent Ethics Committee/Institutional Review Board

The study will be declared at www.ClinicalTrials.gov and will comply with the principles of the Declaration of Helsinki. A copy of the study protocol will be submitted for approval to the ethical committee of Ghent University Hospital and to the Federal Agency for Medicines and Health Products *(FAGG; federal agentschap voor geneesmidddelen en gezondheidsproducten)*

9. Documentation relating to the clinical trial – Trial Master File

All documents related to the trial, e.g. study protocol, source documents, case report forms, … will be handled, stored and archived according to the EU Commission's Directive 2005/28/EC 63 Chapter 4.[40]

10. Publication Policy

The results of this study will be reported and published at conferences and in peer-reviewed clinical journals. Authorship publications will follow the Uniform Requirement for Manuscripts Submitted to Biomedical Journals (International Committee of Medical Journal Editors, 2009), which states:

Authorship credit should be based on (1) substantial contributions to conception and design, acquisition of data, or analysis and interpretation of data; (2) drafting the article or revising it critically for important intellectual content; (3) final approval of the version to be published and (4) Agreement to be accountable for all aspects of the work in ensuring that questions related to the accuracy or integrity of any part of the work are appropriately investigated and resolved. Authors should meet conditions 1, 2, 3 and 4.
For further details , see http://www.icmje.org/recommendations/browse/roles-and-responsibilities/defining-the-role-of-authors-and-contributors.html.

11.References

1- Stecher RM, Hauser H : Heberden's nodes. VII. The roentgenological and clinical appearance of degenerartive joint disease of the fingers. AmJ Roentgenol. 59 :326-337,1948
2- Crain DC : Interphalangeal osteoarthritis. Characterized by painful, inflammatory episodes resulting in deformity of the proximal and distal articulations. JAMA. 175: 1049-1053,1961
3- Peter JB, Pearson CM, Marmor L : Erosive arthritis of the hands. Arthritis Rheum. 9: 365-388,1966
4- Ehrlich GE. Osteoarthritis beginning with inflammation. Definitions and correlations. JAMA. 232: 157-159,1975
5- Verbruggen G and Veys EM. Numerical scoring systems for the anatomic evolution of osteoarthritis of the finger joints. Arthritis Rheum. 1996;**39**:308-20.
6- Zhang Y, Niu J, Kelly-Hayes M, Chaisson CE, Aliabadi P, Felson DT. Prevalence of symptomatic hand osteoarthritis and its impact on functional status among the elderly: The Framingham Study. Am J Epidemiol 2002;**156**:S225.
7- Dahaghin S, Bierma-Zeinstra SMA, Reijman M, Pols HAP, Hazes JMW, Koes BW. Prevalence and determinants of one month hand pain and hand related disability in the elderly (Rotterdam study). Ann Rheum Dis 2005;**64**:99-104.
8 Wittoek R, Vander Cruyssen B, Verbruggen G. Predictors of functional impairment and pain in erosive osteoarthritis of the interphalangeal joints: comparison with controlled inflammatory arthritis.Arthritis Rheum.
9. Leeb BF, Sautner J, Andel L, Rintelen B. A scale for assessment and quantification of chronic rheumatoid affections of the hands. Rheumatology 2003; 42: 1173-78.
10- Dillon CF, Hirsch R, Rasch E, Gu Q. Symptomatic hand osteoarthritis in the United States: prevalence and functional impairment estim ates from the third U.S. National Health and Nutrition Examination Survey, 1991-1994. Am J Phys Med Rehabil. 2007;86:12-21.
11- Kellgren JH. Osteoarthrosis in patients and populations. Br Med J. 1961;2:1-6.
12- Lawrence JS, Bremner JM, Biers F. Osteoarthritis. Prevalence in the population and relationship between symptoms and X-Ray changes. Ann Rheum Dis. 1966;25:1-24.
13- Bagge E, Bjelle A, Valkenburg HA, Svanborg A. Prevalence of radiographic osteoarthritis in two elderly European populations. Rheumatology Int. 1992;12:33-8.
14- Mannoni A, Briganti MP, Di Bari M, Ferrucci L, Constanzo S, Serni U, Masotti G, Marchionni N. Epidemiological profile of symptomatic osteoarthritis in older adults: a population based study in Dicomano, Italy. Ann Rheum Dis. 2003 Jun;62:576-8.
15- Cobby M, Cushnaghan J, Creamer P, Dieppe P, Watt I. Erosive osteoarthritis: is it a separate disease entity? Clinical Radiology 1990;**42**:258-63.
16- Cavasin F, Punzi L, Ramonda R, Pianon M, Oliviero F Sfriso P, Todesco S. Prevalence of erosive osteoarthritis of the hand in a population from Venetian area. Rheumatismo 2004;56:46-50.
17- Punzi L, Ramonda R, Sfriso P. Erosive osteoarthritis. Best Pract Res Clin Rheumatol 2004 ;18:739-58.
18- Poole J, Sayer AA, Hardy R, Wadsworth M, Kuh D, Cooper C. Patterns of interphalangeal hand joint involvement of osteoarthritis among men and women: a British cohort study. Arthritis Rheum. 2003 48:3371-6.
19. Kwok WY, Kloppenburg M, Rosendaal FR, van Meurs JB, Hofman A, Bierma-Zeinstra SMA. Erosive hand osteoarthritis: its prevalence and clinical impact in the general population and symptomatic hand osteoarthritis. Ann Rheum Dis 2011;70:1238-42.

20. Haugen IK, Englund M, Aliabadi P, Niu J, Clancy M, Kvien TK, Felson DT. Prevalence, incidence and progression of hand osteoarthritis in the general population: the Framingham Osteoarthritis Study. Ann Rheum Dis 2011;70:1581-6.
21. Verbruggen G, Wittoek R, Vander Cruyssen B et al. Morbid anatomy of 'erosive osteoarthritis' of the interphalangeal finger joints: an optimised scoring system to monitor disease progression in affected joints. Ann Rheum Dis. 2010;69:862-7.
22. Suda T, Takahashi N, Udagawa N et al. Modulation of osteoclast differentiation and function by the new members of the tumor necrosis factor receptor and ligand families. Endocr Rev.1999;20:345-57.
23. Kobayashi K, Takahashi N, Jimi E, et al. Tumor necrosis factor alpha stimulates osteoclast differentiation by a mechanism independent of the ODF/RANKL-RANK interaction. J Exp Med 2000;191:275-86.
24. Komine M, Kukita A, Kukita T et al. Tumor necrosis factor-alpha cooperates with receptor activator of nuclear factor kappaB ligand in generation of osteoclasts in stromal cell-depleted rat bone marrow cell culture. Bone 2001;28:474-83.
25. Wei S, Kitaura H, Zhou P et al. IL-1 mediates TNF-induced osteoclastogenesis. J Clin Invest 2005;115:282-90.
26- Zwerina J, Redlich K, Polzer K et al. TNF-induced structural joint damage is mediated by IL-1. Proc Natl Acad Sci U S A. 2007;104:11742-7.
27. Lefebvre V, Peeters-Joris C, Vaes G. Modulation by interleukin 1 and tumor necrosis factor alpha of production of collagenase, tissue inhibitor of metalloproteinases and collagen types in differentiated and dedifferentiated articular chondrocytes. Biochim Biophys Acta. 1990;1052:366-78.
28. Wittoek R, Carron P, Verbruggen G. Structural and inflammatory sonographic findings in erosive and non-erosive osteoarthritis of the interphalangeal finger joints. Ann Rheum Dis. 2010;69:2173-6.
29. Jans L, De Coninck T, Wittoek R et al. 3 T DCE-MRI assessment of synovitis of the interphalangeal joints in patients with erosive osteoarthritis for treatment response monitoring. Skeletal Radiol. 2013;42:255-60.
30- Bathon JM, Martin RW, Fleischmann RM, Tesser JR, Schiff MH, Keystone EC, Genovese MC, Chester Wasko M, Moreland LW, Weaver AL, Markenson J, Finck BK. A Comparison of Etanercept and Methotrexate in Patients with Early Rheumatoid Arthritis. NEJM. 2000; 343:1586-93.
31- Lipsky PE, van der Heijde DMFM, St. Clair EW, Furst DE, Breedveld FC, Kalden JR, Smolen JS, Weisman M, Emery P, Feldmann, Gregory R. Harriman GR, Maini RN. Infliximab and Methotrexate in the Treatment of Rheumatoid Arthritis. NEJM. 2000; 343:1594-602.
32. Alten R, Gram H, Joosten LA, van den Berg WB, Sieper J, Wassenberg S, Burmester G, van Riel P, Diaz-Lorente M, Bruin GJ, Woodworth TG, Rordorf C, Batard Y, Wright AM, Jung T. The human anti-IL-1 beta monoclonal antibody ACZ885 is effective in joint inflammation models in mice and in a proof-of-concept study in patients with rheumatoid arthritis. Arthritis Res Ther. 2008;10:R67.
33. Cohen SB, Dore RK, Lane NE, Ory PA, Peterfy CG, Sharp JT, van der Heijde D, Zhou L, Tsuji W, Newmark R; Denosumab Rheumatoid Arthritis Study Group. Denosumab treatment effects on structural damage, bone mineral density, and bone turnover in rheumatoid arthritis: a twelve-month, multicenter, randomized, double-blind, placebo-controlled, phase II clinical trial. Arthritis Rheum. 2008;58:1299-309.

34. Ishiguro N, Tanaka Y, Yamanaka H, Yoneda T, Ohira T, Okubo N, Genant HK, van der Heijde D and Takeuchi T. Consistent Inhibition of Bone Destruction By Denosumab in Important Subgroups of Japanese Patients with Rheumatoid Arthritis. Arthritis Rheumatol 2014; 66:11(Suppl): S831/Presented at ACR meeting 2014
35. Verbruggen G, Wittoek R, Cruyssen BV, Elewaut D. Tumour necrosis factor blockade for the treatment of erosive osteoarthritis of the interphalangeal finger joints: a double blind, randomised trial on structure modification. Ann Rheum Dis. 2012 Jun;71(6):891-8.
36. Prolia SmPC. Sections 4.4 & 4.838.
37. Bone HG, Chapurlat R, Brandi ML, Brown JP, Czerwinski E, Krieg MA, Mellström D, Radominski SC, Reginster JY, Resch H, Ivorra JA, Roux C, Vittinghoff E, Daizadeh NS, , Bradley MN, Franchimont N, Geller ML, Wagman RB, Cummings SR, Papapoulos S. The effect of three or six years of denosumab exposure in women with postmenopausal osteoporosis: results from the FREEDOM extension. Clin Endocrinol Metab. 2013;98:4483-92.
38. Papapoulos S, et al. Eight Years of Denosumab Treatment in Postmenopausal Women with Osteoporosis: Results From the First Five Years of the FREEDOM extension. WCO-IOF-ESCEO Congress 2014. Osteoporis Int 2014; 25 (Suppl 2):S118
39. Haugen IK, Østergaard M, Eshed I, McQueen FM, Bird P, Gandjbakhch F, Foltz V, Genant H, Peterfy C, Lillegraven S, Haavardsholm EA, Bøyesen P, Conaghan PG.3. Iterative development and reliability of the OMERACT hand osteoarthritis MRI scoring system. J Rheumatol. 2014 Feb;41(2):386-91
40. http://eur-lex.europa.eu/LexUriServ/LexUriServ.do?uri=OJ:L:2005:091:0013:0019:en

12.Appendices

Appendix 1. Scoring systems

A. Categorical scoring system was proposed for the progressive radiographic changes in IP finger joint OA. These changes were characterized by complete loss of the joint space preceding or coinciding with the appearance of subchondral cysts eroding the entire subchondral plate. These erosive episodes subsided spontaneously and were followed by processes of repair.[28]

The anatomical phases in the evolution of IP finger joint OA are the following.

Normal ('N') joints: no signs of OA.

Stationary ('S') phase: classical appearance of OA. Small ossification centers and osteophytes are present at the joint margins. They can both increase in size and discrete narrowing of the joint space can occur.

Loss of joint space ('J' phase): after remaining for a variable time in the stationary phase, some joints (almost exclusively PIPs or DIPs) become destroyed. The joint space completely disappears within a relatively short period of time.

Erosive ('E') phase: concurrently with or shortly after the disappearance of the articular cartilage (J phase), the subchondral plate becomes eroded. The appearance is that of a pseudo-enlargement of an irregular joint space. Roentgenograms obtained at yearly intervals showed that changes in phases from 'S' over 'J' to 'E' could occur within one year. This destructive 'J' and 'E' phases are always followed by repair or remodeling.

Remodeling ('R') phase: new irregular sclerotic subchondral plates are formed, and in between these a new joint space becomes visible. Huge osteophytes are formed during this phase. No further evolution is seen in remodeled joints.

B. A quantitative radiographic scoring system, the Ghent University Scoring System, GUSS® [29], is a reliable method to score radiographic change over time in erosive IP OA and detects more progression over a shorter period of time than the classical scoring system. Erosive progression and signs of repair or remodeling are then scored by indicating the proportions of normal subchondral bone, subchondral plate and joint space over time.

The subchondral bone area. The proportions of the subchondral bone area with normal/abnormal-looking bone architecture were assessed in a quadrangle square of which the side equalled the width of the joint space. The joint space was positioned in the centre of this square (figure 2A). In this square, regions where osteolytic activity and remodelling caused a disarrangement of the trabecular pattern, as well as areas where a complete loss of the trabecular structure had occurred, are defined.
Identifiable osteolytic subchondral bone areas are marked on the radiographs and proportions of remaining intact subchondral bone will be calculated, considering the delineated IP joint area being the 100% value.

The subchondral bone plate. In an IP joint that had completely lost its joint space, an existing subchondral plate was defined as a regular radio-opaque linear structure within the position of the original joint space. When the joint space was still identifiable, the subchondral bone plate was identified as a regular linear radio-opaque bone margin flanking the joint space.

Identifiable linear subchondral plate structures were marked on the radiographic images and proportions of remaining subchondral bone plate were computed, considering a twofold joint space width being the 100% value (figure 2B).

The joint space was recognized as a radiotranslucent area bordered with two subchondral plates. Identifiable joint spaces were marked on the radiographic images. Proportions of remaining joint space were estimated as the proportion of the joint width, considering the total joint space width being the 100% value (figure 2B).

Computation of the changes in IP joints in "J", "E" and "E/R" phases. Pictures from the IP joints at three time points in the correct sequence will be read and used by the readers to evaluate the extent of the pathological changes in subchondral bone architecture, and to estimate the presence/absence of both subchondral bone plate and synovial joint space. Proportional changes in these three variables will be recorded. The sum of the three separate scorings constituted the total IP joint score. Equal weight will be attributed to each of the subdomains.

Appendix 2. Overall assessments

	admin dmab/plac	clinical assessm	safety	laboratory						CR hand	US hand	DXA	ECG
				PBC	serum chem	BTM	25OH Vit D	Ca++	preg test*				
SCREENING		X		X	X	X	X	X	X	X	X		X
BASELINE	X	X	X								X	X	
WEEK 6			X										
WEEK 12	X	X	X	X	X	X		X	X		X		
WEEK 24	X	X	X					X	X	X			
WEEK 36	X	X	X					X	X				
WEEK 48	X	X	X	X	X	X		X	X	X	X	X	
WEEK 60	X	X	X					X	X				
WEEK 72	X	X	X			X		X	X	X			
WEEK 84	X	X	X					X	X				
WEEK 96	X	X	X	X	X	X		X	X	X		X	
WEEK 108	X	X	X					X	X				
WEEK 120	X	X	X			X		X	X	X			
WEEK 132	X	X	X					X	X				
WEEK 144		X	X	X	X	X		X	X	X		X	
								* if appropriate					

dmab: denosumab; plac: placebo; PBC: peripheral blood cell count; chem: chemistry; BTM: bone turnover markers
preg: pregnancy - sticks to be provided by the rheumatology dept.; CR: conventional radiography; US: ultrasound;
MRI: magnetic resonace imaging; ECG: electrocardiogramDXA: dual energy X-ray absorptiomery

Basic Serum chemistry will include urea, creatinine, ASAT, ALAT, Albumin. Depending on the individual patient, additional parameters may be added.

W36 is the timing for the last IP dose in the blinded period. All patients will receive a denosumab injection at W48 after the assessment. This would be the first denosumab dose administered in the open label phase.

STATISTICAL ANALYSIS PLAN

RANKL-blockade for the treatment of erosive osteoarthritis (OA) of interphalangeal finger joints.
Randomized, double blind, placebo-controlled study to evaluate the efficacy of denosumab 60mg sc every 3 months in patients with erosive osteoarthritis of the interphalangeal finger joints

EudraCT nummer: 2015-003223-53
EC nummer: 2015/0958

Author: [redacted]
Version 1 dd. 29/03/2020

Content:

1. Study identification
1.1 Study details
1.2 SAP details

2. Background and rationale of the study
2.1 Background
2.2 Objectives and hypotheses
2.3 Study type
2.4 Randomization details and masking
2.5 Sample size calculation

3. Analyses
3.1 Timing of final analysis

4. Study population
4.1 Inclusion and exclusion criteria
  4.1.1 Inclusion criteria
  4.1.2 Exclusion criteria
4.2 Data sets analyzed
  4.2.1 Safety Set
  4.2.2 Full Analysis Set
  4.2.3. Per Protocol Set
4.3 Protocol violations
4.4 Presentation of withdrawal and handling of missing data

5. Outcome measures
5.1 Primary endpoints
  5.1.1 Definition
  5.1.2 Target joints
  5.1.3 Reliability exercise
  5.1.4 Final and consensus scores
5.2 Secondary radiographic endpoints and outcome measures
  5.2.1 Definition
  5.2.2 Target joints
  5.2.3 Reliability exercise
  5.2.4 Final and consensus scores
5.3 Secondary clinical endpoints and outcome measures
  5.3.1 Clinical changes and patient reported outcome measures
  5.3.2 Sonographic changes
5.4. Exploratory endpoints
  5.4.1 DEXA changes
  5.4.2 Post hoc exploratory radiographic endpoints
5.5 Summary of endpoints

6. Statistical analysis
6.1 Summary of baseline data and flow of participants
6.2 Analysis of safety data
6.3 Primary outcome analysis
6.4 Secondary outcome analysis
    6.4.1 Secondary outcome analysis for continuous outcome measures
    6.4.2 Secondary outcome analysis for categorical outcome measures
6.5. Subgroup Analysis
6.6 Descriptive analysis
6.7 Sensitivity Analyses
6.8 Adjustment for multiplicity

7. Safety data

1. Study identification

1.1 Study details

Title: RANKL-blockade for the treatment of erosive osteoarthritis (OA) of interphalangeal finger joints: a Randomized, double blind, placebo-controlled study to evaluate the efficacy of denosumab 60mg sc every 3 months in patients with erosive osteoarthritis of the interphalangeal finger joints

Trial registration number: EudractNr: 2015-003223-53
EC approval number: EC nummer: 2015/0958
Principal Investigators: [redacted]
Amgen Reference Number 20149056

Protocol: final protocol version 1.0 dd 12/08/2015 – Last update version 4.0 17/10/2018

1.2 SAP details
SAP author: [redacted]
Responsibility in the trial: PI, statistical analysis, blinded sonographer
Senior statistician: [redacted]

2. Background and rationale of the study

2.1 Background

Erosive osteoarthritis (OA) of the interphalangeal (IP) finger joints is considered an inflammatory subset of osteoarthritis of the hand. Its inflammatory clinical presentation and destructive nature are unmistakable. The accumulation of destructive changes in the IP joints eventually results in considerable disability. Hitherto, no therapeutic interventions are available that act on underlying disease mechanisms and therefore slow down the structural progression in erosive hand OA. The current standard treatment of care in these patients is limited to symptomatic therapy to reduce pain.

In erosive hand OA, bone resorption at the subchondral bone of IP finger joints is a part of the pathogenetic process. This bone resorption is clearly seen on conventional radiographs (CR). Moreover, other features of structural damage can be seen on CR: joint space narrowing, subchondral sclerosis and development of bony proliferation or osteophytes. The osteolytic ‘erosive’ lesions do characterize erosive hand OA compared to the more common type or non-erosive type of hand OA. This bone resorption induces the collapse of the subchondral plate, normally supporting the overlaying articular cartilage. This is compatible with a pathologic osteoclastic activity supported by the effects of Receptor Activator of Nuclear Factor kappa-β Ligand (RANKL). RANKL is a key driver of maturation and activation of osteoclasts in bone in health and disease. In pathologic conditions, RANKL can be strongly induced in a variety of cell types including stromal cells under the influence of locally produced proinflammatory cytokines such Tumour necrosis factor alpha (TNFα) and Interleukin (IL) 1β.
At the same time, resorption of articular cartilage of the affected IP joints develops. Consequently, the joint space narrowing appears on CR. Key factors in this process are likely to be TNFα and IL-1β which both have important catabolic effects on human chondrocytes. During the course of the disease, inflammatory processes in the synovial

membrane of IP finger joints are clearly seen. Hence, release of cytokines will induce important catabolic effects on the neighbouring chondrocytes.

Thus, similar as observed in other destructive processes noted in inflammatory rheumatic diseases, the TNFα/IL-1β/RANKL-pathway appears to be a key therapeutic target in erosive hand OA.

2.2 Objectives and hypotheses

We hypothesize that treatment with denosumab, RANKL inhibition in erosive IP OA can reduce catabolic osteoclastic activity or structural erosive progression in already affected joints and prevent development of new erosive joints. Moreover, through inhibition of the RANKL pathway, inflammation can be suppressed. By suppressing inflammation and structural progression, beneficial effect on pain experience and functional impairment might follow.

The **primary objective** of the study is to assess the effect of denosumab on the reduction of radiographic erosive progression using GUSS™ (Ghent University Score System) from baseline to week 24.
The **secondary radiographic objective** is to evaluate a reduction in radiographic erosive progression as defined by diminishing the appearance of new erosive IP finger joints from baseline to week 24 and week 48, and between week 24 and week 48.
Secondary clinical objectives are to assess if denosumab provides clinical benefits (improvement of pain, functional limitations (FIHOA and AUSCAN), swollen and tender joint count and patient global assessment of efficacy, and grip strength) compared to placebo at all timepoints, and to assess the effect of denosumab compared to placebo on sonographic features at week 12 and week 48 from baseline. **Exploratory objectives** are to study DEXA changes and mean erosive and remodeling scores by GUSS.

2.3 Study type

A randomized, double blind, placebo-controlled phase 2 clinical trial

2.4 Randomization details and masking

After screening, randomization to placebo or denosumab is randomly (1:1) done by use of a block randomization scheme with a fixed block size of four. The randomization list is generated by a co-worker independent of the study and not being involved in any procedure during the study. Study medication is provided by the pharmacy department. Medication and placebo syringes are identical in appearance and smell. Patients, outcome assessors (GV, RW) and data analyst (RW) retain masked for treatment allocation until lock of the study database.

2.5 Sample size calculation

Sample size calculation was performed based on the estimated difference in GUSS™ over time (Verbruggen G et al. ARD 2010;69(5):862-7). Several assumptions were made, based on data from a previous clinical trial (Verbruggen G et al. ARD 2012;71(6):891-8).
The following assumptions were made:
- the natural progression (mean change) that can be expected over a period of 6 months is

≥ 24 units (data from the placebo treated group),
- the mean difference in GUSS™ change
between the placebo and adalimumab treated group after 6 months was ≥20 units. This was considered as clinically significant since :
1. the smallest detectable difference of GUSS™ was calculated to be 40 units (Verbruggen G. et al. ARD 2010;69(5):862-7) and improved to 10 units after intensive training.
2. the standard deviation of the mean change in GUSS™ is 29,
3. based on the above data, a total change of at least 20 units in GUSS™ in the treatment group is considered to be a clinical relevant effect from a treatment.

The proposed study involves two treatment arms. The level of significance (α) is 0.05.
A sample size of 46 patients in each treatment arm will have 90% power to detect a difference in mean change GUSS™ of 20 units between the placebo and treated group, assuming that the standard deviation is 29 using a t-test with a two-sided 0.05 level of significance.
Taking into account an attrition rate of 8%, a total of 50 patients (46/(1 – 0.08)) should be included in each arm.
From a placebo controlled trial with adalimumab, we learned that, the risk that an individual IP joint evolves from J/S phase to the E phase is 2-3% per year. This risk increases to 15% for joints with a clinical effusion and to 25% for a painful joint with effusion (non-published data). Adalimumab therapy reduced this risk for these inflammatory joints from 25% to 3% (Verbruggen G et al. ARD 2012;71(6):891-8).

Fifty patients in each arm are needed to demonstrate a similar effect of denosumab with a power of 80%.
This sample size analysis took into account the following assumptions :

1) denosumab has a similar effect as adalimumab
2) presence of at least 1 inflamed joint (defined by clinical and sonographic effusion and presence of pain) per patient at baseline.
3) 5% drop-out
4) The proposed study involves two treatment arms. The level of significance (α) is 0.05.
5) a similar background risk for evolution from J/S to E phase.

Taken into consideration both outcome measures, a minimum of 50 patients is required in both treatment arms in order to provide sufficient power for the study.

3. Analyses

3.1 Timing of final analysis

The SAP will be finalized prior to the database lock. The last visit of the last patient was foreseen in June 2019.
The SAP will be finished by 30th of March 2020. Data lock will be done after 1st of April 2020. Statistical analyses will be performed from 2nd of April till approx. end of June 2020. Report to be expected End of September 2020.

4. Study population

4.1 Inclusion and exclusion criteria

Cfr Protocol (cfr final version 4.0 17/10/2018)

4.1.1 Inclusion criteria

A subject will be eligible for study participation if he/she meets the following criteria:

- Males and females ≥ 30 years of age.
- Subjects with hand OA having suffered from transient inflammatory attacks of the interphalangeal finger joints characteristic for what has been termed 'inflammatory' or 'erosive' hand OA.
- Subjects with hand OA showing inflammatory signs, either clinically or ultrasonographically, of the interphalangeal finger joints.
- Subjects with hand OA in which at least 1 interphalangeal finger joint has the typical appearance on the X-rays of a 'J' or 'E' phase joint as defined by the Verbruggen and Veys radiographic scoring system
- Subjects with hand OA where at least 1 interphalangeal finger joint in the 'J' or 'E' phase presents a palpable swelling.
- Able and willing to give written informed consent and to comply with the requirements of the study protocol.

4.1.2 Exclusion criteria

A subject will be excluded from the study if he/she meets any of the following criteria:

- Patients with known hypersensitivities to mammalian-derived drug preparations.
- Patients with clinically significant hypersensitivity to any of the components of Prolia.
- Current and/or Prior treatment with any investigational agent within 90 days, or five half-lives of the product, whichever is longer.
- Previous administration of denosumab from clinical trials or others (e.g. commercial use).
- Vitamin D deficiency [25(OH) vitamin D level < 20 ng/mL (< 49.9 nmol/L)]. Possibility of replenishment and re-screening.
- Subjects with current hypo- or hypercalcemia (normal serum calcium levels: 8.5-10.5 mg/dl or 2.12-2.62 mmol/L).
- Patients currently under bisphosphonate (BP) treatment or any use of oral BPs within 12 months of study enrollment or intravenous BPs or strontium ranelate within 5 years of study enrollment
- Prior use of any chondroprotective drug within 90 days e.g. chondroitin sulfate, glucosamine, avocado-soybean unsaponifiables, tetracyclins, corticosteroids (oral, intramuscular, intra-articular or intralesional).
- Prior use of any immunomodulating drug with possible effects on proinflammatory cytokine metabolism within 90 days a.o. corticosteroids (oral, intramuscular, intra-articular or intralesional), methotrexate, sulfasalazine, leflunomide, D-Penicillin, anti-malarials, cytotoxic drugs, TNF blocking agents.
- History of drug or alcohol abuse in the last year.

- Patients suffering from chronic inflammatory rheumatic disease (e.g. rheumatoid arthritis, spondylarthropathy, psoriatic arthritis, gout, chondrocalcinosis or other auto-immune diseases, e.g. systemic lupus erythematosus).
- History of cancer or lymphoproliferative disease within the past five years, other than a successfully and completely treated squamous cell or basal cell carcinoma of the skin or cervical dysplasia, with no recurrence within the last two years.
- History of any Solid Organ or Bone Marrow Transplant.
- Comorbidities: significant renal function impairment (glomerular filtration < 30 ml/min/1.73m^2 or <50% of normal value), uncontrolled diabetes, unstable ischemic heart disease, congestive heart failure (NYHA III, IV), uncontrolled hypo or hyperparathyroidism, active inflammatory bowel disease, malabsorption, liver failure or chronic hepatic disease (serum AST/ALT levels 3 times above normal), recent stroke (within three months), chronic leg ulcer and any other condition (e.g,. indwelling urinary catheter) which, in the opinion of the investigator, would put the subject at risk by participation in the protocol.
- Subject has any kind of disorder that compromises the ability of the subject to give written informed consent and/or to comply with study procedures .
- Patient who is pregnant or planning pregnancy; if the female subject is of child-bearing age, she must use a valid mean of contraception during the study and for 9 months after last dose of study medication. For males with a partner of childbearing potential: subject refuses to use 1 effective methods of contraception for the duration of the study and for 10 months after the last dose of study medication.
- Female subjects who are breast-feeding.
- History of osteonecrosis of the jaw, and/or recent (within 3 months) tooth extraction or other unhealed dental surgery; or planned invasive dental work during the study.

4.2 Data sets analyzed

4.2.1 Safety Set

The Safety Set includes all patients who receive at least 1 dose of denosumab or placebo. This set will be used to summarize demographics, baseline clinical characteristics and DEXA characteristics, adverse events, laboratory results, vital signs, and safety analysis.

4.2.2 Full Analysis Set

The Full Analysis Set (FAS) will include all patients who are randomly assigned to groups and received at least 1 dose of denosumab or placebo. Intention-to-treat analysis is performed on FAS. This analysis set will be used for efficacy analysis.

4.2.3. Per Protocol Set

The Per-Protocol (PP) Set is a subset of the FAS population, excluding patients with major protocol deviations and who do not complete the study until week 48. This population may be used to summarize efficacy.

The Safety Set, FAS and PP set will be identified prior to database lock.

4.3 Protocol violations

Only major protocol violations thought to affect the valid assessment of the efficacy of the study drug will be considered. A study team, consisting of at least the Principal investigator, study physician and one independent clinician will review the case and make a judgement whether the patient should be excluded from the PP population. Whether a major protocol violation occurred with the consequence of being excluded from the PP population, will be decided before breaking the randomization code.

The following situations will be considered as major protocol violation:

- patients not fulfilling eligibility criteria
- patients having received prohibited concomitant medication
- patients with protocol-required procedure(s) not adhered to
- patients having received less than 50% of study medication according to schedule of study
- patients who withdrew or were withdrawn during the study
- collection of subjective data from patients after they were unblinded (but not withdrawn) during the study period due to medical reasons will not be used in analyses. Objective data will be used in case the study team remained blind to treatment allocation.

4.4 Presentation of withdrawal and handling of missing data

Reasons for early withdrawal from the first year of the study will be summarized.

Missing data will not be imputed for descriptive statistics. For analysis of primary endpoint, data available from all time points will be used in each analysis, accounting for within patient clustering effects by using GEE models. Missing value after the last available visit or questionnaire or assessment due to dropping out the study for any reason will be imputed. The predictors used for the imputation model will be: randomization group, baseline value and values at other time points available (measurements at week 24 will be used to impute measurements at week 48), presence of baseline inflammation (if analyses on ‘joint level’), baseline number of affected joints (S, J, E, E/R).

Surgically modified hand joints (arthrodesis or arthroplasty or amputation) at baseline will be considered missing for joint-specified outcomes (tenderness, soft swelling, radiography and ultrasound) at all time points.

5. Outcome measures

5.1 Primary endpoints

5.1.1 Definition

The primary efficacy endpoint is to assess the effect of denosumab on the reduction of radiographic erosive progression from baseline to week 24. The outcome measure being used is GUSS™ (Ghent University Score System). This scoring system is composed of 3 subdomain: subchondral plate, subchondral bone and joint space. Specific features referring to underlying pathology of the disease are being scored on a numerical scale from 0 to 100, with increments of 10. The maximum score refers to a normal joints or completely restored (i.e., non erosive) joint. A total score per joint is made by an equally weighted sum score of all 3 subdomains (min. 0; max. 300). Details of GUSS™ are described elsewhere and an educational atlas is available to consult by the readers (Verbruggen G. et al. ARD

2010;69(5):862-7). For good understanding, the scoring system can change in positive (i.e., more remodeling) or negative direction (i.e., more erosive progression). The smallest detectable difference is 40 units and improved to 10 units after intensive training: this implies that an absolute change over 40 (10) units on a total of 300 in one joint shows significant change over measurement error.

5.1.2 Target joints

Target joints, prone to response to therapy, either erosive progression, stable condition, or remodeling, are selected on baseline CR: upon selection of target joints, the following criteria needs to be fulfilled:

- **A joint in J or E phase, according the Verbruggen and Veys anatomical scoring system, on baseline Xrays**. According to inclusion criteria of the study, a study patient has at least one target joint upon inclusion. If several target joints are available, all are included for efficacy analysis.
- **Presence of inflammatory activity in the joint**, defined by presence of soft tissue swelling upon clinical examination and presence of sonographic swelling (either synovial proliferation or effusion) at baseline

5.1.3 Reliability exercise
Radiographs will be read independently by two trained assessors, blinded to randomization and clinical data. The radiographs will be read with knowledge of time sequence. First reliability analysis will be performed on the readings of the first 20 patients. If ICCs total GUSS™ scores do not exceed 0.80, retraining will be performed upon further reading. Another reliability analysis will be performed on the following 20 readings until ICCs total GUSS™ exceeds 0.80.
Each reader will perform a second reading of a series of images (first 20 patients) to calculate intra-reader reliability, minimal one month after and blinded to the previous reading.

5.1.4 Final and consensus scores
The final scores of radiographic scorings will be the agreement scores amongst the two assessors. In case of disagreement, a consensus score will be made. The consensus score will be made by the two assessors by discussing and reanalyzing the radiograph and joint of interest.

5.2 Secondary radiographic endpoints and outcome measures
5.2.1 Definition
The secondary efficacy endpoint is to assess the effect of denosumab on the development of new erosive joints from baseline to week 24 and week 48, and to assess the effect of denosumab on the erosive progression between week 24 and week 48. The number/ percentage of new erosive (defined as new J or E) joints, according to the Verbruggen and Veys anatomical scoring system amongst the baseline non-erosive or non-remodeled joints (i.e., only baseline N, S, J joints) in a patient in each treatment group at week 24 and 48, and between 24 and 48, will be determined.

5.2.2 Target joints
For analysis of anatomical phase scoring system, all DIP and PIP joints will be assessed and integrated for analysis.

5.2.3 Reliability exercise
Radiographs will be read independently by two trained assessors, blinded to randomization and clinical data. The radiographs will be read with knowledge of time sequence. First reliability analysis will be performed on the readings of the first 20 patients. If unweighted Kappa statistics of anatomical phase scoring system do not exceed 0.80, retraining will be performed upon further reading. Another reliability analysis will be performed on the following 20 readings until unweighted Kappa exceeds 0.80.
Each reader will perform a second reading of a series of images (first 20 patients) to calculate intra-reader reliability, minimal one month after and blinded to the previous reading.

5.2.4 Final and consensus scores
The final scores of radiographic scorings will be the agreement scores amongst the two assessors. In case of disagreement, a consensus score will be made. The consensus score will be made by the two assessors by discussing and reanalyzing the radiograph and joint of interest.

5.3 Secondary clinical endpoints and outcome measures
The secondary objectives of the study are to explore whether denosumab adds clinical benefit in terms of reduction of pain, number of tender and swollen joints, patient assessment of efficacy, grip strength and functional improvement. Also the effects of denosumab on ultrasonographic features will be assessed.

5.3.1 Clinical changes and patient reported outcome measures
Pain is scored on a numeric rating scale (NRS) from 0 to 10. Changes from baseline to week 24 and week 48 will be calculated.
Functional ability is assessed by two questionnaire for functional impairment, AUSCAN and FIHOA at all visits. AUSCAN is a questionnaire consisting of three domains (pain, stiffness, function) with 15 questions in total: questions are responded on a NRS (0-10). Sum scores range from 0 to 150. FIHOA is a questionnaire consisting of ten questions (NRS 0 – 3): total score ranges from 0 to 30. Number of tender and swollen joints upon clinical examination at PIP2-5 and DIP2-5 of both hands (absence (0)/presence (1), 0-16), mean grip strength of 3 attempts of the most affected hand (defined as number of target joints, in case equal between left and right, the dominant hand is considered), and patient global assessment of efficacy (0-10, NRS) are assessed at all visits. All these outcomes are numerical outcomes: changes from baseline to all visits will be calculated and compared between denosumab and placebo group.

5.3.2 Sonographic changes
Presence of 4 variables are scored at baseline, week 12 and week 48: effusion (0-3), synovial proliferation (0-3), PD signal (0-3) and presence of erosions (0/1) at PIP2-5 and DIP2-5. Sum scores on patient level are made for effusion (0-48), synovial proliferation

(0-48), synovitis (effusion plus synovial proliferation)(0-96), PD signal (0-48), and erosions (0-16). Change from baseline will be measured at week 12 and week 48.

5.4. Exploratory endpoints

5.4.1 DEXA changes

T-score and Z-score of bone mineral density (BMD) at femoral neck and lumbar spine are obtained from baseline and Week 48. Percentage of patients with low BMD suggestive of osteoporosis (T-score ≤-2.5), presence of osteopenia (-2.5 < T-score ≤ -1.5) and normal BMD (T-score >-1.5) will be calculated. Changes in percentage of the above categories and changes in absolute T-scores at week 48 from baseline will be calculated for femur and spine and compared between groups.

5.4.2 Post hoc exploratory radiographic endpoints

In order to extrapolate the scoring system on joint level to patient level, a mean progression score is calculated. The mean progression score is the absolute sum score of all negative changes (i.e. erosive progression) of all target joints divided by the number of target joints for that patient:

$$\text{Mean progression score} = \frac{\sum |\Delta\text{GUSS erosive progression of target joints}|}{n}$$

With Δ GUSS™ assessed between baseline and Week 24 and n = the number of target joints in a patient

Equally, the mean remodeling score per patient will be calculated and equals the absolute sum score of all positive changes (i.e., remodeling) of all target joints divided by the number of target joints for that patient:

$$\text{Mean remodeling score} = \frac{\sum |\Delta\text{GUSS remodeling of target joints}|}{n}$$

With Δ GUSS™ assessed between baseline to Week 24 and n = the number of target joints in a patient. This outcome measure will be an exploratory outcome measure.

Also the mean progression score and mean remodeling score by GUSS™ from baseline to week 48, and week 24 to week 48 will be calculated.

Target joints are defined here by presence of any GUSS changes in the joint throughout the study irrespective of the baseline anatomical score or inflammatory status (clinical swelling or US inflammation).

5.5 Summary of endpoints

Primary endpoint (continuous): GUSS™ (0-300) at week 24

Secondary endpoints:

- Percentage of new erosive joints (J/E) by Verbruggen and Veys amongst the baseline non-erosive or non-remodeled joints (i.e., only baseline N, S, J joints) in a patient
- GUSS™ at week 48 (0-300)
- NRS pain at week 24 (0- 10)
- NRS Patient global assessment of efficacy at week 24 (0-10)
- Functional Index of Hand OA (FIHOA) at week 24 (0-30)
- Australian/Canadian Hand OA index (AUSCAN) at week 24 (0-150)
- Number of tender joints at week 24 (0-16)
- Number of swollen joints at week 24 (0-16)
- Grip strength at week 24 (in kg)
- Ultrasound synovitis score at week 12 (0-96)
- Ultrasound synovial proliferation scores at week 12 (0-48)
- Ultrasound effusion score at week 12 (0 – 48)
- Ultrasound PD score at week at week 12 (0-48)
- Ultrasound erosion score at week 48 (0-16)
- Clinical endpoints (AUSCAN, FIHOA, NRS pain, NRS patient global, swollen joints count, tender joint count, grip strength) and ultrasound parameters at other time points: for clinical endpoints points (week 6, week 12, week 24, week 36), for ultrasound (week 48)
- GUSS™ from week 24 to week 48

Exploratory endpoints:

- DEXA (T- and Z-score at femur total, femoral neck, distal radius and lumbar spine) at week 48
- Percentage in categories according to BMD values (normal, osteopenia, osteoporosis) at week 48
- Mean progression score GUSS™ at week 24 and 48, and from week 24 to 48
- Mean remodeling score GUSS™ at week 24 and 48, and from week 24 to 48

6. Statistical analysis

All statistical calculations will be performed using R version 3.6.1, unless otherwise specified.

6.1 Summary of baseline data and flow of participants

A flow diagram will be produced, in accordance with the consort guidelines (http:/www.consort-statement.org/)

All continuous variables (both primary as secondary endpoints: GUSS™, NRS pain, NRS patient assessment efficacy, AUSCAN score, FIHOA score, grip strength, tender sum scores, swollen sum scores, sonographic sum scores) will be checked for normality. For continuous variables, summary statistics will include sample size (Number of patients, N),

mean and standard deviation (SD) in case of (approximate) normal distribution. Otherwise, median, minimum, maximum values, 25th/75th percentile will be presented. Frequency count and percentages will summarized for categorical variables. If not normal distributed variables, non-parametric testing will be performed.

All demographic, baseline data and medical history information will be summarized using Safety Set. Demographic variables will be studied (age, disease duration, sex, etc). Demographic and baseline characteristics will be compared between treatment groups. If unbalanced data, a sensitivity analysis will be performed for the primary analysis by adding the variables for which an unbalance was found as a covariate to the model.

6.2 Analysis of safety data
Analyses of safety data will be performed in the defined Safety Set (cfr. Paragraph 4.2).

6.3 Primary outcome analysis

Primary efficacy analyses will be performed in an intention to treat (ITT) approach on the FAS population.

Changes in GUSS will be analyzed at joint level with generalized estimating equations (GEE), accounting for within-patient clustering. Robust standard errors will be used and the working correlation structure specified exchangeable. Data from all available time points will be used. The independent variables included in the model are treatment group, visit number (categorical), interaction between treatment group and visit number, and the baseline value of the dependent variable (continuous).
If unbalances of demographic variables were found at baseline. A sensitivity analysis will be performed for the primary analysis by adding the variables for which an unbalance was found as a covariate to the model.

All efficacy analyses will be presented by a point estimate of the difference between the treatment groups, with a 95% confidence interval (95% CI) and the two-sided p-value. A p-value below 0.05 ($p<0.05$) will be considered statistically significant.

6.4 Secondary outcome analysis
All secondary outcomes are measured at the level of the patient.

6.4.1 Secondary outcome analysis for continuous outcome measures
Continuous endpoints will be analyzed with generalized estimating equations (GEE) using robust standard errors and the working correlation structure specified exchangeable. Data from all available time points will be used. The independent variables included in the model are treatment group, visit number (categorical), interaction between treatment group and visit number, and the baseline value of the dependent variable (continuous).

$$Y_{ij} = \beta_0 + \beta_1 * Treatment_i + \beta_2 * Baseline_i + \beta_3 * Visit_i + \beta_4 * Visit_i * Treatment_i + e_{ij}$$

Where

Y_{ij} = Outcome (continuous) of interest for patient i at visit j
i = patient
j = visit
β_0 = Intercept
β_1 = Regression coefficient for treatment (0 = placebo; 1 = Verum)
β_2 = Regression coefficient for baseline value of Y (continuous)
β_3 = Regression coefficient for visit (1 = week 6; 2 = week 12; 3 = week 24; 4 =week 36; 5 = week 48)
β_4 = regression coefficient for treatment-visit interaction

For estimation of the treatment effect after week 24, the model will include separate estimates of the treatment effect at 6, 12 and 24 weeks, with the coefficient corresponding to treatment*visit interaction term (β_4) of week 24 being the estimate of interest, with the corresponding p-value indicating statistical significance. Data of week 36 and 48 will not be included in the model for estimation of the treatment effect at week 24. For estimation of the treatment effect after week 36 and 48, the model will additionally include estimate(s) for treatment effect at week 36, and week 36 and 48, respectively.

Secondary efficacy analyses and exploratory endpoint analyses will be done in the PP population.

6.4.2 Secondary outcome analysis for categorical outcome measures
For the binary outcome measures (for example E joint yes/no) a GEE logistic regression for grouped binomial data will be applied. Odds ratios (OR), 95% confidence interval (95% C.I.) and p-value will be calculated by GEE model with treatment and time effect, to assess the odds for difference in new erosive progression from baseline between denosumab treated group and placebo group at week 24 and 48.

6.5. Subgroup Analysis
For the primary outcome measure, a subgroup analysis for inflammatory activity in the joint (Yes/No) is planned. The interaction between the presence of baseline inflammation and treatment effect on change in GUSS scores over 24 weeks will be tested.

6.6 Descriptive analysis
For the primary outcome measure, another descriptive efficacy analysis is performed on an extended group of target joints: all joints showing any progression to J, E or E/R phase throughout the study that were not defined J or E at baseline will be included to study the change in GUSS progression at week 24.

6.7 Sensitivity Analyses

For the primary outcome measure, sensitivity analyses will be performed:
- An adjusted analysis: including the unbalanced variables in the model
- A Per Protocol (PP) analysis.

6.8 Adjustment for multiplicity

As there is only one primary outcome, no adjustments for multiple testing will be performed.

7. Safety data

Safety will be assessed in all patients who randomly assigned to a treatment group and started the allocated intervention (Safety Set). The safety of denosumab will be assessed based on incidence and severity of adverse events (AEs), serious adverse events (SAEs), and changes from baseline through trial completion in routine clinical laboratory tests (Hb, white blood count, calcium, creatinine (GFR), AST, ALT, phosphor), and pregnancy tests for female patients of childbearing potential. The number of AEs, SAEs, withdrawal because of AEs and changes in biochemistry data between baseline and all time points for calcium, and baseline and week 12 and 48 for other lab data will be determined.

Statistical analysis plan as executed

Changes:

4.4 Presentation of withdrawal and handling of missing data

Missing data for secondary clinical endpoint analyses will be replaced by a conservative approach being the baseline observation carried forward method.

6.4.1 Secondary outcome analysis for continuous outcome measures

Secondary efficacy analyses and exploratory endpoint analyses will be done in the ITT population.

6.7 Sensitivity Analyses

For the primary outcome measure, two sensitivity analyses will be performed:

1. an adjusted analysis with a simple non-responder imputation, being the baseline observation carried forward approach in case of missing data
2. An adjusted analysis with a mixed effects repeated measures approach: a three-level linear mixed model with patient and joint as random effects and total GUSS at baseline, treatment, time (W24 and W48) and the interaction between time and treatment as fixed effects was applied.
