# Supplementary material for: RANKL blockade for erosive hand osteoarthritis: a randomized placebo-controlled phase 2a trial
Source: Nat Med. 2024 Feb 15;30(3):829–36. doi: 10.1038/s41591-024-02822-0 (PMC10957468; doi:10.1038/s41591-024-02822-0)
Supplement: Supplementary file 2 — Reporting Summary [file 41591_2024_2822_MOESM2_ESM.pdf]

nature portfolio

Corresponding author(s): Ruth Wittoek

Last updated by author(s): 2023/12/22

Reporting Summary

Nature Portfolio wishes to improve the reproducibility of the work that we publish. This form provides structure for consistency and transparency in reporting. For further information on Nature Portfolio policies, see our Editorial Policies and the Editorial Policy Checklist.

Statistics

For all statistical analyses, confirm that the following items are present in the figure legend, table legend, main text, or Methods section.

n/a	Confirmed	
☐	☒	The exact sample size (*n*) for each experimental group/condition, given as a discrete number and unit of measurement
☐	☒	A statement on whether measurements were taken from distinct samples or whether the same sample was measured repeatedly
☐	☒	The statistical test(s) used AND whether they are one- or two-sided<br>*Only common tests should be described solely by name; describe more complex techniques in the Methods section.*
☐	☒	A description of all covariates tested
☐	☒	A description of any assumptions or corrections, such as tests of normality and adjustment for multiple comparisons
☐	☒	A full description of the statistical parameters including central tendency (e.g. means) or other basic estimates (e.g. regression coefficient) AND variation (e.g. standard deviation) or associated estimates of uncertainty (e.g. confidence intervals)
☐	☒	For null hypothesis testing, the test statistic (e.g. *F*, *t*, *r*) with confidence intervals, effect sizes, degrees of freedom and *P* value noted<br>*Give P values as exact values whenever suitable.*
☒	☐	For Bayesian analysis, information on the choice of priors and Markov chain Monte Carlo settings
☒	☐	For hierarchical and complex designs, identification of the appropriate level for tests and full reporting of outcomes
☒	☐	Estimates of effect sizes (e.g. Cohen's *d*, Pearson's *r*), indicating how they were calculated

Our web collection on statistics for biologists contains articles on many of the points above.

Software and code

Policy information about availability of computer code

Data collection	No software was used
Data analysis	All statistical calculations were performed using R version 3.6.1 and IBM SPSS Statistics for Windows, version 25 (N.Y., USA).

For manuscripts utilizing custom algorithms or software that are central to the research but not yet described in published literature, software must be made available to editors and reviewers. We strongly encourage code deposition in a community repository (e.g. GitHub). See the Nature Portfolio guidelines for submitting code & software for further information.

Data

Policy information about availability of data

All manuscripts must include a data availability statement. This statement should provide the following information, where applicable:

- Accession codes, unique identifiers, or web links for publicly available datasets
- A description of any restrictions on data availability
- For clinical datasets or third party data, please ensure that the statement adheres to our policy

Deidentified raw data available as supplementary information. To the extent that current legislation allows it, the authors will provide access to additional individual deidentified participant-level data that underlie the data presented in this article to researchers who provide a methodologically sound proposal for academic purposes to interpret, verify and extend research in the article that does not violate intellectual property or confidentiality obligations, beginning 12 months after

article publication. Researchers should contact the corresponding author when applying for additional data access. Use of data will be restricted to the agreed purpose. Requests will be answered within 4 weeks. The study protocol with amendments and statistical analysis plan are available in the appendix.

Human research participants

Policy information about studies involving human research participants and Sex and Gender in Research.

Reporting on sex and gender	No sex nor gender based analyses were performed.Determination of sex by self-reporting. Findings do not apply to one sex or gender. The majority of patients included were females since this represents the epidemiology of a hand Osteoarthritis cohort.
Population characteristics	The study population was mainly female (78%). Mean age was around 61 years. Mean disease duration was 6.5 years and BMI (body mass index) indicated presence of slight overweight.
Recruitment	Patients were recruited from the outpatient clinic of the rheumatology department of the Ghent University hospital in Belgium. All patients fulfilling eligibility criteria were included after having provided informed consent. Patients were approached by a health care professional and asked if they would be interested in participating in a clinical trial. The motivation to enter the trial was asked to assess the degree of volunteer bias.
Ethics oversight	The study was approved by the ethics committee from the Ghent University Hospital. The members of the ethics committee that approved the study were: List of Members of the Ethics committee of the Ghent University Hospital that approved the study: D. MATTHYS (president), J. DECRUYENAERE (secretary), T. BALTHAZAR, W. CEELEN, K. DHONDT, C. DEMEESTERE, P. DERON, M. DE MUYNCK, G. DE SMET, S. JANSSENS, K. KINT, W. NOTEBAERT, M. PETROVIC, R. PIERS, R. RUBENS, P. SCHELSTRAETE, S. STERCKX, B. VANDERHAEGEN, Prof.dr. K. VANDEWOUDE, S. VERCOUTERE, F. VANDEKERCKHOVE, J. VAN ELSEN, K. VAN LIERDE

Note that full information on the approval of the study protocol must also be provided in the manuscript.

Field-specific reporting

Please select the one below that is the best fit for your research. If you are not sure, read the appropriate sections before making your selection.

☒ Life sciences ☐ Behavioural & social sciences ☐ Ecological, evolutionary & environmental sciences

For a reference copy of the document with all sections, see nature.com/documents/nr-reporting-summary-flat.pdf

Life sciences study design

All studies must disclose on these points even when the disclosure is negative.

Sample size	A sample size of 46 patients in each treatment arm was required to detect a difference in mean change GUSS of 20 units between the placebo and treated group at week 24 attaining a power of 90%, assuming that the standard deviation (SD) was 29 using a t-test with a two-sided 0.05 level of significance (α). Taking into account an attrition rate of 8%, 100 patients were included.
Data exclusions	No data were excluded
Replication	-The intra- and interrater reproducibility of radiographic readings (done independently by two experienced readers, unaware of clinical findings and treatment allocation) were found excellent (table 2). Data shown are intra-class coefficients of correlation (ICC) by two-way mixed, absolute agreement, average measures, or stated if otherwise for GUSS from the first 20 patients (accounting for 320 joints). Repeated readings were performed with an interval of minimally one month.<br>-The primary radiographic endpont (Ghent University scoring system, GUSS) was confirmed by another scoring system, the anatomical phase scoring system (secondary endpoint), which is a more robust way of showing the development of new (incident) erosions.
Randomization	Eligible patients were randomly assigned, in a 1:1 ratio, to receive in a blinded fashion denosumab (Amgen) or placebo during the placebo-controlled double-blind phase of the study, by use of a randomization scheme with a fixed block size of four. The randomization list was generated by a co-worker independent of the study and not involved in any procedure during the study.
Blinding	Study medication was provided by the pharmacy department. Medication and placebo syringes were identical in terms of colour and shape, and labelled with an unique sample number and study patient identification number. Patients and investigators retained unaware of the initial allocation during the entire trial, including the open-label extension.

Reporting for specific materials, systems and methods

We require information from authors about some types of materials, experimental systems and methods used in many studies. Here, indicate whether each material, system or method listed is relevant to your study. If you are not sure if a list item applies to your research, read the appropriate section before selecting a response.

Materials & experimental systems

n/a	Involved in the study
☒	☐ Antibodies
☒	☐ Eukaryotic cell lines
☒	☐ Palaeontology and archaeology
☒	☐ Animals and other organisms
☐	☒ Clinical data
☒	☐ Dual use research of concern

Methods

n/a	Involved in the study
☒	☐ ChIP-seq
☒	☐ Flow cytometry
☒	☐ MRI-based neuroimaging

Clinical data

Policy information about clinical studies

All manuscripts should comply with the ICMJE guidelines for publication of clinical research and a completed CONSORT checklist must be included with all submissions.

Clinical trial registration

EUDRACT 2015-003223-53

Study protocol

The complete study protocol (all versions) is uploaded in the appendix.

Data collection

We screened patients for enrolment between March 16, 2016 and July 10, 2018. Patients completed baseline visits and started treatments between March 30, 2016 and July 25, 2018, and the last study visit of the last patient in the placebo-controlled phase was April 3, 2019. All data were collected at the outpatient clinic of the Ghent University Hospital, Belgium.

Outcomes

The primary efficacy endpoint was the change in total GUSS(14) from baseline at week 24. The scoring system can change in positive (i.e., more remodelling) or negative direction (i.e., more erosive progression). Target joints were defined as: all proximal and/or distal IP joints in J or E phase on baseline radiographs (except IP1) with presence of inflammatory activity, defined by both clinical soft tissue swelling and ultrasonographic inflammation (i.e., either synovial proliferation or effusion). If several target joints were available, all were included for efficacy analysis.

Secondary endpoints were total GUSS changes from baseline at week 48, and the percentage of new erosive joints by Verbruggen and Veys amongst the baseline pre-erosive joints (i.e., baseline N, S, J joints) per patient at week 48.

Exploratory clinical endpoints and patient-reported outcomes were NRS pain, NRS global assessment of efficacy by patient, tender joint count, swollen joint count, AUSCAN and FIHOA at week 24 and 48.

Changes in ultrasound scores at week 12 for effusion, synovial proliferation, synovitis score and PD signal, and for erosions at week 48, and percentage changes from baseline in bone mineral density at the femoral neck and lumbar spine at week 48 were other exploratory outcomes.
